# Accuracy of Intraoperative Neuromonitoring in the Diagnosis of Intraoperative Neurological Decline in the Setting of Spinal Surgery—A Systematic Review and Meta-Analysis

**DOI:** 10.1177/21925682231196514

**Published:** 2024-03-25

**Authors:** Mohammed Ali Alvi, Brian K. Kwon, Nader Hejrati, Lindsay A. Tetreault, Nathan Evaniew, Andrea C. Skelly, Michael G. Fehlings

**Affiliations:** 1 Institute of Medical Science, University of Toronto, Toronto, ON, Canada; 2 International Collaboration on Repair Discoveries (ICORD), 8166 University of British Columbia, Vancouver, BC, Canada; 3 Department of Orthopaedics, 8166 University of British Columbia, Vancouver, BC, Canada; 4 Division of Neurosurgery, Krembil Neuroscience Centre, Toronto Western Hospital, 7989 University Health Network, Toronto, ON, Canada; 5 Department of Neurology, 12297 NYU Langone Medical Center, New York, NY, USA; 6 McCaig Institute for Bone and Joint Health, Department of Surgery, Orthopaedic Surgery, Cumming School of Medicine, 2129 University of Calgary, Calgary, AB, Canada; 7 Aggregate Analytics, Inc., Fircrest, WA, USA; 8 Division of Neurosurgery and Spine Program, Department of Surgery, University of Toronto, Toronto, ON, Canada

**Keywords:** spinal cord injury, neuro, trauma, intraoperative neurological injury

## Abstract

**Study Design:**

Systematic review and meta-analysis.

**Objectives:**

In an effort to prevent intraoperative neurological injury during spine surgery, the use of intraoperative neurophysiological monitoring (IONM) has increased significantly in recent years. Using IONM, spinal cord function can be evaluated intraoperatively by recording signals from specific nerve roots, motor tracts, and sensory tracts. We performed a systematic review and meta-analysis of diagnostic test accuracy (DTA) studies to evaluate the efficacy of IONM among patients undergoing spine surgery for any indication.

**Methods:**

The current systematic review and meta-analysis was performed using the Preferred Reporting Items for a Systematic Review and Meta-analysis statement for Diagnostic Test Accuracy Studies (PRISMA-DTA) and was registered on PROSPERO. A comprehensive search was performed using MEDLINE, EMBASE and SCOPUS for all studies assessing the diagnostic accuracy of neuromonitoring, including somatosensory evoked potential (SSEP), motor evoked potential (MEP) and electromyography (EMG), either on their own or in combination (multimodal). Studies were included if they reported raw numbers for True Positives (TP), False Negatives (FN), False Positives (FP) and True Negative (TN) either in a 2 × 2 contingency table or in text, and if they used postoperative neurologic exam as a reference standard. Pooled sensitivity and specificity were calculated to evaluate the overall efficacy of each modality type using a bivariate model adapted by Reitsma et al, for all spine surgeries and for individual disease groups and regions of spine. The risk of bias (ROB) of included studies was assessed using the quality assessment tool for diagnostic accuracy studies (QUADAS-2).

**Results:**

A total of 163 studies were included; 52 of these studies with 16,310 patients reported data for SSEP, 68 studies with 71,144 patients reported data for MEP, 16 studies with 7888 patients reported data for EMG and 69 studies with 17,968 patients reported data for multimodal monitoring. The overall sensitivity, specificity, DOR and AUC for SSEP were 71.4% (95% CI 54.8-83.7), 97.1% (95% CI 95.3-98.3), 41.9 (95% CI 24.1-73.1) and .899, respectively; for MEP, these were 90.2% (95% CI 86.2-93.1), 96% (95% CI 94.3-97.2), 103.25 (95% CI 69.98-152.34) and .927; for EMG, these were 48.3% (95% CI 31.4-65.6), 92.9% (95% CI 84.4-96.9), 11.2 (95% CI 4.84-25.97) and .773; for multimodal, these were found to be 83.5% (95% CI 81-85.7), 93.8% (95% CI 90.6-95.9), 60 (95% CI 35.6-101.3) and .895, respectively. Using the QUADAS-2 ROB analysis, of the 52 studies reporting on SSEP, 13 (25%) were high-risk, 10 (19.2%) had some concerns and 29 (55.8%) were low-risk; for MEP, 8 (11.7%) were high-risk, 21 had some concerns and 39 (57.3%) were low-risk; for EMG, 4 (25%) were high-risk, 3 (18.75%) had some concerns and 9 (56.25%) were low-risk; for multimodal, 14 (20.3%) were high-risk, 13 (18.8%) had some concerns and 42 (60.7%) were low-risk.

**Conclusions:**

These results indicate that all neuromonitoring modalities have diagnostic utility in successfully detecting impending or incident intraoperative neurologic injuries among patients undergoing spine surgery for any condition, although it is clear that the accuracy of each modality differs.

PROSPERO Registration Number: CRD42023384158

## Introduction

Intraoperative neurological injury is a feared complication in surgical spinal procedures, with significant medical, social and economic consequences.^1^ The use of intraoperative neurophysiologic monitoring (IONM) has thus been employed to prevent neurological deficits and identify intraoperative maneuvers that can lead to neurological injury, such as in deformity correction or during intramedullary spinal tumor resections.^2,3^ IONM in current practice refers to various techniques used to assess neural system integrity intraoperatively, including somatosensory evoked potentials (SSEP), motor evoked potentials (MEP), D-waves, and electromyography (EMG).^2–4^ The purpose of using IONM is to detect neurophysiological changes during a surgical procedure that could result in neurological deficits.^4,5^ While the value of using IONM is becoming increasingly recognized, a quantitative assessment of the diagnostic accuracy of various IONM modalities is lacking. Moreover, there is no clear consensus on the use of IONM for spinal surgery.

There have been previous systematic reviews with and without meta-analyses, which have attempted to summarize the role of neurophysiologic monitoring for intraoperative spinal cord injury (ISCI).^4,6–16^ However, these reviews have focused on a specific question or have only included studies comparing one modality to another. A comprehensive assessment of diagnostic test accuracy (DTA) of neuromonitoring following the PRISMA-DTA guidelines and GRADE guidelines has yet to be performed.

Key Question: What is the accuracy of neurophysiological monitoring for diagnosis of intraoperative spinal cord injury (ISCI) compared with immediate postoperative clinical assessment?

## Methods

This systematic review and meta-analysis was performed using the Preferred Reporting Items for Systematic Review and Meta-analysis of Diagnostic Test Accuracy Studies (PRISMA-DTA).^17^ The abstract was drafted using the Preferred Reporting Items for a Systematic Review and Meta-analysis of Diagnostic Test Accuracy Studies (PRISMA-DTA) abstract. A comprehensive search was performed using MEDLINE, EMBASE and SCOPUS for all studies assessing the diagnostic accuracy of neuromonitoring, including SSEP, MEP and EMG, either on their own (unimodal) or in combination (multimodal).

### Criteria for Inclusion/Exclusion of Studies in the Review

The criteria for inclusion and exclusion of studies for this systematic review were specified a priori for population, interventions, outcomes, reference standard, timing, and settings/studies (PICOTS) and are listed in Table 1. Only studies reporting raw numbers for True Positives (TP), False Negatives (FN), False Positives (FP) and True Negative (TN) either in a 2 × 2 contingency table or in text were included. Moreover, only studies using postoperative neurologic exam as a reference standard were included.

**Table 1.** Inclusion and Exclusion Criteria: Population, Interventions, Comparators, Outcomes, Timing, and Study Designs.

	Inclusion	Exclusion
Population	• Adolescents (≥12 years to <18 years old) or adults (≥18 years) undergoing any type of spine surgery for any indication or spine-related pathology (including trauma-related pathology, conus injuries, cauda equina injuries) Subpopulations/groups of interest: • Spinal deformity (eg, scoliosis) • Intra-dural tumors vs extra-dural lesions • Degenerative spine lesions	• Patients <11 years old • Patients with new post-operative compression (eg, hematoma, abscess) • Patients with pedicle screw breach • Patients with neurologic deficits due to cranial pathology (eg, stroke)
Intervention	• Neuromonitoring including somatosensory evoked potentials (SSEP), motor evoked potentials (MEP), electromyography (EMG) and multimodal monitoring	
Outcomes	• True positives (a change in monitoring corresponding to a change in postoperative neurologic status) • False positive (a change in monitoring corresponding to no change in postoperative neurologic status) • False negative (no change in monitoring, but a change in postoperative neurologic status • True negative (no change in monitoring corresponding to no change in postoperative neurologic status)	
Reference standard	• Postoperative neurological change/recovery (based on validated measures) - AIS grade - Motor score - Frankel grade	• Pedicle screw breach
Timing	• Immediate post-operative period	
Study design	The review focus on the evidence at least risk for bias • RCTs or comparative observational studies (comparative cohorts, case control studies) • Case series will be included if comparative studies are not available	• Case series with ≤10 patients • Animal studies • Abstracts, editorials, letters • Duplicate publications of the same study that do not report on different outcomes • Single reports from multicenter trials • White papers • Narrative reviews • Proceedings/abstracts from meetings • Articles identified as preliminary reports when results are published in later versions

#### Study Design

Randomized control trials (RCTs) and high-quality prospective comparative cohort studies that control for confounding and met inclusion criteria were included as the primary evidence source. In the absence of high-quality studies, lower quality studies (eg retrospective observational studies) were considered.

### Literature Search Strategies

#### Literature Databases

MEDLINE^®^, EMBASE and SCOPUS were searched using an appropriate search strategy. We included studies published in English and kept track of studies with English abstracts but not fully published in English that appeared to be relevant. Citations suggested by the clinical authors and guideline development group were compared against the *s* criteria for inclusion and exclusion. The search strategy for MEDLINE/EMBASE and SCOPUS is summarized in Table 2.

**Table 2.** Search Strategy.

String #	Search String	Count of articles
1	(neuromonitoring or intraoperative monitoring or neurophysiologic monitoring or neurophsiological monitoring or intraoperative neurophsiologic monitoring or intraoperative neurophysiological monitoring or IONM or SSEP or somatosensory evoked potential or motor evoked potential or MEP or electromyography or electroneuromyography or d-wave or multi-modal monitoring or multimodal neuromonitoring or multimodal intraoperative neuromonitoring or multimodal intraoperative monitoring or multimodal intraoperative neurophysiologic monitoring).mp. [mp = ti, ab, hw, tn, ot, dm, mf, dv, kf, fx, dq, bt, nm, ox, px, rx, ui, sy]	254696
2	Limit 1 to human	201046
3	(Spine surgery or cervical spine or thoracic spine or lumbar spine or lumbosacral spine or thoracolumbar or cervicothoracic or spinal surgery or spin* surg* or spin* deformity or scoliosis or spin* extradural or spin* intradual or extramedullary tumor or intramedullary tumor or spin * tumor or degenerative spine or spin* myelopathy).mp. [mp = ti, ab, hw, tn, ot, dm, mf, dv, kf, fx, dq, bt, nm, ox, px, rx, ui, sy]	347753
4	Limit 3 to human	291603
5	(Sensitivity or sensiti* or specificity or specif* or accuracy* or true positive or true negative or false positive or false negative).mp. [mp = ti, ab, hw, tn, ot, dm, mf, dv, kf, fx, dq, bt, nm, ox, px, rx, ui, sy]	13186005
6	Limit 5 to human	7681782
7	2 and 4 and 6	2574

SCOPUS.

(TITLE-ABS-KEY ( “neuromonitoring” OR “intraoperative monitoring” OR “neurophysiologic monitoring” OR “neurophsiological monitoring” OR “intraoperative neurophsiologic monitoring” OR “intraoperative neurophysiological monitoring” OR “IONM” OR “SSEP” OR “somatosensory evoked potential” OR “motor evoked potential” OR “MEP” OR “electromyography” OR “electroneuromyography” OR “d-wave” OR “multi-modal monitoring” OR “multimodal neuromonitoring” OR “multimodal intraoperative neuromonitoring” OR “multimodal intraoperative monitoring” OR “multimodal intraoperative neurophysiologic monitoring”).

AND.

TITLE-ABS-KEY (“spine surgery” OR “cervical spine” OR “thoracic spine” OR “lumbar spine” OR “lumbosacral spine” OR “thoracolumbar” OR “cervicothoracic” OR “spinal surgery” OR “scoliosis” OR “intradual tumor” OR “extramedullary tumor” OR “intramedullary tumor” OR “spinal tumor” OR “degenerative spine”).

AND.

TITLE-ABS-KEY (“sensitivity” OR “specificity” OR “accuracy” OR “true positive” OR “true negative” OR “false positive” OR “false negative”).

Number of Documents: 854.

Embase Classic+Embase <1947 to 2022 November 17>

Ovid MEDLINE(R) ALL <1946 to November 17, 2022>

#### Publication Date Range

The search included citations from database inception to September 2022.

#### Hand Searching

Reference lists of included studies, relevant systematic reviews, and pertinent gray literature were also evaluated for eligible studies.

### Process for Selecting Studies

All studies retrieved through the search strategy were uploaded to Covidence.^18^ The pre-established criteria above were used by 2 reviewers to screen the titles and abstracts of the citations identified through our searches (MAA and AQ). Any citation deemed not relevant for full-text review was reviewed by a second researcher to assure accuracy and completeness. Each full-text article was independently reviewed for eligibility by 2 team members (MAA and NH). Any disagreements were resolved by consensus. A record of studies excluded at the full-text level with reasons for exclusion was maintained (supplemental material).

Some of the included studies presented data for more than one type of IONM modality. For example, a study utilizing multimodal neuromonitoring presented data for SSEP, MEP and EMG separately, and all of these were included in their respective groups. Moreover, some studies reported data using different thresholds to define neurologic injury. For example, a study presented data for MEP using both a 75% and 50% threshold and both were included.

### Data Abstraction and Data Management

Abstraction of information related to the key question was limited to information needed to answer the questions. General patient characteristics, relevant surgical information, characteristics of neurophysiological monitoring (including any thresholds) as well as metrics of diagnostic accuracy were abstracted.

After studies were selected for inclusion for the key question, standardized data abstraction included the following (at minimum): patient characteristics (age, sex, comorbidities), completeness (AIS) and level of spinal cord injury (SCI), indication for spine surgery (eg, scoliosis, tumor), clinical/pathology characteristics (eg, myelopathy), surgical procedure characteristics (eg approach, levels, instrumentation), adjunctive treatments (eg, steroids, vasopressors), study-related characteristics (eg, sample size, design, control of confounding, timing of follow-up), intervention characteristics (eg, type, such as MEP, SEPP, timing, thresholds) and outcomes with a focus on the primary outcomes related to neurological recovery and adverse events listed in Table 1.

### Assessment of Methodological Risk of Bias of Individual Studies

The risk of bias (ROB) and applicability of included studies was assessed using the quality assessment tool for diagnostic accuracy studies (QUADAS-2).^19^ Four primary domains make up QUADAS-2:

• Patient Selection
• Index Test
• Reference Standard
• Flow and Time

Each domain is evaluated for risk of bias, and the first 3 are evaluated for issues about application. Signaling questions are offered to help with the assessment of bias risk. We also created traffic light and summary plots to illustrate risk of bias for each study using the robvis tool.^20,21^ Each study was classified as either “low risk, some concerns, or high risk.”

### Data Synthesis and Statistical Analysis

The summary statistics and summary line from 4 sets of fundamental data—TP, FP, FN and TN—were used to describe the DTA. Sensitivity, specificity, diagnostic odds ratio (DOR), forest plot, and summary receiver operating characteristic (SROC) curve are examples of representative summary statistics and summary curves, respectively. Sensitivity is calculated using the formula (TP/(TP+FN)), while specificity is calculated using the formula (TN/(TN+FP)). Logit-transformed data are more frequently used than raw data for such proportion-type data. The logit transformation is a technique for modifying the distribution of data in accordance with statistical hypotheses. The lowest and upper bounds of the proportion-type data are 0 and 1, respectively. Their upper and lower limits should be freed by conducting multiplication and log transformations, respectively, to make the data suitable for the assumptions of statistics.

As with pairwise meta-analysis, a suitable model should be chosen in order to determine the DTA's summary statistics. The Moses-Littenberg SROC model,^22,23^ the bivariate model,^24^ and the hierarchical SROC (HSROC) model^25^ are examples of models that take both sensitivity and specificity into account. The Moses-Littenberg model, a relatively straightforward approach developed early on to compute DTA, uses simple linear regression to estimate the SROC. This is comparable to the fixed-effect model used in pairwise meta-analysis and is unable to evaluate study heterogeneity. Additionally, because this model just offers the SORC curve without providing parameter estimates, standard deviation, or confidence intervals, it can only perform restricted analysis and cannot discriminate between within-study and between-study variations in any variations (CIs). The bivariate model and HSROC model were created based on the hierarchical model to address the shortcomings of the Moses-Littenberg model.^26^ When there is no covariate, these 2 models mathematically provide the same value.^27,28^ This is comparable to the pairwise meta-analysis random-effect model. Both models are capable of estimating the heterogeneity, or the variation of studies both within and between studies. In the bivariate model, the sensitivity and specificity for within-study variations are directly modeled by a binominal distribution, while the sensitivity and specificity for between-study variations are assumed by a bivariate normal distribution. Therefore, we followed a bivariate model for performing pooled DTA analysis. Analyses were performed on R-Studio using the *“mada”,*^29^ *“mvtnorm”,*^30^ *“ellipse”,*^31^ *“mvtmeta”,*^32^ *“meta”,*^33^ *“metafor”,*^34^ *“rmeta”*^35^ packages.

### Publication Bias Assessment

DTA meta-analyses differ from conventional intervention meta-analysis in a number of ways, making it more difficult to estimate the likelihood of publication bias. The Egger test is a statistical method for identifying funnel plot asymmetry in conventional meta-analysis.^36^ In order to test the global null hypothesis that “all of the univariate funnel plots for multiple outcomes are symmetric,” Hong et al (2020) first proposed an expanded version of this test for multivariate meta-analysis.^37^ In comparison to the common univariate publication bias test, this overall test contains various outcome information, and the statistical power is often increased. The Hong's test (also known as MSSET) avoids correlation data among various outcomes that are occasionally absent under some circumstances of multivariate meta-analysis. However, for DTA meta-analysis, the Reitsma's bivariate meta-analysis model has all of the correlation data, and since MSSET does not make use of this data, its statistical power may be wasteful.^24^ For the same global null hypothesis, Noma (2020) created alternative generalized Egger tests that successfully take into account the correlation data (called as MSSET2 and MSSET3). Because Noma's tests make use of correlation data, it is anticipated that they will have greater statistical power than the MSSET when applied to DTA meta-analysis.^38^ Using this information, we used the *“MVPBT”* package in R to compute funnel plots and perform statistical tests for asymmetry.^39^

### Grading the Strength of Evidence for Major Comparisons and Outcomes

The overall quality (strength) of evidence (SOE) for the primary (critical) outcomes of neurological recovery and adverse events was assessed based on the application of GRADE, particularly for DTA.^40^ TSOE was initially evaluated by one methodologist and reviewed independently by a second for consistency and validity before the final assessment. Disagreements were resolved by consensus. For a DTA evidence synthesis, RCT and observational prospective/retrospective studies were initially considered to be high quality of evidence; however, the evidence was downgraded based on the aggregate assessment of risk of bias across studies reporting on the outcome, consistency, imprecision, directness, and publication bias. Comparative observational studies begin as low quality of evidence. There are also situations where the observational evidence may be upgraded (eg, large magnitude of effect, presence of dose-response relationship or existence of plausible unmeasured confounders) as described in the AHRQ Methods Guide.^41^

The sSOE was computed for the main diagnostic groups (SSEP, MEP, EMG and multimodal) and also for subgroups. The SOE was assigned an overall grade of high, moderate, low, or very low according to a four-level scale by evaluating and weighing the combined results of the above domains (Table 3).

**Table 3.** Description of the Quality (Strength) of Evidence Grades.

Strength of evidence	Description
High	We are very confident that the estimate of risk lies close to the true effect for this outcome. The body of evidence has few or no deficiencies. We believe that the findings are stable, ie, another study would not change the conclusions
Moderate	We are moderately confident that the estimate of risk lies close to the true effect for this outcome. The body of evidence has some deficiencies. We believe that the findings are likely to be stable, but some doubt remains
Low	We have limited/low confidence that the estimate of risk lies close to the true effect for this outcome. The body of evidence has major or numerous deficiencies (or both). We believe that additional evidence is needed before concluding either that the findings are stable or that the estimate of effect is close to the true effect
Very low	We have extraordinarily little confidence in the estimate for this outcome. The body of evidence has unacceptable deficiencies

## Results

### Study Selection

**Figure 1.**
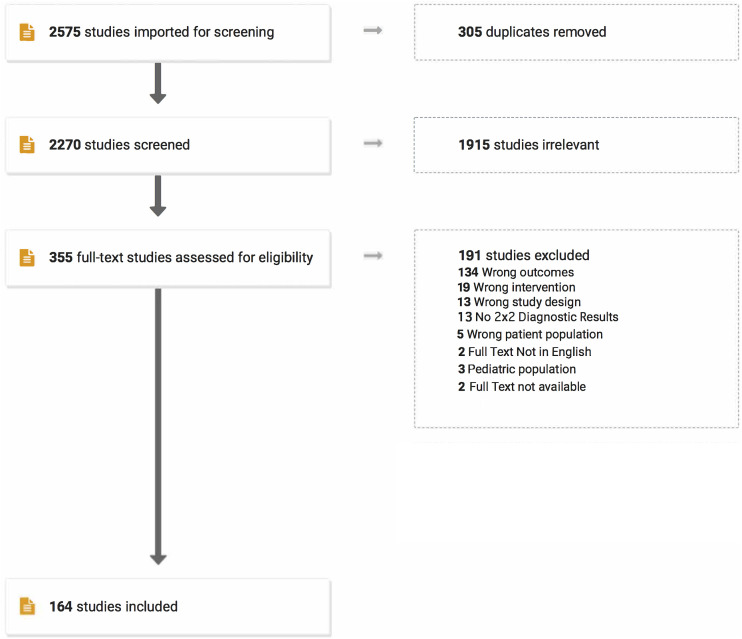
PRISMA-DTA Flowchart for selection of studies.

The search strategy using EMBASE, MEDLINE and SCOPUS yielded a total of 2270 articles after removing 305 duplicates. Of these, 1915 abstracts were considered irrelevant. The full texts of the remaining 355 articles were reviewed. Of these, 189 were excluded. A flowchart summarzing the selection of studies is provided in Figure 1. Details related to excluded studies, including reasons for exclusion, are presented in Supplementary Table 1. A total of 164 studies^2,5,42–52,^ ^52–62,^ ^62–72,^ ^72–82,^ ^82–92,^ ^92–102,^ ^102–112,^ ^112–122,^ ^122–132,^ ^132–142,^ ^142–152,^ ^152–162,^ ^162–172,^ ^172–182,^ ^182–192,^ ^192–203^ consisting of 99937 patients were included. Of the 164 studies, 16 (9.75%) were prospective while 148 (90.25%) were retrospective. In terms of disease group, most studies included patients with mixed pathology (29.87%, n = 49), followed by deformity (26.83%, n = 44), degenerative disease (21.95%, n = 36), tumors (17.68%, n = 29), trauma (1.83%, n = 3), congenital diseases (1.2%, n = 2) and AVM (.6%, n = 1). Most studies featured centers/hospitals from the United States (35.36%, n = 58), followed by Japan (15.85%, n = 26), China (9.1%, n = 15), Korea, UK (5.5% each, n = 9), Canada, Switzerland (4.9% each, n = 8), and others. Several studies consisted of only adult patients (50%, n = 82), while others included both adolescent and adult patients (34.7%, n = 57) or only adolescent patients (9.1%, n = 15). Ten studies (6%) did not specify patient age. Of the 164 studies, 52 studies (31.7%) presented data for SSEP, 75 studies (45.7%) presented data for MEP, 16 studies (9.75%) presented data for EMG, and 69 studies (42.07%) presented data for multimodal neuromonitoring. These study characteristics are summarized in Table 4.

**Table 4.** Characteristics of included studies.

First author and year	Study type	Country/Region	Total patient count	Disease type	Procedures	Population (adult, adolescent or both)	Neuromonitoring type	Data presented for
Abdelkader 2019	Prospective	Egypt	50	Mixed: Degenerative (stenosis, disc, OPLL), trauma, deformity (scoliosis), tumor	Extramedullary manipulations, decompression of an epidural abscess or neoplasm, removal of intramedullary tumor or arteriovenous malformation, or spine correction procedures	Both	SSEP	SSEP
Accadbled 2006	Retrospective	France	191	Deformity	Corrective surgery	Both	Multimodal: SSEP and MEP	Multimodal
Almahdy 2021	Retrospective	USA	50	Mixed: Tumor, abscess, AVM, degenerative (stenosis, disc), trauma	Non- instrumented posterior cervical (C3-6) laminectomy, tumor resection	Both	Multimodal: SSEP and MEP	Multimodal
Avila 2013	Retrospective	USA	208	Tumor: Metastasis	Tumor resection	Both	Multimodal: SSEP, MEP, EMG	MEP
Bhagat 2015	Retrospective	UK	354	Deformity	Corrective surgery	Both	Multimodal: SSEP and MEP	Multimodal, MEP, SSEP
Bhalodia 2013	Retrospective	USA	229	Mixed	Cervical spine operations involving the C-4 and/or C-5 levels	Adult	Multimodal: SSEP, MEP and EMG	Multimodal, MEP
Bir 2021	Retrospective	India	31	Mixed: Degenerative (stenosis, disc, OPLL), trauma, deformity (scoliosis), tumor	Not specified	Adult	Multimodal: MEP, D-wave	MEP
Bose 2002	Retrospective	USA	61	Degenerative	Lumbar fusion	Adult	EMG	EMG
Bose 2007	Retrospective	USA	238	Degenerative	Anterior cervical spinal procedures included single-level discectomies, multilevel discectomies, corpectomies, and stabilization of odontoid fractures	Adult	Multimodal: SSEP, MEP and EMG	Multimodal, MEP, SSEP, EMG
Calancie 1998	Retrospective	USA	34	Mixed: Tumor, spinal deformity (scoliosis, kyphosis, or displaced fracture), arteriovenous fistula, tethered cord	Not specified	Both	Multimodal: MEP and EMG	MEP
Calancie 2008	Retrospective	USA	859	Mixed: Tumor, tethered cord, orthopedic, vascular and cyst	Decompression and/or stabilization of the cervical, thoracic, or thoracolumbar spine	Adult	Multimodal: MEP and EMG	Multimodal, MEP
CaminoWillhuber 2021	Retrospective	Argentina	137	Degenerative	Percutaneous cement discoplasty	Adult	EMG	EMG
Chandanwale 2008	Retrospective	India	112	Trauma	Thoracic or lumbar surgery	Both	SSEP	SSEP
Chen 2015	Retrospective	China	426	Mixed: Degenerative, deformity and tumor	Unclear	Both	Multimodal: SSEP and MEP	Multimodal
Chen Yu 2021	Retrospective	China	113	Degenerative	TLIF	Adult	Multimodal: SSEP, MEP and EMG; multimodal: MEP and EMG	Multimodal, MEP, EMG, SSEP
Choi 2014	Retrospective	Korea	76	Tumor: Intramedullary	Not specified	Adult	Multimodal: SSEP and MEP	MEP
Chung 2009	Prospective	USA	230	Degenerative	Lumbar decompression, discectomy and fusion	Adult	SSEP	SSEP
Clark 2013	Retrospective	USA	140	Degenerative	Anterior or posterior cervical surgery	Adult	MEP	MEP
Clark 2016	Retrospective	USA	144	Mixed: Tumor, infection, trauma, inflammatory	Anterior or posterior cervical surgery	Adult	MEP	MEP
Cornips 2017	Retrospective	Netherlands	77	Degenerative: Thoracic disc herniation	Anterior transthoracic surgery	Adult	MEP	MEP
Costa 2007	Retrospective	Italy	52	Mixed: Trauma, tumor, degenerative (spondylosis), scoliosis, AVM, echinicoccus	Not specified	Adult	Multimodal: SSEP and MEP	Multimodal
Costa 2013	Retrospective	Italy	101	Mixed: Tumor (intra and extra medullary) and degenerative	Not specified	Both	Multimodal: SSEP and MEP	Multimodal
Dauleac 2022	Retrospective	France	99	Mixed: Tumor, degenerative, tethered cord	Not specified	Adult	Multimodal:SSEP and MEP	Multimodal
Delgado-lópez 2022	Retrospective	Spain	103	Degenerative	Anterior cervical surgery	Adult	Multimodal: SSEP and MEP	Multimodal
Deutsch 2000	Retrospective	USA	44	Tumor	Anterior thoracic	Adult	SSEP	SSEP
Dicindio 2003	Retrospective	USA	68	Deformity	Not specified	Adolescent	Multimodal: SSEP and MEP	Multimodal
Ding 2008	Retrospective	China	76	Degenerative	ACDF and laminoplasty	Adult	SSEP	SSEP
Dinner 1986	Retrospective	USA	220	Mixed	Not specified	Adult	SSEP	SSEP
Duncan 2012	Retrospective	USA	115	Degenerative	Lumbar fusion	Adult	Multimodal: SSEP, MEP and EMG	Multimodal, SSEP
Eggspuehler (cervical) 2007	Retrospective	Switzerland	246	Degenerative	Cervical surgery	Adult	Multimodal: SSEP, MEP and EMG	Multimodal
Eggspuehler (deformity) 2007	Retrospective	Switzerland	217	Deformity	Corrective surgery	Adult	Multimodal: SSEP, MEP and EMG	Multimodal
Eggspuehler (thoracic) 2007	Retrospective	Switzerland	36	Degenerative	Thoracic surgery	Adult	Multimodal: SSEP, MEP and EMG	Multimodal
Feng 2012	Retrospective	China	176	Deformity	Corrective surgery	Both	Multimodal:SSEP and MEP	MEP
Ferguson 2014	Retrospective	USA	519	Deformity	Corrective surgery	Adult	Multimodal:SSEP and MEP	Multimodal
Feyissa 2015	Retrospective	USA	19	Tumor	Thoracic Laminectomy, Facetectomy, vertebrectomy	Adult	SSEP	SSEP
Forster 2012	Retrospective	Germany	203	Tumor	Not specified	Adult	Multimodal: SSEP and MEP	Multimodal, MEP
Fujiwara 2016	Retrospective	Japan	160	Degenerative	Laminoplasty	Adult	MEP	MEP
Funaba 2021	Retrospective	Japan	1176	Degenerative	Laminoplasty, ACDF, posterior fusion, circumferential fusion	Adult	MEP	MEP
Funaba 2022	Retrospective	Japan	2476	Mixed: Tumor, infection, trauma, deformity, inflammatory	Decompression, fusion with instrumentation	Both	MEP	MEP
Gavaret 2011	Retrospective	France	300	Mixed: Trauma, tumor, degenerative (spondylosis), scoliosis	Not specified	Both	Multimodal: SSEP and D-wave	SSEP, multimodal
Ghadirpour 2015	Retrospective	Italy	68	Tumor: Extramedullary	Tumor resection	Adult	Multimodal: SSEP, MEP and D-wave	Multimodal
Ghadirpour 2019	Retrospective	Italy	108	Tumor	Not specified	Adult	Multimodal: SSEP MEP and D-wave	Multimodal
Gundanna 2003	Retrospective	USA	186	Mixed: Degenerative, scoliosis	Posterior fusion	Adult	SSEP	SSEP
Gunnarsson 2004	Retrospective	Canada	213	Mixed: Tumor, spinal deformity (scoliosis, kyphosis, or displaced fracture), degenerative, tethered cord	Decompression, microdiscectomy, tumor resection/removal, tethered cord release, others, instrumented fusions	Adult	Multimodal: SSEP and EMG	Multimodal
Harel 2017	Retrospective	Israel	41	Tumor	Not specified	Adult	Multimodal: SSEP, MEP and EMG	Multimodal
Hawary 2006	Retrospective	Canada	41	Deformity	Decompression, release, resection	Adolescent	Multimodal: SSEP and MEP	Multimodal
Hilibrand 2004	Retrospective	USA	427	Degenerative	Anterior, posterior and circumferential cervical surgery	Adult	Multimodal: SSEP and MEP	MEP
Hsu 2008	Retrospective	Australia	144	Deformity	Correction	Both	MEP and CMAP	Multimodal, MEP
Hu 2011	Retrospective	Hong Kong	191	Mixed	Not specified	Adult	SSEP	SSEP
Huang 2016	Retrospective	China	89	Deformity	Not specified	Both	SSEP	SSEP
Hyun 2009	Retrospective	Korea	17	Tumor	Not specified	Adult	Multimodal: SSEP and MEP	Multimodal, MEP
Ille 2021	Retrospective	Germany	71	Tumor	Not specified	Adult	Multimodal: SSEP, MEP and EMG	Multimodal, MEP
Ishida 2019	Retrospective	USA	103	Tumor	Not specified	Adult	Multimodal: SSEP, MEP and EMG	Multimodal
Ito 2012	Retrospective	Japan	884	Mixed: Tumor, degenerative (spondylosis), scoliosis	Not specified	Not specified	MEP	MEP
Ito 2013	Retrospective	Egypt	295	Mixed	Not specified	Adult	Multimodal: SSEP and MEP	MEP, SSEP
Izumi 1993	Retrospective	Japan	20	Tumor	Not specified	Not specified	D-wave	MEP
Jarvis 2013	Retrospective	Canada	37	Deformity	3-Column posterior spinal osteotomies	Adolescent	Multimodal: SSEP and MEP	MEP
Jin 2015	Retrospective	Korea	30	Tumor	Not specified	Adult	Multimodal: MEP and EMG	Multimodal
Kamerlink 2010	Retrospective	USA	281	Deformity	Spinal fusion	Both	Multimodal: SSEP and MEP	Multimodal
Kelleher 2008	Prospective	Canada	1055	Mixed	Not specified	Adult	Multimodal: SSEP, MEP and EMG	SSEP, MEP
Khan 2006	Retrospective	USA	508	Mixed	Cervical corpectomy	Adult	SSEP	SSEP
Kim 2007	Retrospective	USA	52	Degenerative	Cervical ACDF, laminectomy, posterior fusion	Adult	MEP	MEP
Kim 2012	Retrospective	Korea	209	Mixed: Degenerative (stenosis, disc, OPLL), trauma, deformity (scoliosis), tumor	Not specified	Adult	MEP	MEP
Kim 2014	Retrospective	Korea	22	Tumor: Intramedullary	Not specified	Adult	Multimodal: SSEP and MEP	Multimodal
Kim 2017	Retrospective	Korea	200	Degenerative	ACDF	Adult	Multimodal: SSEP and MEP	Multimodal
Kim 2019	Retrospective	USA	275	Degenerative	TLIF	Adult	Multimodal: SSEP, MEP and EMG	Multimodal, MEP, EMG, SSEP
Kim 2021	Retrospective	Korea	196	Degenerative	ACDF	Adult	Multimodal: SSEP, MEP and EMG	Multimodal, MEP, EMG, SSEP
Kobayashi 2014	Retrospective	Japan	959	Mixed: Degenerative (stenosis, disc, OPLL), deformity (scoliosis), tumor	Not specified	Not specified	MEP	MEP
Kobayashi 2017	Prospective	Japan	68	Deformity	Corrective surgery	Adolescent	MEP	MEP
Kobayashi 2018	Retrospective	Japan	83	Mixed: Degenerative (stenosis, disc, OPLL), deformity (scoliosis), tumor	Not specified	Both	MEP	MEP
Kobayashi 2018	Retrospective	Japan	70	Mixed: Degenerative (stenosis, disc, OPLL), deformity (scoliosis), tumor	Not specified	Both	Multimodal: SSEP, MEP and D-wave	MEP, multimodal
Kobayashi 2019	Retrospective	Japan	159	Mixed: Degenerative (stenosis, disc, OPLL), tumor	Thoracic surgery	Both	MEP	MEP
Kobayashi 2021	Prospective	Japan	3560	Mixed: Degenerative (stenosis, disc, OPLL), deformity (scoliosis), tumor	Not specified	Both	MEP	MEP
Kobayashi 2021	Prospective	Japan	233	Tumor: Intramedullary	Not specified	Both	MEP	MEP
Kobayashi 2022	Prospective	Japan	3625	Mixed: Degenerative (stenosis, disc, OPLL), deformity (scoliosis), tumor	Not specified	Both	MEP	MEP
Koffie 2022	Retrospective	USA	498	Degenerative	Posterior cervical laminectomy or laminoplasty with or without fixation and fusion	Adult	Multimodal: SSEP and MEP	MEP, SSEP
Korn 2015	Retrospective	Israel	100	Tumor: Extramedullary	Not specified	Both	Multimodal: SSEP, MEP and EMG	Multimodal
Krishnakumar 2011	Retrospective	India	52	Deformity	Not specified	Both	Multimodal: SSEP and MEP	Multimodal, SSEP, MEP
Kumar 2019	Retrospective	UK	128	Tumor: Metastasis	Not specified	Adult	Multimodal: SSEP, MEP and EMG	Multimodal
Kundnani 2010	Prospective	Singapore	354	Deformity	Corrective surgery	Adolescent	Multimodal: SSEP and MEP	MEP
Kurokawa 2017	Retrospective	Japan	58	Tumor: Intramedullary	Not specified	Both	MEP	MEP
Lakomkin 2017	Retrospective	USA	52	Tumor	Not specified	Both	Multimodal: SSEP and MEP	Multimodal
Langeloo 2003	Retrospective	Netherlands	145	Deformity	Anterior or posterior surgery	Both	MEP	MEP
Lau 2019	Retrospective	USA	242	Deformity	Lumbar pedicle subtraction osteotom	Adult	MEP	MEP
Lau 2022	Retrospective	USA	56	Deformity	Lower cervical and upper thoracic posterior-based three-column osteotomies	Adult	Multimodal: SSEP, MEP and EMG	Multimodal
Lee 2016	Retrospective	Korea	146	Mixed: Degenerative, congenital, tumor, trauma	Goel's technique: Intraoperative CVJ realignment through a rod and screw system	Adult	Multimodal: SSEP and MEP	Multimodal
Leung 2005	Retrospective	UK	871	Deformity	Anterior surgery	Not specified	Multimodal: SSEP and MEP	SSEP
Li 2012	Retrospective	USA	200	Mixed: Degenerative (stenosis, disc, OPLL), trauma, deformity (scoliosis), tumor	Anterior cervical surgery	Adult	Multimodal: SSEP, MEP and EMG	Multimodal
Li 2018	Retrospective	China	55	AVM	Not specified	Both	Multimodal: SSEP, MEP and EMG	Multimodal, MEP, SSEP
Lieberman 2008	Retrospective	USA	35	Deformity	Corrective surgery	Both	Multimodal: MEP, D-wave	MEP, multimodal
Liu 2022	Retrospective	China	326	Degenerative	Posterior thoracic Decompression surgery	Adult	Multimodal: SSEP and MEP	Multimodal
Loder 1991	Retrospective	USA	52	Deformity	Not specified	Both	SSEP	SSEP
Lubitz 1999	Retrospective	USA	60	Deformity	Not specified	Adolescent	MEP	MEP
Luk 2001	Retrospective	Hong Kong	30	Deformity	Not specified	Adolescent	Multimodal: CSEP, SSEP, MEP	MEP, SSEP
MacDonald 2007	Retrospective	Saudi Arabia	206	Mixed: Degenerative (stenosis, disc, OPLL), trauma, deformity (scoliosis), tumor	Thoracolumbar surgeries	Both	Multimodal: SSEP and MEP	Multimodal
Makarov 2012	Retrospective	USA	233	Deformity	External fixation procedures	Adolescent	SSEP	SSEP
Manninen 1998	Retrospective	Canada	309	Mixed: Tumor, abscess, degenerative (stenosis, disc), trauma	Corrective surgery, laminectomy, fusion	Both	SSEP	SSEP
Melachuri 2017	Retrospective	USA	1036	Degenerative	Posterior spinal fusions	Adult	SSEP	SSEP
Melachuri 2019	Retrospective	USA	1057	Degenerative	Lumbar interbody fusions	Adult	SSEP	SSEP
Melachuri 2020	Retrospective	USA	771	Mixed	Thoracic decompression and fusion	Adult	SSEP	SSEP
Melachuri 2021	Retrospective	USA	179	Mixed	Mixed	Adult	Multimodal: SSEP and EMG	Multimodal, EMG
May 2006	Retrospective	UK	182	Mixed: Degenerative (stenosis, disc, OPLL), trauma, deformity (scoliosis), tumor	Anterior or posterior cervical surgery	Adult	SSEP	SSEP
Meyer 1988	Retrospective	USA	150	Trauma	Thoracic and lumbar spine internal fixation	Both	SSEP	SSEP
Miller 2019	Retrospective	USA	125	Deformity	Not specified	Adult	MEP	MEP
Mochida 1997	Retrospective	Japan	32	Mixed: Tumor, AVM, degenerative (OPLL), tethered cord	Thoracic and thoracolumbar surgery	Not specified	MEP	MEP
Muramoto 2013	Retrospective	Japan	80	Mixed: Degenerative, tumor, trauma, deformity	Thoracic spine surgery	Adult	Multimodal: SSEP, MEP and D-wave	MEP
Muramoto 2014	Retrospective	Japan	37	Tumor: Intramedullary	Various	Both	MEP	MEP
Neira 2016	Retrospective	Canada	296	Deformity	Spinal fusion	Adolescent	Multimodal: SSEP and MEP	Multimodal, SSEP
Noonan 2002	Retrospective	USA	165	Deformity	Corrective surgery	Adolescent	Multimodal: SSEP and MEP	Multimodal
Noordeen 1997	Retrospective	UK	99	Deformity	Not specified	Adolescent	SSEP	SSEP
Oya 2017	Retrospective	USA	135	Degenerative	Laminoplasty	Adult	Multimodal: SSEP, MEP and EMG	EMG, MEP
Padberg 1996	Retrospective	USA	74	Mixed: Degenerative, congenital, trauma	Anterior, posterior and circumferential fusion	Both	SSEP	SSEP
Padberg 1998	Retrospective	USA	500	Deformity	Instrumentation	Both	Multimodal: SSEP and MEP	Multimodal
Papastefanou 2000	Retrospective	UK	442	Mixed: Degenerative, trauma, tumor, deformity	Not specified	Both	SSEP	SSEP
Paradiso 2006	Prospective	Canada	44	Adult tethered cord	Detethering procedures	Adult	Multimodal: SSEP and EMG	EMG, SSEP, MEP
Park 2011	Retrospective	USA	29	Deformity	Anterior, posterior and circumferential fusion	Adult	Multimodal: SSEP, MEP and EMG	EMG, SSEP, MEP
Park 2018	Retrospective	Korea	34	Tumor: Intramedullary	Not specified	Both	Multimodal: SSEP, MEP and EMG	SSEP, MEP
Pastorelli 2011	Retrospective	Italy	172	Deformity	Posterior fusion with instrumentation surgery, anterior spinal Fusion by thoracotomy, combined anterior and posterior Approach	Adult	Multimodal: SSEP and MEP	Multimodal
Pastorelli 2015	Retrospective	Italy	40	Deformity	Posterior fusion with instrumentation surgery	Adolescent	Multimodal: SSEP and MEP	Multimodal
Pelosi 2002	Retrospective	UK	97	Mixed: Degenerative, trauma, deformity, infection	Anterior or posterior surgery	Both	Multimodal: SSEP and MEP	Multimodal
Platabello 2015	Retrospective	Spain	75	Mixed: Degenerative, tumor, trauma	Anterior and posterior cervical	Adult	Multimodal: SSEP, MEP and EMG	Multimodal
Qiu 2022	Retrospective	China	87	Chiari malformation, syringomyelia, split-cord malformation, and tethered cord syndrome	Posterior spinal fusion	Both	Multimodal: SSEP and MEP	SSEP, MEP
Quinones 2005	Retrospective	USA	28	Tumor: Intramedullary	Not specified	Adult	MEP	MEP
Quraishi 2009	Retrospective	Canada	102	Deformity	Instrumented fusion, Decompression, Osteotomies, PLIF, ALIF	Adult	Multimodal: SSEP, MEP and EMG	Multimodal, EMG
Ruschel 2021	Retrospective	Brazil	47	Tumor: Intramedullary	Not specified	Adult	Multimodal: SSEP, MEP and D-wave	EMG
Sakaki 2012	Retrospective	Japan	350	Degenerative	ACDF, laminoplasty, laminectomy, posterior fusion	Not specified	MEP	MEP
Schar 2017	Retrospective	Switzerland	17	Degenerative: L5–S1 isthmic spondylolisthesis	Instrumented transforaminal lumbar interbody fusion	Both	Multimodal: SSEP, MEP and EMG	Multimodal
Schwartz 2007	Retrospective	USA	38	Deformity	Anterior, posterior and circumferential fusion	Both	Multimodal: SSEP and MEP	Multimodal
Skaggs 2009	Retrospective	USA	1736	Deformity	Not specified	Both	Multimodal: SSEP and MEP	Multimodal
Skinner 2005	Retrospective	USA	13	Tumor: Intramedullary	Not specified	Both	EMG	EMG
Smith 2006	Retrospective	USA	577	Degenerative: Non-myelopathy:cervical stenosis, radiculopathy, herniated nucleus pulposus, junctional stenosis, Or nonunion from prior surgery	ACDF	Adult	SSEP	SSEP
Stechison 1995	Retrospective	USA	150	Mixed: Degenerative, trauma, tumor, deformity	Various	Not specified	SSEP	SSEP
Sutter 2007	Retrospective	Switzerland	1017	Mixed: Degenerative, deformity and tumor	Various	Both	Multimodal: SSEP, MEP and EMG	Multimodal, EMG
Sutter 2019	Retrospective	Switzerland	2728	Mixed: Degenerative, deformity and tumor	Not specified	Both	Multimodal: SSEP, MEP and EMG	Multimodal, MEP
Sutter I 2007	Retrospective	Switzerland	109	Tumor	Not specified	Both	Multimodal: SSEP, MEP and EMG	Multimodal
Sutter II 2007	Retrospective	Switzerland	409	Degenerative	Not specified	Both	Multimodal: SSEP, MEP and EMG	Multimodal
Takahashi 2021	Prospective	Japan	1934	Mixed: Degenerative (stenosis, disc, OPLL), trauma, deformity (scoliosis), tumor	Not specified	Adult	Multimodal: SSEP, MEP and D-wave	MEP
Tanaka 2015	Retrospective	Japan	196	Degenerative	ACDF, laminoplasty, lumbar fusion, lumbar laminectomy, discectomy, fenestration	Not specified	MEP	MEP
Taylor 2021	Prospective	USA	540	Degenerative	Cervical ACDF, laminectomy, posterior fusion	Not specified	Multimodal: SSEP and MEP	Multimodal
Thirumala 2014	Retrospective	USA	477	Deformity	Corrective surgery	Adolescent	SSEP	SSEP
Tiruchelvarayan 2013	Retrospective	Singapore	13	Tumor: Intramedullary	Laminectomy with or without fixation	Both	Multimodal: SSEP and MEP	Multimodal
Tohmeh 2022	Prospective	USA	59	Mixed: Degenerative, deformity	Transpsoas lateral lumbar interbody fusion	Adult	SSEP	SSEP
Tong Yu 2017	Prospective	China	12	Deformity	Corrective surgery	Adolescent	Multimodal: SSEP, MEP and EMG	Multimodal
Traba 2018	Retrospective	Spain	203	Degenerative	TLIF, XLIF	Adult	Multimodal: SSEP, MEP and EMG	MEP
Tsirikos 2004	Retrospective	UK	82	Trauma	Posterior fusion, cervical laminectomy, anterior corpectomy, ACDF	Both	SSEP	SSEP
Tsirikos 2020	Retrospective	UK	1155	Deformity	Posterior closing wedge osteotomies	Both	Multimodal: SSEP and MEP	Multimodal, SSEP
Ushirozako 2018	Retrospective	Japan	295	Deformity	Posterior corrective surgery, with 3-column osteotomies, with posterior column resections, after LLIF	Adult	MEP	MEP
Ushirozako 2019	Retrospective	Japan	703	Mixed: Degenerative (stenosis, disc, OPLL), trauma, deformity (scoliosis), tumor	Not specified	Adult	MEP	MEP
Ushirozako 2020	Retrospective	Japan	393	Deformity	Corrective surgery	Both	MEP	MEP
Ushirozako 2021	Retrospective	Japan	428	Tumor	Not specified	Adult	MEP	MEP
Van der wal 2021	Retrospective	Netherlands	78	Tumor: Extramedullary	Not specified	Both	Multimodal: SSEP and MEP	Multimodal
Velayutham 2016	Retrospective	India	300	Tumor	Not specified	Both	MEP	MEP
Vitale 2010	Retrospective	USA	162	Deformity	Not specified	Both	Multimodal: SSEP and MEP	Multimodal
Wang 2016	Retrospective	China	117	Deformity	Not specified	Both	MEP	MEP
Wang 2016	Retrospective	China	86	Degenerative	Posterior laminoplasty or laminectomy surgery	Both	MEP	MEP
Welch 1997	Retrospective	USA	32	Degenerative	Lumbar fusion	Adult	EMG	EMG
Wilent 2019	Retrospective	USA	40919	Degenerative	Extradural cervical surgery	Adult	Multimodal: SSEP, MEP and EMG	Multimodal, MEP
Wilent 2020	Retrospective	USA	4386	Degenerative	Extradural posterior lumbosacra	Adult	Multimodal: SSEP, MEP and EMG	MEP, EMG, SSEP
Xu 2011	Retrospective	USA	57	Degenerative	ACDF	Adult	Multimodal: SSEP and MEP	MEP, SSEP
Yang 1994	Prospective	China	11	Mixed: Degenerative (stenosis, disc, OPLL), trauma, deformity (scoliosis), tumor	Various	Adult	Multimodal: SSEP and MEP	MEP, SSEP
Yoshida 2018	Prospective	Japan	2867	Mixed: Degenerative (stenosis, disc, OPLL), trauma, deformity (scoliosis), tumor	Various	Not specified	MEP	MEP
Yoshida 2022	Prospective	Japan	311	Deformity	ALIF, LLIF, PLIF, TLIF	Adult	MEP	MEP
Zhuang 2014	Retrospective	China	1162	Deformity	Pedicle subtraction osteotomy, smith-petersen osteotomy, vertebral column resection	Both	Multimodal: SSEP and MEP	MEP
Zuccaro 2017	Retrospective	USA	806	Deformity	Posterior spinal fusion, vertebral body stapling, vertebral column resection	Adolescent	Multimodal: SSEP and MEP	MEP

## SSEP

**Figure 2.**
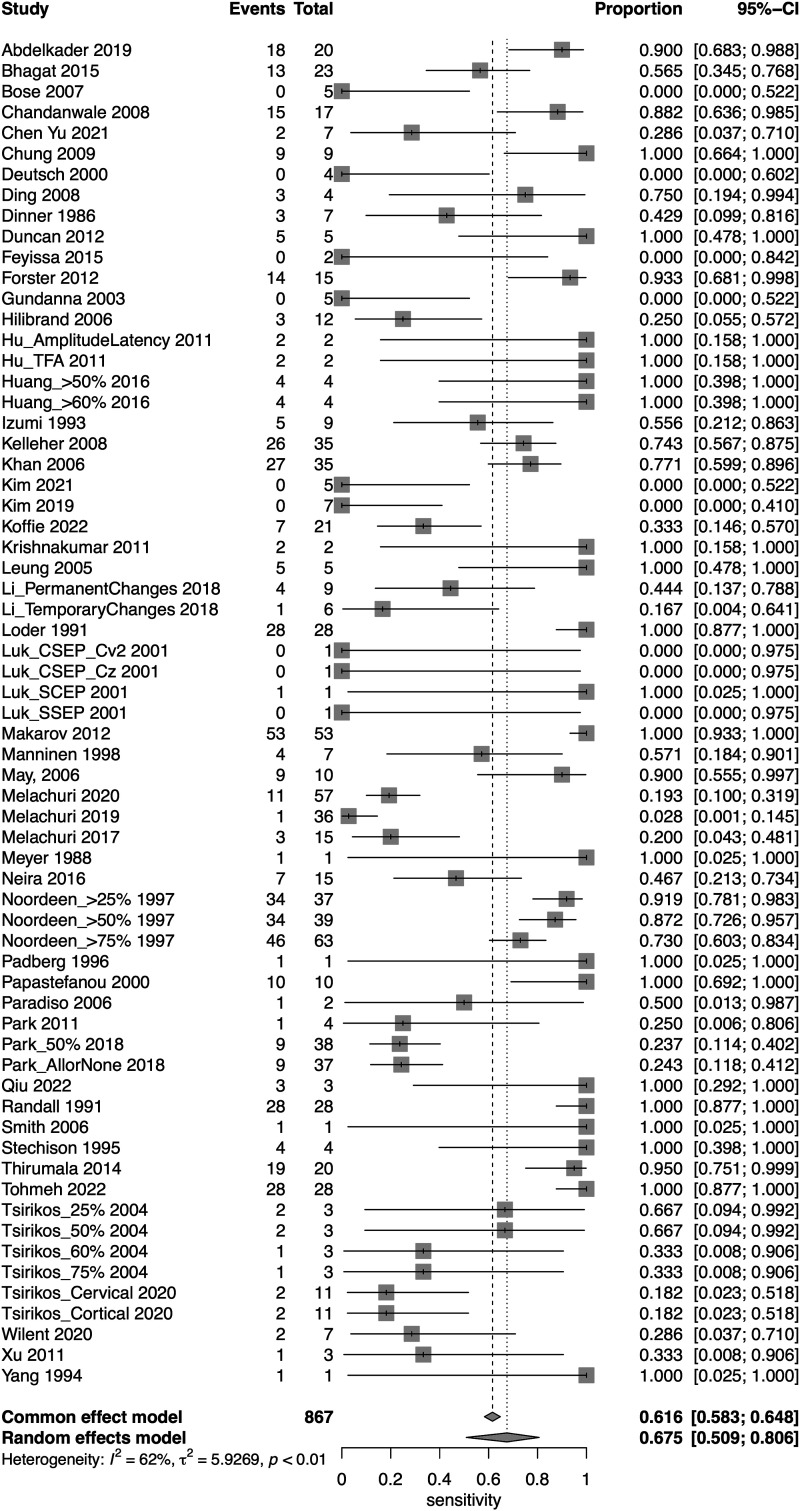
Forest plot for sensitivity of SSEP.

**Figure 3.**
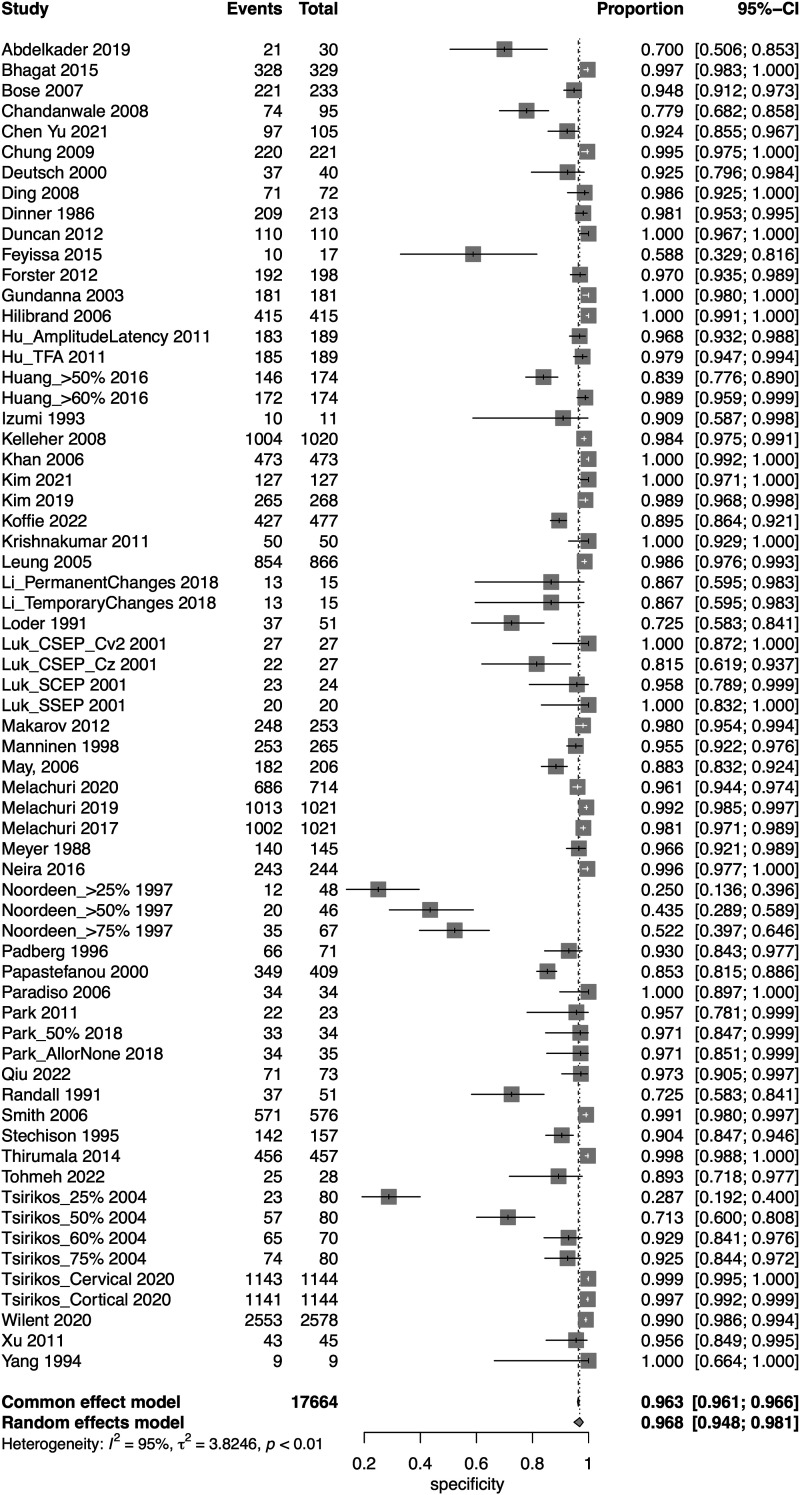
Forest plot for specificity of SSEP.

**Figure 4.**
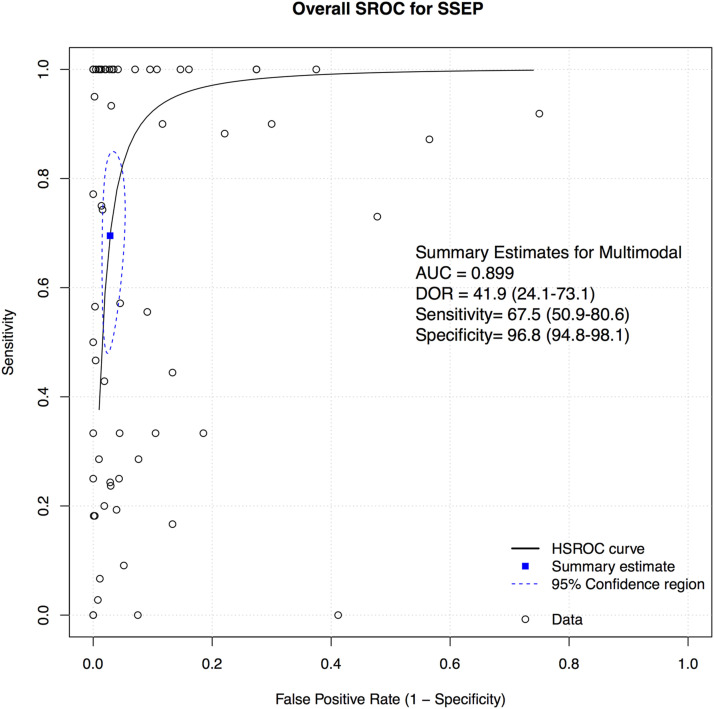
Overall sROC plot for SSEP.

A total of 52 studies presented data for SSEP on a total of 18,076 patients. Overall, the sensitivity of SSEP was 67.5% (95% CI 50.9-80.6, Heterogeneity: I2 = 62%, τ2 = 5.9269, *P* < .01) (Figure 2), while the specificity was 96.8% (95% CI 94.8-98.1, Heterogeneity: I2 = 95%, τ2 = 3.8246, *P* < .01) (Figure 3). Overall, the AUC value was .899, while the DOR was 41.9 (95% CI 24.1-73.1) (Figure 4).

We also performed subgroup analysis for various thresholds for IONM alerts, different reported disease groups, and different regions.

Subgroup Analyses:

1. Thresholds:

The most commonly reported threshold for alert was 50% (n = 43 studies), followed by 60% (n = 4 studies), 25%, 75% (n = 2 studies each) and “all or none” (n = 1 study). Seven studies either reported a different threshold/alert criterion or did not report the actual alert criterion in explicit details; these were classified under “other”. The pooled sensitivities for the 25% threshold, 50% threshold, 60% threshold, 75% threshold, “all or none” alert and other threshold were 90% (95% CI 76.2-96.2, Heterogeneity: I2 = 38%, τ2 = 0, *P* = .20), 71.6% (95% CI 49.5-86.6 Heterogeneity: I2 = 62%, τ2 = 8.4139, *P* < .01, Heterogeneity: I2 = 0%, τ2 = 2.5549, *P* = .99), 62.9% (95% CI 12.9-95.1, Heterogeneity: I2 = 0%, τ2 = 2.55, *P* = .99), 71.2% (95% CI 59.2-80.8, Heterogeneity: I2 = 45%, τ2 = 0, *P* = .18), 24.3% (95% CI 11.8-41.2, Heterogeneity: I2 = 45%, τ2 = 0, *P* = .1) and 41.2% (95% CI 28.6-55, Heterogeneity: I2 = 0%, τ2 = 0, *P* = .93), respectively (Supplemental Figure 1(a)). The pooled specificities for the 25% threshold, 50% threshold, 60% threshold, 75% threshold, “all or none” alert and other threshold were 27.3% (95% CI 20.3-35.7, Heterogeneity: I2 = 0%, τ2 = 0, *P* = .65), 97.5% (95% CI 95.6-98.6, Heterogeneity: I2 = 94%, τ2 = 3.5177, *P* < .01), 98.5% (90.5-99.8, Heterogeneity: I2 = 48%, τ2 = 3.1291, *P* = .12), 78.4% (95% CI 11.5-88.4, Heterogeneity: I2 = 96%, τ2 = 1.4229, *P* < .01), 97.1% (95% CI 85.1-99.9) and 95.3% (95% CI 92.3-97.2, Heterogeneity: I2 = 73%, τ2 = .2627, *P* < .01), respectively (Supplemental Figure 1(b)).

2. Disease Group:

In terms of disease group, most studies presenting data for SSEP consisted of patients with mixed diseases (n = 15), followed by deformity (n = 14), degenerative diseases (n = 13), tumors (n = 5), trauma (n = 3), and others (n = 2). The pooled sensitivity for studies consisting of patients with mixed diseases was 52% (95% CI 26.5-76.5, Heterogeneity: I2 = 78%, τ2 = 3.9806, *P* < .01), for deformity it was 94% (95% CI 77.6-98.6, Heterogeneity: I2 = 3%, τ2 = 5.2433, *P* = .42), for degenerative it was 49.9% (95% CI 11.5-88.4 Heterogeneity: I2 = 0%, τ2 = 10.9146, *P* = .99), for tumor it was 33.2% (95% CI 9.2-70.9, Heterogeneity: I2 = 67%, τ2 = 2.7226, *P* = .01), for trauma it was 69.4% (95% CI 42.8-87.3, Heterogeneity: I2 = 11%, τ2 = .4518, *P* = .35), and for other diseases group, it was 35.3% (95% CI 16.8-59.6, Heterogeneity: I2 = 0%, τ2 = 0, *P* = .52) (Supplemental Figure 2(a)).

The pooled specificity for studies consisting of patients with mixed diseases was 97.6% (95% CI 94.2-99, Heterogeneity: I2 = 93%, τ2 = 2.8975, *P* < .01), for deformity it was 96% (95% CI 89.7-98.5, Heterogeneity: I2 = 95%, τ2 = 4.698, *P* < .01), for degenerative it was 99.1% (95% CI 97.6-99.7, Heterogeneity: I2 = 91%, τ2 = 2.1, *P* < .01), for tumor it was 93.5% (95% CI 83.4-97.6, Heterogeneity: I2 = 81%, τ2 = 1.16, *P* < .01), for trauma it was 83.4% (95% CI 42.8-87.3, Heterogeneity: I2 = 11%, τ2 = .4518, *P* = .35), and for other diseases group, it was 35.3% (95% CI 16.8-59.6, Heterogeneity: I2 = 0%, τ2 = .68, *P* = 1.00) (Supplemental Figure 2(b)).

The AUC for mixed pathology group, deformity, degenerative, tumor and trauma were .911, .908, .948, .791 and .744, respectively (Supplemental Figure 2(c)).

3. Regions

In terms of region of surgery, most studies presenting data for SSEP consisted of patients undergoing surgery for any region (n = 20), followed by surgery in the cervical spine (n = 11), lumbosacral (n = 9), thoracolumbar and cervicothoracic (n = 6 each) segments. The pooled sensitivity for studies consisting of patients with all regions was 77.2% (95% CI 60.4-88.3, Heterogeneity: I2 = 49%, τ2 = 3.27, *P* < .01), for cervical spine surgery it was 46.6% (95% CI 24.3-70.4, Heterogeneity: I2 = 59%, τ2 = 1.91, *P* < .01), for thoracolumbar spine surgery it was 99.1% (95% CI 29.1-100. Heterogeneity: I2 = 0%, τ2 = 16.9, *P* = .99), for lumbosacral it was 49.7% (95% CI 3.5-96.4, Heterogeneity: I2 = 0%, τ2 = 20.5, *P* = .68) and for cervicothoracic spine it was 24% (95% CI 17.8-31.5, Heterogeneity: I2 = 0%, τ2 = 0, *P* = .56) (Supplemental Figure 3(a)).

The pooled specificity for studies consisting of patients with surgery for any region was 95.5% (95% CI 90.5-97.9, Heterogeneity: I2 = 95%, τ2 = 4.57, *P* < .01), for cervical spine it was 98.5% (95% CI 95.2-99.6, Heterogeneity: I2 = 88%, τ2 = 3.3, *P* < .01), for thoracolumbar surgery it was 88.6% (95% CI 74.1-95.4, Heterogeneity: I2 = 91%, τ2 = 1.7, *P* < .01), for lumbosacral it was 99% (95% CI 3.5-96.4, Heterogeneity: I2 = 0%, τ2 = 20.5, *P* < .01), for cervicothoracic it was 96% (95% CI 94.4-97.1, Heterogeneity: I2 = 0%, τ2 = 0, *P* = .01) (Supplemental Figure 3(b)).

The AUC for cervical spine, cervicothoracic, thoracolumbar, lumbosacral and all regions were .928, .729, .879, .926 and .911, respectively (Supplemental Figure 3(c)).

## MEP

**Figure 5.**
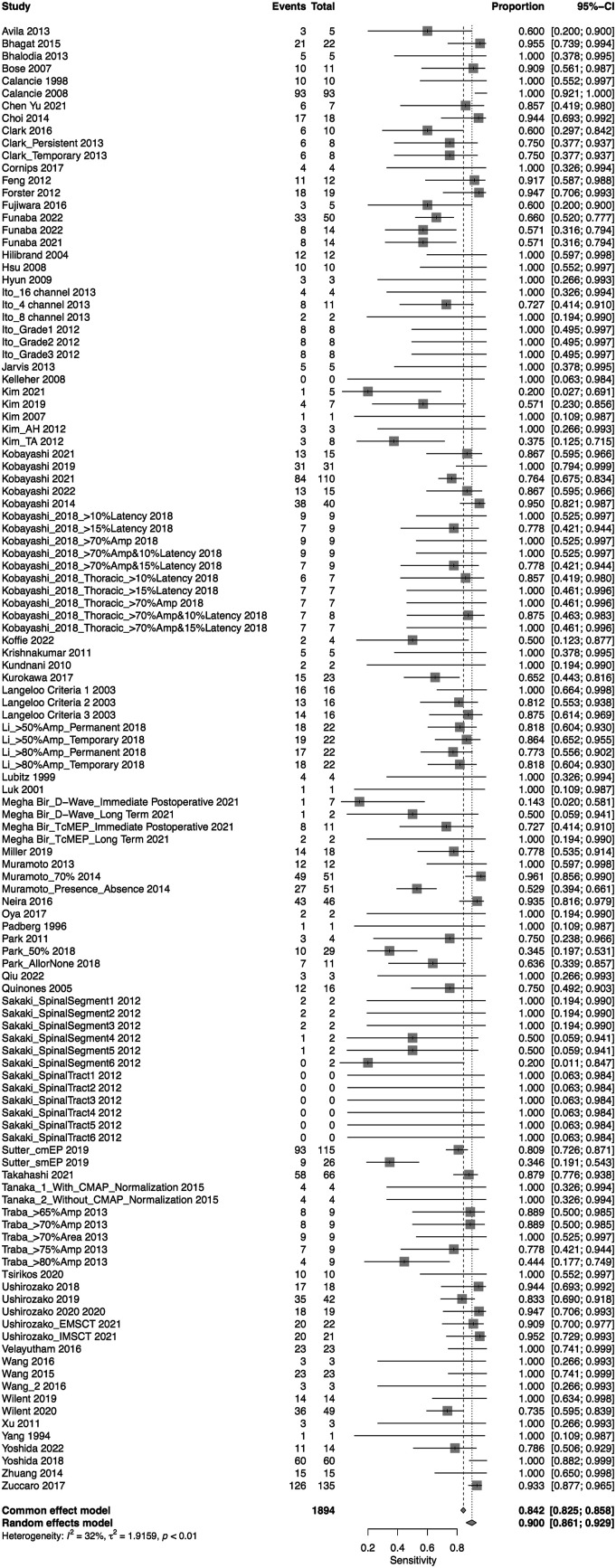
Forest plot for sensitivity of MEP.

**Figure 6.**
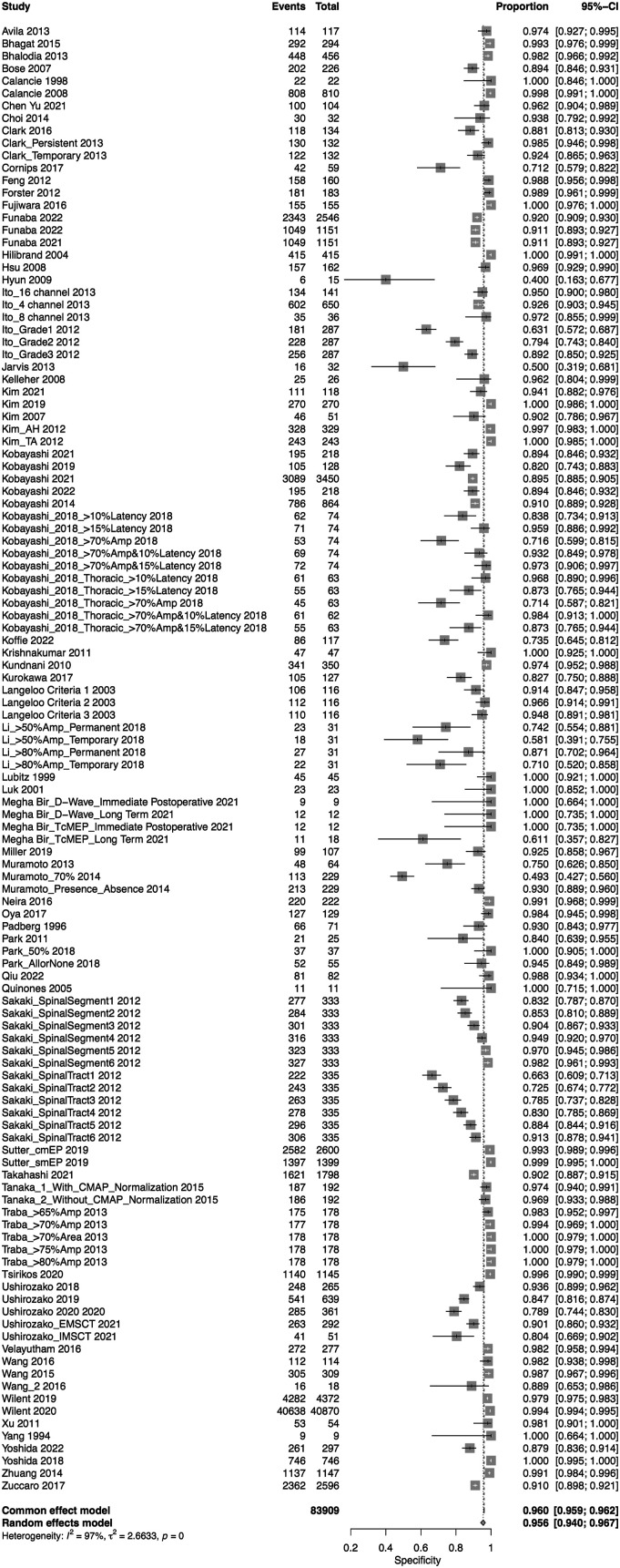
Forest plot for specificity of MEP.

**Figure 7.**
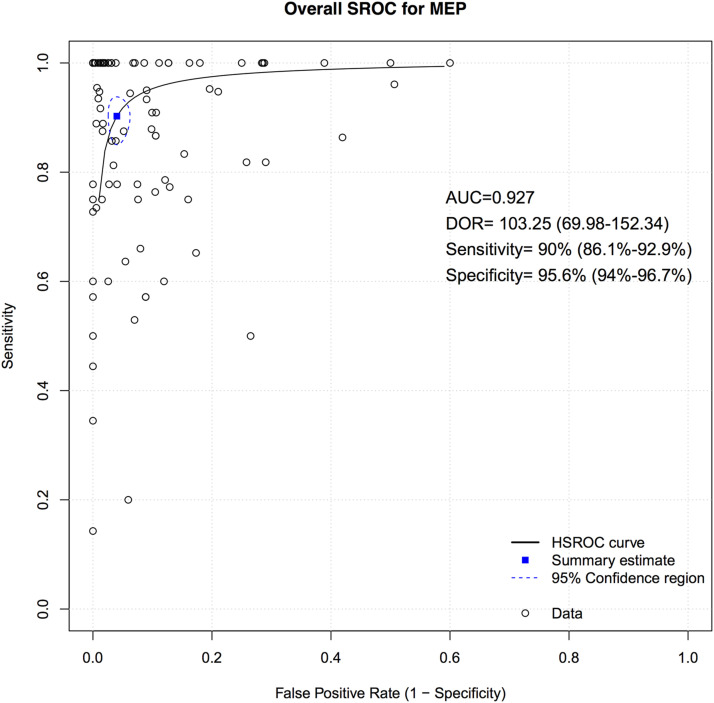
Overall sROC plot for MEP.

A total of 75 studies presented data for MEP on 79,545 patients. Overall, the sensitivity of MEP was 90% (95% CI 86.1-92.9, Heterogeneity: I2 = 32%, τ2 = 1.91, *P* < .01) (Figure 5), while the specificity was 95.6% (95% CI 94-96.7, Heterogeneity: I2 = 97%, τ2 = 2.7, *P* < .01) (Figure 6). Overall, the AUC value was .927, while the DOR was 103.25 (95% CI 69.98—152.34) (Figure 7).

We also performed subgroup analysis for various thresholds for IONM alerts u, different reported disease groups and different regions.

Subgroup Analyses:

1. Thresholds:

The most commonly reported change in amplitude threshold for alert was 50% (n = 24 studies), followed by 70% (n = 20 studies), 80% (n = 16 studies), “all or none” (n = 5 studies), 65% (n = 5 studies), 75% (n = 3 studies), change in latency of >10% and >15% (n = 2 each), 10%, 20%, 30%, 40%, 50%–65%, 50%–80%, 60% (n = 1 each), and significant change. Nine studies either did not specify the criteria for alarm or reported a method other than amplitude change or latency change. The pooled sensitivities for 50% threshold, 70% threshold, 80% threshold, “all or none” alert, 65% threshold and 75% threshold were 85.4% (95% CI 75-92, Heterogeneity: I2 = 50%, τ2 = 1.47, *P* < .01), 91.3% (95% CI 85.4-95, Heterogeneity: I2 = 38%, τ2 = 1.16, *P* = .03), 92.3% (95% CI 79.2-97.4, Heterogeneity: I2 = 0%, τ2 = 2.59, *P* = .65), 56.8% (95% CI 45.3-67.5, Heterogeneity: I2 = 0%, τ2 = 0, *P* = .92), 94% (95% CI 85.1-97.7, Heterogeneity: I2 = 0%, τ2 = 0, *P* = .99) and 89.7% (95% CI 75.7-96.1, Heterogeneity: I2 = 0%, τ2 = 0, *P* = .43), respectively (Supplemental Figure 4(a)). The pooled specificity for 50% threshold, 70% threshold, 80% threshold, “all or none” alert, 65% threshold and 75% threshold were 94.2% (95% CI 88.2-97.2, Heterogeneity: I2 = 99%, τ2 = 3.42, *P* < .01), 90.4% (95% CI 85.4-93.9, Heterogeneity: I2 = 94%, τ2 = 1.39, *P* < .01), 96.7% (95% CI 94.2-98.1, Heterogeneity: I2 = 88%, τ2 = 1.47, *P* < .01), 96.1% (95% CI 92.7-98, Heterogeneity: I2 = 69%, τ2 = .39, *P* < .01), 98.2% (95% CI 97.3-98.8, Heterogeneity: I2 = 0%, τ2 = 0, *P* = .72) and 99% (95% CI 94.1-99.8, Heterogeneity: I2 = 25%, τ2 = 1.39, *P* = .43), respectively (Supplemental Figure 4(b)).

2. Disease Group:

In terms of disease group, most studies presenting data for MEP consisted of patients with mixed diseases (n = 27), followed by deformity (n = 19), degenerative diseases (n = 17), tumors (n = 12), and others (n = 2). The pooled sensitivities for studies consisting of patients with mixed diseases was 93.9% (95% CI 87.1-97.2, Heterogeneity: I2 = 20%, τ2 = 3.63, *P* = .12), for deformity it was 92.4% (95% CI 89.3-94.7, Heterogeneity: I2 = 0%, τ2 = 0, *P* = .95), for degenerative it was 80% (95% CI 66.3-89, Heterogeneity: I2 = 0%, τ2 = 1.0, *P* = .99), and for tumor it was 85.4% (95% CI 72.9-92.7, Heterogeneity: I2 = 73%, τ2 = 1.58, *P* < .01) (Supplemental Figure 5(a)).

The pooled specificities for studies consisting of patients with mixed diseases was 97% (95% CI 94.6-98.3, Heterogeneity: I2 = 93%, τ2 = 2.8975, *P* < .01), for deformity it was 96.1% (95% CI 93-97.9, Heterogeneity: I2 = 93%, τ2 = 3.51, *P* < .01), for degenerative it was 94.9% (95% CI 91.5-97, Heterogeneity: I2 = 99%, τ2 = 2.1, *P* < .01) and for tumor it was 85.4% (95% CI 72.9-92.7, Heterogeneity: I2 = 95%, τ2 = 1.94, *P* < .01) (Supplemental Figure 5(b)).

The AUC for mixed pathology group, deformity, degenerative and tumor were .937, .934, .948, .722 and .915, respectively (Supplemental Figure 5(c)).

3. Regions

In terms of region of surgery, most studies presenting data for MEP consisted of patients undergoing surgery for any region (n = 20), followed by surgery in the cervical spine (n = 11), lumbosacral (n = 9), thoracolumbar and cervicothoracic (n = 6 each) segments. The pooled sensitivity for studies consisting of patients with all regions was found to be 91.6% (95% CI 87.2-94.6, Heterogeneity: I2 = 35%, τ2 = 1.7, *P* < .01), for cervical spine surgery it was 80.2% (95% CI 64.7-89.9, Heterogeneity: I2 = 0%, τ2 = 1.26, *P* = 1.00), for thoracic spine surgery it was 100% (95% CI 0-100. Heterogeneity: I2 = 0%, τ2 = 0, *P* = .99), for lumbosacral it was 74.7% (95% CI 65.1-82.5, Heterogeneity: I2 = 0%, τ2 = 0, *P* = .78) and for cervicothoracic spine it was 90.6% (95% CI 77.6-96.4, Heterogeneity: I2 = 45%, τ2 = 2.37, *P* = .02) (Supplemental Figure 6(a)).

The pooled specificity for studies consisting of patients with surgery for any region was 97% (95% CI 95.1-98.2, Heterogeneity: I2 = 93%, τ2 = 3.44, *P* < .01), for cervical spine, it was 93.3% (95% CI 89.8-95.6, Heterogeneity: I2 = 96%, τ2 = 1.38, *P* < .01), for thoracic spine, it was 77.6% (95% CI 71.2-82.8, Heterogeneity: I2 = 35%, τ2 = .007, *P* < .01), for lumbosacral it was 98% (95% CI 91.1-99.6, Heterogeneity: I2 = 99%, τ2 = 2.7, *P* < .01), for cervicothoracic it was 92.8% (95% CI 87.1-96.1, Heterogeneity: I2 = 92%, τ2 = 1.47, *P* < .01) (Supplemental Figure 6(b)).

The AUC for cervical spine, cervicothoracic, thoracic, lumbosacral and all regions were .742, .919, .895, .804 and .934, respectively (Supplemental Figure 6(c)).

## EMG

**Figure 8.**
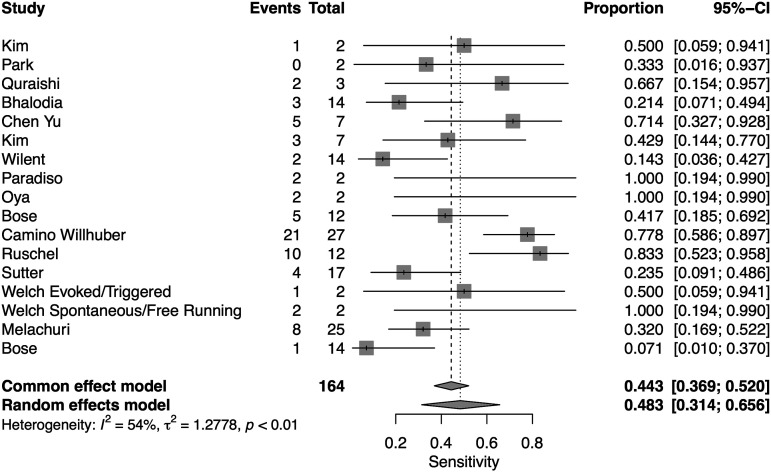
Forest plot for sensitivity of EMG.

**Figure 9.**
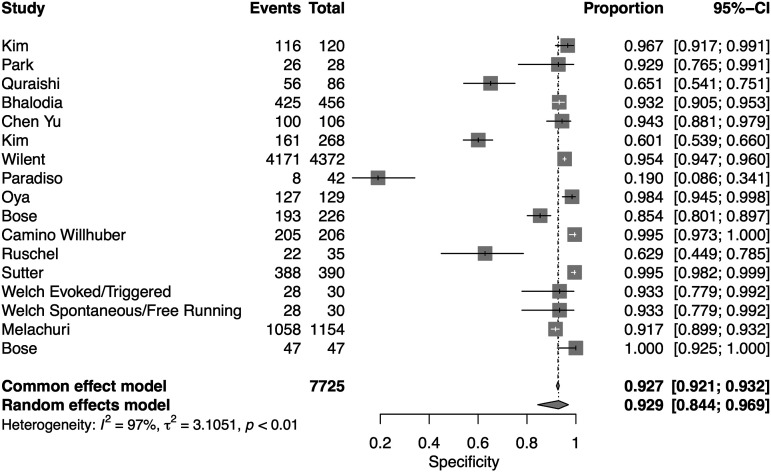
Forest plot for specificity of EMG.

**Figure 10.**
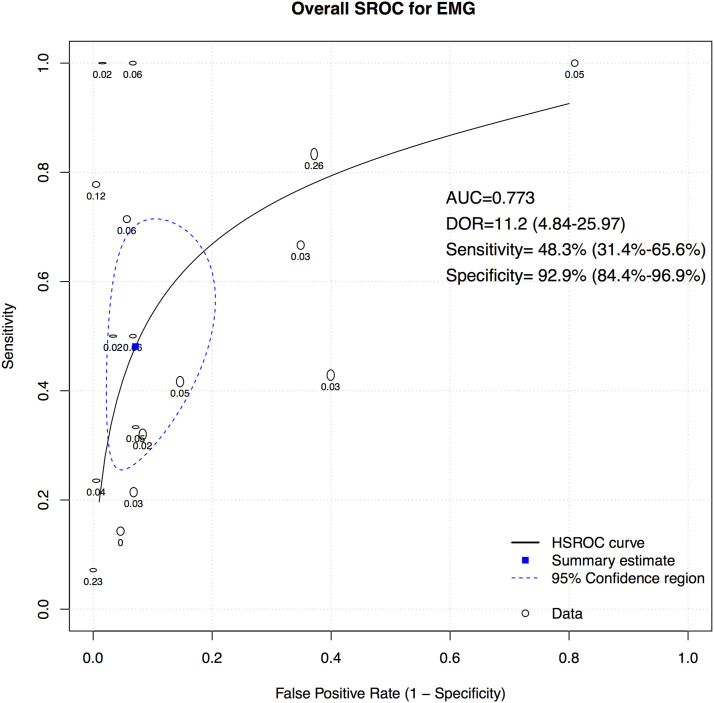
Overall sROC plot for EMG.

A total of 16 studies presented data for EMG on 7004 patients. Overall, the pooled sensitivity for EMG was 48.3% (95% CI 31.4-65.6, Heterogeneity I2 = 54, τ2 = 1.27, *P* < .01) (Figure 8), while the pooled specificity was 92.9% (95% CI 84.4-96.9, Heterogeneity I2 = 97, τ2 = 3.1, *P* < .01) (Figure 9). The AUC was .773 and the DOR was 11.2 (95% CI 4.84-25.97) (Figure 10).

We also performed subgroup analysis for type of EMG, different reported disease groups, and different regions.

1. Type of EMG

Eleven studies reported on free-running or spontaneous EMG, 2 studies reported on evoked/triggered/stimulated EMG, and 4 studies reported on combined free-running and triggered EMG. The pooled sensitivity for free-running EMG was 54.6% (95% CI 33.8-74, Heterogeneity: I2 = 55%, τ2 = 1.13, *P* = .02), for evoked/triggered/stimulated EMG it was 33.3% (95% CI 31.4-65.6, Heterogeneity: I2 = 0%, τ2 = 0, *P* = .61), and for combined free-running and triggered EMG it was 40.7% (95% CI 9.4-82, Heterogeneity: I2 = 60%, τ2 = 2.5, *P* = .06) (Supplemental Figure 7(a)).

The pooled specificity for free-running EMG was 91.9% (95% CI 82.4-96.5, Heterogeneity: I2 = 98%, τ2 = 1.98, *P* < .01), for evoked/triggered/stimulated EMG it was 91.7% (95% CI 90-93.2, Heterogeneity: I2 = 0%, τ2 = 0, *P* = .75), and for combined free-running and triggered EMG it was 96.4% (95% CI 48.2-99, Heterogeneity: I2 = 97%, τ2 = 10.01, *P* < .01) (Supplemental Figure 7(b)).

The AUC for free-running was .773, for triggered EMG it was .873, and for combined free-running and triggered EMG it was .792 (Supplemental Figure 7(c)).

2. Disease Group

Three studies reported data on EMG for deformity surgery, 5 studies reported data for degenerative diseases and 5 studies reported data for surgery of mixed pathologies. One study each reported data for EMG for detethering, tumor and unspecified. The pooled sensitivity for EMG for deformity studies was found to be 30.2% (95% CI 14.7-52.1, Heterogeneity: I2 = 0%, τ2 = 0, *P* = .38), for degenerative studies it was found to be 76.2% (95% CI 61.1-86.7, Heterogeneity: I2 = 0%, τ2 = 0, *P* = .93), while for mixed pathology studies it was found to be 29.2% (95% CI 19.9-40.6, Heterogeneity: I2 = 0%, τ2 = 0, *P* = .50) (Supplemental Figure 8(a)).

The pooled specificity for EMG for deformity studies was found to be 94.7% (95% CI 64.1-99.4, Heterogeneity: I2 = 95%, τ2 = 3.73, *P* < .01), for degenerative studies it was found to be 97.3% (95% CI 95.6-98.3, Heterogeneity: I2 = 40%, τ2 = .36, *P* = .75), while for mixed pathology studies it was found to be 88.6% (95% CI 77.6-94.6, Heterogeneity: I2 = 99%, τ2 = .83, *P* < .01) (Supplemental Figure 8(b)).

The AUC for deformity was found to be .659, for degenerative it was found to be .888, while for various diseases it was found to be .552 (Supplemental Figure 8(c)).

3. Regions

Eight studies provided data for EMG for lumbosacral surgery, 5 studies provided data for cervicothoracic surgery, 2 studies provided data for EMG for surgery for any region, and one study provided data for EMG for cervical spine surgery.

The pooled sensitivity for lumbosacral surgery was 49.6% (95% CI 26.6-72.8, Heterogeneity: I2 = 63%, τ2 = 1.41, *P* < .01), for cervicothoracic surgery it was 36.1% (95% CI 16-62.7, Heterogeneity: I2 = 0%, τ2 = .3, *P* < .01) and for surgery for any region it was 80% (95% CI 53-93.4, Heterogeneity: I2 = 0%, τ2 = 0, *P* < .01) (Supplemental Figure 9(a)).

The pooled specificity for lumbosacral surgery was 94.7% (95% CI 78.6-98.8, Heterogeneity: I2 = 97%, τ2 = 5.1, *P* < .01), for cervicothoracic surgery it was 94.9% (95% CI 89.9-97.5, Heterogeneity: I2 = 91%, τ2 = .50, *P* < .01) and for surgery for any region it was 64.5% (95% CI 55.6-72.5, Heterogeneity: I2 = 0%, τ2 = 0, *P* < .01) (Supplemental Figure 9(b)).

The AUC values for lumbosacral surgery, cervicothoracic surgery and for surgery for any region were .738, .492 and .655, respectively (Supplemental Figure 9(c)).

### Multimodal

**Figure 11.**
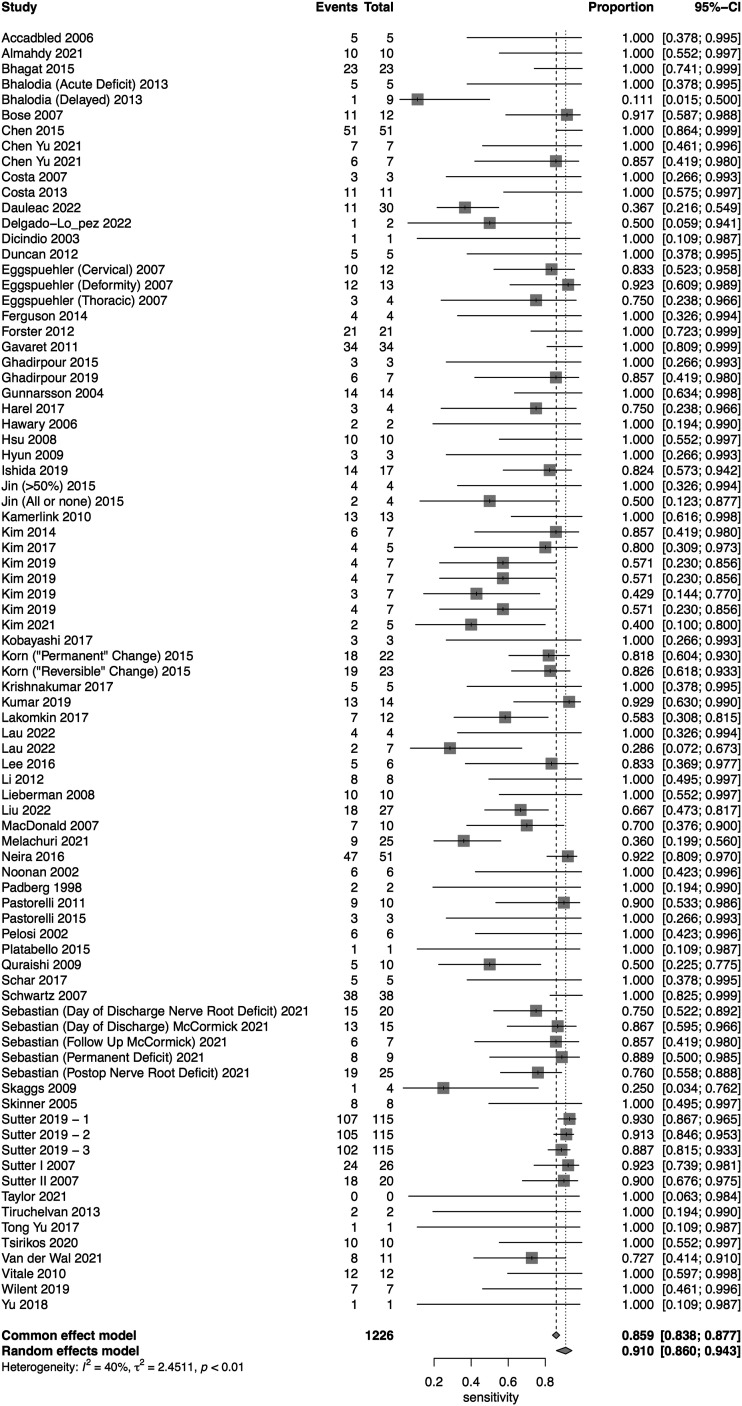
Forest plot for sensitivity of Multimodal Neuromonitoring.

**Figure 12.**
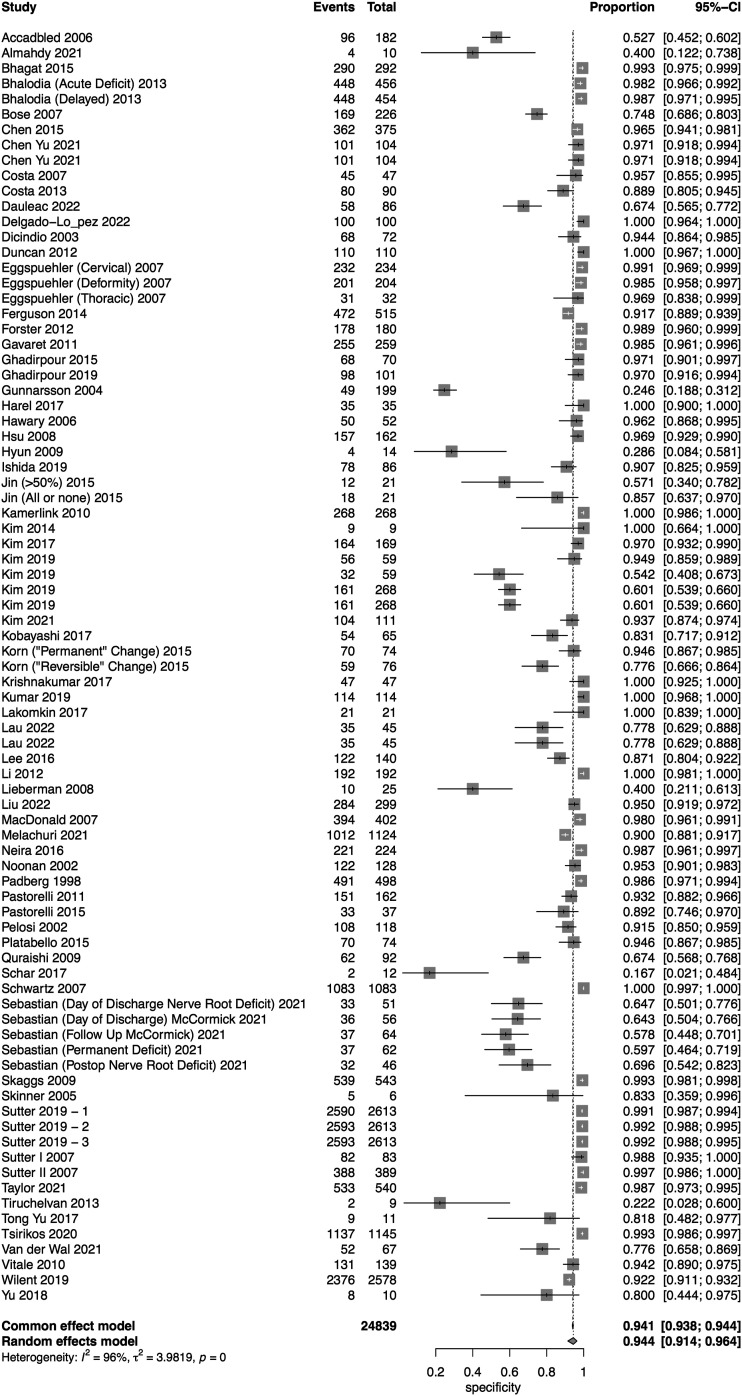
Forest plot for specificity of Multimodal Neuromonitoring.

**Figure 13.**
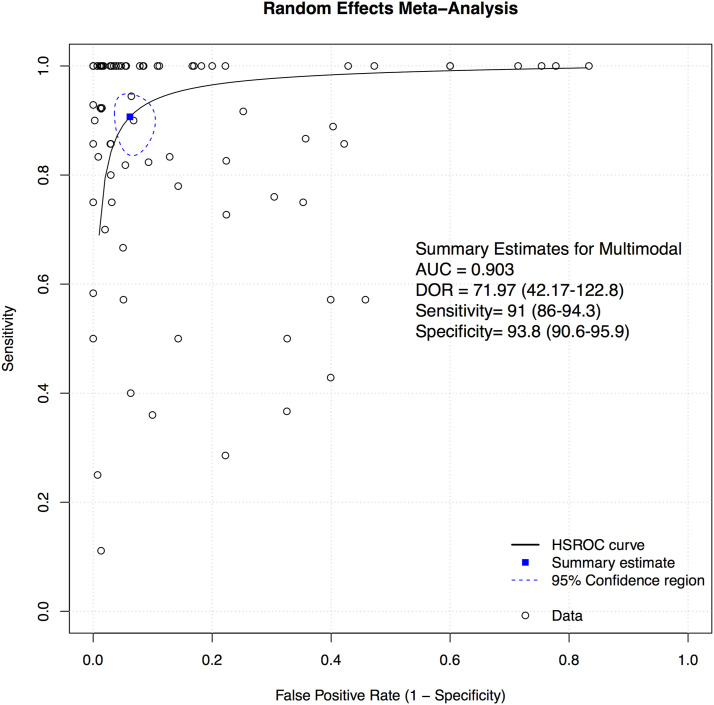
Overall sROC plot for Multimodal Neuromonitoring.

A total of 69 studies presented data for multimodal neuromonitoring on 58,325 patients. Overall, the sensitivity of multimodal neuromonitoring was 91% (95% CI 86%–94.3%) (Figure 11), while the pooled specificity was 93.8% (95% CI 90.6%–95.9%) (Figure 12). The AUC value was .903 while the DOR was 71.97 (95% CI 42.17-122.8) (Figure 13).

We also performed subgroup analyses based on type of multimodal neuromonitoring, disease subset, and region of surgery.

1. Type of Multimodal Neuromonitoring.

A total of 33 studies presented data for combined SSEP and MEP, 27 studies for combined SSEP, MEP and EMG, 6 studies for combined SSEP, MEP and D-wave, 4 studies for combined MEP and EMG, 3 studies for combined MEP and D-wave, 2 studies for combined SSEP and EMG, and one study for combined SSEP and D-wave. The pooled sensitivity for SSEP and MEP was 93.5% (95% CI 83.1-97.7, Heterogeneity: 0%, τ2 = 3.8, *P* = .65), for combined SSEP, MEP and EMG it was 87.7% (95% CI 80-92.7, Heterogeneity: 18%, τ2 = 1.22, *P* = .19), for combined SSEP, MEP and D-wave it was 90.2% (95% CI 63.5-98, Heterogeneity: 63%, τ2 = 2.68, *P* = .01), for combined MEP and EMG it was 92.3% (95% CI 53.8-99.2, Heterogeneity: 0%, τ2 = 2.06, *P* = .65), for combined MEP and D-wave it was 90.4% (95% CI 86-94.3, Heterogeneity: 0%, τ2 = 0, *P* = .81), and for SSEP and EMG it was 90.7% (95% CI 3.6-100, Heterogeneity: 0%, τ2 = 9.91, *P* = .99) (Supplemental Figure 10(a)).

The pooled specificity for SSEP and MEP was 95.3% (95% CI 90.7-97.7, Heterogeneity: 95%, τ2 = 4.11, *P* < .01), for combined SSEP, MEP and EMG it was 94.3% (95% CI 88.7-97.2, Heterogeneity: 96%, τ2 = 4.27, *P* < .01), for combined SSEP, MEP and D-wave it was 93.1% (95% CI 83.6-97.3, Heterogeneity: 90%, τ2 = 1.48, *P* < .01), for combined MEP and EMG it was 77.2% (95% CI 40-94.5, Heterogeneity: 91%, τ2 = 2.35, *P* = .65), for combined MEP and D-wave it was 99.2% (95% CI 98.9-99.4, Heterogeneity: 77%, τ2 = 0, *P* = .01), and for SSEP and EMG it was 63.3% (95% CI 14.6-94.5, Heterogeneity: 100%, τ2 = 2.75, *P* < .01) (Supplemental Figure 10(b)).

The AUC values for combined SSEP and MEP was found to be .908; .881 for combined SSEP, MEP and EMG; .938 for SSEP, MEP and D-wave, and .848 for MEP and EMG (Supplemental Figure 10(c)).

2. Disease Group

A total of 23 studies presented data for multimodal neuromonitoring for deformity surgery, 17 studies for spinal tumors, 16 studies for various disease groups, 12 studies for degenerative, and 1 study for trauma.

The pooled sensitivity for multimodal neuromonitoring for deformity was 98.8% (95% CI 88.9-99.9, Heterogeneity: I2 = 0%, τ2 = 7.92, *P* = .51), for degenerative disease it was 74.7% (95% CI 62.3-84, Heterogeneity: I2 = 0%, τ2 = .35, *P* = .84), for mixed pathology it was 95.6% (95% CI 84.1-98.9, Heterogeneity: I2 = 68%, τ2 = 4.94, *P* < .01), and for tumor it was 83.9% (95% CI 75.6-89.8, Heterogeneity: I2 = 31%, τ2 = .66, *P* = .08) (Supplemental Figure 11(a)).

The pooled specificity for multimodal neuromonitoring for deformity was 96% (95% CI 91.4-98.2, Heterogeneity: I2 = 95%, τ2 = 3.78, *P* < .01) for degenerative disease it was 95.2% (95% CI 86.7-98.3, Heterogeneity: I2 = 96%, τ2 = 4.50, *P* < .01), for mixed pathology it was f 95.8% (95% CI 90.8-98.1, Heterogeneity: I2 = 98%, τ2 = 3.27, *P* < .01), and for tumor it was 88.6% (95% CI 77.1-94.7, Heterogeneity: 84%, τ2 = 3.49, *P* < .01) (Supplemental Figure 11(b)).

The AUC for deformity was .946, for degenerativ disease it was .787, for mixed pathology it was .958, and for tumor it was .844 (Supplemental Figure 11(c)).

3. Regions

Six studies provided data for multimodal neuromonitoring for lumbosacral surgery, 3 studies for cervicothoracic surgery, 37 studies for surgery for any region, 10 studies for cervical spine surgery, 11 studies for thoracolumbar surgery, and 2 studies for thoracic spine surgery.

The pooled sensitivity for lumbosacral surgery was 76.2% (95% CI 54.4-89.6, Heterogeneity: I2 = 32%, τ2 = 1.35, *P* = .15), for cervicothoracic surgery it was 98.4% (95% CI 6.4-100, Heterogeneity: I2 = 0%, τ2 = 13.02, *P* = .99), for surgery for any region it was 92.5% (95% CI 86.9-95.8, Heterogeneity: I2 = 41%, τ2 = 1.92, *P* < .01), for cervical spine surgery it was 81.2% (95% CI 54-94.1, Heterogeneity: 27%, τ2 = 2.4, *P* = .19), for thoracolumbar surgery it was 96.9% (95% CI 82.6-99.5, Heterogeneity: 0%, τ2 = 1.49, *P* = .99), and for thoracic surgery it was 67.7% (95% CI 49.7-81.7, Heterogeneity: 0%, τ2 = 0, *P* = .74) (Supplemental Figure 12(a)).

The pooled specificity for lumbosacral surgery was 91.5% (95% CI 69.3-98.1, Heterogeneity: I2 = 97%, τ2 = 5.78, *P* < .01), for cervicothoracic surgery it was 59.5% (95% CI 33.7-81, Heterogeneity: I2 = 77%, τ2 = .84, *P* < .01), for surgery for any region it was 95.2% (95% CI 91.4-97.4, Heterogeneity: I2 = 97%, τ2 = 4.03, *P* < .01), for cervical spine surgery it was 97.6% (95% CI 94.3-99.1, Heterogeneity: 94%, τ2 = 2, *P* < .01), for thoracolumbar surgery it was 92.1% (95% CI 81.2-97, Heterogeneity: 96%, τ2 = 2.59, *P* < .01) and for thoracic surgery it was 95.2% (95% CI 92.3-97, Heterogeneity: 0%, τ2 = 0, *P* = .64) (Supplemental Figure 12(b)).

The AUC values for cervical surgery, cervicothoracic surgery, thoracic surgery, thoracolumbar, lumbosacral and surgery for any region were found to be .0.928, .718, .845, .89, .791 and .916, respectively (Supplemental Figure 12(c)).

### Publication Bias Assessment Using Funnel Plot

**Figure 14.**
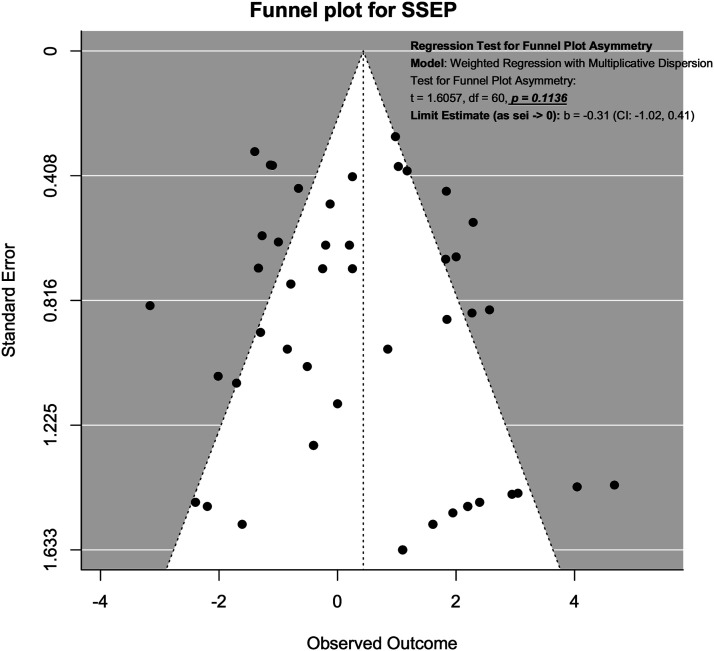
Funnel plot for Assessment of Publication Bias for SSEP.

**Figure 15.**
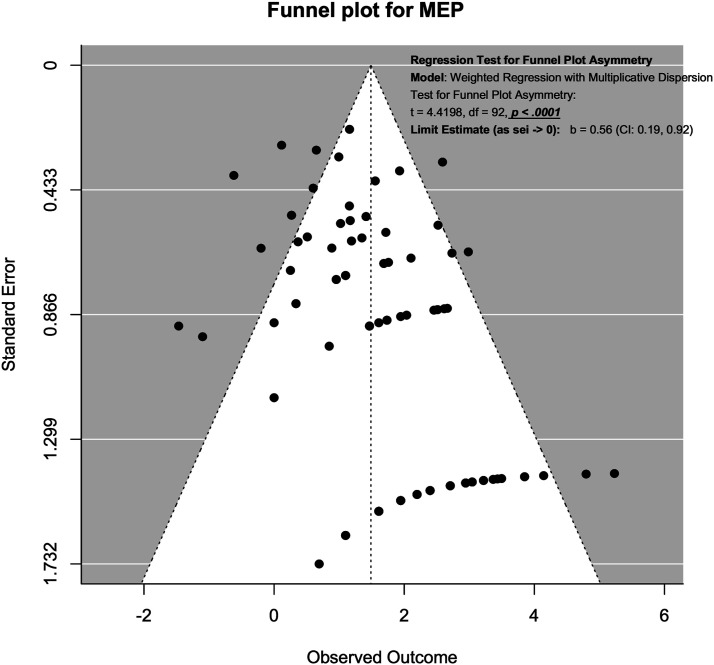
Funnel plot for Assessment of Publication Bias for MEP.

**Figure 16.**
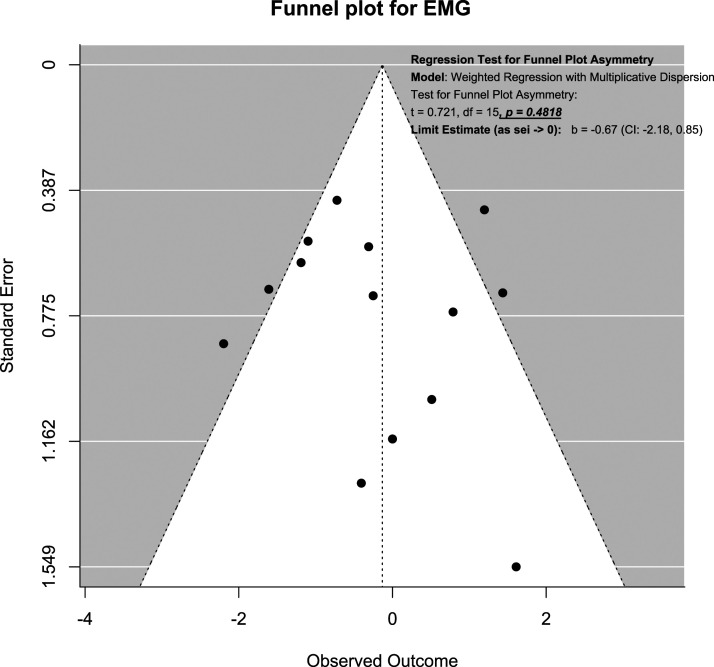
Funnel plot for Assessment of Publication Bias for EMG.

**Figure 17.**
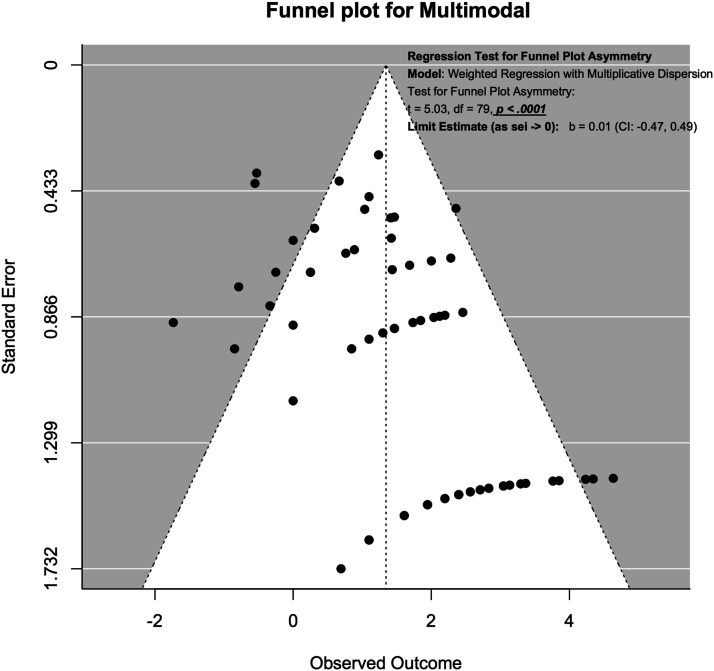
Funnel plot for Assessment of Publication Bias for Multimodal Neuromonitoring.

Publication bias was assessed using funnel plots and modified Hong’s test proposed by Noma.^38^ For SSEP neuromonitoring, (Figure 14), we observed slight asymmetry but the weighted regression with multiplicative dispersion test for asymmetry was not statistically significant (t = 1.61, df = 60, *P* = .11). For MEP neuromonitoring, (Figure 15), we observed asymmetry and the weighted regression with multiplicative dispersion test for asymmetry was statistically significant (t = 4.42, df = 92, *P* < .001). For multimodal neuromonitoring, (Figure 16), we observed asymmetry but the weighted regression with multiplicative dispersion test for asymmetry was not statistically significant (t = .72, df = 15, *P* = .48). For multimodal neuromonitoring, (Figure 17), we observed asymmetry and the weighted regression with multiplicative dispersion test for asymmetry was statistically significant (t = 5.03, df = 79, *P* < .001).

### Risk of Bias Assessment Using QUADAS Tool

#### Risk of bias was assessed using the QUADAS tool

**Figure 18.**
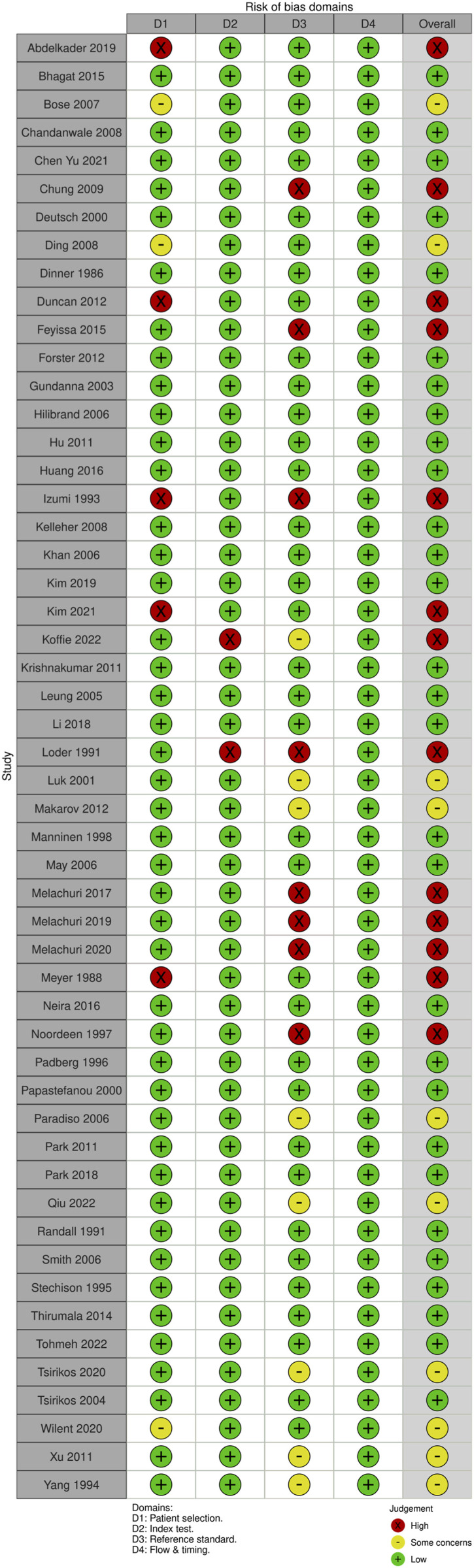
QUADAS-2 risk of bias traffic light plot for SSEP.

**Figure 19.**
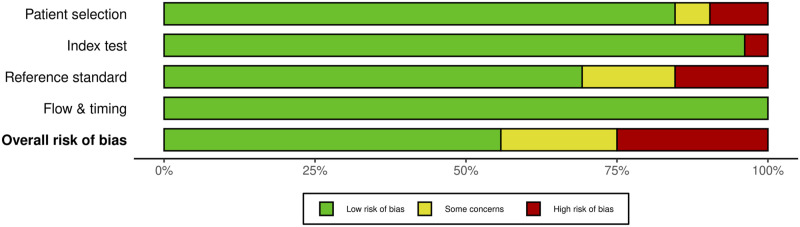
QUADAS-2 risk of bias summary plot for SSEP.

For SSEP monitoring, of the 52 studies, 10 studies (19.2%) had some concerns, 25% (n = 13) were high risk and the remaining 29 studies (55.8%) were low risk. For most of the studies that were graded down, risk of bias was identified in the “reference standard” domain; the reason was lack of specification details of the postoperative examination, or the use of a non-standard exam (Figures 18 and 19).

**Figure 20.**
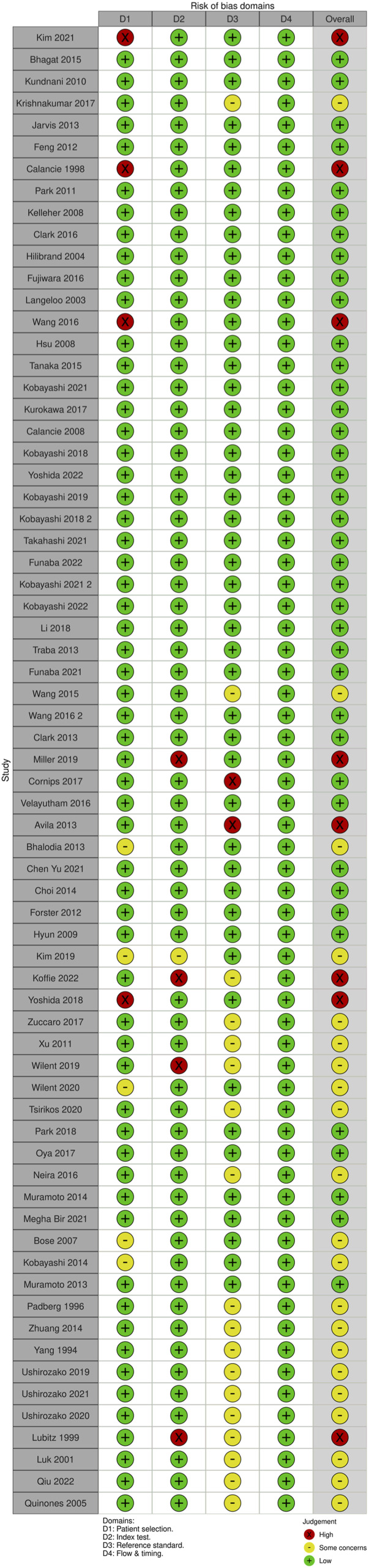
QUADAS-2 risk of bias traffic light plot for MEP.

**Figure 21.**
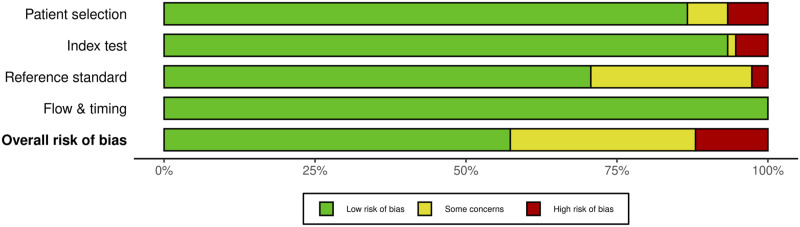
QUADAS-2 risk of bias summary plot for MEP.

For MEP monitoring, of the 75 studies, 21 studies (28%) had some concerns, 10.7% (n = 8) were high risk and the remaining 46 studies (61.3%) were low risk. For most of the studies that were graded down, risk of bias was identified in the “reference standard” domain (Figures 20 and 21).

**Figure 22.**
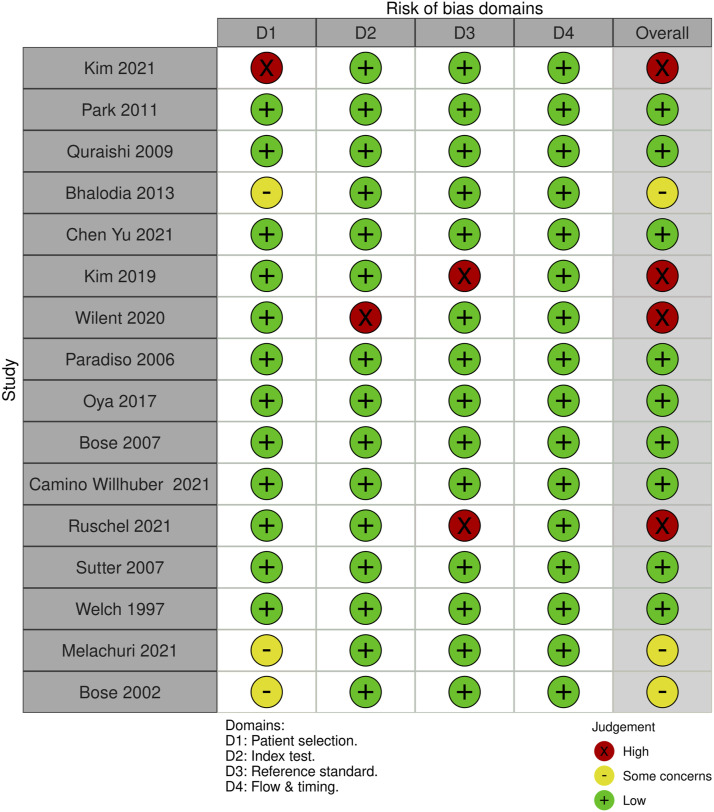
QUADAS-2 risk of bias traffic light plot for EMG.

**Figure 23.**
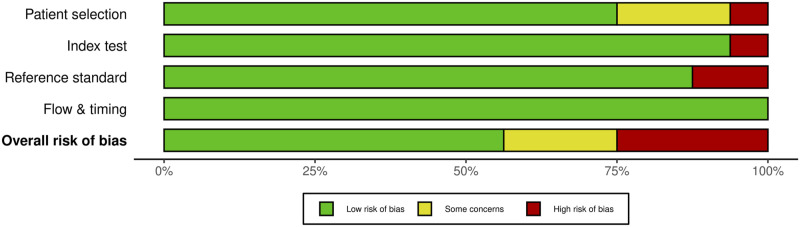
QUADAS-2 risk of bias summary plot for EMG.

For EMG monitoring, of the 16 studies, 3 studies (18.75%) had some concerns, 25% (n = 4) were high risk and the remaining 9 studies (56.25%) were low risk. For most of the studies that were graded down, risk of bias was identified in the “index test” test domain; the reason was lack of specification details of the changes in EMG monitoring that were considered an alert (Figures 22 and 23).

**Figure 24.**
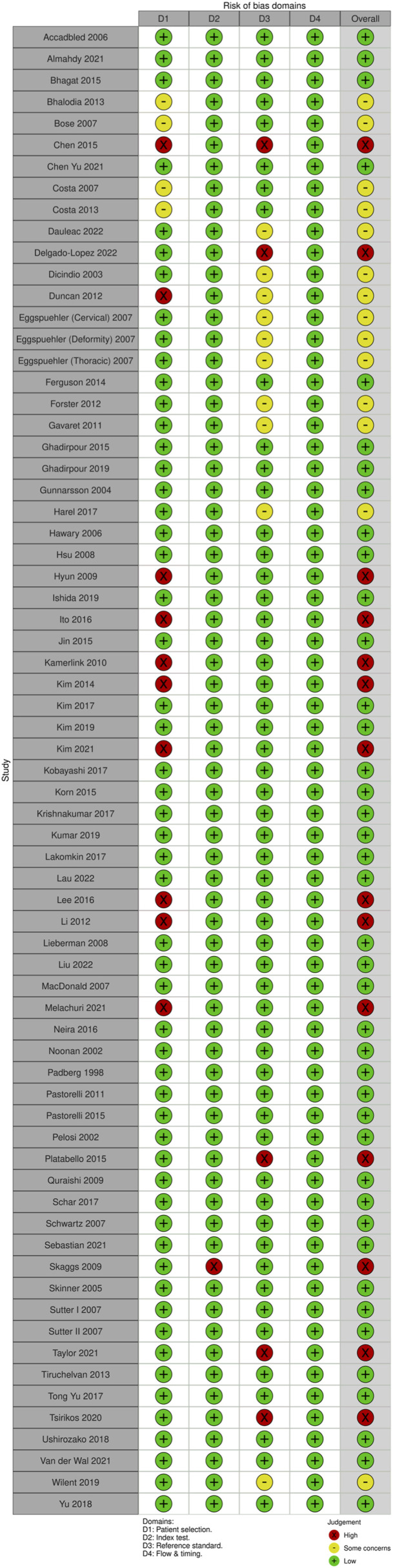
QUADAS-2 risk of bias traffic light plot for multimodal neuromonitoring.

**Figure 25.**
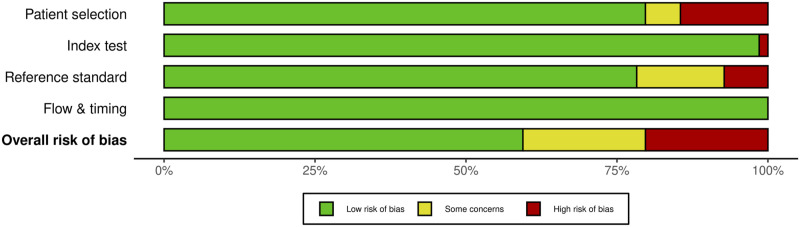
QUADAS-2 risk of bias summary plot for multimodal neuromonitoring.

For multimodal neuromonitoring, of the 69 studies, 14 studies (20.3%) had some concerns, 14 studies (20.3%) were high risk and the remaining 41 studies (59.4%) were low risk. For most of the studies that were graded down, ris of bias was identified in the “index” domain; the reason was lack of specification/details of the criteria that constituted an alert. (Figures 24 and 25).

### GRADE Assessment of Strength of Evidence

We applied the GRADE assessment methodology described by Yang et al to evaluate the strength of evidence for each of the 4 groups:. SSEP, MEP, EMG and multimodal neuromonitoring. These are summarized in Tables 5a, 5b, 5c, and 5d, respectively. For all 4 groups, the final quality of the evidence was “Low”. Evidence was downgraded for “Inconsistency,” “Imprecision” and “Publication Bias.” Studies were downgraded for inconsistency because of differences in included population/pathology type (deformity vs tumor vs degenerative vs mixed population) and because of the use of different “thresholds”. Studies were downgrade for “Imprecision” due to low number of events (TP + FN) resulting in large confidence intervals, particularly for sensitivity. Finally, studies were downgraded for “Publication Bias” due to both observed and statistically significant asymmetry.

**Table 5.** GRADE strength of evidence for different neuromonitoring approaches.

Table 5a: GRADE strength of evidence for SSEP			
Quality criteria	**Rating** (circle one for each criterion)	**Footnotes** (explain reasons for up- or downgrading)	**Quality of the evidence** (circle one per outcome)
Risk of bias	***No*** serious (-1) very serious (-2)	All studies observational with majority of them being low risk of bias	⊕⊕⊕⊕ High ⊕⊕⊕□ Moderate ⊕⊕□□ ***Low*** ⊕□□□ Very low
Inconsistency	No ***serious (-1)*** very serious (-2)	Inconsistency in results/estimates/effects very likely due to differences in included population/pathology type (deformity vs tumor vs degenerative vs mixed population)	
Indirectness	***No*** serious (-1) very serious (-2)	All studies employed a postoperative neurologic examination as a reference standard using which the utility/diagnostic efficacy of neuromonitoring was assessed	
Imprecision	No ***serious (-1)*** very serious (-2)	Due to low number of events (true positive + false negatives), most studies have very large confidence intervals, particularly for sensitivity	
Publication bias	Unlikely ***likely (-1)*** very likely (-2)	Test for asymmetry not statistically significant	
Large effect	Large (+1) Very large (+2)	**No**	
Dose-response gradient	No Yes (+1)	**NA**	
Plausible confounding would change the effect	No Yes (+1)	**NA**	

Table 5a: GRADE strength of evidence for MEP			
Quality criteria	**Rating** (circle one for each criterion)	**Footnotes** (explain reasons for up- or downgrading)	**Quality of the evidence** (circle one per outcome)
Risk of bias	***No*** serious (-1) very serious (-2)	All studies observational with majority of them being low risk of bias	⊕⊕⊕⊕ High ⊕⊕⊕□ Moderate ⊕⊕□□ ***Low*** ⊕□□□ Very low
Inconsistency	No ***serious (-1)*** very serious (-2)	Inconsistency in results/estimates/effects very likely due to differences in included population/pathology type (deformity vs tumor vs degenerative vs mixed population)	
Indirectness	***No*** serious (-1) very serious (-2)	All studies employed a postoperative neurologic examination as a reference standard using which the utility/diagnostic efficacy of neuromonitoring was assessed	
Imprecision	No ***serious (-1)*** very serious (-2)	Due to low number of events (true positive + false negatives), most studies have very large confidence intervals, particularly for sensitivity	
Publication bias	Unlikely ***likely (-1)*** very likely (-2)	Test for asymmetry statistically significant	
Large effect	Large (+1) Very large (+2)	**No**	
Dose-response gradient	No Yes (+1)	**NA**	
Plausible confounding would change the effect	No Yes (+1)	**NA**	

Table 5c: GRADE strength of evidence for EMG			
Quality criteria	**Rating** (circle one for each criterion)	**Footnotes** (explain reasons for up- or downgrading)	**Quality of the evidence** (circle one per outcome)
Risk of bias	***No*** serious (-1) very serious (-2)	All studies observational with majority of them being low risk of bias	⊕⊕⊕⊕ High ⊕⊕⊕□ Moderate ⊕⊕□□ ***Low*** ⊕□□□ Very low
Inconsistency	No ***serious (-1)*** very serious (-2)	Inconsistency in results/estimates/effects very likely due to differences in included population/pathology type (deformity vs tumor vs degenerative vs mixed population)	
Indirectness	***No*** serious (-1) very serious (-2)	All studies employed a postoperative neurologic examination as a reference standard using which the utility/diagnostic efficacy of neuromonitoring was assessed	
Imprecision	No ***serious (-1)*** very serious (-2)	Due to low number of events (true positive + false negatives), most studies have very large confidence intervals, particularly for sensitivity	
Publication bias	Unlikely ***likely (-1)*** very likely (-2)	Test for asymmetry not statistically significant	
Large effect	Large (+1) Very large (+2)	**No**	
Dose-response gradient	No Yes (+1)	**NA**	
Plausible confounding would change the effect	No Yes (+1)	**NA**	

Table 5d: GRADE strength of evidence for multimodal			
Quality criteria	**Rating** (circle one for each criterion)	**Footnotes** (explain reasons for up- or downgrading)	**Quality of the evidence** (circle one per outcome)
Risk of bias	***No*** serious (-1) very serious (-2)	All studies observational with majority of them being low risk of bias	⊕⊕⊕⊕ High ⊕⊕⊕□ Moderate ⊕⊕□□ ***Low*** ⊕□□□ Very low
Inconsistency	No ***serious (-1)*** very serious (-2)	Inconsistency in results/estimates/effects very likely due to differences in included population/pathology type (deformity vs tumor vs degenerative vs mixed population)	
Indirectness	***No*** serious (-1) very serious (-2)	All studies employed a postoperative neurologic examination as a reference standard using which the utility/diagnostic efficacy of neuromonitoring was assessed	
Imprecision	No ***serious (-1)*** very serious (-2)	Due to low number of events (true positive + false negatives), most studies have very large confidence intervals, particularly for sensitivity	
Publication bias	Unlikely ***likely (-1)*** very likely (-2)	Test for asymmetry statistically significant	
Large effect	Large (+1) Very large (+2)	**No**	
Dose-response gradient	No Yes (+1)	**NA**	
Plausible confounding would change the effect	No Yes (+1)	**NA**	

## Discussion

One of the potential benefits of neuromonitoring is that it allows the surgical team to detect a SCI early on and to institute measures that may potentially reverse or minimize the neurologic deficit. The earlier an injury is detected, the more likely it is that corrective action can be taken to prevent or minimize further damage.

Another potential benefit of neuromonitoring is that it may help to reduce the risk of complications during surgery. Neuromonitoring can also help to improve the accuracy and precision of surgical procedures that involve the spinal cord. By providing real-time information about the function of the spinal cord sensory and motor tracts, neuromonitoring can help the surgical team make more informed decisions about how to proceed with the surgery. This may lead to better outcomes and a lower risk of complications. In addition, neuromonitoring can help to reduce the risk of legal liability for the surgical team. If an SCI occurs during surgery, the surgical team may be held responsible if they did not take appropriate precautions to prevent the injury. By using neuromonitoring, the surgical team can demonstrate that they took additional precautions to minimize the risk of injury and protect the patient's health.

In the current systematic review and meta-analysis, the authors sought to comprehensively summarize all available evidence related to the use of neuromonitoring to detect ISCI. Using novel quantitative statistical methods, we found that all neuromonitoring modalities have acceptable test characteristics as evident from the sROC and AUC. Moreover, we were also able to compute diagnostic test accuracy of each neuromonitoring type for specific disease groups and for specific regions of surgery. We discuss briefly the role of neuromonitoring for specific disease groups.

### Monitoring for Cervical Degenerative Surgery

IONM has been more commonly used in degenerative cervical spine surgery recently, even though the risk of neurological complications is low.^204^ SSEP is currently the IONM modality that is used the most frequently.^205^ It is used in cervical spine surgery not only for assessment of the spinal cord and nerve roots following surgical positioning, but also for the monitoring of sensory tracts throughout the procedure. However, SSEP changes during surgery are not necessarily linked to postoperative neurological impairments due to its low specificity, as demonstrated in our analyses. As a result, many experts advise against using it as the sole monitoring modality in complicated cervical surgeries. This is evident from our results given that the sensitivity of using SSEP alone in cervical spine surgery was only 46%.

Numerous studies have proven that MEPs are reliable at detecting probable neurological damage. In their study, Clark et al found that using MEPs for predicting postoperative impairments in patients undergoing surgery for degenerative cervical myelopathy had a sensitivity of 71% and a specificity of 94%.^59^ In 427 consecutive patients who underwent cervical spine surgery, Hilibrand et al compared the utilization of both SSEP and transcranial MEP (tcMEP) monitoring.^2^ The authors described 12 individuals who had considerable monitoring modifications, 2 of whom were later discovered to have new neurological impairments. Since only one of the 2 patients with a deficiency had SSEP alterations, the authors came to the conclusion that the reported sensitivity and specificity for tcMEP were only 25% and 100%, respectively. However, recent studies have shown more promising results and better diagnostic accuracy for detecting intraoperative injury.^47,49,95,97,157,185^ The pooled results yielded a net sensitivity of 80.2% for MEP for cervical surgery.

In order to increase the effectiveness of IONM during cervical decompression surgery, a combination of SSEP, MEP and EMG has been explored due to the safety issues with the use of only SSEPs and the limitations of MEPs, as previously stated. While a previous qualitative analysis found an overall sensitivity of 50%, our analyses yielded a pooled sensitivity of 81.2%.

Some experts argue against the use of IONM in non-complex cervical spine surgeries, despite the fact that numerous researchers have shown its value. Traynelis et al^206^ concluded that surgical decompression and reconstruction for symptomatic cervical spine disease may be safely carried out without the use of IONM after conducting a retrospective examination of 720 patients. Ajiboye et al^207^ likewise discovered no advantage of IONM in the prevention of new postoperative neurological problems following anterior cervical surgery, supporting this study's findings. Our analyses, when restricted to studies investigating the use of multimodal monitoring for cervical spine and non-complex degenerative diseases, yielded a sensitivity of 62.7%. Therefore, there is ongoing debate in the spine community over whether monitoring is necessary for routine, non-complex cases.

### Monitoring for Deformity Surgery

Several studies have highlighted the importance of using IONM in spinal deformity surgery. The incidence of neurological problems following scoliosis surgery has decreased dramatically since the 1970s, when SSEP monitoring was first introduced.^208^ A large study by Nuwer et al published the findings of a survey by the Scoliosis Research Society (SRS), which asked its members to submit information on the surgical outcomes of patients who had undergone surgery, including the use of IONM. With stated sensitivity of 92% and specificity of 98.9%, SSEP monitoring was used in 51263 of 97586 spinal cases (53%), and positive and negative predictive values were 42% and 99.9%, respectively. However, given that this study did not provide 2 × 2 data for TP, FP, FN and TN, it was not included in our analyses. Nevertheless, our results showed optimum performance of all modalities for detecting ISCI during deformity surgery; 94% sensitivity for SSEP, 92.4% for MEP, and 98.8% for multimodal neuromonitoring.

### Degenerative Lumbar Surgery

Although IONM is frequently employed in the treatment of spinal deformity in the present day, its application in degenerative lumbar surgery, particularly in straightforward procedures, is still debatable.^209^ Supporters of IONM emphasize the technology’s significance in accurately identifying spinal nerve root damage, particularly in revision and instrumented fusion cases.^48,164,210,211^ The issue of monitoring spinal nerve root function is still debatable despite developments.^209,212^ Additionally, although numerous studies have supported the use of IONM in lumbar fusion surgery, it is still unclear whether the improved detection of crisis events intraoperatively translates to a decreased rate of postoperative neurological deficit.^213^ Our results indicate that while EMG has poor sensitivity for most other surgeries, its role in nerve root monitoring for degenerative spine surgeries employing pedicle screw fixation and instrumentation in most cases is still valuable, with a pooled sensitivity of 76.2%.

### Spinal Tumor Surgery

The value of neuromonitoring for spinal tumor surgery is also well established. A previous systematic review by Rijs et al included 14 studies and reported a pooled sensitivity of 80.8% for SSEP, 83.8% for MEP, and 83.5% for multimodal monitoring. We were able to identify 29 studies reporting the results of various neuromonitoring techniques for tumor surgery. We found the pooled sensitivity of SSEP for tumor surgery to be 33.2% based on 5 studies; 85.4% for MEP based on 12 studies; and 88.6% for multimodal based on 17 studies. Moreover, we were also able to parse out the statistics based on type of tumors, ie intra-medullary vs extra-medullary. Among patients undergoing surgery for intra-medullary tumors, the sensitivity for MEP monitoring was 74% and for multimodal was 81.4%. For extra-medullary tumors, the sensitivity for MEP and multimodal monitoring were 85.9% and 82.9%, respectively.

It is clear that IONM is a tool with significant usefulness when removing spinal cord tumors. It provides the surgeon with crucial knowledge about potential spinal cord damage and has enabled more complete tumor resections. Its important to remember that IONM is a tool and not a cure. Its value varies depending on the particular circumstance and how the surgeon applies the knowledge from the IONM alert to the resection technique. However, it would be much more useful to gather information with a longer follow-up on both the neurologic result and the quality of life in the present era of value-based health care.^11^

## Conclusion

The present systematic review and meta-analysis has summarized the role of neuromonitoring for detecting ISCI during spine surgery. Our results indicate that there is low level evidence that all neuromonitoring modalities have acceptable performance in terms of detecting ISCI, particularly for high-risk spinal surgery. It is therefore recommended that some form of neuromonitoring be employed, particularly in high-risk spinal surgery.

## Supplemental Material

Supplemental Material - Accuracy of Intraoperative Neuromonitoring in the Diagnosis of Intraoperative Neurological Decline in the Setting of Spinal Surgery—A Systematic Review and Meta-Analysis Supplemental Material for Accuracy of Intraoperative Neuromonitoring in the Diagnosis of Intraoperative Neurological Decline in the Setting of Spinal Surgery—A Systematic Review and Meta-Analysis by Mohammed Ali Alvi, Brian K. Kwon, Nader Hejrati, Lindsay A. Tetreault, Nathan Evaniew, Andrea C. Skelly, and Michael G. Fehlings in Global Spine Journal
