# Supplementary material for: Accuracy of Intraoperative Neuromonitoring in the Diagnosis of Intraoperative Neurological Decline in the Setting of Spinal Surgery—A Systematic Review and Meta-Analysis
Source: Global Spine J. 2024 Mar 25;14(3 Suppl):105S–149S. doi: 10.1177/21925682231196514 (PMC10964897; doi:10.1177/21925682231196514)
Supplement: Supplemental Material - Accuracy of Intraoperative Neuromonitoring in the Diagnosis of Intraoperative Neurological Decline in the Setting of Spinal Surgery—A Systematic Review and Meta-Analysis [file sj-pdf-1-gsj-10.1177_21925682231196514.pdf]

**Supplemental Table: List of Excluded Studies**

| Covidence # | Study | Title | Authors | Journal | Reason for Exclusion |
|---|---|---|---|---|---|
| #10 | Zarchi 2022 | A practical method for real-time detection of pedicle wall breaching during funneling | Zarchi O.; Ohana N.; Mercado E.; Amitai A.; Berestizshevsky Y.; Sheinis D.; Benharroch D.; Bar-On E. | Archives of Orthopaedic and Trauma Surgery | Wrong Outcome |
| #15 | Puvanesarajah 2022 | The deformity angular ratio: can three-dimensional computed tomography improve prediction of intraoperative neuromonitoring events? | Puvanesarajah V.; Marciano G.F.; Hassan F.M.; Lee N.J.; Thuet E.D.; Lombardi J.M.; Sardar Z.M.; Lehman R.A.; Lenke L.G. | Spine Deformity | Wrong Outcome |
| #46 | Jamaludin 2022 | Machine Learning Application of Transcranial Motor-Evoked Potential to Predict Positive Functional Outcomes of Patients | Jamaludin M.R.; Lai K.W.; Chuah J.H.; Zaki M.A.; Hasikin K.; Abd Razak N.A.; Dhanalakshmi S.; Saw L.B.; Wu X. | Computational intelligence and neuroscience | Wrong Outcome |
| #57 | Montes 2022 | Pulse-train versus single-pulse t-EMG stimulation for intraoperative neurophysiologic monitoring | Montes E.; de Blas G.; Barrios C.; Mariscal G.; Burgos J.; Regidor I.; Hevia E. | Clinical Neurology and Neurosurgery | Wrong Outcome |

For Peer Review

| | | | | | |
|---|---|---|---|---|---|
| | | of thoracic pedicle screws in adolescent idiopathic scoliosis | | | |
| #72 | BeaulieuLalanne 2022 | Triggered electromyography (tEMG) in the lumbar spine Its reliable? Correlation of tEMG with postoperative CT images in lumbar instrumentation | Beaulieu Lalanne L.; Larrondo Carmona R.; Alvarez Lemos F.; Oelker Behn C.; Caceres Copetta M.; Munoz Wilson J.T.; Beaulieu Montoya A.M.; Larrondo Martinez V. | Revista espanola de cirugia ortopedica y traumatologia | Full Text Not in English |
| #78 | Shao 2021 | Efficacy and safety for combination of t-EMG with O-arm assisted pedicle screw placement in neurofibromatosis type I scoliosis surgery | Shao X.; Huang Z.; Deng Y.; Yang J.; Sui W. | Journal of orthopaedic surgery and research | Wrong Outcome |
| #83 | Chang 2021 | Diagnostic accuracy of ssep changes during lumbar spine surgery for predicting postoperative neurological deficit: A systematic review and meta-analysis | Chang R.; Reddy R.P.; Coutinho D.V.; Chang Y.-F.; Anetakis K.M.; Crammond D.J.; Balzer J.R.; Thirumala P.D. | Spine | Wrong Study Design |

For Peer Review

| | | | | | |
|---|---|---|---|---|---|
| #99 | Hiyama 2022 | Utility of Power Tool and Intraoperative Neuromonitoring for Percutaneous Pedicle Screw Placement in Single Position Surgery: A Technical Note | Hiyama A.; Nomura S.; Sakai D.; Watanabe M. | World Neurosurgery | Wrong Intervention |
| #116 | Zheng 2021 | Preoperative electrophysiologic assessment of C5-innervated muscles in predicting C5 palsy after posterior cervical decompression | Zheng C.; Nie C.; Zhu Y.; Xu M.; Lyu F.; Jiang J.; Xia X. | European Spine Journal | Wrong Patient Population |
| #177 | Wu 2020 | Application of different thresholds for instrumentation device testing in minimally invasive lumbosacral spine fixation | Wu Y.; Cohen D.; Tellez M.J.; DiGiacinto G.V.; Barquero A.V.; Ulkatan S. | Journal of Clinical Neuroscience | Wrong Outcome |
| #183 | Jasiukaitis 2020 | Trending algorithm discriminates hemodynamic from injury related TcMEP amplitude loss | Jasiukaitis P.; Lyon R. | Journal of Clinical Monitoring and Computing | Wrong Outcome |

For Peer Review

| | | | | | |
|---|---|---|---|---|---|
| #203 | Ravindra 2019 | Electromyography stimulation compared with intraoperative O-arm imaging for evaluating pedicle screw breaches in lumbar spine surgery: a prospective analysis of 1006 screws in 164 patients | Ravindra V.M.; Kalra R.R.; Dailey A.T. | Spine Journal | Wrong Outcome |
| #250 | Zakaria 2018 | Mechanomyography for Intraoperative Assessment of Cortical Breach During Instrumented Spine Surgery | Zakaria H.M.; Tundo K.M.; Sandles C.; Chuang M.; Schultz L.; Aho T.; Abdulhak M. | World Neurosurgery | Wrong Intervention |
| #266 | Zheng 2017 | Screw Placement at the Apex Alters Surgical Outcomes of Moderate Lenke 1 Adolescent Idiopathic Scoliosis | Zheng X.; Qian B.-P.; Liu Z.; Sun X.; Zhu Z.-Z.; Wang B.; Yu Y.; Qiu Y. | Clinical Spine Surgery | Wrong Outcome |
| #267 | Kaliya-Perumal 2017 | Intraoperative electromyographic monitoring to optimize safe lumbar pedicle screw placement - a retrospective analysis | Kaliya-Perumal A.-K.; Charng J.-R.; Niu C.-C.; Tsai T.-T.; Lai P.-L.; Chen L.-H.; Chen W.-J. | BMC Musculoskeletal Disorders | Wrong Intervention |

For Peer Review

| | | | | | |
|---|---|---|---|---|---|
| #270 | Ajiboye 2017 | Utility of Intraoperative Neuromonitoring for Lumbar Pedicle Screw Placement Is Questionable | Ajiboye R.M.; Zoller S.D.; D'Oro A.; Burke Z.D.; Sheppard W.; Wang C.; Buser Z.; Wang J.C.; Pourtaheri S. | Spine | Wrong Outcome |
| #299 | Kassis 2016 | Combining pedicle screw stimulation with spinal navigation, a protocol to maximize the safety of neural elements and minimize radiation exposure in thoracolumbar spine instrumentation | Kassis S.Z.; Abukwedar L.K.; Msaddi A.K.; Majer C.N.; Othman W. | European Spine Journal | Wrong Outcome |
| #320 | Thirumala 2016 | Diagnostic accuracy of combined multimodality somatosensory evoked potential and transcranial motor evoked potential intraoperative monitoring in patients with idiopathic scoliosis | Thirumala P.D.; Huang J.; Thiagarajan K.; Cheng H.; Balzer J.; Crammond D.J. | Spine | Wrong Study Design |

For Peer Review

| | | | | | |
|---|---|---|---|---|---|
| #365 | Wood 2014 | The surgical learning curve and accuracy of minimally invasive lumbar pedicle screw placement using CT based computer-assisted navigation plus continuous electromyography monitoring - A retrospective review of 627 screws in 150 patients | Wood M.J.; McMillen J. | International Journal of Spine Surgery | Wrong Outcome |
| #377 | Azabou 2014 | Predicting intraoperative feasibility of combined TES-mMEP and cSSEP monitoring during scoliosis surgery based on preoperative neurophysiological assessment | Azabou E.; Manel V.; Abelin-Genevois K.; Andre-Obadia N.; Cunin V.; Garin C.; Kohler R.; Berard J.; Ulkatan S. | Spine Journal | Wrong Outcome |
| #393 | Calancie 2014 | Implantation of thoracic pedicle screws: A blinded and randomized clinical study. Part 1. Methods and alarm criteria Clinical article | Calancie B.; Donohue M.L.; Harris C.B.; Canute G.W.; Singla A.; Wilcoxen K.G.; Moquin R.R. | Journal of Neurosurgery: Spine | Wrong Outcome |

For Peer Review

| | | | | | |
|---|---|---|---|---|---|
| #394 | Calancie 2014 | Neuromonitoring with pulse-train stimulation for implantation of thoracic pedicle screws: a blinded and randomized clinical study. Part 1. Methods and alarm criteria | Calancie B.; Donohue M.L.; Harris C.B.; Canute G.W.; Singla A.; Wilcoxen K.G.; Moquin R.R. | Journal of neurosurgery. Spine | Wrong Outcome |
| #397 | Kulik 2013 | A CT-based study investigating the relationship between pedicle screw placement and stimulation threshold of compound muscle action potentials measured by intraoperative neurophysiological monitoring | Kulik G.; Pralong E.; McManus J.; Debatisse D.; Schizas C. | European Spine Journal | Wrong Outcome |
| #412 | Ney 2013 | Cost-benefit analysis: Intraoperative neurophysiological monitoring in spinal surgeries | Ney J.P.; Van Der Goes D.N.; Watanabe J.H. | Journal of Clinical Neurophysiology | Wrong Study Design |
| #422 | Fotakopoulos 2013 | The value of transcranial motor-evoked potentials and free-running electromyography in surgery for cervical disc herniation | Fotakopoulos G.; Alexiou G.A.; Pachatouridis D.; Karagiorgiadis D.; Konitsiotis S.; Kyritsis A.P.; Voulgaris S. | Journal of Clinical Neuroscience | Wrong Outcome |

For Peer Review

| | | | | | |
|---|---|---|---|---|---|
| #429 | Gazzeri 2013 | Safety of intraoperative electrophysiological monitoring (TES and EMG) for spinal and cranial lesions | Gazzeri R.; Faiola A.; Neroni M.; Fiore C.; Callovini G.; Pischedda M.; Galarza M. | Surgical technology international | Full Text not available |
| #431 | Hwang 2012 | Intraoperative Neurophysiological Monitoring in Spine Deformity Surgery | Hwang S.W.; Malhotra N.R.; Shaffrey C.I.; Samdani A.F. | Spine Deformity | Wrong Study Design |
| #441 | Feng 2012 | Minimally invasive transforaminal lumbar interbody fusion aided with computer-assisted spinal navigation system combined with electromyography monitoring | Feng L.; Wei L.; Fan Z.; Tie L.; Du X.-L.; Chen A.-M. | Chinese Medical Journal | Wrong Outcome |
| #458 | Parker 2011 | Ability of electromyographic monitoring to determine the presence of malpositioned pedicle screws in the lumbosacral spine: Analysis of 2450 consecutively placed screws - Clinical article | Parker S.L.; Amin A.G.; Farber S.H.; McGirt M.J.; Sciubba D.M.; Wolinsky J.-P.; Bydon A.; Gokaslan Z.L.; Witham T.F. | Journal of Neurosurgery: Spine | Wrong Outcome |

For Peer Review

| | | | | | |
|---|---|---|---|---|---|
| #464 | Haghighi 2011 | Correlation between transcranial motor and somatosensory-evoked potential findings in cervical myelopathy or radiculopathy during cervical spine surgery | Haghighi S.S.; Mundis G.; Zhang R.; Ramirez B. | Neurological Research | Wrong Outcome |
| #469 | Gavaret 2011 | Intraoperative monitoring in pediatric orthopedic spinal surgery: Three hundred consecutive monitoring cases of which 10% of patients were younger than 4 years of age | Gavaret M.; Trebuchon A.; Aubert S.; Jacopin S.; Blondel B.; Glard Y.; Jouve J.-L.; Bollini G. | Spine | Paediatric population |
| #473 | Mavrogenis 2009 | Accuracy of pedicle screw placement using intraoperative neurophysiological monitoring and computed tomography | Mavrogenis A.F.; Papagelopoulos P.J.; Korres D.S.; Papadopoulos K.; Sakas D.E.; Pneumaticos S. | Journal of Long-Term Effects of Medical Implants | Wrong Outcome |
| #474 | Alemo 2010 | Role of intraoperative neurophysiologic monitoring in lumbosacral spine fusion and instrumentation: A retrospective study | Alemo S.; Sayadipour A. | World Neurosurgery | Wrong Outcome |

For Peer Review

| | | | | | |
|---|---|---|---|---|---|
| #481 | Samdani 2010 | Learning curve for placement of thoracic pedicle screws in the deformed spine | Samdani A.F.; Ranade A.; Saldanha V.; Yondorf M.Z. | Neurosurgery | Wrong Outcome |
| #490 | Yamamoto 2010 | Efficacy and limitations of intraoperative spinal cord monitoring using nasopharyngeal tube electrodes | Yamamoto N.; Kobashi H.; Shiba M.; Itoh T. | Journal of Neurosurgery: Spine | Wrong Intervention |
| #505 | Hu 2007 | Time-frequency feature of intraoperative somatosensory evoked potential signals | Hu Y.; Jiang F.; Luk K.D. | Conference proceedings : ... Annual International Conference of the IEEE Engineering in Medicine and Biology Society. IEEE Engineering in Medicine and Biology Society. Conference | Wrong Study Design |
| #522 | Kim 2008 | Free-hand pedicle screw placement during revision spinal surgery: Analysis of 552 screws | Kim Y.-W.; Lenke L.G.; Kim Y.J.; Bridwell K.H.; Kim Y.B.; Watanabe K. | Spine | Wrong Outcome |

For Peer Review

| | | | | | |
|---|---|---|---|---|---|
| #527 | Rodriguez-Olaverri 2008 | Using triggered electromyographic threshold in the intercostal muscles to evaluate the accuracy of upper thoracic pedicle screw placement (T3-T6) | Rodriguez-Olaverri J.C.; Zimick N.C.; Merola A.; De Blas G.; Burgos J.; Piza-Vallespir G.; Hevia E.; Vicente J.; Sanper I.; Domenech P.; Regidor I. | Spine | Wrong Outcome |
| #547 | Djurasovic 2005 | A prospective analysis of intraoperative electromyographic monitoring of posterior cervical screw fixation | Djurasovic M.; Dimar 2nd. J.R.; Glassman S.D.; Edmonds H.L.; Carreon L.Y. | Journal of spinal disorders & techniques | Wrong Outcome |
| #552 | Huitema 2006 | Screw position after double-rod anterior spinal fusion in idiopathic scoliosis: An evaluation using computerized tomography | Huitema G.C.; Van Rhijn L.W.; Van Ooij A. | Spine | Wrong Intervention |
| #600 | MacDonald 2003 | Monitoring scoliosis surgery with combined multiple pulse transcranial electric motor and cortical somatosensory-evoked potentials from the lower and upper extremities | MacDonald D.B.; Al Zayed Z.; Khoudeir I.; Stigsby B. | Spine | Wrong Outcome |

For Peer Review

| | | | | | |
|---|---|---|---|---|---|
| #611 | Kobara 2000 | Relative effectiveness of electrically- vs mechanically-elicited EMGs in detecting pedicle wall perforation and surgically-induced nerve root damage | Kobara N.; Owen J.H.; Kostuik J.; Huckell C.; Tooke S.M. | Fukuoka igaku zasshi = Hukuoka acta medica | Wrong Outcome |
| #613 | Akay 2002 | Continuous neural monitoring in lumbar spine surgery: Experience with 101 patients | Akay K.M.; Onder S. | Minimally Invasive Neurosurgery | Wrong Outcome |
| #621 | Zileli 2002 | Diagnostic value of electrical stimulation of lumbosacral roots in lumbar spinal stenosis | Zileli B.; Ertekin C.; Zileli M.; Yunten N. | Acta Neurologica Scandinavica | Wrong Patient Population |
| #625 | Pereon 2002 | Combined spinal cord monitoring using neurogenic mixed evoked potentials and collision techniques | Pereon Y.; Nguyen The Tich S.; Delecrin J.; Pham Dang C.; Bodin J.; Drouet J.-C.; Passuti N. | Spine | Wrong Outcome |
| #627 | Reidy 2001 | Evaluation of electromyographic monitoring during insertion of thoracic pedicle screws | Reidy D.P.; Houlden D.; Nolan P.C.; Kim M.; Finkelstein J.A. | Journal of Bone and Joint Surgery - Series B | Wrong Outcome |

For Peer Review

| | | | | | |
|---|---|---|---|---|---|
| #631 | Hu 2001 | Comparison of time-frequency distribution techniques for analysis of spinal somatosensory evoked potential | Hu Y.; Luk K.D.K.; Lu W.W.; Holmes A.; Leong J.C.Y. | Medical and Biological Engineering and Computing | Wrong Outcome |
| #637 | Bosnjak 2000 | Electrical thresholds for biomechanical response in the ankle to direct stimulation of spinal roots L4, L5, and S1: Implications for intraoperative pedicle screw testing | Bosnjak R.; Dolenc V.V. | Spine | Wrong Intervention |
| #639 | Schwartz 2000 | Prevention of positional brachial plexopathy during surgical correction of scoliosis | Schwartz D.M.; Drummond D.S.; Hahn M.; Ecker M.L.; Dormans J.P. | Journal of Spinal Disorders | Wrong Outcome |
| #648 | Luk 1999 | Variability of somatosensory-evoked potentials in different stages of scoliosis surgery | Luk K.D.K.; Hu Y.; Wong Y.W.; Leong J.C.Y. | Spine | Wrong Outcome |
| #662 | Lang 1996 | Myogenic motor-evoked potential monitoring using partial neuromuscular blockade in surgery of the spine | Lang E.W.; Beutler A.S.; Chesnut R.M.; Patel P.M.; Kennelly N.A.; Kalkman C.J.; Drummond J.C.; Garfin S.R. | Spine | Wrong Outcome |

For Peer Review

| | | | | | |
|---|---|---|---|---|---|
| #666 | Nagle 1996 | Intraoperative monitoring of motor evoked potentials: A review of 116 cases | Nagle K.J.; Emerson R.G.; Adams D.C.; Heyer E.J.; Roye D.P.; Schwab F.J.; Weidenbaum M.; McCormick P.; Pile-Spellman J.; Stein B.M.; Farcy J.P.; Gallo E.J.; Dowling K.C.; Turner C.A. | Neurology | Wrong Outcome |
| #670 | Epstein 1996 | Somatosensory evoked potential monitoring (SSEPs) in 173 cervical operations | Epstein N.E. | Neuro-Orthopedics | Wrong Outcome |
| #672 | Clements 1996 | Evoked and spontaneous electromyography to evaluate lumbosacral pedicle screw placement | Clements D.H.; Morledge D.E.; Martin W.H.; Betz R.R. | Spine | Wrong Outcome |
| #690 | Friedman 1987 | Somatosensory evoked potentials after sequential extremity stimulation: A new method for improved monitoring accuracy | Friedman W.A.; Curran M.T. | Neurosurgery | Wrong Outcome |
| #692 | Mostegl 1984 | The application of somatosensory-evoked potentials in orthopedic spine surgery | Mostegl A.; Bauer R. | Archives of Orthopaedic and Traumatic Surgery | Wrong Outcome |

For Peer Review

| | | | | | |
|---|---|---|---|---|---|
| #724 | DiMaria 2022 | Patient Factors Impacting Baseline Motor Evoked Potentials (MEPs) in Patients Undergoing Cervical Spine Surgery for Myelopathy or Radiculopathy. | DiMaria, Stephen; Wilent, W.; Nicholson, Kristen; Tesdahl, Eric; Valiuskyte, Kornelija; Mao, Jennifer; Seger, Philip; Singh, Akash; Sestokas, Anthony; Vaccaro, Alex; MD, PhD | | Wrong Outcome |
| #726 | Li 2022 | Safe Electromyography Stimulation Thresholds Within Kambin's Triangle During Endoscopic Transforaminal Lumbar Interbody Fusion. | Li, Yingda; Wang, Michael | | Wrong Outcome |
| #781 | Kiridly 2021 | Positioning-Related Peripheral Nerve Injury During Spine Surgery and the Role of Intraoperative Neuromonitoring. | Kiridly, Daniel; MD, MBA; Satin, Alexander; Derman, Peter; MD, MBA | | Wrong Study Design |

For Peer Review

| | | | | | |
|---|---|---|---|---|---|
| #787 | Kobayashi 2021 | Characteristics of Cases with Poor Transcranial Motor-evoked Potentials Baseline Waveform Derivation in Spine Surgery: A Prospective Multicenter Study of the Monitoring Committee of the Japanese Society for Spine Surgery and Related Research. | Kobayashi, Kazuyoshi; Imagama, Shiro; Ando, Kei; Yoshida, Go; Ando, Muneharu; Kawabata, Shigenori; Yamada, Kei; Kanchiku, Tsukasa; Fujiwara, Yasushi; Taniguchi, Shinichirou; Iwasaki, Hiroshi; Shigematsu, Hideki; Tadokoro, Nobuaki; Takahashi, Masahito; Wada, Kanichiro; Yamamoto, Naoya; Funaba, Masahiro; Yasuda, Akimasa; Ushirozako, Hiroki; Hashimoto, Jun; Morito, Shinji; Takatani, Nobunori; Tani, Toshikazu; Matsuyama, Yukihiro | | Wrong Outcome |

For Peer Review

| | | | | | |
|---|---|---|---|---|---|
| #797 | Liu 2021 | The Prognostic Value of Intraoperative Neuromonitoring by Combining Somatosensory- and Motor-Evoked Potentials for Thoracic Spinal Decompression Surgery in Patients with Neurological Deficit. | Liu, Tun; Dong, Buhuai; Qi, Huaguang; Yan, Liang; MD, PhD; Zhao, Songchuan; Liu, Zhian; Liu, Xuemei; Wang, Fei; Li, Liang; Cai, Wenbo; Luo, Zhenguo; Wang, Gang | | Wrong Outcome |
| #799 | Zheng 2021 | Comparison of Intraoperative Neuromonitoring Outcome in Treating Thoracic Ossification of the Ligamentum Flavum Through En Bloc Versus Piecemeal Laminectomy. | Zheng, Chaojun; Nie, Cong; Zhu, Yu; Tang, Susu; Jin, Xiang; Lyu, Feizhou; Jiang, Jianyuan; Ma, Xiaosheng | | Wrong Outcome |
| #807 | Zhang 2021 | Multi-modal Neuroelectrophysiological Monitoring in the Treatment of Thoracic Tuberculosis with Debridement and Bone Grafting and Posterior Pedicle Screw Fixation via Costal Transverse Process Approach. | Zhang, Chen-wei; Shi, Shi-yuan; Tao, Xiao; Hu, Jin-ping; Cao, Tian-yi; Fei, Jun | | Wrong Outcome |

For Peer Review

| | | | | | |
|---|---|---|---|---|---|
| #816 | Tanaka 2021 | Effect of Compound Muscle Action Potential After Peripheral Nerve Stimulation Normalization on Anesthetic Fade of Intraoperative Transcranial Motor-Evoked Potential. | Tanaka, Satoshi; Watanabe, Tomoko; Takanashi, Junko; Oka, Hidehiro; Hashimoto, Ryo; Akimoto, Jiro | | Wrong Patient Population |
| #836 | Modi 2021 | Clinical Correlation of Intraoperative Neuromonitoring in 319 Individuals Undergoing Posterior Decompression and Fixation of Spine. | Modi, Hitesh;  MD, MS; Goel, Shakti; Desai, Yatin; Modi, Poonam | | Wrong Outcome |
| #851 | Toll 2021 | Congenital Scoliosis of the Pediatric Cervical Spine: Characterization of a 17-Patient Operative Cohort. | Toll, Brandon; Samdani, Amer; Amanullah, Amir; Pahys, Joshua; Janjua, Muhammad; Hwang, Steven | | Wrong Outcome |
| #856 | Magampa 2021 | Surgeon-directed transcranial motor evoked potential spinal cord monitoring in spinal deformity surgery: A REVIEW OF VIABILITY, SAFETY, AND EFFICACY. | Magampa, R.; Dunn, R. | | Wrong Outcome |

For Peer Review

| | | | | | |
|---|---|---|---|---|---|
| #859 | Kobayashi 2021 | Efficacy of Intraoperative Intervention Following Transcranial Motor-evoked Potentials Alert During Posterior Decompression and Fusion Surgery for Thoracic Ossification of the Posterior Longitudinal Ligament: A Prospective Multicenter Study of the Monitor | Kobayashi, Kazuyoshi; MD, PhD; Imagama, Shiro; Yoshida, Go; Ando, Muneharu; Kawabata, Shigenori; Yamada, Kei; Kanchiku, Tsukasa; Fujiwara, Yasushi; Taniguchi, Shinichirou; Iwasaki, Hiroshi; Tadokoro, Nobuaki; Takahashi, Masahito; Wada, Kanichiro; Yamamoto, Naoya; Shigematsu, Hideki; Funaba, Masahiro; Yasuda, Akimasa; Ushirozako, Hiroki; Tani, Toshikazu; Matsuyama, Yukihiro | | Wrong Outcome |
| #965 | Tanaka 2019 | Muscle-evoked Potentials After Electrical Stimulation to the Brain in Patients Undergoing Spinal Surgery are Less Affected by Anesthetic Fade With Constant-voltage Stimulation Than With Constant-current Stimulation. | Tanaka, Masato; Shigematsu, Hideki; MD, PhD; Kawaguchi, Masahiko; Hayashi, Hironobu; Takatani, Tsunenori; MT, PhD; Iwata, Eiichiro; Okuda, Akinori; Morimoto, Yasuhiko; Kawasaki, Sachiko; Masuda, Keisuke; Yamamoto, Yusuke; Tanaka, Yasuhito | | Wrong Outcome |

For Peer Review

| | | | | | |
|---|---|---|---|---|---|
| #978 | Wang 2019 | Survivals of the Intraoperative Motor-evoked Potentials Response in Pediatric Patients Undergoing Spinal Deformity Correction Surgery: What Are the Neurologic Outcomes of Surgery?. | Wang, Shujie; Li, Chaoxiong; Guo, Lanjun; Hu, Haimei; Jiao, Yang; Shen, Jianxiong; Tian, Ye; Zhang, Jianguo | | Paediatric population |
| #1000 | Levin 2019 | Intraoperative neuromonitoring in paediatric spinal surgery. | Levin, D.; Strantzas, S.; Steinberg, B. | | Wrong Study Design |
| #1042 | Ando 2019 | Wave Change of Intraoperative Transcranial Motor-Evoked Potentials During Corrective Fusion for Syndromic and Neuromuscular Scoliosis. | Ando, Kei; Kobayashi, Kazuyoshi; Ito, Kenyu; Tsushima, Mikito; Morozumi, Masayoshi; Tanaka, Satoshi; Machino, Masaaki; Ota, Kyotaro; Nishida, Yoshihiro; Ishiguro, Naoki; Imagama, Shiro | | Wrong Outcome |
| #1064 | Ashayeri 2018 | Retrospective Analysis of EMG-evoked Potentials in Cortical Bone Trajectory Pedicle Screws. | Ashayeri, Kimberly; Sahasrabudhe, Nikhil; Galic, Vladimir; Beric, Aleksandar; Smith, Michael | | Wrong Outcome |

For Peer Review

| | | | | | |
|---|---|---|---|---|---|
| #1067 | Li 2018 | Clinical outcomes of posterior pedicle screw instrumentation without osteotomy in the management of adolescent idiopathic scoliosis. | Li, Ning; Xu, Chen; Shen, Ming-kui; Luo, Ming; Wang, Jie; Xia, Lei | | Wrong Outcome |
| #1073 | Chen 2018 | Factors Associated With Inadequate Intraoperative Baseline Lower Extremity Somatosensory Evoked Potentials. | Chen, Jonathan; Shilian, Parastou; Cheongsiatmoy, Justin; Gonzalez, Andres | | Wrong Outcome |
| #1074 | Berends 2018 | Influence of the Montage of Stimulation Electrodes for Intraoperative Neuromonitoring During Orthopedic Spine Surgery. | Berends, Hanneke; Journee, Henricus | | Wrong Outcome |

For Peer Review

| | | | | | |
|---|---|---|---|---|---|
| #1090 | Kato 2018 | An Analysis of the Incidence and Outcomes of Major Versus Minor Neurological Decline After Complex Adult Spinal Deformity Surgery: A Subanalysis of Scoli-RISK-1 Study. | Kato, So; Fehlings, Michael; MD, PhD; FACS, FRCSC; Lewis, Stephen; MD, MSc; Lenke, Lawrence; Shaffrey, Christopher; Cheung, Kenneth; Carreon, Leah; Dekutoski, Mark; Schwab, Frank; Boachie-Adjei, Oheneba; Kebaish, Khaled; Ames, Christopher; Qiu, Yong; Matsuyama, Yukihiro; Dahl, Benny; Mehdian, Hossein; Pellise, Ferran; Berven, Sigurd | | Wrong Outcome |
| #1163 | Wang 2017 | Proper Responding Strategies to Neuromonitoring Alerts During Correction Step in Posterior Vertebral Column Resection Patients With Severe Rigid Deformities Can Reduce Postoperative Neurologic Deficits. | Wang, Yingsong; Xie, Jingming; Zhao, Zhi; Li, Tao; Bi, Ni; Zhang, Ying; Shi, Zhiyue | | Wrong Outcome |

For Peer Review

| | | | | | |
|---|---|---|---|---|---|
| #1169 | Hadley 2017 | Guidelines for the Use of Electrophysiological Monitoring for Surgery of the Human Spinal Column and Spinal Cord. | Hadley, Mark; Shank, Christopher; Rozzelle, Curtis; Walters, Beverly; C MD, MSc | | Wrong Study Design |
| #1192 | Cheah 2017 | Intraoperative Use of Neuromonitoring in Multilevel Thoracolumbar Spine Instrumentation and the Effects on Postoperative Neurological Injuries. | Cheah, Jonathan; Zhang, Alan; Tay, Bobby | | Wrong Outcome |
| #1197 | Bartley 2017 | Perioperative and Delayed Major Complications Following Surgical Treatment of Adolescent Idiopathic Scoliosis. | Bartley, Carrie; Yaszay, Burt; Bastrom, Tracey; Shah, Suken; Lonner, Baron; Asghar, Jahangir; Miyanji, Firoz; Samdani, Amer; Newton, Peter | | Wrong Intervention |
| #1198 | Ajiboye 2017 | Utility of Intraoperative Neuromonitoring for Lumbar Pedicle Screw Placement Is Questionable: A Review of 9957 Cases. | Ajiboye, Remi; Zoller, Stephen; D'Oro, Anthony; Burke, Zachary; Sheppard, William; Wang, Christopher; Buser, Zorica; Wang, Jeffrey; Pourtaheri, Sina | | Wrong Outcome |

For Peer Review

| | | | | | |
|---|---|---|---|---|---|
| #1225 | Kim 2017 | Perioperative Neurologic Complications in Adult Spinal Deformity Surgery: Incidence and Risk Factors in 564 Patients. | Kim, Han; Iyer, Sravisht; Zebala, Luke; Kelly, Michael; Sciubba, Daniel; Protopsaltis, Themistocles; Gupta, Munish; Neuman, Brian; Mundis, Gregory; Ames, Christopher; Smith, Justin;  MD, PhD; Hart, Robert; Burton, Douglas; Klineberg, Eric | | Wrong Outcome |
| #1231 | Appel 2017 | Efficacy of Head Repositioning in Restoration of Electrophysiological Signals During Cervical Spine Procedures. | Appel, Shmuel; Korn, Akiva; Biron, Tali; Goldstein, Kobi; Rand, Nahshon; Millgram, Michael; Floman, Yizhar; Ashkenazi, Ely | | Wrong Outcome |
| #1246 | Ajiboye 2017 | Routine Use of Intraoperative Neuromonitoring During ACDFs for the Treatment of Spondylotic Myelopathy and Radiculopathy Is Questionable: A Review of 15,395 Cases. | Ajiboye, Remi; D'Oro, Anthony; Ashana, Adedayo; Buerba, Rafael; Lord, Elizabeth; Buser, Zorica; Wang, Jeffrey; Pourtaheri, Sina | | Wrong Outcome |

For Peer Review

| | | | | | |
|---|---|---|---|---|---|
| #1276 | Wang 2016 | Deformity Angular Ratio Describes the Severity of Spinal Deformity and Predicts the Risk of Neurologic Deficit in Posterior Vertebral Column Resection Surgery. | Wang, Xiao-bin; Lenke, Lawrence; Thuet, Earl; BS, CNIM; Blanke, Kathy; Koester, Linda; Roth, Michael | | Wrong Outcome |
| #1277 | Samdani 2016 | Reversible Intraoperative Neurophysiologic Monitoring Alerts in Patients Undergoing Arthrodesis for Adolescent Idiopathic Scoliosis: What Are the Outcomes of Surgery?. | Samdani, Amer; Bennett, James; Ames, Robert; Asghar, Jahangir; Orlando, Giuseppe; Pahys, Joshua; Yaszay, Burt; Miyanji, Firoz; Lonner, Baron; Lehman, Ronald; Newton, Peter; Cahill, Patrick; Betz, Randal | | Wrong Outcome |
| #1316 | Wang 2016 | Exploration of the Intraoperative Motor Evoked Potential. | Wang, Shujie; Tian, Ye | | Wrong Outcome |
| #1340 | Ney 2015 | Does intraoperative neurophysiologic monitoring matter in noncomplex spine surgeries?. | Ney, John;  MD, MPH; van der Goes, David; Nuwer, Marc; MD, PhD | | Wrong Outcome |

For Peer Review

| | | | | | |
|---|---|---|---|---|---|
| #1352 | Erdogan 2015 | Effective Use of Multimodal Intraoperative Neuromonitoring in Neurosurgery. | Erdogan, Ezgi; Karamursel, Sacit; Kiris, Talat | | Wrong Patient Population |
| #1415 | Gavrancic 2014 | Train-of-Four Test in Intraoperative Neurophysiologic Monitoring: Differences Between Hand and Foot Train-of-Four. | Gavrancic, Brane; Lolis, Athena; Beric, Aleksandar | | Wrong Intervention |
| #1419 | Zhu 2014 | Misplacement Pattern of Pedicle Screws in Pediatric Patients With Spinal Deformity: A Computed Tomography Study. | Zhu, Feng; Sun, Xu; Qiao, Jun; Ding, Yitao; Zhang, Bing; Qiu, Yong | | Wrong Outcome |
| #1422 | Cole 2014 | Intraoperative Neuromonitoring in Single-Level Spinal Procedures: A Retrospective Propensity Score-Matched Analysis in a National Longitudinal Database. | Cole, Tyler; Veeravagu, Anand; Zhang, Michael; Li, Alexander; Ratliff, John;  MD, FACS | | Wrong Outcome |

For Peer Review

| | | | | | |
|---|---|---|---|---|---|
| #1428 | Smith 2014 | Incidence of Lumbar Spine Pedicle Breach After Percutaneous Screw Fixation: A Radiographic Evaluation of 601 Screws in 151 Patients. | Smith, Zachary; Sugimoto, Koichi; Lawton, Cort; Fessler, Richard; MD, PhD | | Wrong Outcome |
| #1431 | Chong 2014 | Direct Comparison of the Effect of Desflurane and Sevoflurane on Intraoperative Motor-evoked Potentials Monitoring. | Chong, Chin; Ted MBBS, MMed; Manninen, Pirjo; MD, FRCPC; Sivanaser, Vanitha; MBBS, MMed; Subramanyam, Rajeev; MD, DNB; MNAMS, MS; Lu, Nancy; Venkatraghavan, Lashmi; MD, FRCA | | Wrong Outcome |
| #1466 | Dede 2014 | Using the Freehand Pedicle Screw Placement Technique in Adolescent Idiopathic Scoliosis Surgery: What Is the Incidence of Neurological Symptoms Secondary to Misplaced Screws?. | Dede, Ozgur; Ward, William; Bosch, Patrick; Bowles, Austin; Roach, James | | Wrong Outcome |

For Peer Review

| | | | | | |
|---|---|---|---|---|---|
| #1482 | Shah 2013 | Transcranial motor evoked potential monitoring outcome in the high-risk brain and spine surgeries: Correlation of clinical and neurophysiological data - An Indian perspective. | Shah, Poornima | | Wrong Outcome |
| #1553 | Imam 2013 | Spinal N22 in diagnosing lumbosacral radiculopathy. | Imam, Mohamed; Hassan, Marwa | | Wrong Intervention |
| #1578 | Michael 2012 | Intraoperative Neuromonitoring in Spine Surgery. | Michael, Keith; Boden, Scott | | Wrong Study Design |
| #1610 | deBlas 2012 | Safe Pedicle Screw Placement in Thoracic Scoliotic Curves Using t-EMG: Stimulation Threshold Variability at Concavity and Convexity in Apex Segments. | de Blas, Gema; MD, PhD; Barrios, Carlos; Regidor, Ignacio; Montes, Elena; Burgos, Jesus; Piza-Vallespir, Gabriel; Hevia, Eduardo | | Wrong Outcome |
| #1659 | Ovadia 2011 | The Contribution of an Electronic Conductivity Device to the Safety of Pedicle Screw Insertion in Scoliosis Surgery. | Ovadia, Dror; Korn, Akiva; Fishkin, Michael; Steinberg, David; Wientroub, Shlomo; Ofiram, Elisha | | Wrong Outcome |

For Peer Review

| | | | | | |
|---|---|---|---|---|---|
| #1698 | Parker 2011 | Accuracy of Free-Hand Pedicle Screws in the Thoracic and Lumbar Spine: Analysis of 6816 Consecutive Screws. | Parker, Scott; McGirt, Matthew; Farber, S; Amin, Anubhav; Rick, Anne-Marie; Suk, Ian; Bydon, Ali; Sciubba, Daniel; Wolinsky, Jean-Paul; Gokaslan, Ziya; Witham, Timothy | | Wrong Intervention |
| #1742 | Modi 2010 | Accuracy of Thoracic Pedicle Screw Using Ideal Pedicle Entry Point in Severe Scoliosis. | Modi, Hitesh; MS, PhD; Suh, Seung-Woo; MD, PhD; 1, a; Hong, Jae-Young; Yang, Jae-Hyuk | | Wrong Outcome |
| #1754 | Ayhan 2010 | Transthoracic Surgical Treatment for Centrally Located Thoracic Disc Herniations Presenting With Myelopathy: A 5-year Institutional Experience. | Ayhan, Selim; Nelson, Clarke; Gok, Beril; Petteys, Rory; Wolinsky, Jean-Paul; Witham, Timothy; Bydon, Ali; Gokaslan, Ziya; Sciubba, Daniel | | Wrong Outcome |
| #1767 | Takahashi 2010 | Accuracy of Multilevel Registration in Image-Guided Pedicle Screw Insertion for Adolescent Idiopathic Scoliosis. | Takahashi, Jun; Hirabayashi, Hiroki; Hashidate, Hiroyuki; Ogihara, Nobuhide; Kato, Hiroyuki | | Wrong Outcome |

For Peer Review

| | | | | | |
|---|---|---|---|---|---|
| #1771 | Duffy 2010 | Usefulness of Electromyography Compared to Computed Tomography Scans in Pedicle Screw Placement. | Duffy, Michael; Phillips, Jonathan; Knapp, Dennis; Herrera-Soto, Jose | | Wrong Outcome |
| #1786 | Castellon 2009 | Intraoperative Neurophysiologic Spinal Cord Monitoring in Thoracolumbar Burst Fractures. | Castellon, Alfredo; Meves, Robert; Avanzi, Osmar | | Wrong Outcome |
| #1791 | Hart 2009 | Intraoperative Neuromonitoring in Pediatric Spinal Deformity Surgery. | Hart, Erin; Grottkau, Brian | | Wrong Study Design |
| #1800 | Xu 2009 | Sensitivity and efficacy of intraoperative electromyography monitoring in combination with 3D imaging modality during complex lumbosacral procedures. | Xu, Feng; Yu, Xing; Bi, Lian-yong; Gao, Fei; Ke, Hai; Xu, Lin | Ne: Symbol. | Full Text Not in English |

For Peer Review

| | | | | | |
|---|---|---|---|---|---|
| #1819 | | Motor and Somatosensory Evoked Potentials Are Well Maintained in Patients Given Dexmedetomidine During Spine Surgery. | | | Wrong Outcome |
| #1850 | Dimopoulos 2009 | Quantitative Estimation of the Recurrent Laryngeal Nerve Irritation by Employing Spontaneous Intraoperative Electromyographic Monitoring During Anterior Cervical Discectomy and Fusion. | Dimopoulos, Vasilios; Chung, Induk; Lee, Gregory; Johnston, Kim; MD, FACS; Kapsalakis, Ioannis; Smisson, Hugh; III MD, FACS; Grigorian, Arthur; MD, PhD; Robinson, Joe; Jr MD, FACS; Fountas, Kostas | | Wrong Intervention |
| #1869 | TOBIAS 2008 | Effects of dexmedetomidine on intraoperative motor and somatosensory evoked potential monitoring during spinal surgery in adolescents. | TOBIAS, JOSEPH; GOBLE, TIMOTHY; BATES, GUY; ANDERSON, JOHN; HOERNSCHEMEYER, DANIEL | | Wrong Outcome |
| #1882 | Bala 2008 | Motor and Somatosensory Evoked Potentials Are Well Maintained in Patients Given Dexmedetomidine during Spine Surgery. | Bala, Endrit; Sessler, Daniel; Nair, Dileep; McLain, Robert; Dalton, Jarrod; Farag, Ehab | | Wrong Outcome |

For Peer Review

| | | | | | |
|---|---|---|---|---|---|
| #1901 | Donohue 2008 | Pulse-Train Stimulation for Detecting Medial Malpositioning of Thoracic Pedicle Screws. | Donohue, Miriam; Murtagh-Schaffer, Catherine; Basta, John; Moquin, Ross; Bashir, Asif; Calancie, Blair | | Wrong Outcome |
| #1906 | Sakai 2008 | Segmental Pedicle Screwing for Idiopathic Scoliosis Using Computer-assisted Surgery. | Sakai, Yoshihito; Matsuyama, Yukihiro; Nakamura, Hiroshi; Katayama, Yoshito; Imagama, Shiro; Ito, Zenya; Ishiguro, Naoki | | Wrong Outcome |
| #1925 | Anschel 2008 | Successful Intraoperative Spinal Cord Monitoring During Scoliosis Surgery Using a Total Intravenous Anesthetic Regimen Including Dexmedetomidine. | Anschel, David; Aherne, Andrew; Soto, Roy; Carrion, Wesley; Hoegerl, Carl; Nori, Palgun; Seidman, Peggy | | Wrong Outcome |
| #1937 | Lehman 2007 | Computed Tomography Evaluation of Pedicle Screws Placed in the Pediatric Deformed Spine Over an 8-Year Period. | Lehman, Ronald; Lenke, Lawrence; Keeler, Kathryn; Kim, Yongjung; Cheh, Gene | | Wrong Outcome |

For Peer Review

| | | | | | |
|---|---|---|---|---|---|
| #1970 | | The Use of Somatosensory Evoked Potentials to Determine the Relationship Between Patient Positioning and Impending Upper Extremity Nerve Injury During Spine Surgery: A Retrospective Analysis. | | | Wrong Outcome |
| #1995 | Tanaka 2006 | Postoperative Segmental C5 Palsy After Cervical Laminoplasty May Occur Without Intraoperative Nerve Injury: A Prospective Study With Transcranial Electric Motor-Evoked Potentials. | Tanaka, Nobuhiro; MD, PhD; Nakanishi, Kazuyoshi; Fujiwara, Yasushi; Kamei, Naosuke; Ochi, Mitsuo | | Wrong Outcome |
| #2027 | Sala 2006 | Motor Evoked Potential Monitoring Improves Outcome after Surgery for Intramedullary Spinal Cord Tumors: A Historical Control Study. | Sala, Francesco; Palandri, Giorgio; Basso, Elisabetta; Lanteri, Paola; Deletis, Vedran; Faccioli, Franco; Bricolo, Albino | | Wrong Outcome |

For Peer Review

| | | | | | |
|---|---|---|---|---|---|
| #2072 | Smorgick 2005 | Accuracy and Safety of Thoracic Pedicle Screw Placement in Spinal Deformities. | Smorgick, Yossi; Millgram, Michael; Anekstein, Yoram; Floman, Yizhar; Mirovsky, Yigal | | Wrong Outcome |
| #2085 | Thuet 2005 | Increased Risk of Postoperative Neurologic Deficit for Spinal Surgery Patients With Unobtainable Intraoperative Evoked Potential Data. | Thuet, Earl; Padberg, Anne; Raynor, Barry; Bridwell, Keith; Riew, K; Taylor, Brett; Lenke, Lawrence | | Wrong Outcome |
| #2127 | Liu 2005 | Effects of isoflurane and propofol on cortical somatosensory evoked potentials during comparable depth of anaesthesia as guided by bispectral index. | Liu, E.; Wong, H.; Chia, C.; Lim, H.; Chen, Z.; Lee, T. | | Wrong Patient Population |
| #2186 | Kim 2004 | Free Hand Pedicle Screw Placement in the Thoracic Spine: Is it Safe? | Kim, Yongjung; Lenke, Lawrence; Bridwell, Keith; Cho, Yongsun; Riew, K. | | Wrong Outcome |

For Peer Review

| | | | | | |
|---|---|---|---|---|---|
| #2203 | Rohde 2003 | Measurement of motor evoked potentials following repetitive magnetic motor cortex stimulation during isoflurane or propofol anaesthesia. | Rohde, V.; Krombach, G.; Baumert, J.; Kreitschmann-Andermahr, I.; Weinzierl, M.; Gilsbach, J. | | Wrong Intervention |
| #2225 | Shi 2003 | Electrical Stimulation for Intraoperative Evaluation of Thoracic Pedicle Screw Placement. | Shi, Yong-bing; MD, PhD; Binette, Michael; Martin, William; Pearson, James; Hart, Robert | | Wrong Outcome |
| #2236 | Aglio 2002 | The Use of Transcranial Magnetic Stimulation for Monitoring Descending Spinal Cord Motor Function. | Aglio, Linda; Romero, Rafael; Desai, Sukumar; Ramirez, Marcela; Gonzalez, Andres; Gugino, Laverne; MD, PhD | | Wrong Outcome |
| #2246 | Inoue 2002 | Intraoperative Monitoring of Myogenic Motor-Evoked Potentials From the External Anal Sphincter Muscle to Transcranial Electrical Stimulation. | Inoue, Satoki; Kawaguchi, Masahiko; Takashi, Shimoda; Kakimoto, Meiko; Sakamoto, Takanori; Kitaguchi, Katsuyasu; Furuya, Hitoshi; Morimoto, Tetsuya; Sakaki, Toshisuke | | Wrong Outcome |

For Peer Review

| | | | | | |
|---|---|---|---|---|---|
| #2269 | Ku 2002 | Effect of sevoflurane/nitrous oxide versus propofol anaesthesia on somatosensory evoked potential monitoring of the spinal cord during surgery to correct scoliosis. | Ku, A.; Hu, Y.; Irwin, M.; Chow, B.; Gunawardene, S.; Tan, E.; Luk, K. | | Wrong Outcome |
| #2291 | Reidy 2001 | Evaluation of electromyographic monitoring during insertion of thoracic pedicle screws. | Reidy, D.; Houlden, D.; Nolan, P.; Kim, M.; Finkelstein, J. | | Wrong Outcome |
| #2328 | Wilson-Holden 2000 | A Prospective Comparison of Neurogenic Mixed Evoked Potential Stimulation Methods: Utility of Epidural Elicitation During Posterior Spinal Surgery. | Wilson-Holden, Tracy; Padberg, Anne; Parkinson, Jeffery; Bridwell, Keith; Lenke, Lawrence; Bassett, George | | Wrong Intervention |
| #2334 | Toleikis 2000 | The Usefulness of Electrical Stimulation for Assessing Pedicle Screw Placements. | Toleikis, J.; Skelly, Jon; Carlvin, Arnold; Toleikis, Sandra; Bernard, Thomas; Burkus, J.; Burr, Max; Dorchak, John; Goldman, Marc; Walsh, Thomas | | Wrong Outcome |

For Peer Review

| | | | | | |
|---|---|---|---|---|---|
| #2360 | Owen 1999 | The Application of Intraoperative Monitoring During Surgery for Spinal Deformity. | Owen, Jeffrey | | Wrong Study Design |
| #2385 | Laureau 1999 | Comparative Study of Propofol and Midazolam Effects on Somatosensory Evoked Potentials during Surgical Treatment of Scoliosis. | Laureau, Emmanuele; Marciniak, Bruno; Hebrard, Anne; Herbaux, Bernard; Guieu, Jean | | Wrong Outcome |
| #2394 | Ubags 1999 | A Comparison of Myogenic Motor Evoked Responses to Electrical and Magnetic Transcranial Stimulation During Nitrous Oxide/Opioid Anesthesia. | Ubags, Leon; MD, PhD; Kalkman, Cor; Been, Henk; Koelman, Johannis; de Visser, Bram; Ongerboer MD, PhD | | Wrong Outcome |
| #2418 | Balzer 1998 | Simultaneous Somatosensory Evoked Potential and Electromyographic Recordings during Lumbosacral Decompression and Instrumentation. | Balzer, Jeffrey; Rose, Robert; Welch, William; Sclabassi, Robert; MD, PhD | | Wrong Outcome |

For Peer Review

| | | | | | |
|---|---|---|---|---|---|
| #2426 | Cheng 1998 | Posterior Tibial Nerve Somatosensory Cortical Evoked Potentials in Adolescent Idiopathic Scoliosis. | Cheng, Jack; MBBS, FRCS; FACS, FHKAM; Guo, Xia; Sher, Andy | | Wrong Outcome |
| #2428 | Darden 1998 | A Comparison of Impedance and Electromyogram Measurements in Detecting the Presence of Pedicle Wall Breakthrough. | Darden, Bruce; Owen, Jeffrey; Hatley, Martha; Kostuik, John; Tooke, S. | | Wrong Outcome |
| #2431 | Vedran 1997 | The Role of Motor Evoked Potentials during Surgery for Intramedullary Spinal Cord Tumors. | Vedran MD, PhD | | Wrong Outcome |
| #2449 | Schwarzenbach 1997 | Accuracy of Computer-Assisted Pedicle Screw Placement: An In Vivo Computed Tomography Analysis. | Schwarzenbach, Othmar; Berlemann, Ulrich; Jost, Bernhard; Visarius, Heiko; Arm, Erich; Langlotz, Frank; Nolte, Lutz-P.; Ozdoba, Christoph | | Wrong Intervention |

For Peer Review

| | | | | | |
|---|---|---|---|---|---|
| #2460 | Bernard 1996 | Effects of Isoflurane and Desflurane on Neurogenic Motor- and Somatosensory-evoked Potential Monitoring for Scoliosis Surgery. | Bernard, Jean-Marc; MD, PhD; Pereon, Yann; Fayet, Guillemette; Guiheneuc, Pierre | | Wrong Outcome |
| #2468 | Pechstein 1996 | Transcranial High-frequency Repetitive Electrical Stimulation for Recording Myogenic Motor Evoked Potentials with the Patient under General Anesthesia. | Pechstein, Ulrich; Cedzich, Cornelia; Nadstawek, Joachim; Schramm, Johannes | | Wrong Outcome |
| #2475 | Woodforth 1996 | Variability of Motor-Evoked Potentials Recorded During Nitrous Oxide Anesthesia from the Tibialis Anterior Muscle After Transcranial Electrical Stimulation. | Woodforth, Ian; MB, BS; Hicks, Richard; Crawford, Matthew; Stephen, John; Burke, David; MD, DSc | | Wrong Outcome |
| #2483 | Dennis 1996 | Monitoring of Median Nerve Somatosensory Evoked Potentials During Cervical Spinal Cord Decompression. | Dennis, G.; Dehkordi, O.; Millis, R.; Cole, A.; Brown, D.; Paul, O. | | Wrong Outcome |

For Peer Review

| | | | | | |
|---|---|---|---|---|---|
| #2491 | Lee 1995 | Intraoperative Monitoring of Motor Function by Magnetic Motor Evoked Potentials. | Lee, Wei-Yuh; Hou, Wen-Yeong; Yang, Lin-Huse; Lin, Swei-Ming | | Wrong Intervention |
| #2496 | Koyanagi 1993 | Spinal Cord Evoked Potential Monitoring after Spinal Cord Stimulation during Surgery of Spinal Cord Tumors. | Koyanagi, Izumi; Iwasaki, Yoshinobu; Isu, Toyohiko; Abe, Hiroshi; Akino, Minoru; Kuroda, Satoshi | | Wrong Intervention |
| #2498 | Rappaport 1992 | Effects of Anesthesia and Stimulus Intensity on Posterior Tibial Nerve Somatosensory Evoked Potentials. | Rappaport, Maurice; Leonard, Jill; Portillo, Sarbelio | | Wrong Outcome |
| #2504 | DVORAK 1990 | Motor-Evoked Potentials in Patients with Cervical Spine Disorders. | DVORAK, JIRI; HERDMANN, JORG; JANSSEN, BEATRICE; THEILER, ROBERT; GROB, DIETER | | Wrong Intervention |
| #2517 | Min 2018 | Accuracy of thoracic pedicle screw placement using freehand technique and triggered EMG in adolescent idiopathic scoliosis: Is it different between concave and convex side?. | Min, Woo-Kie; Na, Sang-Bong; Jang, Jin-An | Journal of orthopaedic surgery (Hong Kong) | Wrong Outcome |

For Peer Review

| | | | | | |
|---|---|---|---|---|---|
| #2523 | Iacopino 2017 | EMG-Guided Percutaneous Placement of Cement-Augmented Pedicle Screws for Osteoporotic Thoracolumbar Burst Fractures. | Iacopino, Domenico Gerardo; Certo, Francesco; Graziano, Francesca; Basile, Luigi; Guli, Carlo; Visocchi, Massimiliano; Conti, Alfredo; Maugeri, Rosario | Acta neurochirurgica. Supplement | Wrong Outcome |
| #2529 | Kumar 2014 | Elevated preoperative blood pressure predicts the intraoperative loss of SSEP neuromonitoring signals during spinal surgery. | Kumar, Akash; Chen, Yuangen; Lin, Hung-Mo; Deiner, Stacie | Journal of clinical monitoring and computing | Wrong Outcome |
| #2532 | Holdefer 2013 | Utility of evoked EMG monitoring to improve bone screw placements in the cervical spine. | Holdefer, Robert N; Heffez, Daniel S; Cohen, Bernard A | Journal of spinal disorders & techniques | Wrong Outcome |
| #2538 | Regidor 2011 | Recording triggered EMG thresholds from axillary chest wall electrodes: a new refined technique for accurate upper thoracic (T2-T6) pedicle screw placement. | Regidor, Ignacio; de Blas, Gema; Barrios, Carlos; Burgos, Jesus; Montes, Elena; Garcia-Urquiza, Sergio; Hevia, Edurado | European spine journal : official publication of the European Spine Society, the European Spinal Deformity Society, and the European Section of the Cervical Spine Research Society | Wrong Outcome |

For Peer Review

| | | | | | |
|---|---|---|---|---|---|
| #2540 | Samdani 2011 | Triggered electromyography for placement of thoracic pedicle screws: is it reliable?. | Samdani, Amer F; Tantorski, Mark; Cahill, Patrick J; Ranade, Ashish; Koch, Stephen; Clements, David H; Betz, Randal R; Asghar, Jahangir | European spine journal : official publication of the European Spine Society, the European Spinal Deformity Society, and the European Section of the Cervical Spine Research Society | Wrong Outcome |
| #2543 | Drake 2010 | Intraoperative neurophysiological monitoring during complex spinal deformity cases in pediatric patients: methodology, utility, prognostication, and outcome. | Drake, James; Zeller, Reinhard; Kulkarni, Abhaya V; Strantzas, Samuel; Holmes, Laura | Child's nervous system : ChNS : official journal of the International Society for Pediatric Neurosurgery | Wrong Outcome |
| #2549 | Zaarour 2007 | Effect of low-dose ketamine on voltage requirement for transcranial electrical motor evoked potentials in children. | Zaarour, Christian; Engelhardt, Thomas; Strantzas, Samuel; Pehora, Carolyne; Lewis, Stephen; Crawford, Mark W | Spine | Wrong Outcome |
| #2551 | Sutter 2007 | Multimodal intraoperative monitoring: an overview and proposal of methodology based on 1,017 cases. | Sutter, Martin; Eggspuehler, Andreas; Muller, Alfred; Dvorak, Jiri | European spine journal : official publication of the European Spine Society, the European Spinal Deformity Society, and the European | Wrong Outcome |

For Peer Review

| | | | | | |
|---|---|---|---|---|---|
| | | | | Section of the Cervical Spine Research Society | |
| #2558 | Bose 2004 | Neurophysiological monitoring of spinal cord function during instrumented anterior cervical fusion. | Bose, Bikash; Sestokas, Anthony K; Schwartz, Daniel M | The spine journal : official journal of the North American Spine Society | Wrong Outcome |
| #2564 | Cioni 1999 | Intraoperative motor evoked potentials monitoring in spinal neurosurgery. | Cioni, B; Meglio, M; Rossi, G F | Archives italiennes de biologie | Wrong Study Design |
| #2569 | Maguire 1995 | Evaluation of intrapedicular screw position using intraoperative evoked electromyography. | Maguire, J; Wallace, S; Madiga, R; Leppanen, R; Draper, V | Spine | Wrong Outcome |
| #2570 | Erwin 1993 | Up and down the spinal cord: intraoperative monitoring of sensory and motor spinal cord pathways. | Erwin, C W; Erwin, A C | Journal of clinical neurophysiology : official publication of the American Electroencephalographic Society | Wrong Study Design |
| #285 | Agarwal 2017 | Intraoperative Neurophysiologic Monitoring | Agarwal N.; Hamilton D.K.; Ozpinar A.; Choi P.; Hart R.; Yaylali I. | World Neurosurgery | No 2x2 |

For Peer Review

| | | | | | |
|---|---|---|---|---|---|
| | | for Adult Patients Undergoing Posterior Spinal Fusion | | | |
| #169 | Barsotti 2020 | Diagnostic accuracy of perioperative electromyography in the positioning of pedicle screws in adolescent idiopathic scoliosis treatment: A cross-sectional diagnostic study | Barsotti C.E.; Gavassi B.M.; Prado F.E.; Batista B.N.; De Resende Pratali R.; Ribeiro A.P.; De Oliveira C.E.S.; Ferreira R.R. | BMC Musculoskeletal Disorders | No 2x2 |
| #1904 | Cheh 2008 | Loss of Spinal Cord Monitoring Signals in Children During Thoracic Kyphosis Correction With Spinal Osteotomy: Why Does It Occur and What Should You Do? | Cheh, Gene; Lenke, Lawrence; Padberg, Anne; MS, CCC-A; Kim, Yongjung; Daubs, Michael; MD, FACS; Kuhns, Craig; Stobbs, Georgia; Hensley, Marsha | | Wrong Outcome |
| #41 | Chen 2013 | Comparison of the wake-up test and combined TES-MEP and CSEP monitoring in spinal surgery | Chen B.; Chen Y.; Yang J.; Xie D.; Su H.; Li F.; Wan Y.; Peng X.; Zheng Z. | Journal of Spinal Disorders and Techniques | Wrong Outcome |

For Peer Review

| | | | | | |
|---|---|---|---|---|---|
| #756 | Chen 2022 | Intraoperative Neuromonitoring Auxiliary Significance of DNEP for MEP-positive Event During Severe Spinal Deformity Surgery. | Chen, Jian; Deng, Yao-long; Sui, Wen-yuan; Yang, Jing-fan; MD, PhD; Xu, Jing; Huang, Zi-fang; Yang, Jun-lin | | No 2x2 |
| #168 | Cousino 2020 | Anterolateral S1 screw malposition detected with intraoperative neurophysiological monitoring during posterior lumbosacral fusion | Cousino J.P.C.; Luna F.; Torche M.; Vigueras S.; Torche E.; Valdes G. | Surgical Neurology International | No 2x2 |
| #828 | Krzeptowsky 2021 | Neurophysiological Intraoperative Monitoring in the Elderly. | De la Maza Krzeptowsky, Lilia; San-Juan, Daniel; Ximenez Camilli, Cecilia; Alvarez Perera, Luis; Valdez Ruvalcaba, Hector; Morales Baez, Jorge; Anschel, David | | No 2x2 |

For Peer Review

| | | | | | |
|---|---|---|---|---|---|
| #1676 | Hamilton 2011 | Rates of New Neurological Deficit Associated With Spine Surgery Based on 108,419 Procedures: A Report of the Scoliosis Research Society Morbidity and Mortality Committee. | Hamilton, D.; Smith, Justin; MD, PhD; Sansur, Charles; MD, MHSc; Glassman, Steven; Ames, Christopher; Berven, Sigurd; Polly, David; Perra, Joseph; Knapp, Dennis; Boachie-Adjei, Oheneba; McCarthy, Richard; Shaffrey, Christopher | | No 2x2 |
| #2234 | Hu 2003 | Application of time-frequency analysis to somatosensory evoked potential for intraoperative spinal cord monitoring. | Hu, Y; Luk, K; Lu, W; Leong, J | | Wrong Outcome |
| #144 | Kobayashi 2021 | Effects of Preoperative Motor Status on Intraoperative Motor-evoked Potential Monitoring for High-risk Spinal Surgery: A Prospective Multicenter Study | Kobayashi K.; Imagama S.; Yoshida G.; Ando M.; Kawabata S.; Yamada K.; Kanchiku T.; Fujiwara Y.; Taniguchi S.; Iwasaki H.; Tadokoro N.; Takahashi M.; Wada K.; Yamamoto N.; Shigematsu H.; Funaba M.; Yasuda A.; Kobayashi S.; | Spine | Wrong Outcome |

For Peer Review

| | | | | | |
|---|---|---|---|---|---|
| | | | Ushirozako H.; Tani T.; Matsuyama Y. | | |
| #234 | Lakomkin 2018 | Utility of Intraoperative Monitoring in the Resection of Spinal Cord Tumors | Lakomkin N.; Mistry A.M.; Zuckerman S.L.; Ladner T.; Kothari P.; Lee N.J.; Stannard B.; Vasquez R.A.; Cheng J.S. | Spine | Wrong Intervention |
| #201 | Lieberman 2019 | The reliability of motor evoked potentials to predict dorsiflexion injuries during lumbosacral deformity surgery: importance of multiple myotomal monitoring | Lieberman J.A.; Lyon R.; Jasiukaitis P.; Berven S.H.; Burch S.; Feiner J. | Spine Journal | Wrong Outcome |
| #292 | Morishige 2017 | Application of compound action potential of facial muscles evoked by transcranial stimulation as a reference waveform of motor-evoked potential in spinal surgery | Morishige M.; Takeda M.; Yamaguchf S.; Sugiyama K.; Kurisu K. | Hiroshima Journal of Medical Sciences | Wrong Intervention |

For Peer Review

| | | | | | |
|---|---|---|---|---|---|
| #2388 | Norcross-Nechay 1999 | Intraoperative Somatosensory Evoked Potential Findings in Acute and Chronic Spinal Canal Compromise. | Norcross-Nechay, Karyl; MD, PhD; Mathew, Titus; Simmons, James; Hadjipavlou, Alexander | | No 2x2 |
| #2537 | Parker 2011 | Ability of electromyographic monitoring to determine the presence of malpositioned pedicle screws in the lumbosacral spine: analysis of 2450 consecutively placed screws. | Parker, Scott L; Amin, Anubhav G; Farber, S Harrison; McGirt, Matthew J; Sciubba, Daniel M; Wolinsky, Jean-Paul; Bydon, Ali; Gokaslan, Ziya L; Witham, Timothy F | Journal of neurosurgery. Spine | Wrong Outcome |
| #543 | Raynor 2007 | Correlation between low triggered electromyographic thresholds and lumbar pedicle screw malposition: Analysis of 4857 screws | Raynor B.L.; Lenke L.G.; Bridwell K.H.; Taylor B.A.; Padberg A.M. | Spine | Wrong Outcome |
| #207 | Shao 2019 | The efficacy of intraoperative multimodal monitoring in pedicle subtraction osteotomies of the lumbar spine | Shao J.; Lee M.Y.; Louis S.; Knusel K.; Lee B.S.; Pelle D.W.; Savage J.; Tanenbaum J.E.; Mroz T.E.; Steinmetz M.P. | Journal of Neurosurgery: Spine | No 2x2 |

For Peer Review

| | | | | | |
|---|---|---|---|---|---|
| #2506 | Szalay 1986 | Sensitivity of Spinal Cord Monitoring to Intraoperative Events. | Szalay, Elizabeth; Carollo, James; Roach, James | | No Full Text |
| #231 | Tamkus 2018 | Transcranial Motor Evoked Potential Alarm Criteria to Predict Foot Drop Injury during Lumbosacral Surgery | Tamkus A.; Rice K.S.; Hoffman G. | Spine | No 2x2 |
| #1023 | Tanaka 2019 | Cutoff Points, Sensitivities, and Specificities of Intraoperative Motor-Evoked Potential Monitoring Determined Using Receiver Operating Characteristic Analysis. | Tanaka, Satoshi; Akimoto, Jiro; Hashimoto, Ryo; Takanashi, Junko; Oka, Hidehiro | | No 2x2 |
| #25 | Tani 2022 | Threshold-based Monitoring of Compound Muscle Action Potentials for Percutaneous Pedicle Screw Placement in the Lumbosacral Spine: Can We Rely on Stimulation of the Uninsulated Screw to Provide a Valid Safety Warning? | Tani Y.; Saito T.; Taniguchi S.; Ishihara M.; Paku M.; Adachi T.; Ando M.; Kohara N.; Kimura J. | Spine | Wrong Outcome |

For Peer Review

| | | | | | |
|---|---|---|---|---|---|
| #268 | Tobert 2017 | Efficacy of Intraoperative Neurophysiologic Monitoring for Pediatric Cervical Spine Surgery | Tobert D.G.; Glotzbecker M.P.; Hresko M.T.; Karlin L.I.; Proctor M.R.; Emans J.B.; Miller P.E.; Hedequist D.J. | Spine | Paediatric population |
| #387 | Ukegawa 2014 | Efficacy of biphasic transcranial electric stimulation in intraoperative motor evoked potential monitoring for cervical compression myelopathy | Ukegawa D.; Kawabata S.; Sakaki K.; Ishii S.; Tomizawa S.; Inose H.; Yoshii T.; Kato T.; Enomoto M.; Okawa A. | Spine | Wrong Outcome |
| #645 | Wilson-Holden 1999 | Efficacy of intraoperative monitoring for pediatric patients with spinal cord pathology undergoing spinal deformity surgery | Wilson-Holden T.J.; Padberg A.M.; Lenke L.G.; Larson B.J.; Bridwell K.H.; Bassett G.S. | Spine | No 2x2 |
| #678 | Yang 1994 | Intraoperative transcranial electrical motor evoked potential monitoring during spinal surgery under intravenous ketamine or etomidate anaesthesia | Yang L.-H.; Lin S.-M.; Lee W.-Y.; Liu C.-C. | Acta Neurochirurgica | Wrong Outcome |

For Peer Review

| | | | | | |
|---|---|---|---|---|---|
| #187 | Yoshida 2019 | Adverse Events Related to Transcranial Electric Stimulation for Motor-evoked Potential Monitoring in High-risk Spinal Surgery | Yoshida G.; Imagama S.; Kawabata S.; Yamada K.; Kanchiku T.; Fujiwara Y.; Tadokoro N.; Takahashi M.; Wada K.; Yamamoto N.; Ushirozako H.; Kobayashi K.; Yasuda A.; Ando M.; Tani T.; Matsuyama Y. | Spine | Wrong Outcome |
| #313 | Raynor 2016 | Failure of Intraoperative Monitoring to Detect Postoperative Neurologic Deficits: A 25-year Experience in 12,375 Spinal Surgeries | Raynor B.L.; Padberg A.M.; Lenke L.G.; Bridwell K.H.; Riew K.D.; Buchowski J.M.; Luhmann S.J. | Spine | No 2x2 |
| #345 | Murcow 2013 | pinal Cord Monitoring With Transcranial Motor Evoked Potentials in Patients With Neural Axis Abnormalities Undergoing Spinal Deformity Surgery | Ryan D. Muchow, MDa,*, Anna McClung, BSN, RNb, Patricia Rampy, MS, R EP T, CNIMb, Elizabeth Van Allen, MS, R EP T, CNIMb, Steven Sparagana, MDb, Daniel J. Sucato, MD, MS | Spine Deformity | No/ Incomplete 2x2 |

For Peer Review

**Supplemental Figure 1a. Forest plot for Sensitivity for SSEP by Threshold Type**

| Study | Events | Total | Proportion | 95%-CI |
|---|---|---|---|---|
| **Threshold = 50%** | | | | |
| Abdelkader 2019 | 18 | 20 | 0.900 | [0.683; 0.988] |
| Bhagat 2015 | 13 | 23 | 0.565 | [0.345; 0.768] |
| Chandanwale 2008 | 15 | 17 | 0.882 | [0.636; 0.985] |
| Chen Yu 2021 | 2 | 7 | 0.286 | [0.037; 0.710] |
| Chung 2009 | 9 | 9 | 1.000 | [0.664; 1.000] |
| Deutsch 2000 | 0 | 4 | 0.000 | [0.000; 0.602] |
| Ding 2008 | 3 | 4 | 0.750 | [0.194; 0.994] |
| Duncan 2012 | 5 | 5 | 1.000 | [0.478; 1.000] |
| Feyissa 2015 | 0 | 2 | 0.000 | [0.000; 0.842] |
| Forster 2012 | 14 | 15 | 0.933 | [0.681; 0.998] |
| Gundanna 2003 | 0 | 5 | 0.000 | [0.000; 0.522] |
| Hu_AmplitudeLatency 2011 | 2 | 2 | 1.000 | [0.158; 1.000] |
| Hu_TFA 2011 | 2 | 2 | 1.000 | [0.158; 1.000] |
| Huang_>50% 2016 | 4 | 4 | 1.000 | [0.398; 1.000] |
| Kelleher 2008 | 26 | 35 | 0.743 | [0.567; 0.875] |
| Khan 2006 | 27 | 35 | 0.771 | [0.599; 0.896] |
| Kim 2021 | 0 | 5 | 0.000 | [0.000; 0.522] |
| Kim 2019 | 0 | 7 | 0.000 | [0.000; 0.410] |
| Krishnakumar 2011 | 2 | 2 | 1.000 | [0.158; 1.000] |
| Leung 2005 | 5 | 5 | 1.000 | [0.478; 1.000] |
| Li_PermanentChanges 2018 | 4 | 9 | 0.444 | [0.137; 0.788] |
| Li_TemporaryChanges 2018 | 1 | 6 | 0.167 | [0.004; 0.641] |
| Loder 1991 | 28 | 28 | 1.000 | [0.877; 1.000] |
| Luk_CSEP_Cv2 2001 | 0 | 1 | 0.000 | [0.000; 0.975] |
| Luk_CSEP_Cz 2001 | 0 | 1 | 0.000 | [0.000; 0.975] |
| Luk_SCEP 2001 | 1 | 1 | 1.000 | [0.025; 1.000] |
| Luk_SSEP 2001 | 0 | 1 | 0.000 | [0.000; 0.975] |
| Makarov 2012 | 53 | 53 | 1.000 | [0.933; 1.000] |
| May, 2006 | 9 | 10 | 0.900 | [0.555; 0.997] |
| Melachuri 2020 | 11 | 57 | 0.193 | [0.100; 0.319] |
| Melachuri 2019 | 1 | 36 | 0.028 | [0.001; 0.145] |
| Melachuri 2017 | 3 | 15 | 0.200 | [0.043; 0.481] |
| Neira 2016 | 7 | 15 | 0.467 | [0.213; 0.734] |
| Noordeen_>50% 1997 | 34 | 39 | 0.872 | [0.726; 0.957] |
| Papastefanou 2000 | 10 | 10 | 1.000 | [0.692; 1.000] |
| Paradiso 2006 | 1 | 2 | 0.500 | [0.013; 0.987] |
| Park 2011 | 1 | 4 | 0.250 | [0.006; 0.806] |
| Park_50% 2018 | 9 | 38 | 0.237 | [0.114; 0.402] |
| Qiu 2022 | 3 | 3 | 1.000 | [0.292; 1.000] |
| Randall 1991 | 28 | 28 | 1.000 | [0.877; 1.000] |
| Smith 2006 | 1 | 1 | 1.000 | [0.025; 1.000] |
| Stechison 1995 | 4 | 4 | 1.000 | [0.398; 1.000] |
| Thirumala 2014 | 19 | 20 | 0.950 | [0.751; 0.999] |
| Tohmeh 2022 | 28 | 28 | 1.000 | [0.877; 1.000] |
| Tsirikos_50% 2004 | 2 | 3 | 0.667 | [0.094; 0.992] |
| Tsirikos_Cervical 2020 | 2 | 11 | 0.182 | [0.023; 0.518] |
| Tsirikos_Cortical 2020 | 2 | 11 | 0.182 | [0.023; 0.518] |
| Wilent 2020 | 2 | 7 | 0.286 | [0.037; 0.710] |
| Xu 2011 | 1 | 3 | 0.333 | [0.008; 0.906] |
| **Common effect model** | | **653** | **0.631** | **[0.593; 0.667]** |
| **Random effects model** | | | **0.716** | **[0.495; 0.866]** |
| Heterogeneity: $I^2 = 62\%$, $\tau^2 = 8.4139$, $p < 0.01$ | | | | |
| **Threshold = Other** | | | | |
| Bose 2007 | 0 | 5 | 0.000 | [0.000; 0.522] |
| Dinner 1986 | 3 | 7 | 0.429 | [0.099; 0.816] |
| Izumi 1993 | 5 | 9 | 0.556 | [0.212; 0.863] |
| Koffie 2022 | 7 | 21 | 0.333 | [0.146; 0.570] |
| Manninen 1998 | 4 | 7 | 0.571 | [0.184; 0.901] |
| Meyer 1988 | 1 | 1 | 1.000 | [0.025; 1.000] |
| Yang 1994 | 1 | 1 | 1.000 | [0.025; 1.000] |
| **Common effect model** | | **51** | **0.412** | **[0.286; 0.550]** |
| **Random effects model** | | | **0.412** | **[0.286; 0.550]** |
| Heterogeneity: $I^2 = 0\%$, $\tau^2 = 0$, $p = 0.93$ | | | | |
| **Threshold = 60%** | | | | |
| Hilibrand 2006 | 3 | 12 | 0.250 | [0.055; 0.572] |
| Huang_>60% 2016 | 4 | 4 | 1.000 | [0.398; 1.000] |
| Padberg 1996 | 1 | 1 | 1.000 | [0.025; 1.000] |
| Tsirikos_60% 2004 | 1 | 3 | 0.333 | [0.008; 0.906] |
| **Common effect model** | | **20** | **0.450** | **[0.253; 0.664]** |
| **Random effects model** | | | **0.629** | **[0.129; 0.951]** |
| Heterogeneity: $I^2 = 0\%$, $\tau^2 = 2.5549$, $p = 0.99$ | | | | |
| **Threshold = 25%** | | | | |
| Noordeen_>25% 1997 | 34 | 37 | 0.919 | [0.781; 0.983] |
| Tsirikos_25% 2004 | 2 | 3 | 0.667 | [0.094; 0.992] |
| **Common effect model** | | **40** | **0.900** | **[0.762; 0.962]** |
| **Random effects model** | | | **0.900** | **[0.762; 0.962]** |
| Heterogeneity: $I^2 = 38\%$, $\tau^2 = 0$, $p = 0.20$ | | | | |
| **Threshold = 75%** | | | | |
| Noordeen_>75% 1997 | 46 | 63 | 0.730 | [0.603; 0.834] |
| Tsirikos_75% 2004 | 1 | 3 | 0.333 | [0.008; 0.906] |
| **Common effect model** | | **66** | **0.712** | **[0.592; 0.808]** |
| **Random effects model** | | | **0.712** | **[0.592; 0.808]** |
| Heterogeneity: $I^2 = 45\%$, $\tau^2 = 0$, $p = 0.18$ | | | | |
| **Threshold = All or none** | | | | |
| Park_AllorNone 2018 | 9 | 37 | 0.243 | [0.118; 0.412] |
| **Common effect model** | | **867** | **0.616** | **[0.583; 0.648]** |
| **Random effects model** | | | **0.675** | **[0.509; 0.806]** |

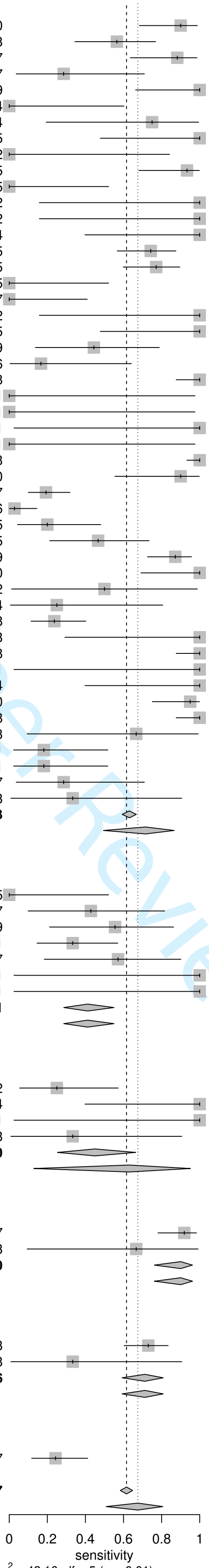


Heterogeneity: $I^2 = 62\%$, $\tau^2 = 5.9269$, $p < 0.01$
Test for subgroup differences (common effect): $\chi^2_5 = 42.16$, df = 5 ($p < 0.01$)
Test for subgroup differences (random effects): $\chi^2_5 = 38.98$, df = 5 ($p < 0.01$)

For Peer Review

**Supplemental Figure 1b. Forest plot for Specificity for SSEP by Threshold Type**



| Study | Events | Total | Proportion | 95%−CI |
|---|---|---|---|---|
| **Threshold = 50%** | | | | |
| Abdelkader 2019 | 21 | 30 | 0.700 | [0.506; 0.853] |
| Bhagat 2015 | 328 | 329 | 0.997 | [0.983; 1.000] |
| Chandanwale 2008 | 74 | 95 | 0.779 | [0.682; 0.858] |
| Chen Yu 2021 | 97 | 105 | 0.924 | [0.855; 0.967] |
| Chung 2009 | 220 | 221 | 0.995 | [0.975; 1.000] |
| Deutsch 2000 | 37 | 40 | 0.925 | [0.796; 0.984] |
| Ding 2008 | 71 | 72 | 0.986 | [0.925; 1.000] |
| Duncan 2012 | 110 | 110 | 1.000 | [0.967; 1.000] |
| Feyissa 2015 | 10 | 17 | 0.588 | [0.329; 0.816] |
| Forster 2012 | 192 | 198 | 0.970 | [0.935; 0.989] |
| Gundanna 2003 | 181 | 181 | 1.000 | [0.980; 1.000] |
| Hu_AmplitudeLatency 2011 | 183 | 189 | 0.968 | [0.932; 0.988] |
| Hu_TFA 2011 | 185 | 189 | 0.979 | [0.947; 0.994] |
| Huang_>50% 2016 | 146 | 174 | 0.839 | [0.776; 0.890] |
| Kelleher 2008 | 1004 | 1020 | 0.984 | [0.975; 0.991] |
| Khan 2006 | 473 | 473 | 1.000 | [0.992; 1.000] |
| Kim 2021 | 127 | 127 | 1.000 | [0.971; 1.000] |
| Kim 2019 | 265 | 268 | 0.989 | [0.968; 0.998] |
| Krishnakumar 2011 | 50 | 50 | 1.000 | [0.929; 1.000] |
| Leung 2005 | 854 | 866 | 0.986 | [0.976; 0.993] |
| Li_PermanentChanges 2018 | 13 | 15 | 0.867 | [0.595; 0.983] |
| Li_TemporaryChanges 2018 | 13 | 15 | 0.867 | [0.595; 0.983] |
| Loder 1991 | 37 | 51 | 0.725 | [0.583; 0.841] |
| Luk_CSEP_Cv2 2001 | 27 | 27 | 1.000 | [0.872; 1.000] |
| Luk_CSEP_Cz 2001 | 22 | 27 | 0.815 | [0.619; 0.937] |
| Luk_SCEP 2001 | 23 | 24 | 0.958 | [0.789; 0.999] |
| Luk_SSEP 2001 | 20 | 20 | 1.000 | [0.832; 1.000] |
| Makarov 2012 | 248 | 253 | 0.980 | [0.954; 0.994] |
| May, 2006 | 182 | 206 | 0.883 | [0.832; 0.924] |
| Melachuri 2020 | 686 | 714 | 0.961 | [0.944; 0.974] |
| Melachuri 2019 | 1013 | 1021 | 0.992 | [0.985; 0.997] |
| Melachuri 2017 | 1002 | 1021 | 0.981 | [0.971; 0.989] |
| Neira 2016 | 243 | 244 | 0.996 | [0.977; 1.000] |
| Noordeen_>50% 1997 | 20 | 46 | 0.435 | [0.289; 0.589] |
| Papastefanou 2000 | 349 | 409 | 0.853 | [0.815; 0.886] |
| Paradiso 2006 | 34 | 34 | 1.000 | [0.897; 1.000] |
| Park 2011 | 22 | 23 | 0.957 | [0.781; 0.999] |
| Park_50% 2018 | 33 | 34 | 0.971 | [0.847; 0.999] |
| Qiu 2022 | 71 | 73 | 0.973 | [0.905; 0.997] |
| Randall 1991 | 37 | 51 | 0.725 | [0.583; 0.841] |
| Smith 2006 | 571 | 576 | 0.991 | [0.980; 0.997] |
| Stechison 1995 | 142 | 157 | 0.904 | [0.847; 0.946] |
| Thirumala 2014 | 456 | 457 | 0.998 | [0.988; 1.000] |
| Tohmeh 2022 | 25 | 28 | 0.893 | [0.718; 0.977] |
| Tsirikos_50% 2004 | 57 | 80 | 0.713 | [0.600; 0.808] |
| Tsirikos_Cervical 2020 | 1143 | 1144 | 0.999 | [0.995; 1.000] |
| Tsirikos_Cortical 2020 | 1141 | 1144 | 0.997 | [0.992; 0.999] |
| Wilent 2020 | 2553 | 2578 | 0.990 | [0.986; 0.994] |
| Xu 2011 | 43 | 45 | 0.956 | [0.849; 0.995] |
| **Common effect model** | | **15271** | **0.973** | **[0.970; 0.975]** |
| **Random effects model** | | | **0.975** | **[0.956; 0.986]** |
| Heterogeneity: $I^2$ = 94%, $\tau^2$ = 3.5177, $p$ < 0.01 | | | | |
| **Threshold = Other** | | | | |
| Bose 2007 | 221 | 233 | 0.948 | [0.912; 0.973] |
| Dinner 1986 | 209 | 213 | 0.981 | [0.953; 0.995] |
| Izumi 1993 | 10 | 11 | 0.909 | [0.587; 0.998] |
| Koffie 2022 | 427 | 477 | 0.895 | [0.864; 0.921] |
| Manninen 1998 | 253 | 265 | 0.955 | [0.922; 0.976] |
| Meyer 1988 | 140 | 145 | 0.966 | [0.921; 0.989] |
| Yang 1994 | 9 | 9 | 1.000 | [0.664; 1.000] |
| **Common effect model** | | **1353** | **0.938** | **[0.924; 0.950]** |
| **Random effects model** | | | **0.953** | **[0.923; 0.972]** |
| Heterogeneity: $I^2$ = 73%, $\tau^2$ = 0.2627, $p$ < 0.01 | | | | |
| **Threshold = 60%** | | | | |
| Hilibrand 2006 | 415 | 415 | 1.000 | [0.991; 1.000] |
| Huang_>60% 2016 | 172 | 174 | 0.989 | [0.959; 0.999] |
| Padberg 1996 | 66 | 71 | 0.930 | [0.843; 0.977] |
| Tsirikos_60% 2004 | 65 | 70 | 0.929 | [0.841; 0.976] |
| **Common effect model** | | **730** | **0.984** | **[0.971; 0.991]** |
| **Random effects model** | | | **0.985** | **[0.905; 0.998]** |
| Heterogeneity: $I^2$ = 48%, $\tau^2$ = 3.1291, $p$ = 0.12 | | | | |
| **Threshold = 25%** | | | | |
| Noordeen_>25% 1997 | 12 | 48 | 0.250 | [0.136; 0.396] |
| Tsirikos_25% 2004 | 23 | 80 | 0.287 | [0.192; 0.400] |
| **Common effect model** | | **128** | **0.273** | **[0.203; 0.357]** |
| **Random effects model** | | | **0.273** | **[0.203; 0.357]** |
| Heterogeneity: $I^2$ = 0%, $\tau^2$ = 0, $p$ = 0.65 | | | | |
| **Threshold = 75%** | | | | |
| Noordeen_>75% 1997 | 35 | 67 | 0.522 | [0.397; 0.646] |
| Tsirikos_75% 2004 | 74 | 80 | 0.925 | [0.844; 0.972] |
| **Common effect model** | | **147** | **0.741** | **[0.665; 0.806]** |
| **Random effects model** | | | **0.784** | **[0.394; 0.953]** |
| Heterogeneity: $I^2$ = 96%, $\tau^2$ = 1.4229, $p$ < 0.01 | | | | |
| **Threshold = All or none** | | | | |
| Park_AllorNone 2018 | 34 | 35 | 0.971 | [0.851; 0.999] |
| **Common effect model** | | **17664** | **0.963** | **[0.961; 0.966]** |
| **Random effects model** | | | **0.968** | **[0.948; 0.981]** |

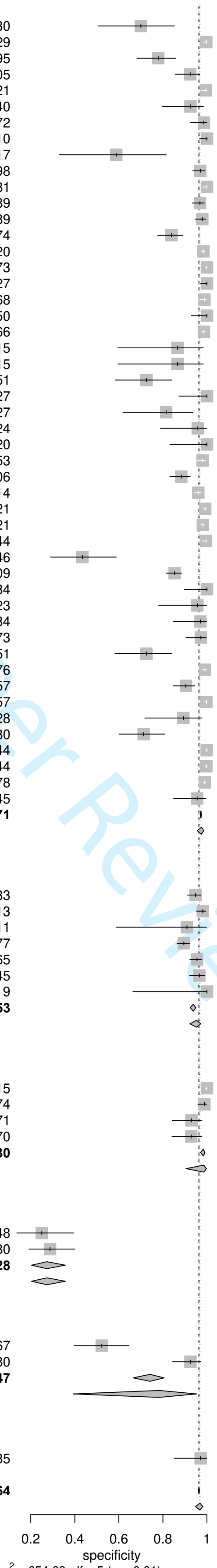


Heterogeneity: $I^2$ = 95%, $\tau^2$ = 3.8246, $p$ < 0.01
Test for subgroup differences (common effect): $\chi^2_5$ = 654.63, df = 5 ($p$ < 0.01)
Test for subgroup differences (random effects): $\chi^2_5$ = 248.53, df = 5 ($p$ < 0.01)

For Peer Review

| Study | Events | Total | Proportion | 95%-CI |
|---|---|---|---|---|
| **Pathology = Various** | | | | |
| Abdelkader 2019 | 18 | 20 | 0.900 | [0.683; 0.988] |
| Bose 2007 | 0 | 5 | 0.000 | [0.000; 0.522] |
| Chen Yu 2021 | 2 | 7 | 0.286 | [0.037; 0.710] |
| Dinner 1986 | 3 | 7 | 0.429 | [0.099; 0.816] |
| Kelleher 2008 | 26 | 35 | 0.743 | [0.567; 0.875] |
| Khan 2006 | 27 | 35 | 0.771 | [0.599; 0.896] |
| Manninen 1998 | 4 | 7 | 0.571 | [0.184; 0.901] |
| May, 2006 | 9 | 10 | 0.900 | [0.555; 0.997] |
| Melachuri 2020 | 11 | 57 | 0.193 | [0.100; 0.319] |
| Melachuri 2019 | 1 | 36 | 0.028 | [0.001; 0.145] |
| Papastefanou 2000 | 10 | 10 | 1.000 | [0.692; 1.000] |
| Park 2011 | 1 | 4 | 0.250 | [0.006; 0.806] |
| Stechison 1995 | 4 | 4 | 1.000 | [0.398; 1.000] |
| Tsirikos_Cervical 2020 | 2 | 11 | 0.182 | [0.023; 0.518] |
| Tsirikos_Cortical 2020 | 2 | 11 | 0.182 | [0.023; 0.518] |
| Yang 1994 | 1 | 1 | 1.000 | [0.025; 1.000] |
| **Common effect model** | | **260** | **0.465** | **[0.406; 0.526]** |
| **Random effects model** | | | **0.520** | **[0.265; 0.765]** |
| Heterogeneity: $I^2 = 78\%$, $\tau^2 = 3.9806$, $p < 0.01$ | | | | |
| **Pathology = Deformity** | | | | |
| Bhagat 2015 | 13 | 23 | 0.565 | [0.345; 0.768] |
| Hu_AmplitudeLatency 2011 | 2 | 2 | 1.000 | [0.158; 1.000] |
| Hu_TFA 2011 | 2 | 2 | 1.000 | [0.158; 1.000] |
| Huang_>50% 2016 | 4 | 4 | 1.000 | [0.398; 1.000] |
| Huang_>60% 2016 | 4 | 4 | 1.000 | [0.398; 1.000] |
| Krishnakumar 2011 | 2 | 2 | 1.000 | [0.158; 1.000] |
| Leung 2005 | 5 | 5 | 1.000 | [0.478; 1.000] |
| Loder 1991 | 28 | 28 | 1.000 | [0.877; 1.000] |
| Luk_CSEP_Cv2 2001 | 0 | 1 | 0.000 | [0.000; 0.975] |
| Luk_CSEP_Cz 2001 | 0 | 1 | 0.000 | [0.000; 0.975] |
| Luk_SCEP 2001 | 1 | 1 | 1.000 | [0.025; 1.000] |
| Luk_SSEP 2001 | 0 | 1 | 0.000 | [0.000; 0.975] |
| Makarov 2012 | 53 | 53 | 1.000 | [0.933; 1.000] |
| Neira 2016 | 7 | 15 | 0.467 | [0.213; 0.734] |
| Noordeen_>25% 1997 | 34 | 37 | 0.919 | [0.781; 0.983] |
| Noordeen_>50% 1997 | 34 | 39 | 0.872 | [0.726; 0.957] |
| Noordeen_>75% 1997 | 46 | 63 | 0.730 | [0.603; 0.834] |
| Padberg 1996 | 1 | 1 | 1.000 | [0.025; 1.000] |
| Qiu 2022 | 3 | 3 | 1.000 | [0.292; 1.000] |
| Randall 1991 | 28 | 28 | 1.000 | [0.877; 1.000] |
| Thirumala 2014 | 19 | 20 | 0.950 | [0.751; 0.999] |
| **Common effect model** | | **333** | **0.859** | **[0.817; 0.892]** |
| **Random effects model** | | | **0.940** | **[0.776; 0.986]** |
| Heterogeneity: $I^2 = 3\%$, $\tau^2 = 5.2433$, $p = 0.42$ | | | | |
| **Pathology = Trauma** | | | | |
| Chandanwale 2008 | 15 | 17 | 0.882 | [0.636; 0.985] |
| Meyer 1988 | 1 | 1 | 1.000 | [0.025; 1.000] |
| Tsirikos_25% 2004 | 2 | 3 | 0.667 | [0.094; 0.992] |
| Tsirikos_50% 2004 | 2 | 3 | 0.667 | [0.094; 0.992] |
| Tsirikos_60% 2004 | 1 | 3 | 0.333 | [0.008; 0.906] |
| Tsirikos_75% 2004 | 1 | 3 | 0.333 | [0.008; 0.906] |
| **Common effect model** | | **30** | **0.733** | **[0.550; 0.861]** |
| **Random effects model** | | | **0.694** | **[0.428; 0.873]** |
| Heterogeneity: $I^2 = 11\%$, $\tau^2 = 0.4518$, $p = 0.35$ | | | | |
| **Pathology = Degenerative** | | | | |
| Chung 2009 | 9 | 9 | 1.000 | [0.664; 1.000] |
| Ding 2008 | 3 | 4 | 0.750 | [0.194; 0.994] |
| Duncan 2012 | 5 | 5 | 1.000 | [0.478; 1.000] |
| Gundanna 2003 | 0 | 5 | 0.000 | [0.000; 0.522] |
| Hilibrand 2006 | 3 | 12 | 0.250 | [0.055; 0.572] |
| Kim 2021 | 0 | 5 | 0.000 | [0.000; 0.522] |
| Kim 2019 | 0 | 7 | 0.000 | [0.000; 0.410] |
| Koffie 2022 | 7 | 21 | 0.333 | [0.146; 0.570] |
| Melachuri 2017 | 3 | 15 | 0.200 | [0.043; 0.481] |
| Smith 2006 | 1 | 1 | 1.000 | [0.025; 1.000] |
| Tohmeh 2022 | 28 | 28 | 1.000 | [0.877; 1.000] |
| Wilent 2020 | 2 | 7 | 0.286 | [0.037; 0.710] |
| Xu 2011 | 1 | 3 | 0.333 | [0.008; 0.906] |
| **Common effect model** | | **122** | **0.508** | **[0.420; 0.596]** |
| **Random effects model** | | | **0.499** | **[0.115; 0.884]** |
| Heterogeneity: $I^2 = 0\%$, $\tau^2 = 10.9146$, $p = 0.99$ | | | | |
| **Pathology = Tumor** | | | | |
| Deutsch 2000 | 0 | 4 | 0.000 | [0.000; 0.602] |
| Feyissa 2015 | 0 | 2 | 0.000 | [0.000; 0.842] |
| Forster 2012 | 14 | 15 | 0.933 | [0.681; 0.998] |
| Izumi 1993 | 5 | 9 | 0.556 | [0.212; 0.863] |
| Park_50% 2018 | 9 | 38 | 0.237 | [0.114; 0.402] |
| Park_AllorNone 2018 | 9 | 37 | 0.243 | [0.118; 0.412] |
| **Common effect model** | | **105** | **0.352** | **[0.267; 0.448]** |
| **Random effects model** | | | **0.332** | **[0.092; 0.709]** |
| Heterogeneity: $I^2 = 67\%$, $\tau^2 = 2.7226$, $p = 0.01$ | | | | |
| **Pathology = Other** | | | | |
| Li_PermanentChanges 2018 | 4 | 9 | 0.444 | [0.137; 0.788] |
| Li_TemporaryChanges 2018 | 1 | 6 | 0.167 | [0.004; 0.641] |
| Paradiso 2006 | 1 | 2 | 0.500 | [0.013; 0.987] |
| **Common effect model** | | **17** | **0.353** | **[0.168; 0.596]** |
| **Random effects model** | | | **0.353** | **[0.168; 0.596]** |
| Heterogeneity: $I^2 = 0\%$, $\tau^2 = 0$, $p = 0.52$ | | | | |
| **Common effect model** | | **867** | **0.616** | **[0.583; 0.648]** |
| **Random effects model** | | | **0.675** | **[0.509; 0.806]** |

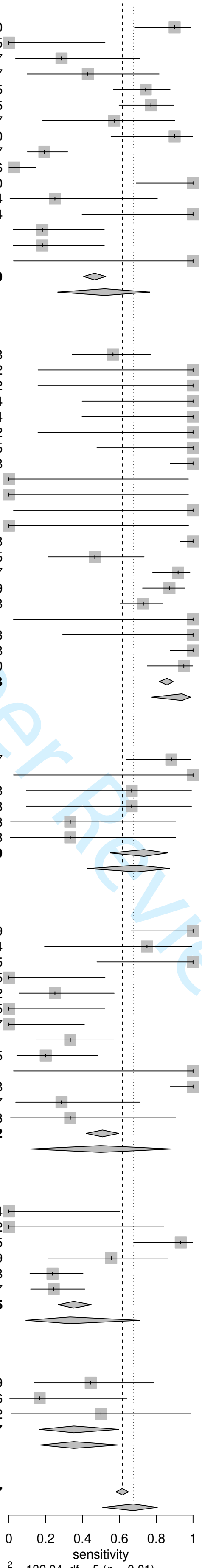


Heterogeneity: $I^2 = 62\%$, $\tau^2 = 5.9269$, $p < 0.01$
Test for subgroup differences (common effect): $\chi^2_5 = 132.04$, df = 5 ($p < 0.01$)
Test for subgroup differences (random effects): $\chi^2_5 = 15.99$, df = 5 ($p < 0.01$)

For Peer Review

**Supplemental Figure 2b. Forest plot for Specificity for SSEP by Disease Group**



| Study | Events | Total | Proportion | 95%−CI |
|---|---|---|---|---|
| **Pathology = Various** | | | | |
| Abdelkader | 21 | 30 | 0.700 | [0.506; 0.853] |
| Bose | 221 | 233 | 0.948 | [0.912; 0.973] |
| Chen Yu | 97 | 105 | 0.924 | [0.855; 0.967] |
| Dinner | 209 | 213 | 0.981 | [0.953; 0.995] |
| Kelleher | 1004 | 1020 | 0.984 | [0.975; 0.991] |
| Khan | 473 | 473 | 1.000 | [0.992; 1.000] |
| Manninen | 253 | 265 | 0.955 | [0.922; 0.976] |
| May | 182 | 206 | 0.883 | [0.832; 0.924] |
| Melachuri | 686 | 714 | 0.961 | [0.944; 0.974] |
| Melachuri | 1013 | 1021 | 0.992 | [0.985; 0.997] |
| Papastefanou | 349 | 409 | 0.853 | [0.815; 0.886] |
| Park | 22 | 23 | 0.957 | [0.781; 0.999] |
| Stechison | 142 | 157 | 0.904 | [0.847; 0.946] |
| Tsirikos_Cervical | 1143 | 1144 | 0.999 | [0.995; 1.000] |
| Tsirikos_Cortical | 1141 | 1144 | 0.997 | [0.992; 0.999] |
| Yang | 9 | 9 | 1.000 | [0.664; 1.000] |
| **Common effect model** | | **7166** | **0.972** | **[0.968; 0.976]** |
| **Random effects model** | | | **0.976** | **[0.942; 0.990]** |
| Heterogeneity: $I^2 = 93\%$, $\tau^2 = 2.8975$, $p < 0.01$ | | | | |
| **Pathology = Deformity** | | | | |
| Bhagat | 328 | 329 | 0.997 | [0.983; 1.000] |
| Hu_AmplitudeLatency | 183 | 189 | 0.968 | [0.932; 0.988] |
| Hu_TFA | 185 | 189 | 0.979 | [0.947; 0.994] |
| Huang_>50% | 146 | 174 | 0.839 | [0.776; 0.890] |
| Huang_>60% | 172 | 174 | 0.989 | [0.959; 0.999] |
| Krishnakumar | 50 | 50 | 1.000 | [0.929; 1.000] |
| Leung | 854 | 866 | 0.986 | [0.976; 0.993] |
| Loder | 37 | 51 | 0.725 | [0.583; 0.841] |
| Luk_CSEP_Cv2 | 27 | 27 | 1.000 | [0.872; 1.000] |
| Luk_CSEP_Cz | 22 | 27 | 0.815 | [0.619; 0.937] |
| Luk_SCEP | 23 | 24 | 0.958 | [0.789; 0.999] |
| Luk_SSEP | 20 | 20 | 1.000 | [0.832; 1.000] |
| Makarov | 248 | 253 | 0.980 | [0.954; 0.994] |
| Neira | 243 | 244 | 0.996 | [0.977; 1.000] |
| Noordeen_>25% | 12 | 48 | 0.250 | [0.136; 0.396] |
| Noordeen_>50% | 20 | 46 | 0.435 | [0.289; 0.589] |
| Noordeen_>75% | 35 | 67 | 0.522 | [0.397; 0.646] |
| Padberg | 66 | 71 | 0.930 | [0.843; 0.977] |
| Qiu | 71 | 73 | 0.973 | [0.905; 0.997] |
| Randall | 37 | 51 | 0.725 | [0.583; 0.841] |
| Thirumala | 456 | 457 | 0.998 | [0.988; 1.000] |
| **Common effect model** | | **3430** | **0.943** | **[0.935; 0.950]** |
| **Random effects model** | | | **0.960** | **[0.897; 0.985]** |
| Heterogeneity: $I^2 = 95\%$, $\tau^2 = 4.6985$, $p < 0.01$ | | | | |
| **Pathology = Trauma** | | | | |
| Chandanwale | 74 | 95 | 0.779 | [0.682; 0.858] |
| Meyer | 140 | 145 | 0.966 | [0.921; 0.989] |
| Tsirikos_25% | 23 | 80 | 0.287 | [0.192; 0.400] |
| Tsirikos_50% | 57 | 80 | 0.713 | [0.600; 0.808] |
| Tsirikos_60% | 65 | 70 | 0.929 | [0.841; 0.976] |
| Tsirikos_75% | 74 | 80 | 0.925 | [0.844; 0.972] |
| **Common effect model** | | **550** | **0.787** | **[0.751; 0.819]** |
| **Random effects model** | | | **0.834** | **[0.614; 0.941]** |
| Heterogeneity: $I^2 = 96\%$, $\tau^2 = 1.9357$, $p < 0.01$ | | | | |
| **Pathology = Degenerative** | | | | |
| Chung | 220 | 221 | 0.995 | [0.975; 1.000] |
| Ding | 71 | 72 | 0.986 | [0.925; 1.000] |
| Duncan | 110 | 110 | 1.000 | [0.967; 1.000] |
| Gundanna | 181 | 181 | 1.000 | [0.980; 1.000] |
| Hilibrand | 415 | 415 | 1.000 | [0.991; 1.000] |
| Kim | 127 | 127 | 1.000 | [0.971; 1.000] |
| Kim | 265 | 268 | 0.989 | [0.968; 0.998] |
| Koffie | 427 | 477 | 0.895 | [0.864; 0.921] |
| Melachuri | 1002 | 1021 | 0.981 | [0.971; 0.989] |
| Smith | 571 | 576 | 0.991 | [0.980; 0.997] |
| Tohmeh | 25 | 28 | 0.893 | [0.718; 0.977] |
| Wilent | 2553 | 2578 | 0.990 | [0.986; 0.994] |
| Xu | 43 | 45 | 0.956 | [0.849; 0.995] |
| **Common effect model** | | **6119** | **0.982** | **[0.979; 0.985]** |
| **Random effects model** | | | **0.991** | **[0.976; 0.997]** |
| Heterogeneity: $I^2 = 91\%$, $\tau^2 = 2.1004$, $p < 0.01$ | | | | |
| **Pathology = Tumor** | | | | |
| Deutsch | 37 | 40 | 0.925 | [0.796; 0.984] |
| Feyissa | 10 | 17 | 0.588 | [0.329; 0.816] |
| Forster | 192 | 198 | 0.970 | [0.935; 0.989] |
| Izumi | 10 | 11 | 0.909 | [0.587; 0.998] |
| Park_50% | 33 | 34 | 0.971 | [0.847; 0.999] |
| Park_AllorNone | 34 | 35 | 0.971 | [0.851; 0.999] |
| **Common effect model** | | **335** | **0.943** | **[0.913; 0.964]** |
| **Random effects model** | | | **0.935** | **[0.834; 0.976]** |
| Heterogeneity: $I^2 = 81\%$, $\tau^2 = 1.1691$, $p < 0.01$ | | | | |
| **Pathology = Other** | | | | |
| Li_PermanentChanges | 13 | 15 | 0.867 | [0.595; 0.983] |
| Li_TemporaryChanges | 13 | 15 | 0.867 | [0.595; 0.983] |
| Paradiso | 34 | 34 | 1.000 | [0.897; 1.000] |
| **Common effect model** | | **64** | **0.937** | **[0.845; 0.976]** |
| **Random effects model** | | | **0.941** | **[0.783; 0.986]** |
| Heterogeneity: $I^2 = 0\%$, $\tau^2 = 0.6848$, $p = 1.00$ | | | | |
| **Common effect model** | | **17664** | **0.963** | **[0.961; 0.966]** |
| **Random effects model** | | | **0.968** | **[0.948; 0.981]** |

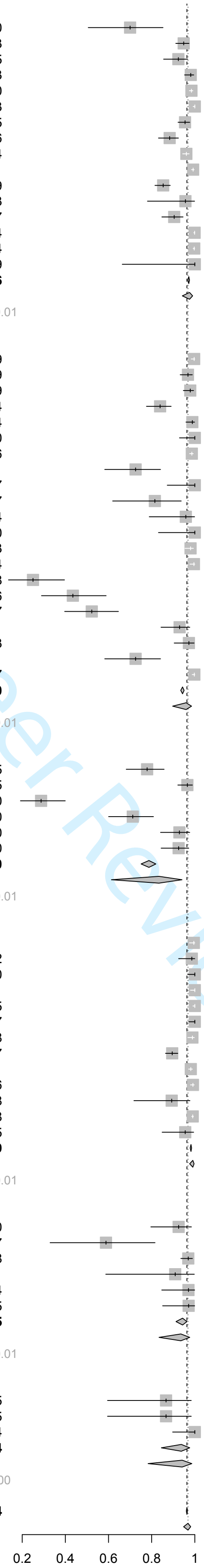


Heterogeneity: $I^2 = 95\%$, $\tau^2 = 3.8246$, $p < 0.01$
Test for subgroup differences (common effect): $\chi^2_5 = 437.87$, df = 5 ($p < 0.01$)
Test for subgroup differences (random effects): $\chi^2_5 = 18.56$, df = 5 ($p < 0.01$)

For Peer Review

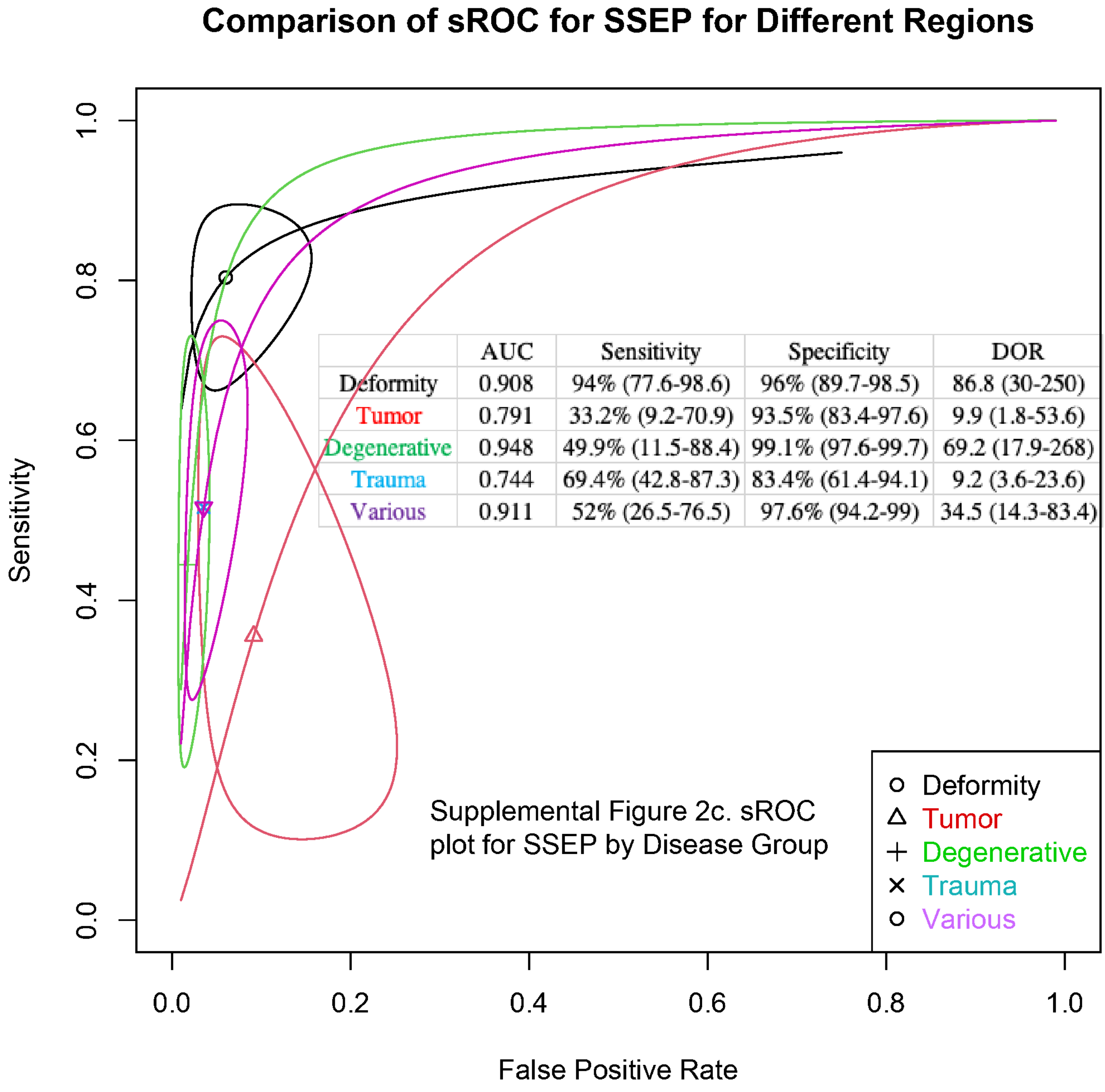


| | AUC | Sensitivity | Specificity | DOR |
|---|---|---|---|---|
| Deformity | 0.908 | 94% (77.6-98.6) | 96% (89.7-98.5) | 86.8 (30-250) |
| Tumor | 0.791 | 33.2% (9.2-70.9) | 93.5% (83.4-97.6) | 9.9 (1.8-53.6) |
| Degenerative | 0.948 | 49.9% (11.5-88.4) | 99.1% (97.6-99.7) | 69.2 (17.9-268) |
| Trauma | 0.744 | 69.4% (42.8-87.3) | 83.4% (61.4-94.1) | 9.2 (3.6-23.6) |
| Various | 0.911 | 52% (26.5-76.5) | 97.6% (94.2-99) | 34.5 (14.3-83.4) |

Supplemental Figure 2c. sROC plot for SSEP by Disease Group

**Supplemental Figure 3a. Forest plot for Sensitivity for SSEP by Region of Surgery**

| Study | Events | Total | Proportion | 95%-CI |
|---|---|---|---|---|
| **Region = All** | | | | |
| Abdelkader 2019 | 18 | 20 | 0.900 | [0.683; 0.988] |
| Bhagat 2015 | 13 | 23 | 0.565 | [0.345; 0.768] |
| Dinner 1986 | 3 | 7 | 0.429 | [0.099; 0.816] |
| Forster 2012 | 14 | 15 | 0.933 | [0.681; 0.998] |
| Huang_>50% 2016 | 4 | 4 | 1.000 | [0.398; 1.000] |
| Huang_>60% 2016 | 4 | 4 | 1.000 | [0.398; 1.000] |
| Krishnakumar 2011 | 2 | 2 | 1.000 | [0.158; 1.000] |
| Leung 2005 | 5 | 5 | 1.000 | [0.478; 1.000] |
| Li_PermanentChanges 2018 | 4 | 9 | 0.444 | [0.137; 0.788] |
| Li_TemporaryChanges 2018 | 1 | 6 | 0.167 | [0.004; 0.641] |
| Luk_CSEP_Cv2 2001 | 0 | 1 | 0.000 | [0.000; 0.975] |
| Luk_CSEP_Cz 2001 | 0 | 1 | 0.000 | [0.000; 0.975] |
| Luk_SCEP 2001 | 1 | 1 | 1.000 | [0.025; 1.000] |
| Luk_SSEP 2001 | 0 | 1 | 0.000 | [0.000; 0.975] |
| Makarov 2012 | 53 | 53 | 1.000 | [0.933; 1.000] |
| Manninen 1998 | 4 | 7 | 0.571 | [0.184; 0.901] |
| Neira 2016 | 7 | 15 | 0.467 | [0.213; 0.734] |
| Noordeen_>25% 1997 | 34 | 37 | 0.919 | [0.781; 0.983] |
| Noordeen_>50% 1997 | 34 | 39 | 0.872 | [0.726; 0.957] |
| Noordeen_>75% 1997 | 46 | 63 | 0.730 | [0.603; 0.834] |
| Padberg 1996 | 1 | 1 | 1.000 | [0.025; 1.000] |
| Papastefanou 2000 | 10 | 10 | 1.000 | [0.692; 1.000] |
| Qiu 2022 | 3 | 3 | 1.000 | [0.292; 1.000] |
| Stechison 1995 | 4 | 4 | 1.000 | [0.398; 1.000] |
| Thirumala 2014 | 19 | 20 | 0.950 | [0.751; 0.999] |
| Tsirikos_25% 2004 | 2 | 3 | 0.667 | [0.094; 0.992] |
| Tsirikos_50% 2004 | 2 | 3 | 0.667 | [0.094; 0.992] |
| Tsirikos_60% 2004 | 1 | 3 | 0.333 | [0.008; 0.906] |
| Tsirikos_75% 2004 | 1 | 3 | 0.333 | [0.008; 0.906] |
| Tsirikos_Cervical 2020 | 2 | 11 | 0.182 | [0.023; 0.518] |
| Tsirikos_Cortical 2020 | 2 | 11 | 0.182 | [0.023; 0.518] |
| **Common effect model** | | **385** | **0.764** | **[0.719; 0.803]** |
| **Random effects model** | | | **0.772** | **[0.604; 0.883]** |
| Heterogeneity: $I^2 = 49\%$, $\tau^2 = 3.2717$, $p < 0.01$ | | | | |
| **Region = Cervical** | | | | |
| Bose 2007 | 0 | 5 | 0.000 | [0.000; 0.522] |
| Chen Yu 2021 | 2 | 7 | 0.286 | [0.037; 0.710] |
| Ding 2008 | 3 | 4 | 0.750 | [0.194; 0.994] |
| Hilibrand 2006 | 3 | 12 | 0.250 | [0.055; 0.572] |
| Kelleher 2008 | 26 | 35 | 0.743 | [0.567; 0.875] |
| Khan 2006 | 27 | 35 | 0.771 | [0.599; 0.896] |
| Kim 2021 | 0 | 5 | 0.000 | [0.000; 0.522] |
| Koffie 2022 | 7 | 21 | 0.333 | [0.146; 0.570] |
| May, 2006 | 9 | 10 | 0.900 | [0.555; 0.997] |
| Smith 2006 | 1 | 1 | 1.000 | [0.025; 1.000] |
| Xu 2011 | 1 | 3 | 0.333 | [0.008; 0.906] |
| **Common effect model** | | **138** | **0.572** | **[0.489; 0.652]** |
| **Random effects model** | | | **0.466** | **[0.243; 0.704]** |
| Heterogeneity: $I^2 = 59\%$, $\tau^2 = 1.9173$, $p < 0.01$ | | | | |
| **Region = Thoracolumbar** | | | | |
| Chandanwale 2008 | 15 | 17 | 0.882 | [0.636; 0.985] |
| Feyissa 2015 | 0 | 2 | 0.000 | [0.000; 0.842] |
| Hu_AmplitudeLatency 2011 | 2 | 2 | 1.000 | [0.158; 1.000] |
| Hu_TFA 2011 | 2 | 2 | 1.000 | [0.158; 1.000] |
| Loder 1991 | 28 | 28 | 1.000 | [0.877; 1.000] |
| Meyer 1988 | 1 | 1 | 1.000 | [0.025; 1.000] |
| Randall 1991 | 28 | 28 | 1.000 | [0.877; 1.000] |
| **Common effect model** | | **80** | **0.950** | **[0.874; 0.981]** |
| **Random effects model** | | | **0.991** | **[0.291; 1.000]** |
| Heterogeneity: $I^2 = 0\%$, $\tau^2 = 16.8841$, $p = 1.00$ | | | | |
| **Region = Lumbosacral** | | | | |
| Chung 2009 | 9 | 9 | 1.000 | [0.664; 1.000] |
| Duncan 2012 | 5 | 5 | 1.000 | [0.478; 1.000] |
| Gundanna 2003 | 0 | 5 | 0.000 | [0.000; 0.522] |
| Kim 2019 | 0 | 7 | 0.000 | [0.000; 0.410] |
| Melachuri 2019 | 1 | 36 | 0.028 | [0.001; 0.145] |
| Melachuri 2017 | 3 | 15 | 0.200 | [0.043; 0.481] |
| Paradiso 2006 | 1 | 2 | 0.500 | [0.013; 0.987] |
| Tohmeh 2022 | 28 | 28 | 1.000 | [0.877; 1.000] |
| Wilent 2020 | 2 | 7 | 0.286 | [0.037; 0.710] |
| **Common effect model** | | **114** | **0.430** | **[0.342; 0.522]** |
| **Random effects model** | | | **0.497** | **[0.035; 0.964]** |
| Heterogeneity: $I^2 = 0\%$, $\tau^2 = 20.5046$, $p = 0.68$ | | | | |
| **Region = Cervicothoracic** | | | | |
| Deutsch 2000 | 0 | 4 | 0.000 | [0.000; 0.602] |
| Izumi 1993 | 5 | 9 | 0.556 | [0.212; 0.863] |
| Melachuri 2020 | 11 | 57 | 0.193 | [0.100; 0.319] |
| Park 2011 | 1 | 4 | 0.250 | [0.006; 0.806] |
| Park_50% 2018 | 9 | 38 | 0.237 | [0.114; 0.402] |
| Park_AllorNone 2018 | 9 | 37 | 0.243 | [0.118; 0.412] |
| Yang 1994 | 1 | 1 | 1.000 | [0.025; 1.000] |
| **Common effect model** | | **150** | **0.240** | **[0.178; 0.315]** |
| **Random effects model** | | | **0.240** | **[0.178; 0.315]** |
| Heterogeneity: $I^2 = 0\%$, $\tau^2 = 0$, $p = 0.56$ | | | | |
| **Common effect model** | | **867** | **0.616** | **[0.583; 0.648]** |
| **Random effects model** | | | **0.675** | **[0.509; 0.806]** |

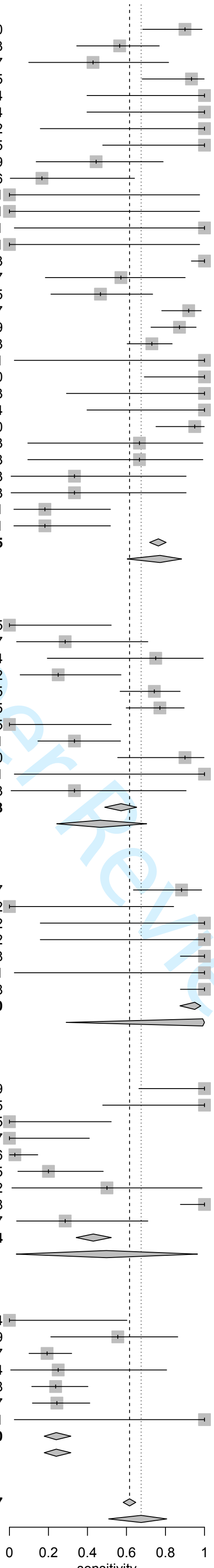


Heterogeneity: $I^2 = 62\%$, $\tau^2 = 5.9269$, $p < 0.01$
Test for subgroup differences (common effect): $\chi^2_4 = 145.45$, df = 4 ($p < 0.01$)
Test for subgroup differences (random effects): $\chi^2_4 = 32.65$, df = 4 ($p < 0.01$)

For Peer Review

**Supplemental Figure 3b. Forest plot for Specificity for SSEP by Region of Surgery**

| Study | Events | Total | Proportion | 95%−CI |
|---|---|---|---|---|
| Region = All | | | | |
| Abdelkader 2019 | 21 | 30 | 0.700 | [0.506; 0.853] |
| Bhagat 2015 | 328 | 329 | 0.997 | [0.983; 1.000] |
| Dinner 1986 | 209 | 213 | 0.981 | [0.953; 0.995] |
| Forster 2012 | 192 | 198 | 0.970 | [0.935; 0.989] |
| Huang_>50% 2016 | 146 | 174 | 0.839 | [0.776; 0.890] |
| Huang_>60% 2016 | 172 | 174 | 0.989 | [0.959; 0.999] |
| Krishnakumar 2011 | 50 | 50 | 1.000 | [0.929; 1.000] |
| Leung 2005 | 854 | 866 | 0.986 | [0.976; 0.993] |
| Li_PermanentChanges 2018 | 13 | 15 | 0.867 | [0.595; 0.983] |
| Li_TemporaryChanges 2018 | 13 | 15 | 0.867 | [0.595; 0.983] |
| Luk_CSEP_Cv2 2001 | 27 | 27 | 1.000 | [0.872; 1.000] |
| Luk_CSEP_Cz 2001 | 22 | 27 | 0.815 | [0.619; 0.937] |
| Luk_SCEP 2001 | 23 | 24 | 0.958 | [0.789; 0.999] |
| Luk_SSEP 2001 | 20 | 20 | 1.000 | [0.832; 1.000] |
| Makarov 2012 | 248 | 253 | 0.980 | [0.954; 0.994] |
| Manninen 1998 | 253 | 265 | 0.955 | [0.922; 0.976] |
| Neira 2016 | 243 | 244 | 0.996 | [0.977; 1.000] |
| Noordeen_>25% 1997 | 12 | 48 | 0.250 | [0.136; 0.396] |
| Noordeen_>50% 1997 | 20 | 46 | 0.435 | [0.289; 0.589] |
| Noordeen_>75% 1997 | 35 | 67 | 0.522 | [0.397; 0.646] |
| Padberg 1996 | 66 | 71 | 0.930 | [0.843; 0.977] |
| Papastefanou 2000 | 349 | 409 | 0.853 | [0.815; 0.886] |
| Qiu 2022 | 71 | 73 | 0.973 | [0.905; 0.997] |
| Stechison 1995 | 142 | 157 | 0.904 | [0.847; 0.946] |
| Thirumala 2014 | 456 | 457 | 0.998 | [0.988; 1.000] |
| Tsirikos_25% 2004 | 23 | 80 | 0.287 | [0.192; 0.400] |
| Tsirikos_50% 2004 | 57 | 80 | 0.713 | [0.600; 0.808] |
| Tsirikos_60% 2004 | 65 | 70 | 0.929 | [0.841; 0.976] |
| Tsirikos_75% 2004 | 74 | 80 | 0.925 | [0.844; 0.972] |
| Tsirikos_Cervical 2020 | 1143 | 1144 | 0.999 | [0.995; 1.000] |
| Tsirikos_Cortical 2020 | 1141 | 1144 | 0.997 | [0.992; 0.999] |
| Common effect model | | 6850 | 0.947 | [0.942; 0.952] |
| Random effects model | | | 0.955 | [0.905; 0.979] |
| Heterogeneity: $I^2 = 95\%$, $\tau^2 = 4.5734$, $p < 0.01$ | | | | |
| Region = Cervical | | | | |
| Bose 2007 | 221 | 233 | 0.948 | [0.912; 0.973] |
| Chen Yu 2021 | 97 | 105 | 0.924 | [0.855; 0.967] |
| Ding 2008 | 71 | 72 | 0.986 | [0.925; 1.000] |
| Hilibrand 2006 | 415 | 415 | 1.000 | [0.991; 1.000] |
| Kelleher 2008 | 1004 | 1020 | 0.984 | [0.975; 0.991] |
| Khan 2006 | 473 | 473 | 1.000 | [0.992; 1.000] |
| Kim 2021 | 127 | 127 | 1.000 | [0.971; 1.000] |
| Koffie 2022 | 427 | 477 | 0.895 | [0.864; 0.921] |
| May, 2006 | 182 | 206 | 0.883 | [0.832; 0.924] |
| Smith 2006 | 571 | 576 | 0.991 | [0.980; 0.997] |
| Xu 2011 | 43 | 45 | 0.956 | [0.849; 0.995] |
| Common effect model | | 3749 | 0.969 | [0.962; 0.974] |
| Random effects model | | | 0.985 | [0.952; 0.996] |
| Heterogeneity: $I^2 = 88\%$, $\tau^2 = 3.2662$, $p < 0.01$ | | | | |
| Region = Thoracolumbar | | | | |
| Chandanwale 2008 | 74 | 95 | 0.779 | [0.682; 0.858] |
| Feyissa 2015 | 10 | 17 | 0.588 | [0.329; 0.816] |
| Hu_AmplitudeLatency 2011 | 183 | 189 | 0.968 | [0.932; 0.988] |
| Hu_TFA 2011 | 185 | 189 | 0.979 | [0.947; 0.994] |
| Loder 1991 | 37 | 51 | 0.725 | [0.583; 0.841] |
| Meyer 1988 | 140 | 145 | 0.966 | [0.921; 0.989] |
| Randall 1991 | 37 | 51 | 0.725 | [0.583; 0.841] |
| Common effect model | | 737 | 0.904 | [0.880; 0.923] |
| Random effects model | | | 0.886 | [0.741; 0.954] |
| Heterogeneity: $I^2 = 91\%$, $\tau^2 = 1.6435$, $p < 0.01$ | | | | |
| Region = Lumbosacral | | | | |
| Chung 2009 | 220 | 221 | 0.995 | [0.975; 1.000] |
| Duncan 2012 | 110 | 110 | 1.000 | [0.967; 1.000] |
| Gundanna 2003 | 181 | 181 | 1.000 | [0.980; 1.000] |
| Kim 2019 | 265 | 268 | 0.989 | [0.968; 0.998] |
| Melachuri 2019 | 1013 | 1021 | 0.992 | [0.985; 0.997] |
| Melachuri 2017 | 1002 | 1021 | 0.981 | [0.971; 0.989] |
| Paradiso 2006 | 34 | 34 | 1.000 | [0.897; 1.000] |
| Tohmeh 2022 | 25 | 28 | 0.893 | [0.718; 0.977] |
| Wilent 2020 | 2553 | 2578 | 0.990 | [0.986; 0.994] |
| Common effect model | | 5462 | 0.989 | [0.986; 0.992] |
| Random effects model | | | 0.990 | [0.983; 0.994] |
| Heterogeneity: $I^2 = 62\%$, $\tau^2 = 0.1745$, $p < 0.01$ | | | | |
| Region = Cervicothoracic | | | | |
| Deutsch 2000 | 37 | 40 | 0.925 | [0.796; 0.984] |
| Izumi 1993 | 10 | 11 | 0.909 | [0.587; 0.998] |
| Melachuri 2020 | 686 | 714 | 0.961 | [0.944; 0.974] |
| Park 2011 | 22 | 23 | 0.957 | [0.781; 0.999] |
| Park_50% 2018 | 33 | 34 | 0.971 | [0.847; 0.999] |
| Park_AllorNone 2018 | 34 | 35 | 0.971 | [0.851; 0.999] |
| Yang 1994 | 9 | 9 | 1.000 | [0.664; 1.000] |
| Common effect model | | 866 | 0.960 | [0.944; 0.971] |
| Random effects model | | | 0.960 | [0.944; 0.971] |
| Heterogeneity: $I^2 = 0\%$, $\tau^2 = 0$, $p = 0.91$ | | | | |
| **Common effect model** | | **17664** | **0.963** | **[0.961; 0.966]** |
| **Random effects model** | | | **0.968** | **[0.948; 0.981]** |

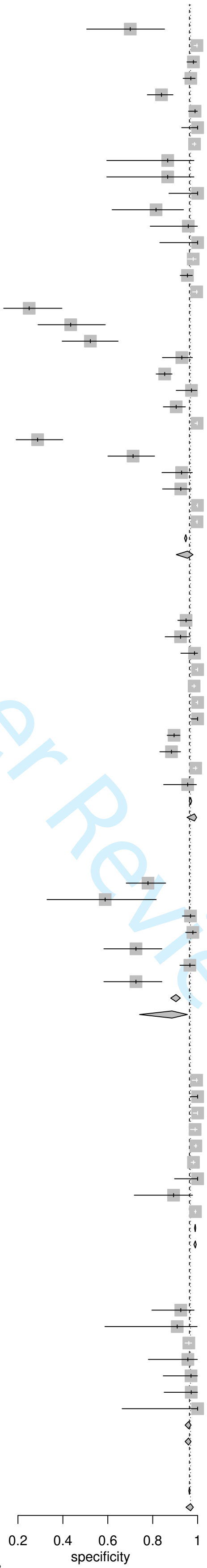


Heterogeneity: $I^2 = 95\%$, $\tau^2 = 3.8246$, $p < 0.01$
Test for subgroup differences (common effect): $\chi^2_4 = 192.79$, df = 4 ($p < 0.01$)
Test for subgroup differences (random effects): $\chi^2_4 = 32.01$, df = 4 ($p < 0.01$)

For Peer Review

## Comparison of sROC for SSEP for Different Regions

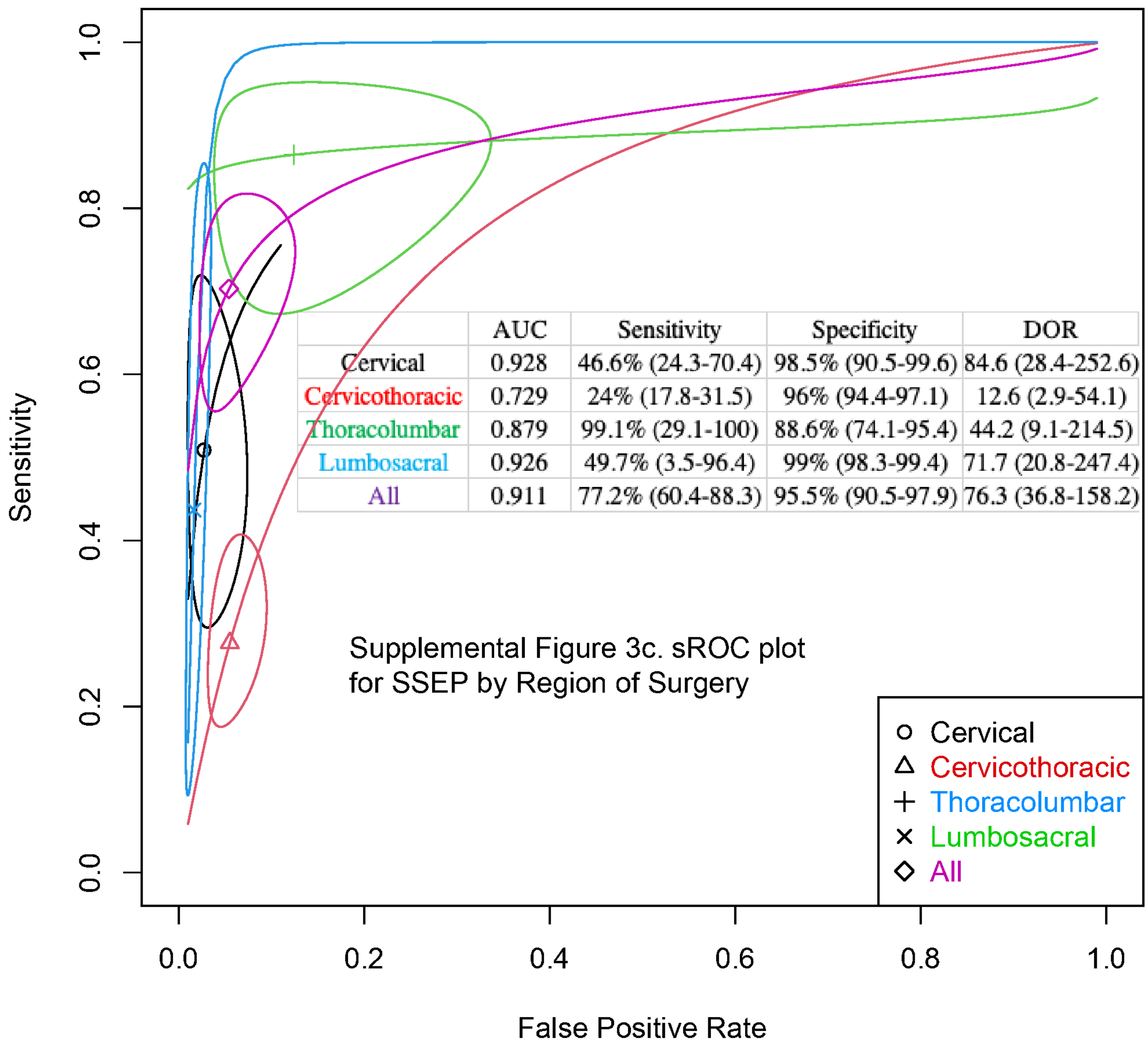


| | AUC | Sensitivity | Specificity | DOR |
|---|---|---|---|---|
| Cervical | 0.928 | 46.6% (24.3-70.4) | 98.5% (90.5-99.6) | 84.6 (28.4-252.6) |
| Cervicothoracic | 0.729 | 24% (17.8-31.5) | 96% (94.4-97.1) | 12.6 (2.9-54.1) |
| Thoracolumbar | 0.879 | 99.1% (29.1-100) | 88.6% (74.1-95.4) | 44.2 (9.1-214.5) |
| Lumbosacral | 0.926 | 49.7% (3.5-96.4) | 99% (98.3-99.4) | 71.7 (20.8-247.4) |
| All | 0.911 | 77.2% (60.4-88.3) | 95.5% (90.5-97.9) | 76.3 (36.8-158.2) |

Supplemental Figure 3c. sROC plot for SSEP by Region of Surgery

Supplemental Figure 4a. Forest plot for Sensitivity for MEP by Threshold Type

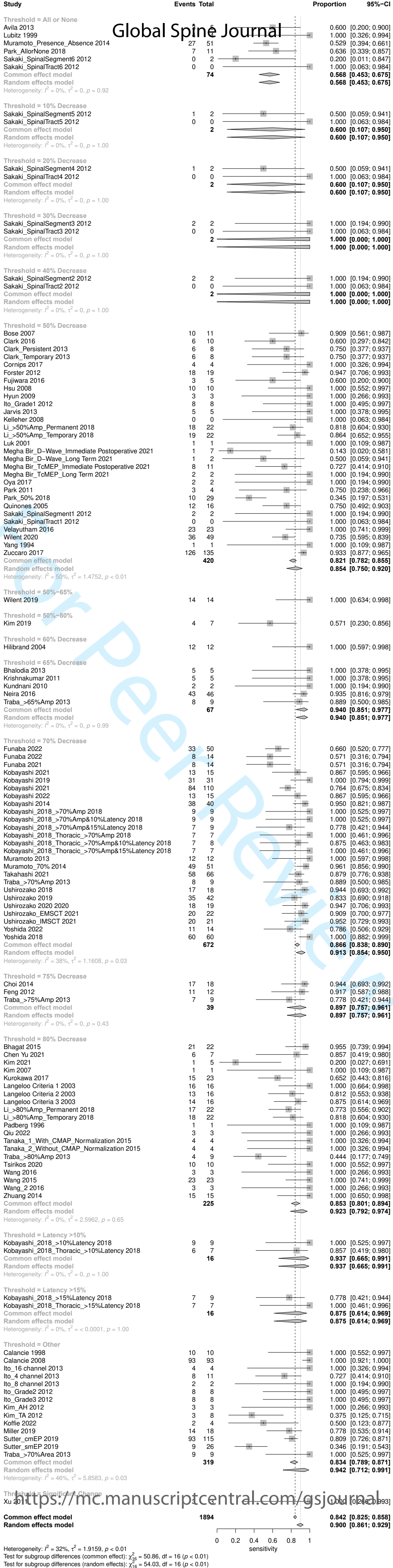


For Peer Review

Supplemental Figure 4b. Forest plot for Specificity for MEP by Threshold Type

Global Spine Journal

| Study | Events | Total | Proportion | 95%−CI |
|---|---|---|---|---|
| **Threshold = All or None** | | | | |
| Avila 2013 | 114 | 117 | 0.974 | [0.927; 0.995] |
| Lubitz 1999 | 45 | 45 | 1.000 | [0.921; 1.000] |
| Muramoto_Presence_Absence 2014 | 213 | 229 | 0.930 | [0.889; 0.960] |
| Park_AllorNone 2018 | 52 | 55 | 0.945 | [0.849; 0.989] |
| Sakaki_SpinalSegment6 2012 | 327 | 333 | 0.982 | [0.961; 0.993] |
| Sakaki_SpinalTract6 2012 | 306 | 335 | 0.913 | [0.878; 0.941] |
| **Random effects model** | | **1114** | **0.961** | **[0.927; 0.980]** |
| Heterogeneity: $I^2 = 69\%$, $\tau^2 = 0.3972$, $p < 0.01$ | | | | |
| **Threshold = 10% Decrease** | | | | |
| Sakaki_SpinalSegment5 2012 | 323 | 333 | 0.970 | [0.945; 0.986] |
| Sakaki_SpinalTract5 2012 | 296 | 335 | 0.884 | [0.844; 0.916] |
| **Random effects model** | | **668** | **0.939** | **[0.847; 0.977]** |
| Heterogeneity: $I^2 = 94\%$, $\tau^2 = 0.4764$, $p < 0.01$ | | | | |
| **Threshold = 20% Decrease** | | | | |
| Sakaki_SpinalSegment4 2012 | 316 | 333 | 0.949 | [0.920; 0.970] |
| Sakaki_SpinalTract4 2012 | 278 | 335 | 0.830 | [0.785; 0.869] |
| **Random effects model** | | **668** | **0.904** | **[0.786; 0.960]** |
| Heterogeneity: $I^2 = 95\%$, $\tau^2 = 0.4165$, $p < 0.01$ | | | | |
| **Threshold = 30% Decrease** | | | | |
| Sakaki_SpinalSegment3 2012 | 301 | 333 | 0.904 | [0.867; 0.933] |
| Sakaki_SpinalTract3 2012 | 263 | 335 | 0.785 | [0.737; 0.828] |
| **Random effects model** | | **668** | **0.853** | **[0.750; 0.918]** |
| Heterogeneity: $I^2 = 94\%$, $\tau^2 = 0.2006$, $p < 0.01$ | | | | |
| **Threshold = 40% Decrease** | | | | |
| Sakaki_SpinalSegment2 2012 | 284 | 333 | 0.853 | [0.810; 0.889] |
| Sakaki_SpinalTract2 2012 | 243 | 335 | 0.725 | [0.674; 0.772] |
| **Random effects model** | | **668** | **0.796** | **[0.692; 0.871]** |
| Heterogeneity: $I^2 = 94\%$, $\tau^2 = 0.1364$, $p < 0.01$ | | | | |
| **Threshold = 50% Decrease** | | | | |
| Bose 2007 | 202 | 226 | 0.894 | [0.846; 0.931] |
| Clark 2016 | 118 | 134 | 0.881 | [0.813; 0.930] |
| Clark_Persistent 2013 | 130 | 132 | 0.985 | [0.946; 0.998] |
| Clark_Temporary 2013 | 122 | 132 | 0.924 | [0.865; 0.963] |
| Cornips 2017 | 42 | 59 | 0.712 | [0.579; 0.822] |
| Forster 2012 | 181 | 183 | 0.989 | [0.961; 0.999] |
| Fujiwara 2016 | 155 | 155 | 1.000 | [0.976; 1.000] |
| Hsu 2008 | 157 | 162 | 0.969 | [0.929; 0.990] |
| Hyun 2009 | 6 | 15 | 0.400 | [0.163; 0.677] |
| Ito_Grade1 2012 | 181 | 287 | 0.631 | [0.572; 0.687] |
| Jarvis 2013 | 16 | 32 | 0.500 | [0.319; 0.681] |
| Kelleher 2008 | 25 | 26 | 0.962 | [0.804; 0.999] |
| Li_>50%Amp_Permanent 2018 | 23 | 31 | 0.742 | [0.554; 0.881] |
| Li_>50%Amp_Temporary 2018 | 18 | 31 | 0.581 | [0.391; 0.755] |
| Luk 2001 | 23 | 23 | 1.000 | [0.852; 1.000] |
| Megha Bir_D−Wave_Immediate Postoperative 2021 | 9 | 9 | 1.000 | [0.664; 1.000] |
| Megha Bir_D−Wave_Long Term 2021 | 12 | 12 | 1.000 | [0.735; 1.000] |
| Megha Bir_TcMEP_Immediate Postoperative 2021 | 12 | 12 | 1.000 | [0.735; 1.000] |
| Megha Bir_TcMEP_Long Term 2021 | 11 | 18 | 0.611 | [0.357; 0.827] |
| Oya 2017 | 127 | 129 | 0.984 | [0.945; 0.998] |
| Park 2011 | 21 | 25 | 0.840 | [0.639; 0.955] |
| Park_50% 2018 | 37 | 37 | 1.000 | [0.905; 1.000] |
| Quinones 2005 | 11 | 11 | 1.000 | [0.715; 1.000] |
| Sakaki_SpinalSegment1 2012 | 277 | 333 | 0.832 | [0.787; 0.870] |
| Sakaki_SpinalTract1 2012 | 222 | 335 | 0.663 | [0.609; 0.713] |
| Velayutham 2016 | 272 | 277 | 0.982 | [0.958; 0.994] |
| Wilent 2020 | 40638 | 40870 | 0.994 | [0.994; 0.995] |
| Yang 1994 | 9 | 9 | 1.000 | [0.664; 1.000] |
| Zuccaro 2017 | 2362 | 2596 | 0.910 | [0.898; 0.921] |
| **Random effects model** | | **46301** | **0.942** | **[0.882; 0.972]** |
| Heterogeneity: $I^2 = 99\%$, $\tau^2 = 3.4257$, $p = 0$ | | | | |
| **Threshold = 50%−65%** | | | | |
| Wilent 2019 | 4282 | 4372 | 0.979 | [0.975; 0.983] |
| **Threshold = 50%−80%** | | | | |
| Kim 2019 | 270 | 270 | 1.000 | [0.986; 1.000] |
| **Threshold = 60% Decrease** | | | | |
| Hilibrand 2004 | 415 | 415 | 1.000 | [0.991; 1.000] |
| **Threshold = 65% Decrease** | | | | |
| Bhalodia 2013 | 448 | 456 | 0.982 | [0.966; 0.992] |
| Krishnakumar 2011 | 47 | 47 | 1.000 | [0.925; 1.000] |
| Kundnani 2010 | 341 | 350 | 0.974 | [0.952; 0.988] |
| Neira 2016 | 220 | 222 | 0.991 | [0.968; 0.999] |
| Traba_>65%Amp 2013 | 175 | 178 | 0.983 | [0.952; 0.997] |
| **Random effects model** | | **1253** | **0.982** | **[0.973; 0.988]** |
| Heterogeneity: $I^2 = 0\%$, $\tau^2 = 0$, $p = 0.72$ | | | | |
| **Threshold = 70% Decrease** | | | | |
| Funaba 2022 | 2343 | 2546 | 0.920 | [0.909; 0.930] |
| Funaba 2022 | 1049 | 1151 | 0.911 | [0.893; 0.927] |
| Funaba 2021 | 1049 | 1151 | 0.911 | [0.893; 0.927] |
| Kobayashi 2021 | 195 | 218 | 0.894 | [0.846; 0.932] |
| Kobayashi 2019 | 105 | 128 | 0.820 | [0.743; 0.883] |
| Kobayashi 2021 | 3089 | 3450 | 0.895 | [0.885; 0.905] |
| Kobayashi 2022 | 195 | 218 | 0.894 | [0.846; 0.932] |
| Kobayashi 2014 | 786 | 864 | 0.910 | [0.889; 0.928] |
| Kobayashi_2018_>70%Amp 2018 | 53 | 74 | 0.716 | [0.599; 0.815] |
| Kobayashi_2018_>70%Amp&10%Latency 2018 | 69 | 74 | 0.932 | [0.849; 0.978] |
| Kobayashi_2018_>70%Amp&15%Latency 2018 | 72 | 74 | 0.973 | [0.906; 0.997] |
| Kobayashi_2018_Thoracic_>70%Amp 2018 | 45 | 63 | 0.714 | [0.587; 0.821] |
| Kobayashi_2018_Thoracic_>70%Amp&10%Latency 2018 | 61 | 62 | 0.984 | [0.913; 1.000] |
| Kobayashi_2018_Thoracic_>70%Amp&15%Latency 2018 | 55 | 63 | 0.873 | [0.765; 0.944] |
| Muramoto 2013 | 48 | 64 | 0.750 | [0.626; 0.850] |
| Muramoto_70% 2014 | 113 | 229 | 0.493 | [0.427; 0.560] |
| Takahashi 2021 | 1621 | 1798 | 0.902 | [0.887; 0.915] |
| Traba_>70%Amp 2013 | 177 | 178 | 0.994 | [0.969; 1.000] |
| Ushirozako 2018 | 248 | 265 | 0.936 | [0.899; 0.962] |
| Ushirozako 2019 | 541 | 639 | 0.847 | [0.816; 0.874] |
| Ushirozako 2020 2020 | 285 | 361 | 0.789 | [0.744; 0.830] |
| Ushirozako_EMSCT 2021 | 263 | 292 | 0.901 | [0.860; 0.932] |
| Ushirozako_IMSCT 2021 | 41 | 51 | 0.804 | [0.669; 0.902] |
| Yoshida 2022 | 261 | 297 | 0.879 | [0.836; 0.914] |
| Yoshida 2018 | 746 | 746 | 1.000 | [0.995; 1.000] |
| **Random effects model** | | **15056** | **0.904** | **[0.854; 0.939]** |
| Heterogeneity: $I^2 = 94\%$, $\tau^2 = 1.3941$, $p < 0.01$ | | | | |
| **Threshold = 75% Decrease** | | | | |
| Choi 2014 | 30 | 32 | 0.938 | [0.792; 0.992] |
| Feng 2012 | 158 | 160 | 0.988 | [0.956; 0.998] |
| Traba_>75%Amp 2013 | 178 | 178 | 1.000 | [0.979; 1.000] |
| **Random effects model** | | **370** | **0.990** | **[0.941; 0.998]** |
| Heterogeneity: $I^2 = 25\%$, $\tau^2 = 1.3930$, $p = 0.27$ | | | | |
| **Threshold = 80% Decrease** | | | | |
| Bhagat 2015 | 292 | 294 | 0.993 | [0.976; 0.999] |
| Chen Yu 2021 | 100 | 104 | 0.962 | [0.904; 0.989] |
| Kim 2021 | 111 | 118 | 0.941 | [0.882; 0.976] |
| Kim 2007 | 46 | 51 | 0.902 | [0.786; 0.967] |
| Kurokawa 2017 | 105 | 127 | 0.827 | [0.750; 0.888] |
| Langeloo Criteria 1 2003 | 106 | 116 | 0.914 | [0.847; 0.958] |
| Langeloo Criteria 2 2003 | 112 | 116 | 0.966 | [0.914; 0.991] |
| Langeloo Criteria 3 2003 | 110 | 116 | 0.948 | [0.891; 0.981] |
| Li_>80%Amp_Permanent 2018 | 27 | 31 | 0.871 | [0.702; 0.964] |
| Li_>80%Amp_Temporary 2018 | 22 | 31 | 0.710 | [0.520; 0.858] |
| Padberg 1996 | 66 | 71 | 0.930 | [0.843; 0.977] |
| Qiu 2022 | 81 | 82 | 0.988 | [0.934; 1.000] |
| Tanaka_1_With_CMAP_Normalization 2015 | 187 | 192 | 0.974 | [0.940; 0.991] |
| Tanaka_2_Without_CMAP_Normalization 2015 | 186 | 192 | 0.969 | [0.933; 0.988] |
| Traba_>80%Amp 2013 | 178 | 178 | 1.000 | [0.979; 1.000] |
| Tsirikos 2020 | 1140 | 1145 | 0.996 | [0.990; 0.999] |
| Wang 2016 | 112 | 114 | 0.982 | [0.938; 0.998] |
| Wang 2015 | 305 | 309 | 0.987 | [0.967; 0.996] |
| Wang_2 2016 | 16 | 18 | 0.889 | [0.653; 0.986] |
| Zhuang 2014 | 1137 | 1147 | 0.991 | [0.984; 0.996] |
| **Random effects model** | | **4552** | **0.967** | **[0.942; 0.981]** |
| Heterogeneity: $I^2 = 88\%$, $\tau^2 = 1.4734$, $p < 0.01$ | | | | |
| **Threshold = Latency >10%** | | | | |
| Kobayashi_2018_>10%Latency 2018 | 62 | 74 | 0.838 | [0.734; 0.913] |
| Kobayashi_2018_Thoracic_>10%Latency 2018 | 61 | 63 | 0.968 | [0.890; 0.996] |
| **Random effects model** | | **137** | **0.918** | **[0.762; 0.975]** |
| Heterogeneity: $I^2 = 80\%$, $\tau^2 = 0.5387$, $p = 0.02$ | | | | |
| **Threshold = Latency >15%** | | | | |
| Kobayashi_2018_>15%Latency 2018 | 71 | 74 | 0.959 | [0.886; 0.992] |
| Kobayashi_2018_Thoracic_>15%Latency 2018 | 55 | 63 | 0.873 | [0.765; 0.944] |
| **Random effects model** | | **137** | **0.923** | **[0.836; 0.965]** |
| Heterogeneity: $I^2 = 68\%$, $\tau^2 = 0.1526$, $p = 0.08$ | | | | |
| **Threshold = Other** | | | | |
| Calancie 1998 | 22 | 22 | 1.000 | [0.846; 1.000] |
| Calancie 2008 | 808 | 810 | 0.998 | [0.991; 1.000] |
| Ito_16 channel 2013 | 134 | 141 | 0.950 | [0.900; 0.980] |
| Ito_4 channel 2013 | 602 | 650 | 0.926 | [0.903; 0.945] |
| Ito_8 channel 2013 | 35 | 36 | 0.972 | [0.855; 0.999] |
| Ito_Grade2 2012 | 228 | 287 | 0.794 | [0.743; 0.840] |
| Ito_Grade3 2012 | 256 | 287 | 0.892 | [0.850; 0.925] |
| Kim_AH 2012 | 328 | 329 | 0.997 | [0.983; 1.000] |
| Kim_TA 2012 | 243 | 243 | 1.000 | [0.985; 1.000] |
| Koffie 2022 | 86 | 117 | 0.735 | [0.645; 0.812] |
| Miller 2019 | 99 | 107 | 0.925 | [0.858; 0.967] |
| Sutter_cmEP 2019 | 2582 | 2600 | 0.993 | [0.989; 0.996] |
| Sutter_smEP 2019 | 1397 | 1399 | 0.999 | [0.995; 1.000] |
| Traba_>70%Area 2013 | 178 | 178 | 1.000 | [0.979; 1.000] |
| **Random effects model** | | **7206** | **0.985** | **[0.951; 0.996]** |
| Heterogeneity: $I^2 = 96\%$, $\tau^2 = 4.4404$, $p < 0.01$ | | | | |
| **Threshold = Significant Change** | | | | |
| Xu 2011 | 53 | 54 | 0.981 | [0.901; 1.000] |
| **Random effects model** | | **83909** | **0.956** | **[0.940; 0.967]** |

Heterogeneity: $I^2 = 97\%$, $\tau^2 = 2.6633$, $p = 0$
Test for subgroup differences: $\chi^2_{16} = 144.69$, df = 16 ($p < 0.01$)



For Peer Review

**Supplemental Figure 5a. Forest plot for Sensitivity for MEP by Disease Group**

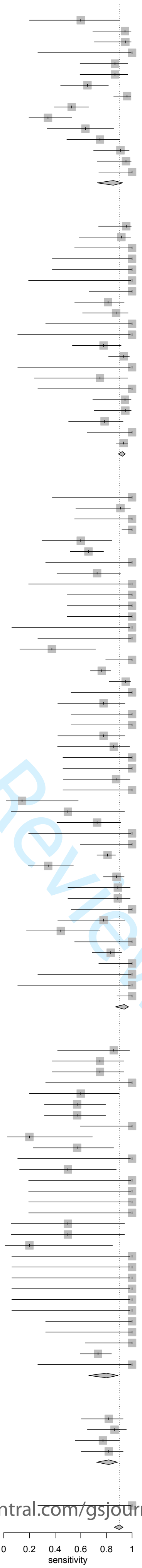


| Study | Events | Total | Proportion | 95%−CI |
|---|---|---|---|---|
| **Pathology = Tumor** | | | | |
| Avila 2013 | 3 | 5 | 0.600 | [0.200; 0.900] |
| Choi 2014 | 17 | 18 | 0.944 | [0.693; 0.992] |
| Forster 2012 | 18 | 19 | 0.947 | [0.706; 0.993] |
| Hyun 2009 | 3 | 3 | 1.000 | [0.266; 0.993] |
| Kobayashi 2021 | 13 | 15 | 0.867 | [0.595; 0.966] |
| Kobayashi 2022 | 13 | 15 | 0.867 | [0.595; 0.966] |
| Kurokawa 2017 | 15 | 23 | 0.652 | [0.443; 0.816] |
| Muramoto_70% 2014 | 49 | 51 | 0.961 | [0.856; 0.990] |
| Muramoto_Presence_Absence 2014 | 27 | 51 | 0.529 | [0.394; 0.661] |
| Park_50% 2018 | 10 | 29 | 0.345 | [0.197; 0.531] |
| Park_AllorNone 2018 | 7 | 11 | 0.636 | [0.339; 0.857] |
| Quinones 2005 | 12 | 16 | 0.750 | [0.492; 0.903] |
| Ushirozako_EMSCT 2021 | 20 | 22 | 0.909 | [0.700; 0.977] |
| Ushirozako_IMSCT 2021 | 20 | 21 | 0.952 | [0.729; 0.993] |
| Velayutham 2016 | 23 | 23 | 1.000 | [0.741; 0.999] |
| Random effects model | | **322** | **0.854** | **[0.729; 0.927]** |
| Heterogeneity: $I^2 = 73\%$, $\tau^2 = 1.5875$, $p < 0.01$ | | | | |
| **Pathology = Deformity** | | | | |
| Bhagat 2015 | 21 | 22 | 0.955 | [0.739; 0.994] |
| Feng 2012 | 11 | 12 | 0.917 | [0.587; 0.988] |
| Hsu 2008 | 10 | 10 | 1.000 | [0.552; 0.997] |
| Jarvis 2013 | 5 | 5 | 1.000 | [0.378; 0.995] |
| Krishnakumar 2011 | 5 | 5 | 1.000 | [0.378; 0.995] |
| Kundnani 2010 | 2 | 2 | 1.000 | [0.194; 0.990] |
| Langeloo Criteria 1 2003 | 16 | 16 | 1.000 | [0.664; 0.998] |
| Langeloo Criteria 2 2003 | 13 | 16 | 0.812 | [0.553; 0.938] |
| Langeloo Criteria 3 2003 | 14 | 16 | 0.875 | [0.614; 0.969] |
| Lubitz 1999 | 4 | 4 | 1.000 | [0.326; 0.994] |
| Luk 2001 | 1 | 1 | 1.000 | [0.109; 0.987] |
| Miller 2019 | 14 | 18 | 0.778 | [0.535; 0.914] |
| Neira 2016 | 43 | 46 | 0.935 | [0.816; 0.979] |
| Padberg 1996 | 1 | 1 | 1.000 | [0.109; 0.987] |
| Park 2011 | 3 | 4 | 0.750 | [0.238; 0.966] |
| Qiu 2022 | 3 | 3 | 1.000 | [0.266; 0.993] |
| Ushirozako 2018 | 17 | 18 | 0.944 | [0.693; 0.992] |
| Ushirozako 2020 2020 | 18 | 19 | 0.947 | [0.706; 0.993] |
| Yoshida 2022 | 11 | 14 | 0.786 | [0.506; 0.929] |
| Zhuang 2014 | 15 | 15 | 1.000 | [0.650; 0.998] |
| Zuccaro 2017 | 126 | 135 | 0.933 | [0.877; 0.965] |
| Random effects model | | **382** | **0.924** | **[0.893; 0.947]** |
| Heterogeneity: $I^2 = 0\%$, $\tau^2 = 0$, $p = 0.95$ | | | | |
| **Pathology = Various** | | | | |
| Bhalodia 2013 | 5 | 5 | 1.000 | [0.378; 0.995] |
| Bose 2007 | 10 | 11 | 0.909 | [0.561; 0.987] |
| Calancie 1998 | 10 | 10 | 1.000 | [0.552; 0.997] |
| Calancie 2008 | 93 | 93 | 1.000 | [0.921; 1.000] |
| Clark 2016 | 6 | 10 | 0.600 | [0.297; 0.842] |
| Funaba 2022 | 33 | 50 | 0.660 | [0.520; 0.777] |
| Ito_16 channel 2013 | 4 | 4 | 1.000 | [0.326; 0.994] |
| Ito_4 channel 2013 | 8 | 11 | 0.727 | [0.414; 0.910] |
| Ito_8 channel 2013 | 2 | 2 | 1.000 | [0.194; 0.990] |
| Ito_Grade1 2012 | 8 | 8 | 1.000 | [0.495; 0.997] |
| Ito_Grade2 2012 | 8 | 8 | 1.000 | [0.495; 0.997] |
| Ito_Grade3 2012 | 8 | 8 | 1.000 | [0.495; 0.997] |
| Kelleher 2008 | 0 | 0 | 1.000 | [0.063; 0.984] |
| Kim_AH 2012 | 3 | 3 | 1.000 | [0.266; 0.993] |
| Kim_TA 2012 | 3 | 8 | 0.375 | [0.125; 0.715] |
| Kobayashi 2019 | 31 | 31 | 1.000 | [0.794; 0.999] |
| Kobayashi 2021 | 84 | 110 | 0.764 | [0.675; 0.834] |
| Kobayashi 2014 | 38 | 40 | 0.950 | [0.821; 0.987] |
| Kobayashi_2018_>10%Latency 2018 | 9 | 9 | 1.000 | [0.525; 0.997] |
| Kobayashi_2018_>15%Latency 2018 | 7 | 9 | 0.778 | [0.421; 0.944] |
| Kobayashi_2018_>70%Amp 2018 | 9 | 9 | 1.000 | [0.525; 0.997] |
| Kobayashi_2018_>70%Amp&10%Latency 2018 | 9 | 9 | 1.000 | [0.525; 0.997] |
| Kobayashi_2018_>70%Amp&15%Latency 2018 | 7 | 9 | 0.778 | [0.421; 0.944] |
| Kobayashi_2018_Thoracic_>10%Latency 2018 | 6 | 7 | 0.857 | [0.419; 0.980] |
| Kobayashi_2018_Thoracic_>15%Latency 2018 | 7 | 7 | 1.000 | [0.461; 0.996] |
| Kobayashi_2018_Thoracic_>70%Amp 2018 | 7 | 7 | 1.000 | [0.461; 0.996] |
| Kobayashi_2018_Thoracic_>70%Amp&10%Latency 2018 | 7 | 8 | 0.875 | [0.463; 0.983] |
| Kobayashi_2018_Thoracic_>70%Amp&15%Latency 2018 | 7 | 7 | 1.000 | [0.461; 0.996] |
| Megha Bir_D−Wave_Immediate Postoperative 2021 | 1 | 7 | 0.143 | [0.020; 0.581] |
| Megha Bir_D−Wave_Long Term 2021 | 1 | 2 | 0.500 | [0.059; 0.941] |
| Megha Bir_TcMEP_Immediate Postoperative 2021 | 8 | 11 | 0.727 | [0.414; 0.910] |
| Megha Bir_TcMEP_Long Term 2021 | 2 | 2 | 1.000 | [0.194; 0.990] |
| Muramoto 2013 | 12 | 12 | 1.000 | [0.597; 0.998] |
| Sutter_cmEP 2019 | 93 | 115 | 0.809 | [0.726; 0.871] |
| Sutter_smEP 2019 | 9 | 26 | 0.346 | [0.191; 0.543] |
| Takahashi 2021 | 58 | 66 | 0.879 | [0.776; 0.938] |
| Traba_>65%Amp 2013 | 8 | 9 | 0.889 | [0.500; 0.985] |
| Traba_>70%Amp 2013 | 8 | 9 | 0.889 | [0.500; 0.985] |
| Traba_>70%Area 2013 | 9 | 9 | 1.000 | [0.525; 0.997] |
| Traba_>75%Amp 2013 | 7 | 9 | 0.778 | [0.421; 0.944] |
| Traba_>80%Amp 2013 | 4 | 9 | 0.444 | [0.177; 0.749] |
| Tsirikos 2020 | 10 | 10 | 1.000 | [0.552; 0.997] |
| Ushirozako 2019 | 35 | 42 | 0.833 | [0.690; 0.918] |
| Wang 2015 | 23 | 23 | 1.000 | [0.741; 0.999] |
| Wang_2 2016 | 3 | 3 | 1.000 | [0.266; 0.993] |
| Yang 1994 | 1 | 1 | 1.000 | [0.109; 0.987] |
| Yoshida 2018 | 60 | 60 | 1.000 | [0.882; 0.999] |
| Random effects model | | **918** | **0.939** | **[0.871; 0.972]** |
| Heterogeneity: $I^2 = 20\%$, $\tau^2 = 3.6309$, $p = 0.12$ | | | | |
| **Pathology = Degenerative** | | | | |
| Chen Yu 2021 | 6 | 7 | 0.857 | [0.419; 0.980] |
| Clark_Persistent 2013 | 6 | 8 | 0.750 | [0.377; 0.937] |
| Clark_Temporary 2013 | 6 | 8 | 0.750 | [0.377; 0.937] |
| Cornips 2017 | 4 | 4 | 1.000 | [0.326; 0.994] |
| Fujiwara 2016 | 3 | 5 | 0.600 | [0.200; 0.900] |
| Funaba 2022 | 8 | 14 | 0.571 | [0.316; 0.794] |
| Funaba 2021 | 8 | 14 | 0.571 | [0.316; 0.794] |
| Hilibrand 2004 | 12 | 12 | 1.000 | [0.597; 0.998] |
| Kim 2021 | 1 | 5 | 0.200 | [0.027; 0.691] |
| Kim 2019 | 4 | 7 | 0.571 | [0.230; 0.856] |
| Kim 2007 | 1 | 1 | 1.000 | [0.109; 0.987] |
| Koffie 2022 | 2 | 4 | 0.500 | [0.123; 0.877] |
| Oya 2017 | 2 | 2 | 1.000 | [0.194; 0.990] |
| Sakaki_SpinalSegment1 2012 | 2 | 2 | 1.000 | [0.194; 0.990] |
| Sakaki_SpinalSegment2 2012 | 2 | 2 | 1.000 | [0.194; 0.990] |
| Sakaki_SpinalSegment3 2012 | 2 | 2 | 1.000 | [0.194; 0.990] |
| Sakaki_SpinalSegment4 2012 | 1 | 2 | 0.500 | [0.059; 0.941] |
| Sakaki_SpinalSegment5 2012 | 1 | 2 | 0.500 | [0.059; 0.941] |
| Sakaki_SpinalSegment6 2012 | 0 | 2 | 0.200 | [0.011; 0.847] |
| Sakaki_SpinalTract1 2012 | 0 | 0 | 1.000 | [0.063; 0.984] |
| Sakaki_SpinalTract2 2012 | 0 | 0 | 1.000 | [0.063; 0.984] |
| Sakaki_SpinalTract3 2012 | 0 | 0 | 1.000 | [0.063; 0.984] |
| Sakaki_SpinalTract4 2012 | 0 | 0 | 1.000 | [0.063; 0.984] |
| Sakaki_SpinalTract5 2012 | 0 | 0 | 1.000 | [0.063; 0.984] |
| Sakaki_SpinalTract6 2012 | 0 | 0 | 1.000 | [0.063; 0.984] |
| Tanaka_1_With_CMAP_Normalization 2015 | 4 | 4 | 1.000 | [0.326; 0.994] |
| Tanaka_2_Without_CMAP_Normalization 2015 | 4 | 4 | 1.000 | [0.326; 0.994] |
| Wilent 2019 | 14 | 14 | 1.000 | [0.634; 0.998] |
| Wilent 2020 | 36 | 49 | 0.735 | [0.595; 0.839] |
| Xu 2011 | 3 | 3 | 1.000 | [0.266; 0.993] |
| Random effects model | | **180** | **0.800** | **[0.662; 0.890]** |
| Heterogeneity: $I^2 = 0\%$, $\tau^2 = 1.0002$, $p = 1.00$ | | | | |
| **Pathology = AVM** | | | | |
| Li_>50%Amp_Permanent 2018 | 18 | 22 | 0.818 | [0.604; 0.930] |
| Li_>50%Amp_Temporary 2018 | 19 | 22 | 0.864 | [0.652; 0.955] |
| Li_>80%Amp_Permanent 2018 | 17 | 22 | 0.773 | [0.556; 0.902] |
| Li_>80%Amp_Temporary 2018 | 18 | 22 | 0.818 | [0.604; 0.930] |
| Random effects model | | **88** | **0.818** | **[0.724; 0.886]** |
| Heterogeneity: $I^2 = 0\%$, $\tau^2 = 0$, $p = 0.90$ | | | | |
| **Pathology = Neural Axis Abnormality** | | | | |
| Wang 2016 | 3 | 3 | 1.000 | [0.266; 0.993] |
| **Random effects model** | | **1894** | **0.900** | **[0.861; 0.929]** |
| Heterogeneity: $I^2 = 32\%$, $\tau^2 = 1.9159$, $p < 0.01$ | | | | |
| Test for subgroup differences: $\chi^2_5 = 15.81$, df = 5 ($p < 0.01$) | | | | |

For Peer Review

Supplemental Figure 5b. Forest plot for Specificity for MEP by Disease Group

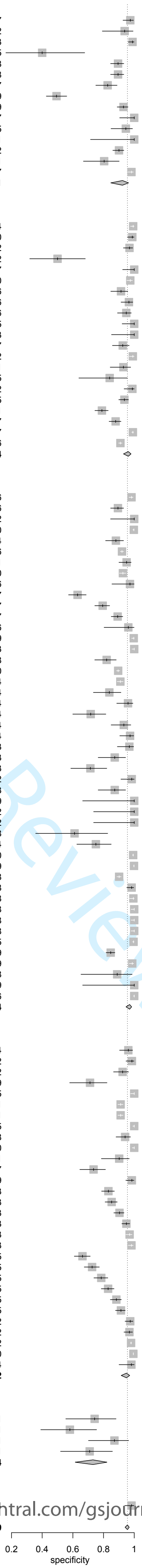

| Study | Events | Total | Proportion | 95%-CI |
|---|---|---|---|---|
| **Pathology = Tumor** | | | | |
| Avila 2013 | 114 | 117 | 0.974 | [0.927; 0.995] |
| Choi 2014 | 30 | 32 | 0.938 | [0.792; 0.992] |
| Forster 2012 | 181 | 183 | 0.989 | [0.961; 0.999] |
| Hyun 2009 | 6 | 15 | 0.400 | [0.163; 0.677] |
| Kobayashi 2021 | 195 | 218 | 0.894 | [0.846; 0.932] |
| Kobayashi 2022 | 195 | 218 | 0.894 | [0.846; 0.932] |
| Kurokawa 2017 | 105 | 127 | 0.827 | [0.750; 0.888] |
| Muramoto_70% 2014 | 113 | 229 | 0.493 | [0.427; 0.560] |
| Muramoto_Presence_Absence 2014 | 213 | 229 | 0.930 | [0.889; 0.960] |
| Park_50% 2018 | 37 | 37 | 1.000 | [0.905; 1.000] |
| Park_AllorNone 2018 | 52 | 55 | 0.945 | [0.849; 0.989] |
| Quinones 2005 | 11 | 11 | 1.000 | [0.715; 1.000] |
| Ushirozako_EMSCT 2021 | 263 | 292 | 0.901 | [0.860; 0.932] |
| Ushirozako_IMSCT 2021 | 41 | 51 | 0.804 | [0.669; 0.902] |
| Velayutham 2016 | 272 | 277 | 0.982 | [0.958; 0.994] |
| Random effects model | | **2091** | **0.923** | **[0.848; 0.963]** |
| Heterogeneity: $I^2 = 95\%$, $\tau^2 = 1.9432$, $p < 0.01$ | | | | |
| **Pathology = Deformity** | | | | |
| Bhagat 2015 | 292 | 294 | 0.993 | [0.976; 0.999] |
| Feng 2012 | 158 | 160 | 0.988 | [0.956; 0.998] |
| Hsu 2008 | 157 | 162 | 0.969 | [0.929; 0.990] |
| Jarvis 2013 | 16 | 32 | 0.500 | [0.319; 0.681] |
| Krishnakumar 2011 | 47 | 47 | 1.000 | [0.925; 1.000] |
| Kundnani 2010 | 341 | 350 | 0.974 | [0.952; 0.988] |
| Langeloo Criteria 1 2003 | 106 | 116 | 0.914 | [0.847; 0.958] |
| Langeloo Criteria 2 2003 | 112 | 116 | 0.966 | [0.914; 0.991] |
| Langeloo Criteria 3 2003 | 110 | 116 | 0.948 | [0.891; 0.981] |
| Lubitz 1999 | 45 | 45 | 1.000 | [0.921; 1.000] |
| Luk 2001 | 23 | 23 | 1.000 | [0.852; 1.000] |
| Miller 2019 | 99 | 107 | 0.925 | [0.858; 0.967] |
| Neira 2016 | 220 | 222 | 0.991 | [0.968; 0.999] |
| Padberg 1996 | 66 | 71 | 0.930 | [0.843; 0.977] |
| Park 2011 | 21 | 25 | 0.840 | [0.639; 0.955] |
| Qiu 2022 | 81 | 82 | 0.988 | [0.934; 1.000] |
| Ushirozako 2018 | 248 | 265 | 0.936 | [0.899; 0.962] |
| Ushirozako 2020 2020 | 285 | 361 | 0.789 | [0.744; 0.830] |
| Yoshida 2022 | 261 | 297 | 0.879 | [0.836; 0.914] |
| Zhuang 2014 | 1137 | 1147 | 0.991 | [0.984; 0.996] |
| Zuccaro 2017 | 2362 | 2596 | 0.910 | [0.898; 0.921] |
| Random effects model | | **6634** | **0.961** | **[0.930; 0.979]** |
| Heterogeneity: $I^2 = 91\%$, $\tau^2 = 1.7329$, $p < 0.01$ | | | | |
| **Pathology = Various** | | | | |
| Bhalodia 2013 | 448 | 456 | 0.982 | [0.966; 0.992] |
| Bose 2007 | 202 | 226 | 0.894 | [0.846; 0.931] |
| Calancie 1998 | 22 | 22 | 1.000 | [0.846; 1.000] |
| Calancie 2008 | 808 | 810 | 0.998 | [0.991; 1.000] |
| Clark 2016 | 118 | 134 | 0.881 | [0.813; 0.930] |
| Funaba 2022 | 2343 | 2546 | 0.920 | [0.909; 0.930] |
| Ito_16 channel 2013 | 134 | 141 | 0.950 | [0.900; 0.980] |
| Ito_4 channel 2013 | 602 | 650 | 0.926 | [0.903; 0.945] |
| Ito_8 channel 2013 | 35 | 36 | 0.972 | [0.855; 0.999] |
| Ito_Grade1 2012 | 181 | 287 | 0.631 | [0.572; 0.687] |
| Ito_Grade2 2012 | 228 | 287 | 0.794 | [0.743; 0.840] |
| Ito_Grade3 2012 | 256 | 287 | 0.892 | [0.850; 0.925] |
| Kelleher 2008 | 25 | 26 | 0.962 | [0.804; 0.999] |
| Kim_AH 2012 | 328 | 329 | 0.997 | [0.983; 1.000] |
| Kim_TA 2012 | 243 | 243 | 1.000 | [0.985; 1.000] |
| Kobayashi 2019 | 105 | 128 | 0.820 | [0.743; 0.883] |
| Kobayashi 2021 | 3089 | 3450 | 0.895 | [0.885; 0.905] |
| Kobayashi 2014 | 786 | 864 | 0.910 | [0.889; 0.928] |
| Kobayashi_2018_>10%Latency 2018 | 62 | 74 | 0.838 | [0.734; 0.913] |
| Kobayashi_2018_>15%Latency 2018 | 71 | 74 | 0.959 | [0.886; 0.992] |
| Kobayashi_2018_>70%Amp 2018 | 53 | 74 | 0.716 | [0.599; 0.815] |
| Kobayashi_2018_>70%Amp&10%Latency 2018 | 69 | 74 | 0.932 | [0.849; 0.978] |
| Kobayashi_2018_>70%Amp&15%Latency 2018 | 72 | 74 | 0.973 | [0.906; 0.997] |
| Kobayashi_2018_Thoracic_>10%Latency 2018 | 61 | 63 | 0.968 | [0.890; 0.996] |
| Kobayashi_2018_Thoracic_>15%Latency 2018 | 55 | 63 | 0.873 | [0.765; 0.944] |
| Kobayashi_2018_Thoracic_>70%Amp 2018 | 45 | 63 | 0.714 | [0.587; 0.821] |
| Kobayashi_2018_Thoracic_>70%Amp&10%Latency 2018 | 61 | 62 | 0.984 | [0.913; 1.000] |
| Kobayashi_2018_Thoracic_>70%Amp&15%Latency 2018 | 55 | 63 | 0.873 | [0.765; 0.944] |
| Megha Bir_D−Wave_Immediate Postoperative 2021 | 9 | 9 | 1.000 | [0.664; 1.000] |
| Megha Bir_D−Wave_Long Term 2021 | 12 | 12 | 1.000 | [0.735; 1.000] |
| Megha Bir_TcMEP_Immediate Postoperative 2021 | 12 | 12 | 1.000 | [0.735; 1.000] |
| Megha Bir_TcMEP_Long Term 2021 | 11 | 18 | 0.611 | [0.357; 0.827] |
| Muramoto 2013 | 48 | 64 | 0.750 | [0.626; 0.850] |
| Sutter_cmEP 2019 | 2582 | 2600 | 0.993 | [0.989; 0.996] |
| Sutter_smEP 2019 | 1397 | 1399 | 0.999 | [0.995; 1.000] |
| Takahashi 2021 | 1621 | 1798 | 0.902 | [0.887; 0.915] |
| Traba_>65%Amp 2013 | 175 | 178 | 0.983 | [0.952; 0.997] |
| Traba_>70%Amp 2013 | 177 | 178 | 0.994 | [0.969; 1.000] |
| Traba_>70%Area 2013 | 178 | 178 | 1.000 | [0.979; 1.000] |
| Traba_>75%Amp 2013 | 178 | 178 | 1.000 | [0.979; 1.000] |
| Traba_>80%Amp 2013 | 178 | 178 | 1.000 | [0.979; 1.000] |
| Tsirikos 2020 | 1140 | 1145 | 0.996 | [0.990; 0.999] |
| Ushirozako 2019 | 541 | 639 | 0.847 | [0.816; 0.874] |
| Wang 2015 | 305 | 309 | 0.987 | [0.967; 0.996] |
| Wang_2 2016 | 16 | 18 | 0.889 | [0.653; 0.986] |
| Yang 1994 | 9 | 9 | 1.000 | [0.664; 1.000] |
| Yoshida 2018 | 746 | 746 | 1.000 | [0.995; 1.000] |
| Random effects model | | **21274** | **0.970** | **[0.946; 0.983]** |
| Heterogeneity: $I^2 = 93\%$, $\tau^2 = 3.5196$, $p < 0.01$ | | | | |
| **Pathology = Degenerative** | | | | |
| Chen Yu 2021 | 100 | 104 | 0.962 | [0.904; 0.989] |
| Clark_Persistent 2013 | 130 | 132 | 0.985 | [0.946; 0.998] |
| Clark_Temporary 2013 | 122 | 132 | 0.924 | [0.865; 0.963] |
| Cornips 2017 | 42 | 59 | 0.712 | [0.579; 0.822] |
| Fujiwara 2016 | 155 | 155 | 1.000 | [0.976; 1.000] |
| Funaba 2022 | 1049 | 1151 | 0.911 | [0.893; 0.927] |
| Funaba 2021 | 1049 | 1151 | 0.911 | [0.893; 0.927] |
| Hilibrand 2004 | 415 | 415 | 1.000 | [0.991; 1.000] |
| Kim 2021 | 111 | 118 | 0.941 | [0.882; 0.976] |
| Kim 2019 | 270 | 270 | 1.000 | [0.986; 1.000] |
| Kim 2007 | 46 | 51 | 0.902 | [0.786; 0.967] |
| Koffie 2022 | 86 | 117 | 0.735 | [0.645; 0.812] |
| Oya 2017 | 127 | 129 | 0.984 | [0.945; 0.998] |
| Sakaki_SpinalSegment1 2012 | 277 | 333 | 0.832 | [0.787; 0.870] |
| Sakaki_SpinalSegment2 2012 | 284 | 333 | 0.853 | [0.810; 0.889] |
| Sakaki_SpinalSegment3 2012 | 301 | 333 | 0.904 | [0.867; 0.933] |
| Sakaki_SpinalSegment4 2012 | 316 | 333 | 0.949 | [0.920; 0.970] |
| Sakaki_SpinalSegment5 2012 | 323 | 333 | 0.970 | [0.945; 0.986] |
| Sakaki_SpinalSegment6 2012 | 327 | 333 | 0.982 | [0.961; 0.993] |
| Sakaki_SpinalTract1 2012 | 222 | 335 | 0.663 | [0.609; 0.713] |
| Sakaki_SpinalTract2 2012 | 243 | 335 | 0.725 | [0.674; 0.772] |
| Sakaki_SpinalTract3 2012 | 263 | 335 | 0.785 | [0.737; 0.828] |
| Sakaki_SpinalTract4 2012 | 278 | 335 | 0.830 | [0.785; 0.869] |
| Sakaki_SpinalTract5 2012 | 296 | 335 | 0.884 | [0.844; 0.916] |
| Sakaki_SpinalTract6 2012 | 306 | 335 | 0.913 | [0.878; 0.941] |
| Tanaka_1_With_CMAP_Normalization 2015 | 187 | 192 | 0.974 | [0.940; 0.991] |
| Tanaka_2_Without_CMAP_Normalization 2015 | 186 | 192 | 0.969 | [0.933; 0.988] |
| Wilent 2019 | 4282 | 4372 | 0.979 | [0.975; 0.983] |
| Wilent 2020 | 40638 | 40870 | 0.994 | [0.994; 0.995] |
| Xu 2011 | 53 | 54 | 0.981 | [0.901; 1.000] |
| Random effects model | | **53672** | **0.949** | **[0.915; 0.970]** |
| Heterogeneity: $I^2 = 99\%$, $\tau^2 = 2.1743$, $p = 0$ | | | | |
| **Pathology = AVM** | | | | |
| Li_>50%Amp_Permanent 2018 | 23 | 31 | 0.742 | [0.554; 0.881] |
| Li_>50%Amp_Temporary 2018 | 18 | 31 | 0.581 | [0.391; 0.755] |
| Li_>80%Amp_Permanent 2018 | 27 | 31 | 0.871 | [0.702; 0.964] |
| Li_>80%Amp_Temporary 2018 | 22 | 31 | 0.710 | [0.520; 0.858] |
| Random effects model | | **124** | **0.731** | **[0.616; 0.822]** |
| Heterogeneity: $I^2 = 52\%$, $\tau^2 = 0.1180$, $p = 0.10$ | | | | |
| **Pathology = Neural Axis Abnormality** | | | | |
| Wang 2016 | 112 | 114 | 0.982 | [0.938; 0.998] |
| **Random effects model** | | **83909** | **0.956** | **[0.940; 0.967]** |
| Heterogeneity: $I^2 = 97\%$, $\tau^2 = 2.6633$, $p = 0$ | | | | |
| Test for subgroup differences: $\chi^2_5 = 52.49$, df = 5 ($p < 0.01$) | | | | |

For Peer Review

## Comparison of sROC for MEP by Different Pathology

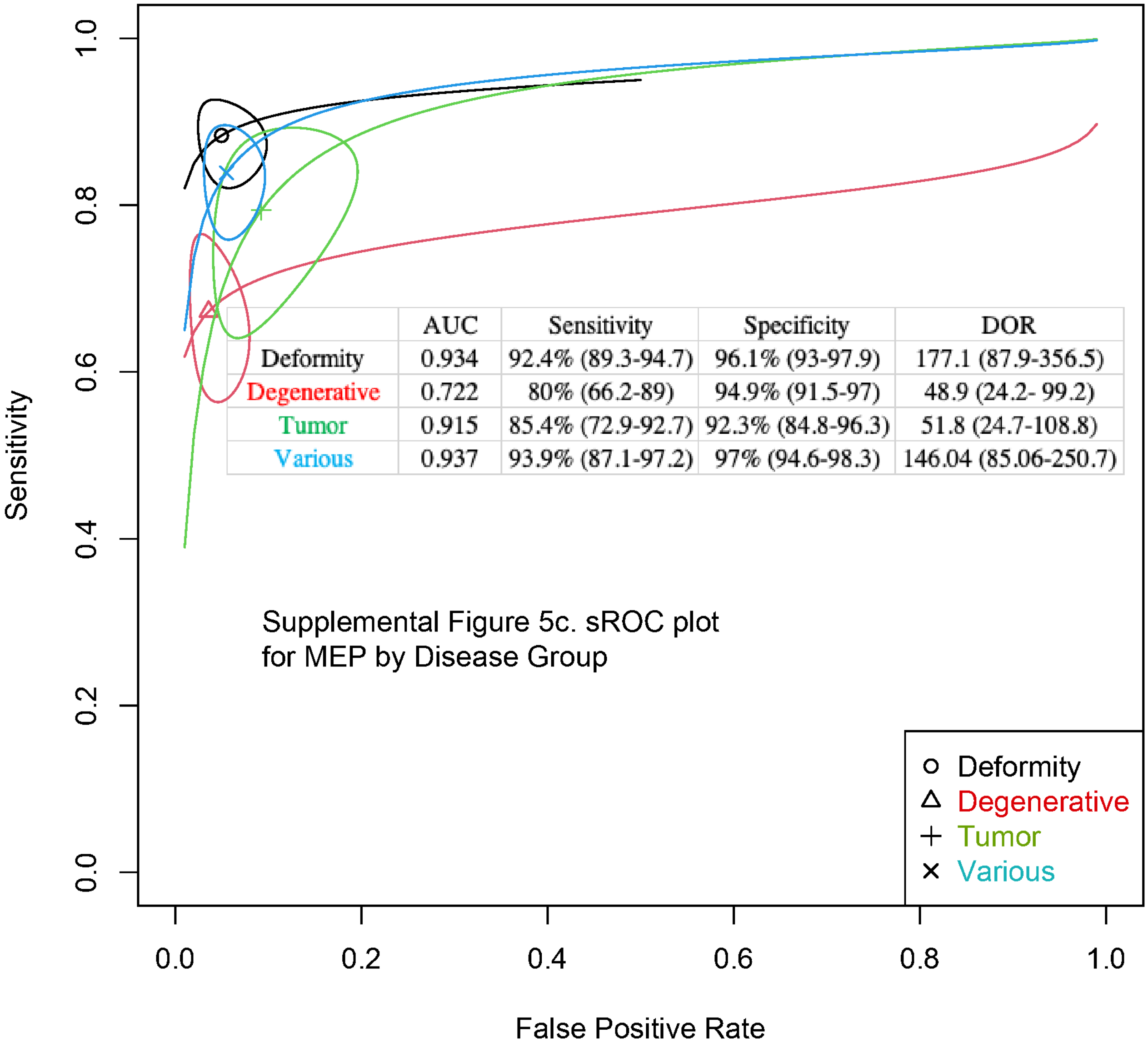


| | AUC | Sensitivity | Specificity | DOR |
|---|---|---|---|---|
| Deformity | 0.934 | 92.4% (89.3-94.7) | 96.1% (93-97.9) | 177.1 (87.9-356.5) |
| Degenerative | 0.722 | 80% (66.2-89) | 94.9% (91.5-97) | 48.9 (24.2- 99.2) |
| Tumor | 0.915 | 85.4% (72.9-92.7) | 92.3% (84.8-96.3) | 51.8 (24.7-108.8) |
| Various | 0.937 | 93.9% (87.1-97.2) | 97% (94.6-98.3) | 146.04 (85.06-250.7) |

Supplemental Figure 5c. sROC plot
for MEP by Disease Group

Supplemental Figure 6a. Forest plot for Sensitivity for MEP by Region of Surgery

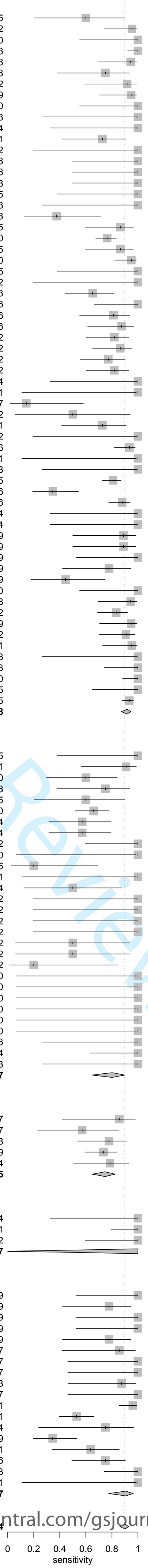


| Study | Events | Total | Proportion | 95%−CI |
|---|---|---|---|---|
| **Region = All** | | | | |
| Avila 2013 | 3 | 5 | 0.600 | [0.200; 0.900] |
| Bhagat 2015 | 21 | 22 | 0.955 | [0.739; 0.994] |
| Calancie 1998 | 10 | 10 | 1.000 | [0.552; 0.997] |
| Calancie 2008 | 93 | 93 | 1.000 | [0.921; 1.000] |
| Choi 2014 | 17 | 18 | 0.944 | [0.693; 0.992] |
| Clark_Persistent 2013 | 6 | 8 | 0.750 | [0.377; 0.937] |
| Feng 2012 | 11 | 12 | 0.917 | [0.587; 0.988] |
| Forster 2012 | 18 | 19 | 0.947 | [0.706; 0.993] |
| Hsu 2008 | 10 | 10 | 1.000 | [0.552; 0.997] |
| Hyun 2009 | 3 | 3 | 1.000 | [0.266; 0.993] |
| Ito_16 channel 2013 | 4 | 4 | 1.000 | [0.326; 0.994] |
| Ito_4 channel 2013 | 8 | 11 | 0.727 | [0.414; 0.910] |
| Ito_8 channel 2013 | 2 | 2 | 1.000 | [0.194; 0.990] |
| Ito_Grade1 2012 | 8 | 8 | 1.000 | [0.495; 0.997] |
| Ito_Grade2 2012 | 8 | 8 | 1.000 | [0.495; 0.997] |
| Ito_Grade3 2012 | 8 | 8 | 1.000 | [0.495; 0.997] |
| Jarvis 2013 | 5 | 5 | 1.000 | [0.378; 0.995] |
| Kim_AH 2012 | 3 | 3 | 1.000 | [0.266; 0.993] |
| Kim_TA 2012 | 3 | 8 | 0.375 | [0.125; 0.715] |
| Kobayashi 2021 | 13 | 15 | 0.867 | [0.595; 0.966] |
| Kobayashi 2021 | 84 | 110 | 0.764 | [0.675; 0.834] |
| Kobayashi 2022 | 13 | 15 | 0.867 | [0.595; 0.966] |
| Kobayashi 2014 | 38 | 40 | 0.950 | [0.821; 0.987] |
| Krishnakumar 2011 | 5 | 5 | 1.000 | [0.378; 0.995] |
| Kundnani 2010 | 2 | 2 | 1.000 | [0.194; 0.990] |
| Kurokawa 2017 | 15 | 23 | 0.652 | [0.443; 0.816] |
| Langeloo Criteria 1 2003 | 16 | 16 | 1.000 | [0.664; 0.998] |
| Langeloo Criteria 2 2003 | 13 | 16 | 0.812 | [0.553; 0.938] |
| Langeloo Criteria 3 2003 | 14 | 16 | 0.875 | [0.614; 0.969] |
| Li_>50%Amp_Permanent 2018 | 18 | 22 | 0.818 | [0.604; 0.930] |
| Li_>50%Amp_Temporary 2018 | 19 | 22 | 0.864 | [0.652; 0.955] |
| Li_>80%Amp_Permanent 2018 | 17 | 22 | 0.773 | [0.556; 0.902] |
| Li_>80%Amp_Temporary 2018 | 18 | 22 | 0.818 | [0.604; 0.930] |
| Lubitz 1999 | 4 | 4 | 1.000 | [0.326; 0.994] |
| Luk 2001 | 1 | 1 | 1.000 | [0.109; 0.987] |
| Megha Bir_D−Wave_Immediate Postoperative 2021 | 1 | 7 | 0.143 | [0.020; 0.581] |
| Megha Bir_D−Wave_Long Term 2021 | 1 | 2 | 0.500 | [0.059; 0.941] |
| Megha Bir_TcMEP_Immediate Postoperative 2021 | 8 | 11 | 0.727 | [0.414; 0.910] |
| Megha Bir_TcMEP_Long Term 2021 | 2 | 2 | 1.000 | [0.194; 0.990] |
| Neira 2016 | 43 | 46 | 0.935 | [0.816; 0.979] |
| Padberg 1996 | 1 | 1 | 1.000 | [0.109; 0.987] |
| Qiu 2022 | 3 | 3 | 1.000 | [0.266; 0.993] |
| Sutter_cmEP 2019 | 93 | 115 | 0.809 | [0.726; 0.871] |
| Sutter_smEP 2019 | 9 | 26 | 0.346 | [0.191; 0.543] |
| Takahashi 2021 | 58 | 66 | 0.879 | [0.776; 0.938] |
| Tanaka_1_With_CMAP_Normalization 2015 | 4 | 4 | 1.000 | [0.326; 0.994] |
| Tanaka_2_Without_CMAP_Normalization 2015 | 4 | 4 | 1.000 | [0.326; 0.994] |
| Traba_>65%Amp 2013 | 8 | 9 | 0.889 | [0.500; 0.985] |
| Traba_>70%Amp 2013 | 8 | 9 | 0.889 | [0.500; 0.985] |
| Traba_>70%Area 2013 | 9 | 9 | 1.000 | [0.525; 0.997] |
| Traba_>75%Amp 2013 | 7 | 9 | 0.778 | [0.421; 0.944] |
| Traba_>80%Amp 2013 | 4 | 9 | 0.444 | [0.177; 0.749] |
| Tsirikos 2020 | 10 | 10 | 1.000 | [0.552; 0.997] |
| Ushirozako 2018 | 17 | 18 | 0.944 | [0.693; 0.992] |
| Ushirozako 2019 | 35 | 42 | 0.833 | [0.690; 0.918] |
| Ushirozako 2020 2020 | 18 | 19 | 0.947 | [0.706; 0.993] |
| Ushirozako_EMSCT 2021 | 20 | 22 | 0.909 | [0.700; 0.977] |
| Ushirozako_IMSCT 2021 | 20 | 21 | 0.952 | [0.729; 0.993] |
| Wang 2016 | 3 | 3 | 1.000 | [0.266; 0.993] |
| Wang 2015 | 23 | 23 | 1.000 | [0.741; 0.999] |
| Yoshida 2018 | 60 | 60 | 1.000 | [0.882; 0.999] |
| Zhuang 2014 | 15 | 15 | 1.000 | [0.650; 0.998] |
| Zuccaro 2017 | 126 | 135 | 0.933 | [0.877; 0.965] |
| Random effects model | | **1308** | **0.916** | **[0.872; 0.946]** |
| Heterogeneity: $I^2 = 35\%$, $\tau^2 = 1.7039$, $p < 0.01$ | | | | |
| **Region = Cervical** | | | | |
| Bhalodia 2013 | 5 | 5 | 1.000 | [0.378; 0.995] |
| Bose 2007 | 10 | 11 | 0.909 | [0.561; 0.987] |
| Clark 2016 | 6 | 10 | 0.600 | [0.297; 0.842] |
| Clark_Temporary 2013 | 6 | 8 | 0.750 | [0.377; 0.937] |
| Fujiwara 2016 | 3 | 5 | 0.600 | [0.200; 0.900] |
| Funaba 2022 | 33 | 50 | 0.660 | [0.520; 0.777] |
| Funaba 2022 | 8 | 14 | 0.571 | [0.316; 0.794] |
| Funaba 2021 | 8 | 14 | 0.571 | [0.316; 0.794] |
| Hilibrand 2004 | 12 | 12 | 1.000 | [0.597; 0.998] |
| Kelleher 2008 | 0 | 0 | 1.000 | [0.063; 0.984] |
| Kim 2021 | 1 | 5 | 0.200 | [0.027; 0.691] |
| Kim 2007 | 1 | 1 | 1.000 | [0.109; 0.987] |
| Koffie 2022 | 2 | 4 | 0.500 | [0.123; 0.877] |
| Oya 2017 | 2 | 2 | 1.000 | [0.194; 0.990] |
| Sakaki_SpinalSegment1 2012 | 2 | 2 | 1.000 | [0.194; 0.990] |
| Sakaki_SpinalSegment2 2012 | 2 | 2 | 1.000 | [0.194; 0.990] |
| Sakaki_SpinalSegment3 2012 | 2 | 2 | 1.000 | [0.194; 0.990] |
| Sakaki_SpinalSegment4 2012 | 1 | 2 | 0.500 | [0.059; 0.941] |
| Sakaki_SpinalSegment5 2012 | 1 | 2 | 0.500 | [0.059; 0.941] |
| Sakaki_SpinalSegment6 2012 | 0 | 2 | 0.200 | [0.011; 0.847] |
| Sakaki_SpinalTract1 2012 | 0 | 0 | 1.000 | [0.063; 0.984] |
| Sakaki_SpinalTract2 2012 | 0 | 0 | 1.000 | [0.063; 0.984] |
| Sakaki_SpinalTract3 2012 | 0 | 0 | 1.000 | [0.063; 0.984] |
| Sakaki_SpinalTract4 2012 | 0 | 0 | 1.000 | [0.063; 0.984] |
| Sakaki_SpinalTract5 2012 | 0 | 0 | 1.000 | [0.063; 0.984] |
| Sakaki_SpinalTract6 2012 | 0 | 0 | 1.000 | [0.063; 0.984] |
| Wang_2 2016 | 3 | 3 | 1.000 | [0.266; 0.993] |
| Wilent 2019 | 14 | 14 | 1.000 | [0.634; 0.998] |
| Xu 2011 | 3 | 3 | 1.000 | [0.266; 0.993] |
| Random effects model | | **177** | **0.802** | **[0.647; 0.899]** |
| Heterogeneity: $I^2 = 0\%$, $\tau^2 = 1.2602$, $p = 1.00$ | | | | |
| **Region = Lumbosacral** | | | | |
| Chen Yu 2021 | 6 | 7 | 0.857 | [0.419; 0.980] |
| Kim 2019 | 4 | 7 | 0.571 | [0.230; 0.856] |
| Miller 2019 | 14 | 18 | 0.778 | [0.535; 0.914] |
| Wilent 2020 | 36 | 49 | 0.735 | [0.595; 0.839] |
| Yoshida 2022 | 11 | 14 | 0.786 | [0.506; 0.929] |
| Random effects model | | **95** | **0.747** | **[0.651; 0.825]** |
| Heterogeneity: $I^2 = 0\%$, $\tau^2 = 0$, $p = 0.78$ | | | | |
| **Region = Thoracic** | | | | |
| Cornips 2017 | 4 | 4 | 1.000 | [0.326; 0.994] |
| Kobayashi 2019 | 31 | 31 | 1.000 | [0.794; 0.999] |
| Muramoto 2013 | 12 | 12 | 1.000 | [0.597; 0.998] |
| Random effects model | | **47** | **1.000** | **[0.000; 1.000]** |
| Heterogeneity: $I^2 = 0\%$, $\tau^2 = 0$, $p = 1.00$ | | | | |
| **Region = Cervicothoracic** | | | | |
| Kobayashi_2018_>10%Latency 2018 | 9 | 9 | 1.000 | [0.525; 0.997] |
| Kobayashi_2018_>15%Latency 2018 | 7 | 9 | 0.778 | [0.421; 0.944] |
| Kobayashi_2018_>70%Amp 2018 | 9 | 9 | 1.000 | [0.525; 0.997] |
| Kobayashi_2018_>70%Amp&10%Latency 2018 | 9 | 9 | 1.000 | [0.525; 0.997] |
| Kobayashi_2018_>70%Amp&15%Latency 2018 | 7 | 9 | 0.778 | [0.421; 0.944] |
| Kobayashi_2018_Thoracic_>10%Latency 2018 | 6 | 7 | 0.857 | [0.419; 0.980] |
| Kobayashi_2018_Thoracic_>15%Latency 2018 | 7 | 7 | 1.000 | [0.461; 0.996] |
| Kobayashi_2018_Thoracic_>70%Amp 2018 | 7 | 7 | 1.000 | [0.461; 0.996] |
| Kobayashi_2018_Thoracic_>70%Amp&10%Latency 2018 | 7 | 8 | 0.875 | [0.463; 0.983] |
| Kobayashi_2018_Thoracic_>70%Amp&15%Latency 2018 | 7 | 7 | 1.000 | [0.461; 0.996] |
| Muramoto_70% 2014 | 49 | 51 | 0.961 | [0.856; 0.990] |
| Muramoto_Presence_Absence 2014 | 27 | 51 | 0.529 | [0.394; 0.661] |
| Park 2011 | 3 | 4 | 0.750 | [0.238; 0.966] |
| Park_50% 2018 | 10 | 29 | 0.345 | [0.197; 0.531] |
| Park_AllorNone 2018 | 7 | 11 | 0.636 | [0.339; 0.857] |
| Quinones 2005 | 12 | 16 | 0.750 | [0.492; 0.903] |
| Velayutham 2016 | 23 | 23 | 1.000 | [0.741; 0.999] |
| Yang 1994 | 1 | 1 | 1.000 | [0.109; 0.987] |
| Random effects model | | **267** | **0.906** | **[0.776; 0.964]** |
| Heterogeneity: $I^2 = 45\%$, $\tau^2 = 2.3763$, $p = 0.02$ | | | | |
| **Random effects model** | | **1894** | **0.900** | **[0.861; 0.929]** |
| Heterogeneity: $I^2 = 32\%$, $\tau^2 = 1.9159$, $p < 0.01$ | | | | |
| Test for subgroup differences: $\chi^2_4 = 16.78$, df = 4 ($p < 0.01$) | | | | |

For Peer Review

Supplemental Figure 6b. Forest plot for Specificity for MEP by Region of Surgery

| Study | Events | Total | Proportion | 95%-CI |
|---|---|---|---|---|
| **Region = All** | | | | |
| Avila 2013 | 114 | 117 | 0.974 | [0.927; 0.995] |
| Bhagat 2015 | 292 | 294 | 0.993 | [0.976; 0.999] |
| Calancie 1998 | 22 | 22 | 1.000 | [0.846; 1.000] |
| Calancie 2008 | 808 | 810 | 0.998 | [0.991; 1.000] |
| Choi 2014 | 30 | 32 | 0.938 | [0.792; 0.992] |
| Clark_Persistent 2013 | 130 | 132 | 0.985 | [0.946; 0.998] |
| Feng 2012 | 158 | 160 | 0.988 | [0.956; 0.998] |
| Forster 2012 | 181 | 183 | 0.989 | [0.961; 0.999] |
| Hsu 2008 | 157 | 162 | 0.969 | [0.929; 0.990] |
| Hyun 2009 | 6 | 15 | 0.400 | [0.163; 0.677] |
| Ito_16 channel 2013 | 134 | 141 | 0.950 | [0.900; 0.980] |
| Ito_4 channel 2013 | 602 | 650 | 0.926 | [0.903; 0.945] |
| Ito_8 channel 2013 | 35 | 36 | 0.972 | [0.855; 0.999] |
| Ito_Grade1 2012 | 181 | 287 | 0.631 | [0.572; 0.687] |
| Ito_Grade2 2012 | 228 | 287 | 0.794 | [0.743; 0.840] |
| Ito_Grade3 2012 | 256 | 287 | 0.892 | [0.850; 0.925] |
| Jarvis 2013 | 16 | 32 | 0.500 | [0.319; 0.681] |
| Kim_AH 2012 | 328 | 329 | 0.997 | [0.983; 1.000] |
| Kim_TA 2012 | 243 | 243 | 1.000 | [0.985; 1.000] |
| Kobayashi 2021 | 195 | 218 | 0.894 | [0.846; 0.932] |
| Kobayashi 2021 | 3089 | 3450 | 0.895 | [0.885; 0.905] |
| Kobayashi 2022 | 195 | 218 | 0.894 | [0.846; 0.932] |
| Kobayashi 2014 | 786 | 864 | 0.910 | [0.889; 0.928] |
| Krishnakumar 2011 | 47 | 47 | 1.000 | [0.925; 1.000] |
| Kundnani 2010 | 341 | 350 | 0.974 | [0.952; 0.988] |
| Kurokawa 2017 | 105 | 127 | 0.827 | [0.750; 0.888] |
| Langeloo Criteria 1 2003 | 106 | 116 | 0.914 | [0.847; 0.958] |
| Langeloo Criteria 2 2003 | 112 | 116 | 0.966 | [0.914; 0.991] |
| Langeloo Criteria 3 2003 | 110 | 116 | 0.948 | [0.891; 0.981] |
| Li_>50%Amp_Permanent 2018 | 23 | 31 | 0.742 | [0.554; 0.881] |
| Li_>50%Amp_Temporary 2018 | 18 | 31 | 0.581 | [0.391; 0.755] |
| Li_>80%Amp_Permanent 2018 | 27 | 31 | 0.871 | [0.702; 0.964] |
| Li_>80%Amp_Temporary 2018 | 22 | 31 | 0.710 | [0.520; 0.858] |
| Lubitz 1999 | 45 | 45 | 1.000 | [0.921; 1.000] |
| Luk 2001 | 23 | 23 | 1.000 | [0.852; 1.000] |
| Megha Bir_D−Wave_Immediate Postoperative 2021 | 9 | 9 | 1.000 | [0.664; 1.000] |
| Megha Bir_D−Wave_Long Term 2021 | 12 | 12 | 1.000 | [0.735; 1.000] |
| Megha Bir_TcMEP_Immediate Postoperative 2021 | 12 | 12 | 1.000 | [0.735; 1.000] |
| Megha Bir_TcMEP_Long Term 2021 | 11 | 18 | 0.611 | [0.357; 0.827] |
| Neira 2016 | 220 | 222 | 0.991 | [0.968; 0.999] |
| Padberg 1996 | 66 | 71 | 0.930 | [0.843; 0.977] |
| Qiu 2022 | 81 | 82 | 0.988 | [0.934; 1.000] |
| Sutter_cmEP 2019 | 2582 | 2600 | 0.993 | [0.989; 0.996] |
| Sutter_smEP 2019 | 1397 | 1399 | 0.999 | [0.995; 1.000] |
| Takahashi 2021 | 1621 | 1798 | 0.902 | [0.887; 0.915] |
| Tanaka_1_With_CMAP_Normalization 2015 | 187 | 192 | 0.974 | [0.940; 0.991] |
| Tanaka_2_Without_CMAP_Normalization 2015 | 186 | 192 | 0.969 | [0.933; 0.988] |
| Traba_>65%Amp 2013 | 175 | 178 | 0.983 | [0.952; 0.997] |
| Traba_>70%Amp 2013 | 177 | 178 | 0.994 | [0.969; 1.000] |
| Traba_>70%Area 2013 | 178 | 178 | 1.000 | [0.979; 1.000] |
| Traba_>75%Amp 2013 | 178 | 178 | 1.000 | [0.979; 1.000] |
| Traba_>80%Amp 2013 | 178 | 178 | 1.000 | [0.979; 1.000] |
| Tsirikos 2020 | 1140 | 1145 | 0.996 | [0.990; 0.999] |
| Ushirozako 2018 | 248 | 265 | 0.936 | [0.899; 0.962] |
| Ushirozako 2019 | 541 | 639 | 0.847 | [0.816; 0.874] |
| Ushirozako 2020 2020 | 285 | 361 | 0.789 | [0.744; 0.830] |
| Ushirozako_EMSCT 2021 | 263 | 292 | 0.901 | [0.860; 0.932] |
| Ushirozako_IMSCT 2021 | 41 | 51 | 0.804 | [0.669; 0.902] |
| Wang 2016 | 112 | 114 | 0.982 | [0.938; 0.998] |
| Wang 2015 | 305 | 309 | 0.987 | [0.967; 0.996] |
| Yoshida 2018 | 746 | 746 | 1.000 | [0.995; 1.000] |
| Zhuang 2014 | 1137 | 1147 | 0.991 | [0.984; 0.996] |
| Zuccaro 2017 | 2362 | 2596 | 0.910 | [0.898; 0.921] |
| **Random effects model** | | **25195** | **0.970** | **[0.951; 0.982]** |
| Heterogeneity: $I^2 = 93\%$, $\tau^2 = 3.4418$, $p < 0.01$ | | | | |
| **Region = Cervical** | | | | |
| Bhalodia 2013 | 448 | 456 | 0.982 | [0.966; 0.992] |
| Bose 2007 | 202 | 226 | 0.894 | [0.846; 0.931] |
| Clark 2016 | 118 | 134 | 0.881 | [0.813; 0.930] |
| Clark_Temporary 2013 | 122 | 132 | 0.924 | [0.865; 0.963] |
| Fujiwara 2016 | 155 | 155 | 1.000 | [0.976; 1.000] |
| Funaba 2022 | 2343 | 2546 | 0.920 | [0.909; 0.930] |
| Funaba 2022 | 1049 | 1151 | 0.911 | [0.893; 0.927] |
| Funaba 2021 | 1049 | 1151 | 0.911 | [0.893; 0.927] |
| Hilibrand 2004 | 415 | 415 | 1.000 | [0.991; 1.000] |
| Kelleher 2008 | 25 | 26 | 0.962 | [0.804; 0.999] |
| Kim 2021 | 111 | 118 | 0.941 | [0.882; 0.976] |
| Kim 2007 | 46 | 51 | 0.902 | [0.786; 0.967] |
| Koffie 2022 | 86 | 117 | 0.735 | [0.645; 0.812] |
| Oya 2017 | 127 | 129 | 0.984 | [0.945; 0.998] |
| Sakaki_SpinalSegment1 2012 | 277 | 333 | 0.832 | [0.787; 0.870] |
| Sakaki_SpinalSegment2 2012 | 284 | 333 | 0.853 | [0.810; 0.889] |
| Sakaki_SpinalSegment3 2012 | 301 | 333 | 0.904 | [0.867; 0.933] |
| Sakaki_SpinalSegment4 2012 | 316 | 333 | 0.949 | [0.920; 0.970] |
| Sakaki_SpinalSegment5 2012 | 323 | 333 | 0.970 | [0.945; 0.986] |
| Sakaki_SpinalSegment6 2012 | 327 | 333 | 0.982 | [0.961; 0.993] |
| Sakaki_SpinalTract1 2012 | 222 | 335 | 0.663 | [0.609; 0.713] |
| Sakaki_SpinalTract2 2012 | 243 | 335 | 0.725 | [0.674; 0.772] |
| Sakaki_SpinalTract3 2012 | 263 | 335 | 0.785 | [0.737; 0.828] |
| Sakaki_SpinalTract4 2012 | 278 | 335 | 0.830 | [0.785; 0.869] |
| Sakaki_SpinalTract5 2012 | 296 | 335 | 0.884 | [0.844; 0.916] |
| Sakaki_SpinalTract6 2012 | 306 | 335 | 0.913 | [0.878; 0.941] |
| Wang_2 2016 | 16 | 18 | 0.889 | [0.653; 0.986] |
| Wilent 2019 | 4282 | 4372 | 0.979 | [0.975; 0.983] |
| Xu 2011 | 53 | 54 | 0.981 | [0.901; 1.000] |
| **Random effects model** | | **15259** | **0.933** | **[0.898; 0.956]** |
| Heterogeneity: $I^2 = 96\%$, $\tau^2 = 1.3814$, $p < 0.01$ | | | | |
| **Region = Lumbosacral** | | | | |
| Chen Yu 2021 | 100 | 104 | 0.962 | [0.904; 0.989] |
| Kim 2019 | 270 | 270 | 1.000 | [0.986; 1.000] |
| Miller 2019 | 99 | 107 | 0.925 | [0.858; 0.967] |
| Wilent 2020 | 40638 | 40870 | 0.994 | [0.994; 0.995] |
| Yoshida 2022 | 261 | 297 | 0.879 | [0.836; 0.914] |
| **Random effects model** | | **41648** | **0.980** | **[0.911; 0.996]** |
| Heterogeneity: $I^2 = 99\%$, $\tau^2 = 2.7207$, $p < 0.01$ | | | | |
| **Region = Thoracic** | | | | |
| Cornips 2017 | 42 | 59 | 0.712 | [0.579; 0.822] |
| Kobayashi 2019 | 105 | 128 | 0.820 | [0.743; 0.883] |
| Muramoto 2013 | 48 | 64 | 0.750 | [0.626; 0.850] |
| **Random effects model** | | **251** | **0.776** | **[0.712; 0.828]** |
| Heterogeneity: $I^2 = 35\%$, $\tau^2 = 0.0075$, $p = 0.22$ | | | | |
| **Region = Cervicothoracic** | | | | |
| Kobayashi_2018_>10%Latency 2018 | 62 | 74 | 0.838 | [0.734; 0.913] |
| Kobayashi_2018_>15%Latency 2018 | 71 | 74 | 0.959 | [0.886; 0.992] |
| Kobayashi_2018_>70%Amp 2018 | 53 | 74 | 0.716 | [0.599; 0.815] |
| Kobayashi_2018_>70%Amp&10%Latency 2018 | 69 | 74 | 0.932 | [0.849; 0.978] |
| Kobayashi_2018_>70%Amp&15%Latency 2018 | 72 | 74 | 0.973 | [0.906; 0.997] |
| Kobayashi_2018_Thoracic_>10%Latency 2018 | 61 | 63 | 0.968 | [0.890; 0.996] |
| Kobayashi_2018_Thoracic_>15%Latency 2018 | 55 | 63 | 0.873 | [0.765; 0.944] |
| Kobayashi_2018_Thoracic_>70%Amp 2018 | 45 | 63 | 0.714 | [0.587; 0.821] |
| Kobayashi_2018_Thoracic_>70%Amp&10%Latency 2018 | 61 | 62 | 0.984 | [0.913; 1.000] |
| Kobayashi_2018_Thoracic_>70%Amp&15%Latency 2018 | 55 | 63 | 0.873 | [0.765; 0.944] |
| Muramoto_70% 2014 | 113 | 229 | 0.493 | [0.427; 0.560] |
| Muramoto_Presence_Absence 2014 | 213 | 229 | 0.930 | [0.889; 0.960] |
| Park 2011 | 21 | 25 | 0.840 | [0.639; 0.955] |
| Park_50% 2018 | 37 | 37 | 1.000 | [0.905; 1.000] |
| Park_AllorNone 2018 | 52 | 55 | 0.945 | [0.849; 0.989] |
| Quinones 2005 | 11 | 11 | 1.000 | [0.715; 1.000] |
| Velayutham 2016 | 272 | 277 | 0.982 | [0.958; 0.994] |
| Yang 1994 | 9 | 9 | 1.000 | [0.664; 1.000] |
| **Random effects model** | | **1556** | **0.928** | **[0.871; 0.961]** |
| Heterogeneity: $I^2 = 92\%$, $\tau^2 = 1.4707$, $p < 0.01$ | | | | |
| **Random effects model** | | **83909** | **0.956** | **[0.940; 0.967]** |

Heterogeneity: $I^2 = 97\%$, $\tau^2 = 2.6633$, $p = 0$
Test for subgroup differences: $\chi^2_4 = 65.39$, df = 4 ($p < 0.01$)

specificity (axis: 0.2, 0.4, 0.6, 0.8, 1)

## Comparison of sROC for MEP by Different Region

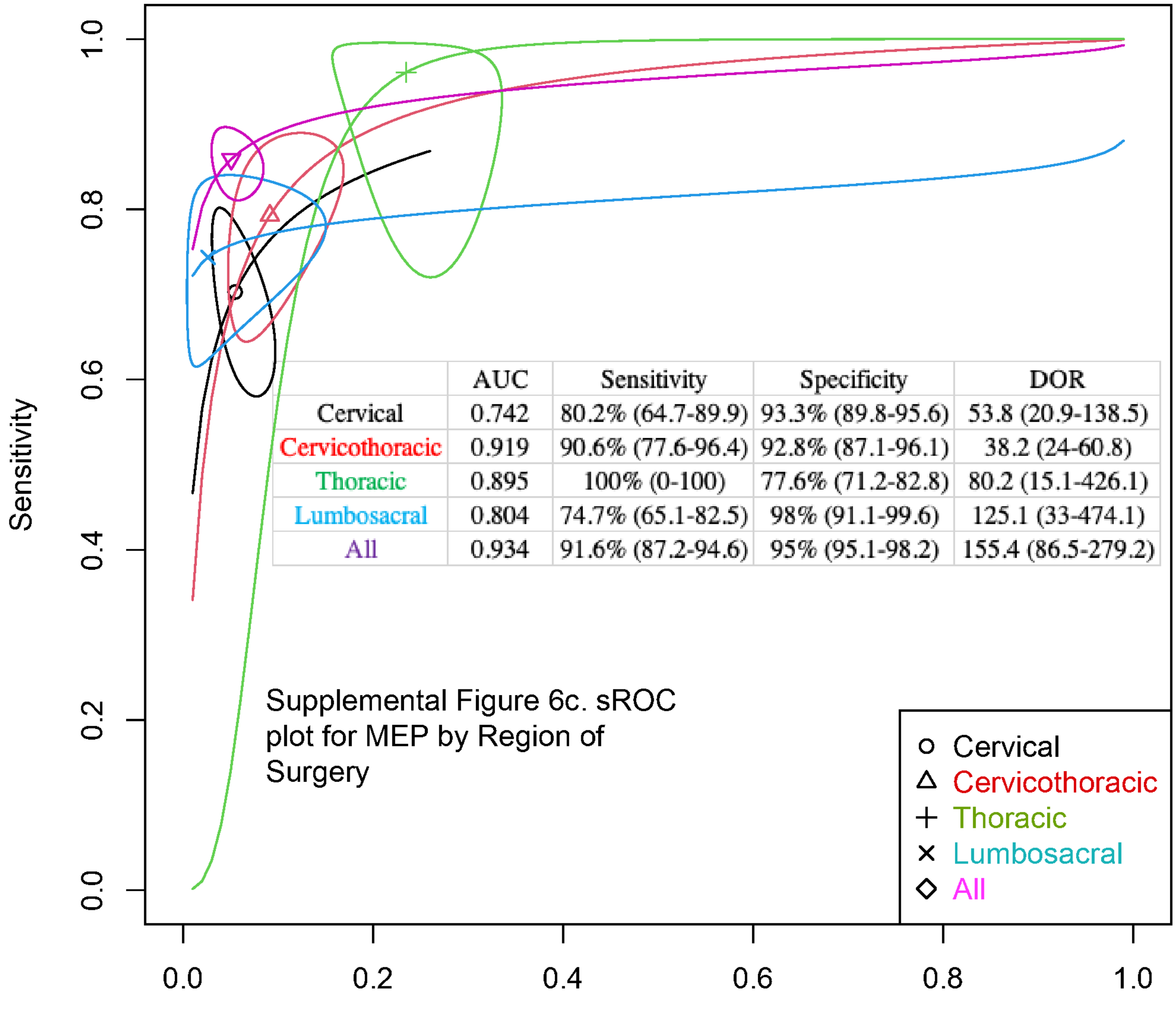


| | AUC | Sensitivity | Specificity | DOR |
|---|---|---|---|---|
| Cervical | 0.742 | 80.2% (64.7-89.9) | 93.3% (89.8-95.6) | 53.8 (20.9-138.5) |
| Cervicothoracic | 0.919 | 90.6% (77.6-96.4) | 92.8% (87.1-96.1) | 38.2 (24-60.8) |
| Thoracic | 0.895 | 100% (0-100) | 77.6% (71.2-82.8) | 80.2 (15.1-426.1) |
| Lumbosacral | 0.804 | 74.7% (65.1-82.5) | 98% (91.1-99.6) | 125.1 (33-474.1) |
| All | 0.934 | 91.6% (87.2-94.6) | 95% (95.1-98.2) | 155.4 (86.5-279.2) |

Supplemental Figure 6c. sROC plot for MEP by Region of Surgery

**Supplemental Figure 7a. Forest plot for Sensitivity for EMG by EMG Type**

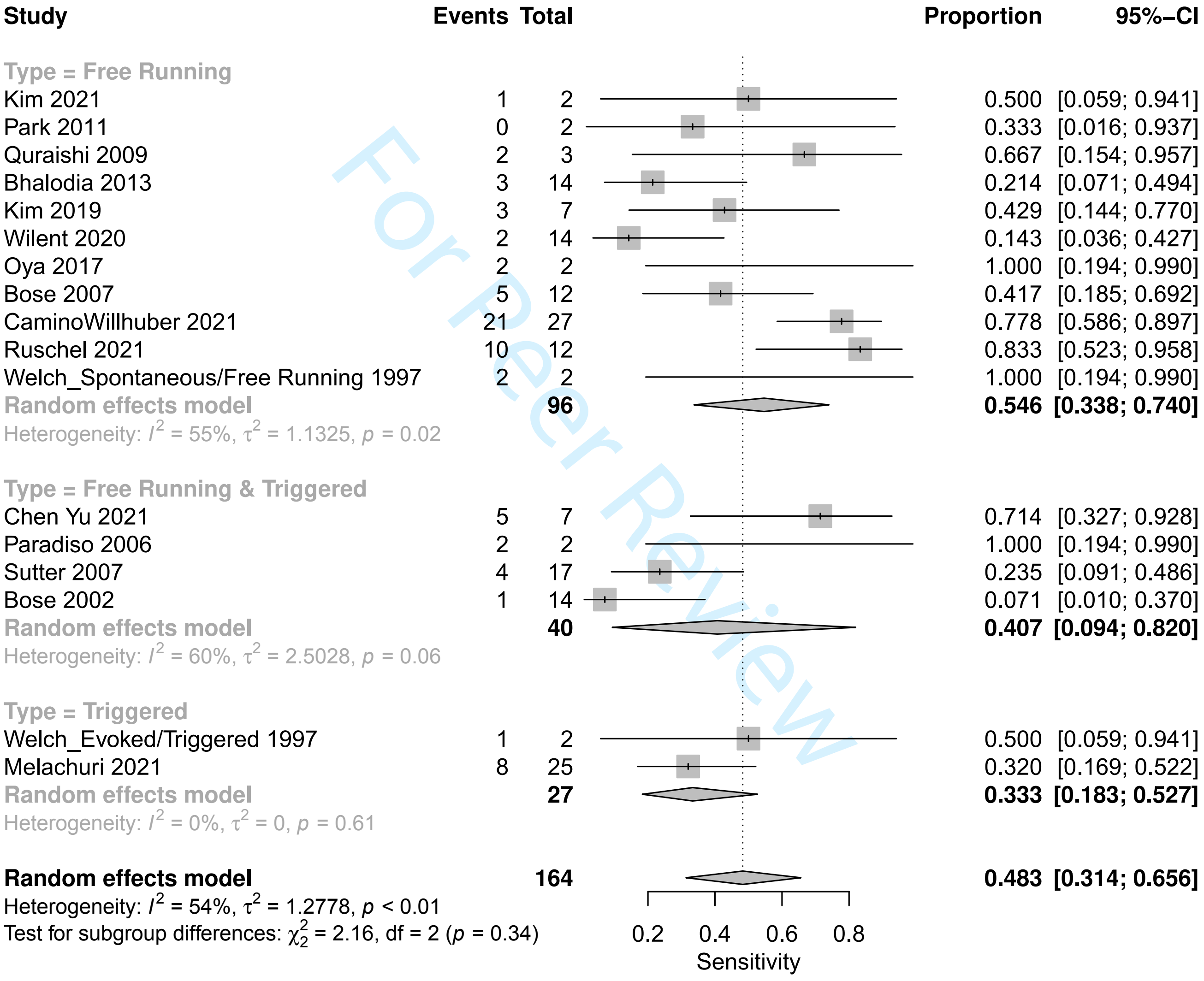


For Peer Review

**Supplemental Figure 7b. Forest plot for Specificity for EMG by EMG Type**

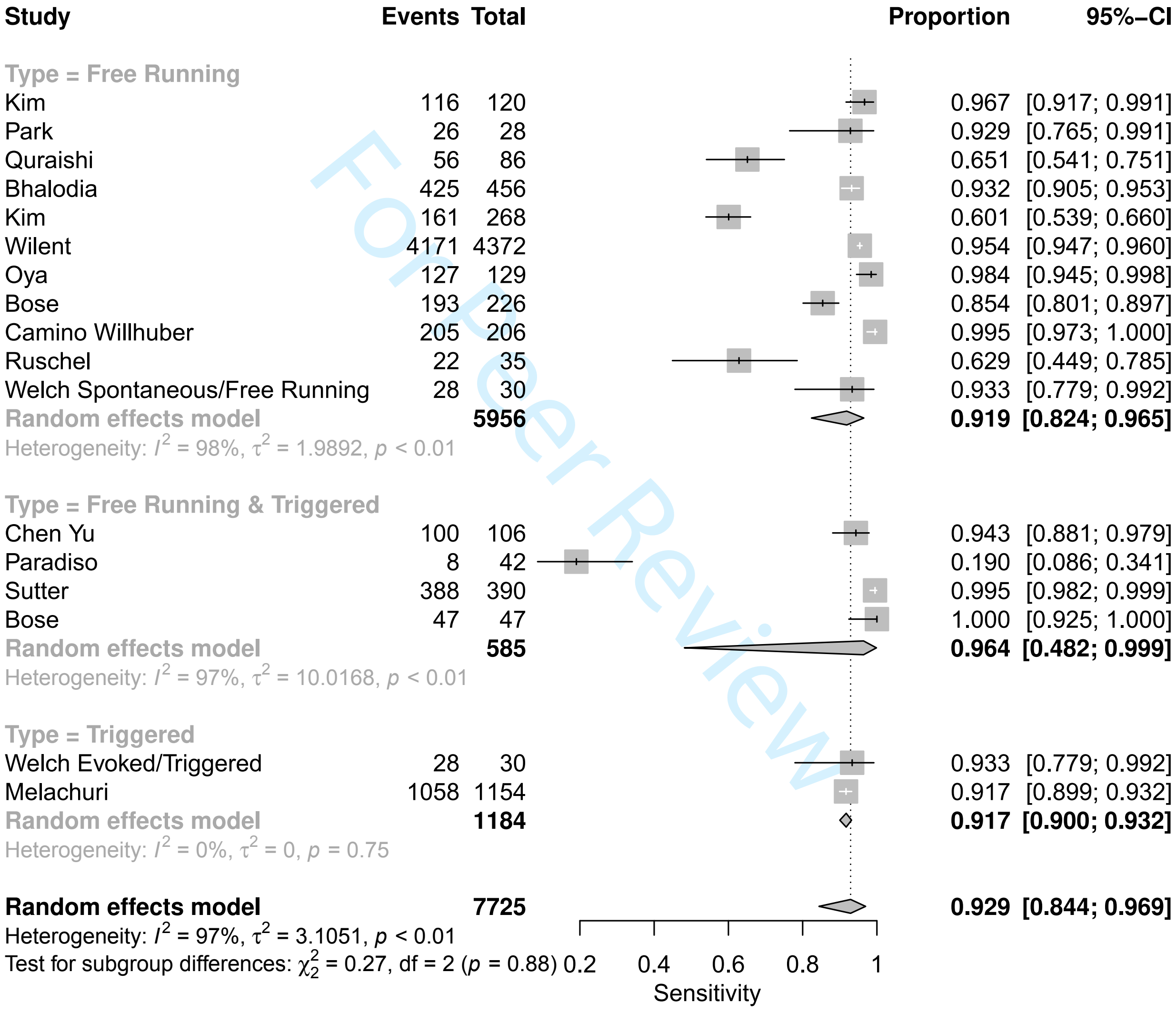


For Peer Review

## Comparison of sROC for EMG by Different Type

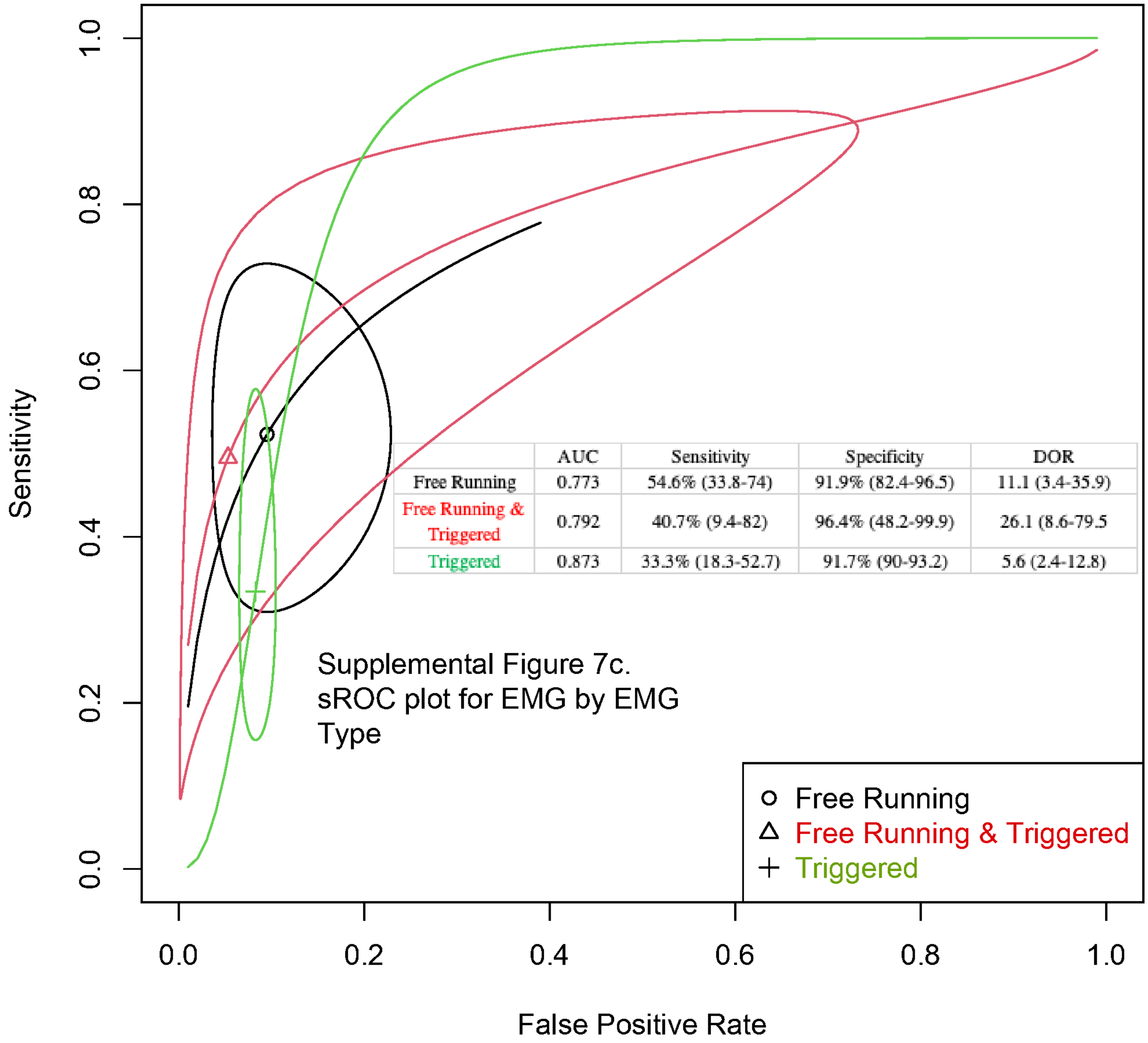


| | AUC | Sensitivity | Specificity | DOR |
|---|---|---|---|---|
| Free Running | 0.773 | 54.6% (33.8-74) | 91.9% (82.4-96.5) | 11.1 (3.4-35.9) |
| Free Running & Triggered | 0.792 | 40.7% (9.4-82) | 96.4% (48.2-99.9) | 26.1 (8.6-79.5 |
| Triggered | 0.873 | 33.3% (18.3-52.7) | 91.7% (90-93.2) | 5.6 (2.4-12.8) |

Supplemental Figure 7c. sROC plot for EMG by EMG Type

**Supplemental Figure 8a. Forest plot for Sensitivity for EMG by Disease Group**

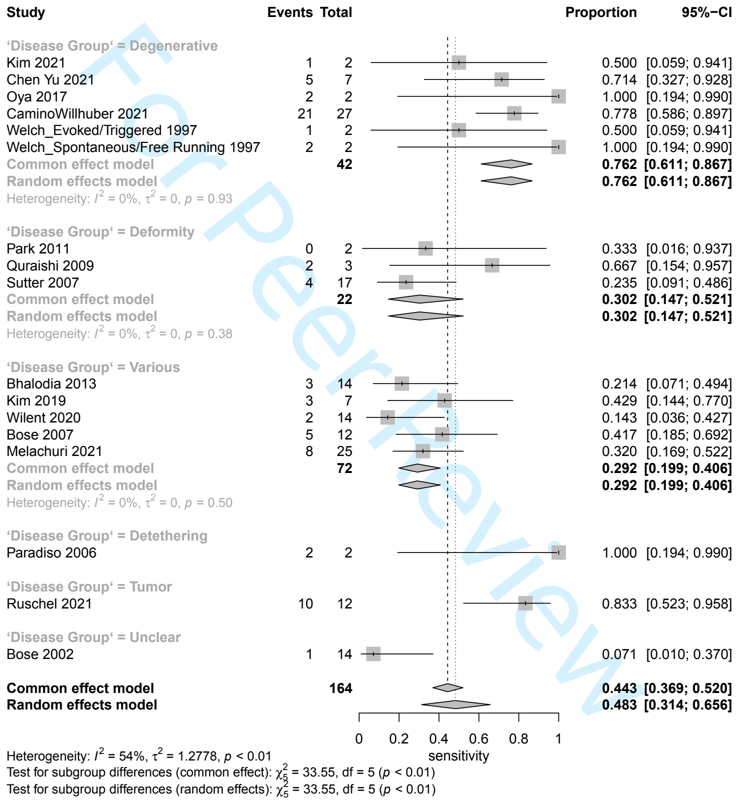


For Peer Review

**Supplemental Figure 8b. Forest plot for Specificity for EMG by Disease Group**

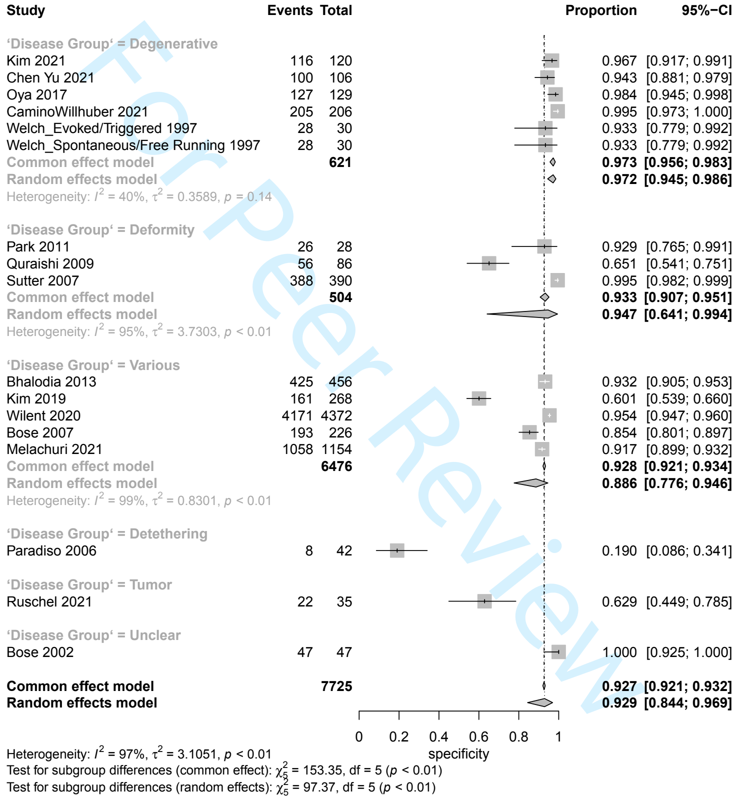


For Peer Review

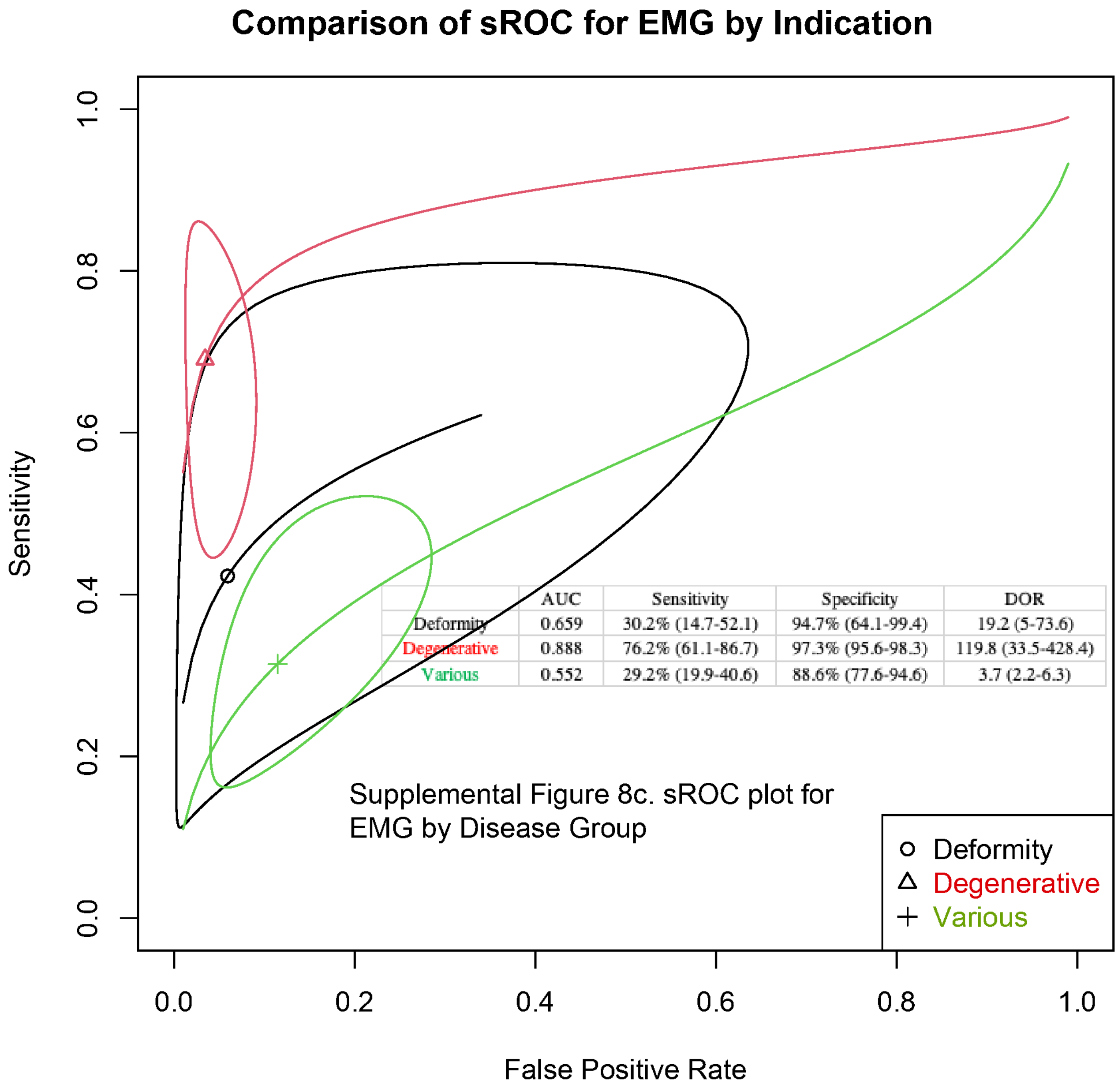


Supplemental Figure 8c. sROC plot for EMG by Disease Group

**Supplemental Figure 9a. Forest plot for Sensitivity for EMG by Region of Surgery**

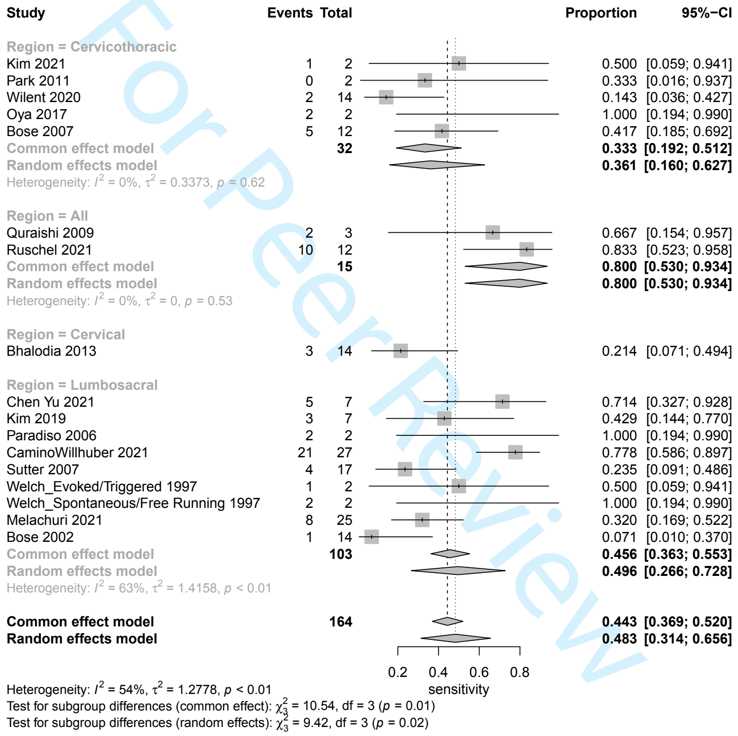


For Peer Review

**Supplemental Figure 9b. Forest plot for Specificity for EMG by Region of Surgery**

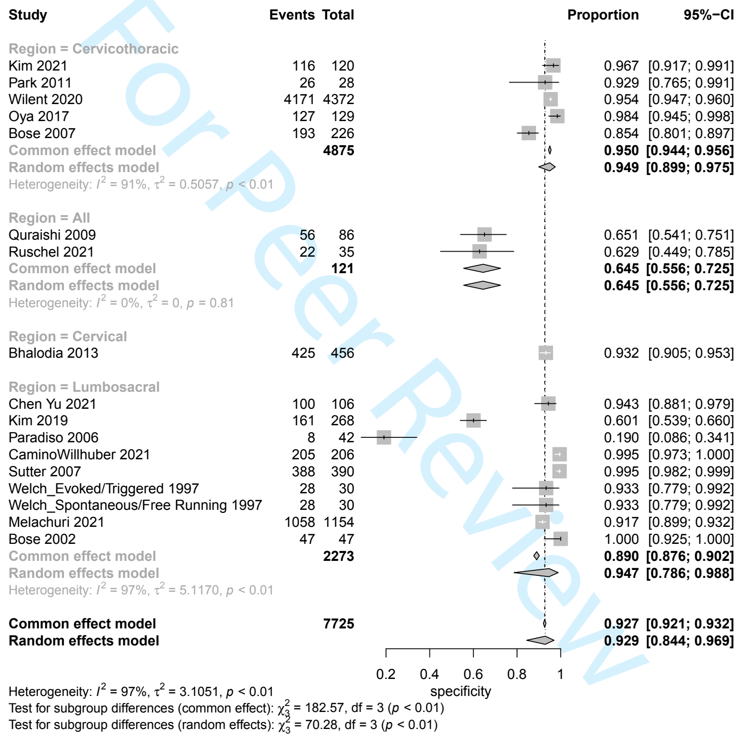


For Peer Review

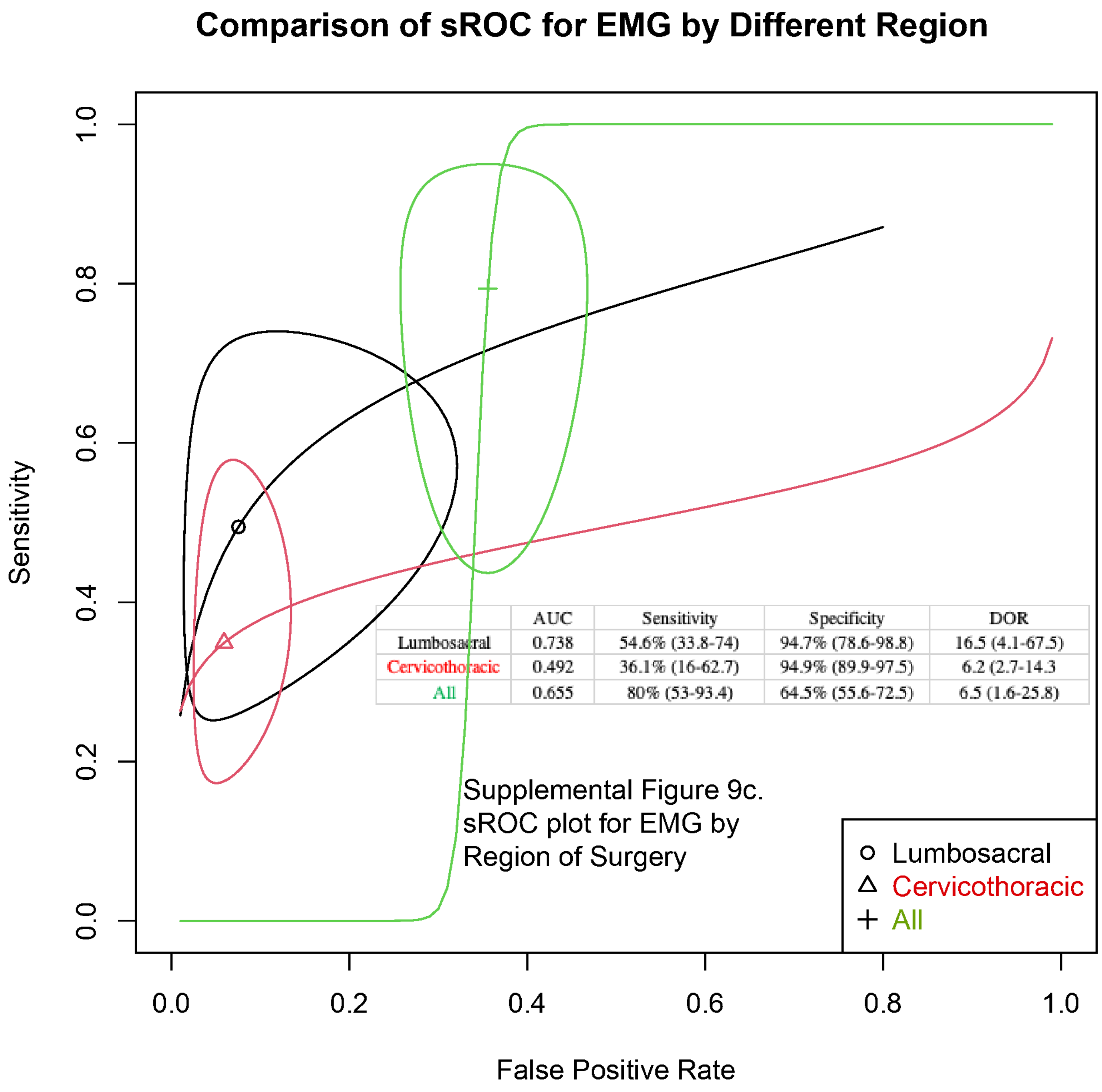


Supplemental Figure 9c.
sROC plot for EMG by
Region of Surgery

**Supplemental Figure 10a. Forest plot for Sensitivity for Multimodal by Type of Multimodal Monitoring**

| Study | Events | Total | Proportion | 95%-CI |
|---|---|---|---|---|
| **Group = SSEP and MEP** | | | | |
| Accadbled 2006 | 5 | 5 | 1.000 | [0.378; 0.995] |
| Almahdy 2021 | 10 | 10 | 1.000 | [0.552; 0.997] |
| Chen 2015 | 51 | 51 | 1.000 | [0.864; 0.999] |
| Costa 2007 | 3 | 3 | 1.000 | [0.266; 0.993] |
| Dicindio 2003 | 1 | 1 | 1.000 | [0.109; 0.987] |
| Hawary 2006 | 2 | 2 | 1.000 | [0.194; 0.990] |
| Hyun 2009 | 3 | 3 | 1.000 | [0.266; 0.993] |
| Kim 2014 | 6 | 7 | 0.857 | [0.419; 0.980] |
| Kim 2017 | 4 | 5 | 0.800 | [0.309; 0.973] |
| Kim 2019 | 4 | 7 | 0.571 | [0.230; 0.856] |
| Kim 2019 | 3 | 7 | 0.429 | [0.144; 0.770] |
| Kim 2021 | 2 | 5 | 0.400 | [0.100; 0.800] |
| Krishnakumar 2017 | 5 | 5 | 1.000 | [0.378; 0.995] |
| Kumar 2019 | 13 | 14 | 0.929 | [0.630; 0.990] |
| Lakomkin 2017 | 7 | 12 | 0.583 | [0.308; 0.815] |
| Lau 2022 | 4 | 4 | 1.000 | [0.326; 0.994] |
| Lau 2022 | 2 | 7 | 0.286 | [0.072; 0.673] |
| Lee 2016 | 5 | 6 | 0.833 | [0.369; 0.977] |
| Li 2012 | 8 | 8 | 1.000 | [0.495; 0.997] |
| Liu 2022 | 18 | 27 | 0.667 | [0.473; 0.817] |
| Neira 2016 | 47 | 51 | 0.922 | [0.809; 0.970] |
| Noonan 2002 | 6 | 6 | 1.000 | [0.423; 0.996] |
| Padberg 1998 | 2 | 2 | 1.000 | [0.194; 0.990] |
| Pastorelli 2011 | 9 | 10 | 0.900 | [0.533; 0.986] |
| Pastorelli 2015 | 3 | 3 | 1.000 | [0.266; 0.993] |
| Pelosi 2002 | 6 | 6 | 1.000 | [0.423; 0.996] |
| Schwartz 2007 | 38 | 38 | 1.000 | [0.825; 0.999] |
| Skaggs 2009 | 1 | 4 | 0.250 | [0.034; 0.762] |
| Taylor 2021 | 0 | 0 | 1.000 | [0.063; 0.984] |
| Tiruchelvan 2013 | 2 | 2 | 1.000 | [0.194; 0.990] |
| Tsirikos 2020 | 10 | 10 | 1.000 | [0.552; 0.997] |
| Van der Wal 2021 | 8 | 11 | 0.727 | [0.414; 0.910] |
| Vitale 2010 | 12 | 12 | 1.000 | [0.597; 0.998] |
| Common effect model | | **344** | **0.872** | **[0.833; 0.904]** |
| Random effects model | | | **0.935** | **[0.831; 0.977]** |
| Heterogeneity: $I^2 = 0\%$, $\tau^2 = 3.8326$, $p = 0.65$ | | | | |
| **Group = SSEP, MEP and EMG** | | | | |
| Bhagat 2015 | 23 | 23 | 1.000 | [0.741; 0.999] |
| Bhalodia (Acute Deficit) 2013 | 5 | 5 | 1.000 | [0.378; 0.995] |
| Bhalodia (Delayed) 2013 | 1 | 9 | 0.111 | [0.015; 0.500] |
| Bose 2007 | 11 | 12 | 0.917 | [0.587; 0.988] |
| Chen Yu 2021 | 7 | 7 | 1.000 | [0.461; 0.996] |
| Delgado−Lo_pez 2022 | 1 | 2 | 0.500 | [0.059; 0.941] |
| Duncan 2012 | 5 | 5 | 1.000 | [0.378; 0.995] |
| Eggspuehler (Cervical) 2007 | 10 | 12 | 0.833 | [0.523; 0.958] |
| Eggspuehler (Deformity) 2007 | 12 | 13 | 0.923 | [0.609; 0.989] |
| Eggspuehler (Thoracic) 2007 | 3 | 4 | 0.750 | [0.238; 0.966] |
| Ferguson 2014 | 4 | 4 | 1.000 | [0.326; 0.994] |
| Harel 2017 | 3 | 4 | 0.750 | [0.238; 0.966] |
| Ishida 2019 | 14 | 17 | 0.824 | [0.573; 0.942] |
| Jin (>50%) 2015 | 4 | 4 | 1.000 | [0.326; 0.994] |
| Jin (All or none) 2015 | 2 | 4 | 0.500 | [0.123; 0.877] |
| Kamerlink 2010 | 13 | 13 | 1.000 | [0.616; 0.998] |
| Kim 2019 | 4 | 7 | 0.571 | [0.230; 0.856] |
| Kobayashi 2017 | 3 | 3 | 1.000 | [0.266; 0.993] |
| MacDonald 2007 | 7 | 10 | 0.700 | [0.376; 0.900] |
| Platabello 2015 | 1 | 1 | 1.000 | [0.109; 0.987] |
| Quraishi 2009 | 5 | 10 | 0.500 | [0.225; 0.775] |
| Schar 2017 | 5 | 5 | 1.000 | [0.378; 0.995] |
| Sebastian (Day of Discharge Nerve Root Deficit) 2021 | 15 | 20 | 0.750 | [0.522; 0.892] |
| Sebastian (Day of Discharge) McCormick 2021 | 13 | 15 | 0.867 | [0.595; 0.966] |
| Sebastian (Follow Up McCormick) 2021 | 6 | 7 | 0.857 | [0.419; 0.980] |
| Sebastian (Permanent Deficit) 2021 | 8 | 9 | 0.889 | [0.500; 0.985] |
| Sebastian (Postop Nerve Root Deficit) 2021 | 19 | 25 | 0.760 | [0.558; 0.888] |
| Sutter 2019 − 1 | 107 | 115 | 0.930 | [0.867; 0.965] |
| Sutter I 2007 | 24 | 26 | 0.923 | [0.739; 0.981] |
| Sutter II 2007 | 18 | 20 | 0.900 | [0.676; 0.975] |
| Tong Yu 2017 | 1 | 1 | 1.000 | [0.109; 0.987] |
| Wilent 2019 | 7 | 7 | 1.000 | [0.461; 0.996] |
| Yu 2018 | 1 | 1 | 1.000 | [0.109; 0.987] |
| Common effect model | | **420** | **0.862** | **[0.825; 0.892]** |
| Random effects model | | | **0.877** | **[0.800; 0.927]** |
| Heterogeneity: $I^2 = 18\%$, $\tau^2 = 1.2172$, $p = 0.19$ | | | | |
| **Group = MEP and EMG** | | | | |
| Chen Yu 2021 | 6 | 7 | 0.857 | [0.419; 0.980] |
| Kim 2019 | 4 | 7 | 0.571 | [0.230; 0.856] |
| Lieberman 2008 | 10 | 10 | 1.000 | [0.552; 0.997] |
| Skinner 2005 | 8 | 8 | 1.000 | [0.495; 0.997] |
| Common effect model | | **32** | **0.875** | **[0.711; 0.952]** |
| Random effects model | | | **0.923** | **[0.538; 0.992]** |
| Heterogeneity: $I^2 = 0\%$, $\tau^2 = 2.0606$, $p = 0.73$ | | | | |
| **Group = SSEP, MEP and D−Wave** | | | | |
| Costa 2013 | 11 | 11 | 1.000 | [0.575; 0.997] |
| Dauleac 2022 | 11 | 30 | 0.367 | [0.216; 0.549] |
| Forster 2012 | 21 | 21 | 1.000 | [0.723; 0.999] |
| Ghadirpour 2015 | 3 | 3 | 1.000 | [0.266; 0.993] |
| Ghadirpour 2019 | 6 | 7 | 0.857 | [0.419; 0.980] |
| Korn ("Permanent" Change) 2015 | 18 | 22 | 0.818 | [0.604; 0.930] |
| Korn ("Reversible" Change) 2015 | 19 | 23 | 0.826 | [0.618; 0.933] |
| Common effect model | | **117** | **0.761** | **[0.675; 0.829]** |
| Random effects model | | | **0.902** | **[0.635; 0.980]** |
| Heterogeneity: $I^2 = 63\%$, $\tau^2 = 2.6791$, $p = 0.01$ | | | | |
| **Group = SSEP and D−Wave** | | | | |
| Gavaret 2011 | 34 | 34 | 1.000 | [0.809; 0.999] |
| **Group = SSEP and EMG** | | | | |
| Gunnarsson 2004 | 14 | 14 | 1.000 | [0.634; 0.998] |
| Melachuri 2021 | 9 | 25 | 0.360 | [0.199; 0.560] |
| Common effect model | | **39** | **0.590** | **[0.432; 0.731]** |
| Random effects model | | | **0.907** | **[0.036; 1.000]** |
| Heterogeneity: $I^2 = 0\%$, $\tau^2 = 9.9176$, $p = 1.00$ | | | | |
| **Group = MEP and D−Wave** | | | | |
| Hsu 2008 | 10 | 10 | 1.000 | [0.552; 0.997] |
| Sutter 2019 − 2 | 105 | 115 | 0.913 | [0.846; 0.953] |
| Sutter 2019 − 3 | 102 | 115 | 0.887 | [0.815; 0.933] |
| Common effect model | | **240** | **0.904** | **[0.860; 0.935]** |
| Random effects model | | | **0.904** | **[0.860; 0.935]** |
| Heterogeneity: $I^2 = 0\%$, $\tau^2 = 0$, $p = 0.81$ | | | | |
| **Common effect model** | | **1226** | **0.859** | **[0.838; 0.877]** |
| **Random effects model** | | | **0.910** | **[0.860; 0.943]** |

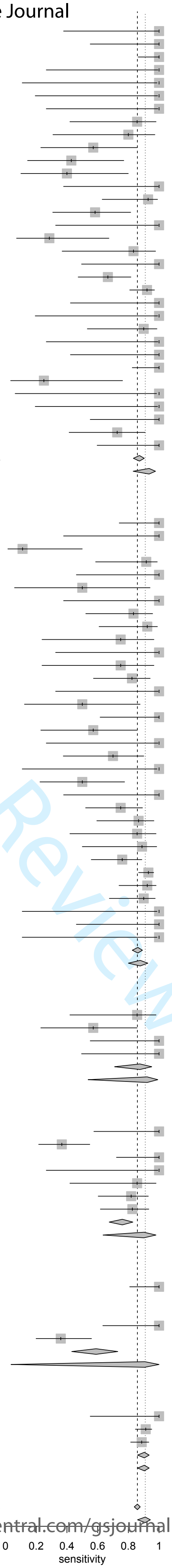


Heterogeneity: $I^2 = 40\%$, $\tau^2 = 2.4511$, $p < 0.01$
Test for subgroup differences (common effect): $\chi^2_6 = 32.21$, df = 6 ($p < 0.01$)
Test for subgroup differences (random effects): $\chi^2_6 = 1.46$, df = 6 ($p = 0.96$)

For Peer Review

Supplemental Figure 10b. Forest plot for Specificity for Multimodal by Type of Multimodal Monitoring

| Study | Events | Total | Proportion | 95%−CI |
|---|---|---|---|---|
| **Group = SSEP and MEP** | | | | |
| Accadbled 2006 | 96 | 182 | 0.527 | [0.452; 0.602] |
| Almahdy 2021 | 4 | 10 | 0.400 | [0.122; 0.738] |
| Chen 2015 | 362 | 375 | 0.965 | [0.941; 0.981] |
| Costa 2007 | 45 | 47 | 0.957 | [0.855; 0.995] |
| Dicindio 2003 | 68 | 72 | 0.944 | [0.864; 0.985] |
| Hawary 2006 | 50 | 52 | 0.962 | [0.868; 0.995] |
| Hyun 2009 | 4 | 14 | 0.286 | [0.084; 0.581] |
| Kim 2014 | 9 | 9 | 1.000 | [0.664; 1.000] |
| Kim 2017 | 164 | 169 | 0.970 | [0.932; 0.990] |
| Kim 2019 | 56 | 59 | 0.949 | [0.859; 0.989] |
| Kim 2019 | 161 | 268 | 0.601 | [0.539; 0.660] |
| Kim 2021 | 104 | 111 | 0.937 | [0.874; 0.974] |
| Krishnakumar 2017 | 47 | 47 | 1.000 | [0.925; 1.000] |
| Kumar 2019 | 114 | 114 | 1.000 | [0.968; 1.000] |
| Lakomkin 2017 | 21 | 21 | 1.000 | [0.839; 1.000] |
| Lau 2022 | 35 | 45 | 0.778 | [0.629; 0.888] |
| Lau 2022 | 35 | 45 | 0.778 | [0.629; 0.888] |
| Lee 2016 | 122 | 140 | 0.871 | [0.804; 0.922] |
| Li 2012 | 192 | 192 | 1.000 | [0.981; 1.000] |
| Liu 2022 | 284 | 299 | 0.950 | [0.919; 0.972] |
| Neira 2016 | 221 | 224 | 0.987 | [0.961; 0.997] |
| Noonan 2002 | 122 | 128 | 0.953 | [0.901; 0.983] |
| Padberg 1998 | 491 | 498 | 0.986 | [0.971; 0.994] |
| Pastorelli 2011 | 151 | 162 | 0.932 | [0.882; 0.966] |
| Pastorelli 2015 | 33 | 37 | 0.892 | [0.746; 0.970] |
| Pelosi 2002 | 108 | 118 | 0.915 | [0.850; 0.959] |
| Schwartz 2007 | 1083 | 1083 | 1.000 | [0.997; 1.000] |
| Skaggs 2009 | 539 | 543 | 0.993 | [0.981; 0.998] |
| Taylor 2021 | 533 | 540 | 0.987 | [0.973; 0.995] |
| Tiruchelvan 2013 | 2 | 9 | 0.222 | [0.028; 0.600] |
| Tsirikos 2020 | 1137 | 1145 | 0.993 | [0.986; 0.997] |
| Van der Wal 2021 | 52 | 67 | 0.776 | [0.658; 0.869] |
| Vitale 2010 | 131 | 139 | 0.942 | [0.890; 0.975] |
| Common effect model | | **6964** | **0.944** | **[0.939; 0.949]** |
| Random effects model | | | **0.953** | **[0.907; 0.977]** |
| Heterogeneity: $I^2 = 95\%$, $\tau^2 = 4.1170$, $p < 0.01$ | | | | |
| **Group = SSEP, MEP and EMG** | | | | |
| Bhagat 2015 | 290 | 292 | 0.993 | [0.975; 0.999] |
| Bhalodia (Acute Deficit) 2013 | 448 | 456 | 0.982 | [0.966; 0.992] |
| Bhalodia (Delayed) 2013 | 448 | 454 | 0.987 | [0.971; 0.995] |
| Bose 2007 | 169 | 226 | 0.748 | [0.686; 0.803] |
| Chen Yu 2021 | 101 | 104 | 0.971 | [0.918; 0.994] |
| Delgado−Lo_pez 2022 | 100 | 100 | 1.000 | [0.964; 1.000] |
| Duncan 2012 | 110 | 110 | 1.000 | [0.967; 1.000] |
| Eggspuehler (Cervical) 2007 | 232 | 234 | 0.991 | [0.969; 0.999] |
| Eggspuehler (Deformity) 2007 | 201 | 204 | 0.985 | [0.958; 0.997] |
| Eggspuehler (Thoracic) 2007 | 31 | 32 | 0.969 | [0.838; 0.999] |
| Ferguson 2014 | 472 | 515 | 0.917 | [0.889; 0.939] |
| Harel 2017 | 35 | 35 | 1.000 | [0.900; 1.000] |
| Ishida 2019 | 78 | 86 | 0.907 | [0.825; 0.959] |
| Jin (>50%) 2015 | 12 | 21 | 0.571 | [0.340; 0.782] |
| Jin (All or none) 2015 | 18 | 21 | 0.857 | [0.637; 0.970] |
| Kamerlink 2010 | 268 | 268 | 1.000 | [0.986; 1.000] |
| Kim 2019 | 161 | 268 | 0.601 | [0.539; 0.660] |
| Kobayashi 2017 | 54 | 65 | 0.831 | [0.717; 0.912] |
| MacDonald 2007 | 394 | 402 | 0.980 | [0.961; 0.991] |
| Platabello 2015 | 70 | 74 | 0.946 | [0.867; 0.985] |
| Quraishi 2009 | 62 | 92 | 0.674 | [0.568; 0.768] |
| Schar 2017 | 2 | 12 | 0.167 | [0.021; 0.484] |
| Sebastian (Day of Discharge Nerve Root Deficit) 2021 | 33 | 51 | 0.647 | [0.501; 0.776] |
| Sebastian (Day of Discharge) McCormick 2021 | 36 | 56 | 0.643 | [0.504; 0.766] |
| Sebastian (Follow Up McCormick) 2021 | 37 | 64 | 0.578 | [0.448; 0.701] |
| Sebastian (Permanent Deficit) 2021 | 37 | 62 | 0.597 | [0.464; 0.719] |
| Sebastian (Postop Nerve Root Deficit) 2021 | 32 | 46 | 0.696 | [0.542; 0.823] |
| Sutter 2019 − 1 | 2590 | 2613 | 0.991 | [0.987; 0.994] |
| Sutter I 2007 | 82 | 83 | 0.988 | [0.935; 1.000] |
| Sutter II 2007 | 388 | 389 | 0.997 | [0.986; 1.000] |
| Tong Yu 2017 | 9 | 11 | 0.818 | [0.482; 0.977] |
| Wilent 2019 | 2376 | 2578 | 0.922 | [0.911; 0.932] |
| Yu 2018 | 8 | 10 | 0.800 | [0.444; 0.975] |
| Common effect model | | **10034** | **0.935** | **[0.930; 0.940]** |
| Random effects model | | | **0.943** | **[0.887; 0.972]** |
| Heterogeneity: $I^2 = 96\%$, $\tau^2 = 4.2756$, $p < 0.01$ | | | | |
| **Group = MEP and EMG** | | | | |
| Chen Yu 2021 | 101 | 104 | 0.971 | [0.918; 0.994] |
| Kim 2019 | 32 | 59 | 0.542 | [0.408; 0.673] |
| Lieberman 2008 | 10 | 25 | 0.400 | [0.211; 0.613] |
| Skinner 2005 | 5 | 6 | 0.833 | [0.359; 0.996] |
| Common effect model | | **194** | **0.763** | **[0.698; 0.817]** |
| Random effects model | | | **0.772** | **[0.400; 0.945]** |
| Heterogeneity: $I^2 = 91\%$, $\tau^2 = 2.3559$, $p < 0.01$ | | | | |
| **Group = SSEP, MEP and D−Wave** | | | | |
| Costa 2013 | 80 | 90 | 0.889 | [0.805; 0.945] |
| Dauleac 2022 | 58 | 86 | 0.674 | [0.565; 0.772] |
| Forster 2012 | 178 | 180 | 0.989 | [0.960; 0.999] |
| Ghadirpour 2015 | 68 | 70 | 0.971 | [0.901; 0.997] |
| Ghadirpour 2019 | 98 | 101 | 0.970 | [0.916; 0.994] |
| Korn ("Permanent" Change) 2015 | 70 | 74 | 0.946 | [0.867; 0.985] |
| Korn ("Reversible" Change) 2015 | 59 | 76 | 0.776 | [0.666; 0.864] |
| Common effect model | | **677** | **0.903** | **[0.878; 0.923]** |
| Random effects model | | | **0.931** | **[0.836; 0.973]** |
| Heterogeneity: $I^2 = 90\%$, $\tau^2 = 1.4841$, $p < 0.01$ | | | | |
| **Group = SSEP and D−Wave** | | | | |
| Gavaret 2011 | 255 | 259 | 0.985 | [0.961; 0.996] |
| **Group = SSEP and EMG** | | | | |
| Gunnarsson 2004 | 49 | 199 | 0.246 | [0.188; 0.312] |
| Melachuri 2021 | 1012 | 1124 | 0.900 | [0.881; 0.917] |
| Common effect model | | **1323** | **0.802** | **[0.780; 0.823]** |
| Random effects model | | | **0.633** | **[0.146; 0.945]** |
| Heterogeneity: $I^2 = 100\%$, $\tau^2 = 2.7549$, $p < 0.01$ | | | | |
| **Group = MEP and D−Wave** | | | | |
| Hsu 2008 | 157 | 162 | 0.969 | [0.929; 0.990] |
| Sutter 2019 − 2 | 2593 | 2613 | 0.992 | [0.988; 0.995] |
| Sutter 2019 − 3 | 2593 | 2613 | 0.992 | [0.988; 0.995] |
| Common effect model | | **5388** | **0.992** | **[0.989; 0.994]** |
| Random effects model | | | **0.992** | **[0.989; 0.994]** |
| Heterogeneity: $I^2 = 77\%$, $\tau^2 = 0$, $p = 0.01$ | | | | |
| **Common effect model** | | **24839** | **0.941** | **[0.938; 0.944]** |
| **Random effects model** | | | **0.944** | **[0.914; 0.964]** |

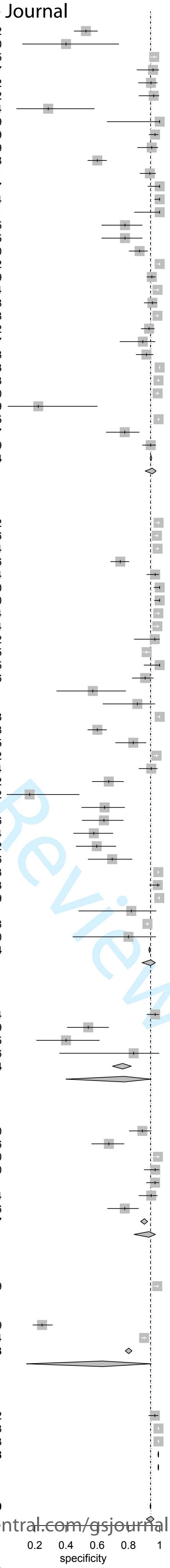


Heterogeneity: $I^2 = 96\%$, $\tau^2 = 3.9819$, $p = 0$
Test for subgroup differences (common effect): $\chi^2_6 = 620.09$, df = 6 ($p < 0.01$)
Test for subgroup differences (random effects): $\chi^2_6 = 70.20$, df = 6 ($p < 0.01$)

For Peer Review

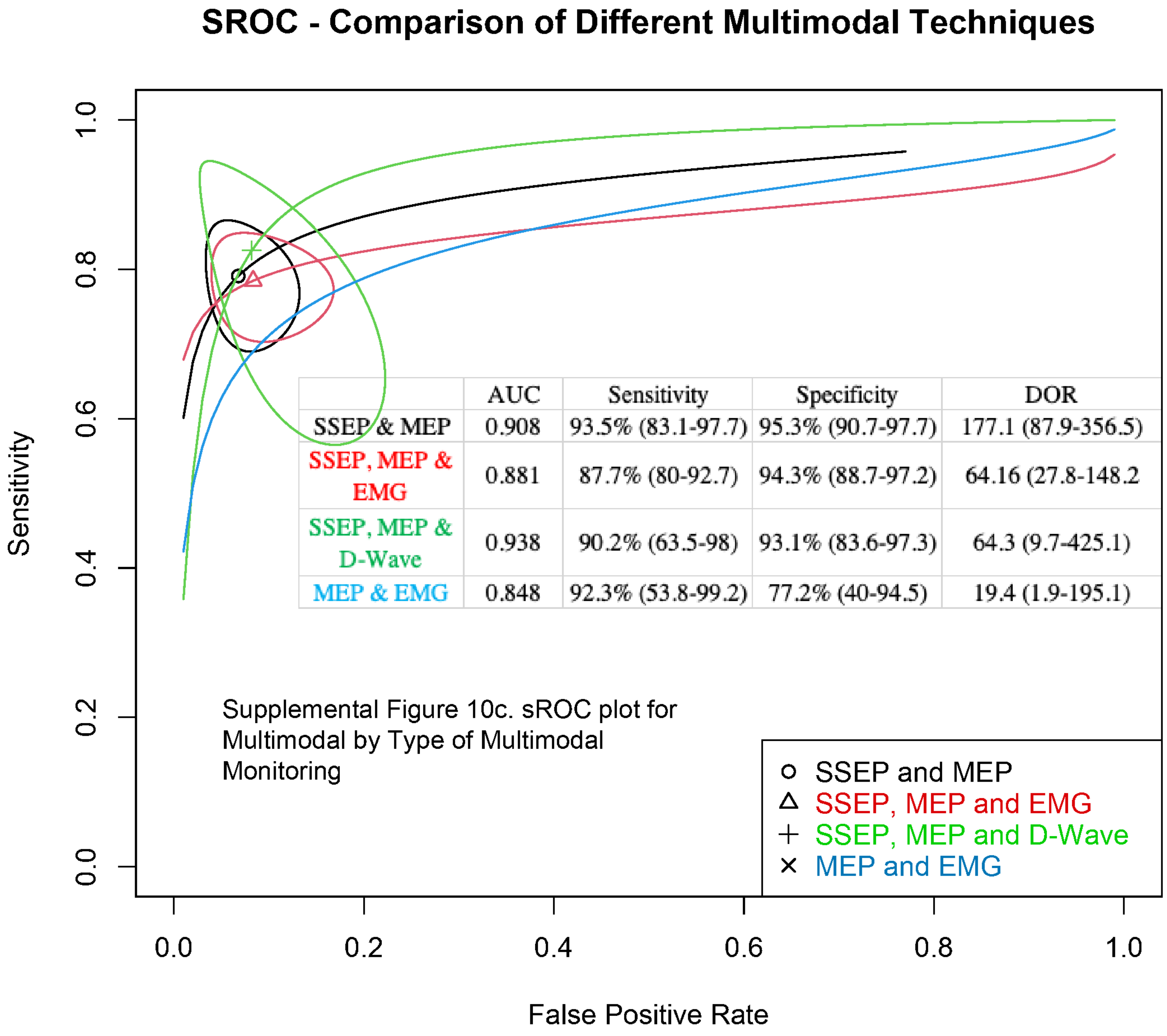


| | AUC | Sensitivity | Specificity | DOR |
|---|---|---|---|---|
| SSEP & MEP | 0.908 | 93.5% (83.1-97.7) | 95.3% (90.7-97.7) | 177.1 (87.9-356.5) |
| SSEP, MEP & EMG | 0.881 | 87.7% (80-92.7) | 94.3% (88.7-97.2) | 64.16 (27.8-148.2 |
| SSEP, MEP & D-Wave | 0.938 | 90.2% (63.5-98) | 93.1% (83.6-97.3) | 64.3 (9.7-425.1) |
| MEP & EMG | 0.848 | 92.3% (53.8-99.2) | 77.2% (40-94.5) | 19.4 (1.9-195.1) |

Supplemental Figure 10c. sROC plot for Multimodal by Type of Multimodal Monitoring

**Supplemental Figure 11a. Forest plot for Sensitivity for Multimodal by Disease Grou**

For Peer Review

| Study | Events | Total | Proportion | 95%−CI |
|---|---|---|---|---|
| **Pathology = Deformity** | | | | |
| Accadbled 2006 | 5 | 5 | 1.000 | [0.378; 0.995] |
| Bhagat 2015 | 23 | 23 | 1.000 | [0.741; 0.999] |
| Dicindio 2003 | 1 | 1 | 1.000 | [0.109; 0.987] |
| Eggspuehler (Deformity) 2007 | 12 | 13 | 0.923 | [0.609; 0.989] |
| Ferguson 2014 | 4 | 4 | 1.000 | [0.326; 0.994] |
| Hawary 2006 | 2 | 2 | 1.000 | [0.194; 0.990] |
| Hsu 2008 | 10 | 10 | 1.000 | [0.552; 0.997] |
| Kamerlink 2010 | 13 | 13 | 1.000 | [0.616; 0.998] |
| Kobayashi 2017 | 3 | 3 | 1.000 | [0.266; 0.993] |
| Krishnakumar 2017 | 5 | 5 | 1.000 | [0.378; 0.995] |
| Lau 2022 | 4 | 4 | 1.000 | [0.326; 0.994] |
| Lau 2022 | 2 | 7 | 0.286 | [0.072; 0.673] |
| Lieberman 2008 | 10 | 10 | 1.000 | [0.552; 0.997] |
| Neira 2016 | 47 | 51 | 0.922 | [0.809; 0.970] |
| Noonan 2002 | 6 | 6 | 1.000 | [0.423; 0.996] |
| Padberg 1998 | 2 | 2 | 1.000 | [0.194; 0.990] |
| Pastorelli 2011 | 9 | 10 | 0.900 | [0.533; 0.986] |
| Pastorelli 2015 | 3 | 3 | 1.000 | [0.266; 0.993] |
| Quraishi 2009 | 5 | 10 | 0.500 | [0.225; 0.775] |
| Schwartz 2007 | 38 | 38 | 1.000 | [0.825; 0.999] |
| Skaggs 2009 | 1 | 4 | 0.250 | [0.034; 0.762] |
| Tong Yu 2017 | 1 | 1 | 1.000 | [0.109; 0.987] |
| Tsirikos 2020 | 10 | 10 | 1.000 | [0.552; 0.997] |
| Vitale 2010 | 12 | 12 | 1.000 | [0.597; 0.998] |
| Common effect model | | **247** | **0.923** | **[0.883; 0.950]** |
| Random effects model | | | **0.988** | **[0.889; 0.999]** |
| Heterogeneity: $I^2 = 0\%$, $\tau^2 = 7.9203$, $p = 0.51$ | | | | |
| **Pathology = Various** | | | | |
| Almahdy 2021 | 10 | 10 | 1.000 | [0.552; 0.997] |
| Bhalodia (Acute Deficit) 2013 | 5 | 5 | 1.000 | [0.378; 0.995] |
| Bhalodia (Delayed) 2013 | 1 | 9 | 0.111 | [0.015; 0.500] |
| Bose 2007 | 11 | 12 | 0.917 | [0.587; 0.988] |
| Chen 2015 | 51 | 51 | 1.000 | [0.864; 0.999] |
| Costa 2007 | 3 | 3 | 1.000 | [0.266; 0.993] |
| Costa 2013 | 11 | 11 | 1.000 | [0.575; 0.997] |
| Eggspuehler (Cervical) 2007 | 10 | 12 | 0.833 | [0.523; 0.958] |
| Gavaret 2011 | 34 | 34 | 1.000 | [0.809; 0.999] |
| Gunnarsson 2004 | 14 | 14 | 1.000 | [0.634; 0.998] |
| Lee 2016 | 5 | 6 | 0.833 | [0.369; 0.977] |
| Li 2012 | 8 | 8 | 1.000 | [0.495; 0.997] |
| MacDonald 2007 | 7 | 10 | 0.700 | [0.376; 0.900] |
| Melachuri 2021 | 9 | 25 | 0.360 | [0.199; 0.560] |
| Pelosi 2002 | 6 | 6 | 1.000 | [0.423; 0.996] |
| Sutter 2019 − 1 | 107 | 115 | 0.930 | [0.867; 0.965] |
| Sutter 2019 − 2 | 105 | 115 | 0.913 | [0.846; 0.953] |
| Sutter 2019 − 3 | 102 | 115 | 0.887 | [0.815; 0.933] |
| Wilent 2019 | 7 | 7 | 1.000 | [0.461; 0.996] |
| Common effect model | | **568** | **0.891** | **[0.862; 0.914]** |
| Random effects model | | | **0.956** | **[0.841; 0.989]** |
| Heterogeneity: $I^2 = 68\%$, $\tau^2 = 4.9436$, $p < 0.01$ | | | | |
| **Pathology = Degenerative** | | | | |
| Chen Yu 2021 | 7 | 7 | 1.000 | [0.461; 0.996] |
| Chen Yu 2021 | 6 | 7 | 0.857 | [0.419; 0.980] |
| Delgado−Lo_pez 2022 | 1 | 2 | 0.500 | [0.059; 0.941] |
| Duncan 2012 | 5 | 5 | 1.000 | [0.378; 0.995] |
| Eggspuehler (Thoracic) 2007 | 3 | 4 | 0.750 | [0.238; 0.966] |
| Kim 2017 | 4 | 5 | 0.800 | [0.309; 0.973] |
| Kim 2019 | 4 | 7 | 0.571 | [0.230; 0.856] |
| Kim 2019 | 4 | 7 | 0.571 | [0.230; 0.856] |
| Kim 2019 | 3 | 7 | 0.429 | [0.144; 0.770] |
| Kim 2019 | 4 | 7 | 0.571 | [0.230; 0.856] |
| Kim 2021 | 2 | 5 | 0.400 | [0.100; 0.800] |
| Liu 2022 | 18 | 27 | 0.667 | [0.473; 0.817] |
| Platabello 2015 | 1 | 1 | 1.000 | [0.109; 0.987] |
| Schar 2017 | 5 | 5 | 1.000 | [0.378; 0.995] |
| Sutter II 2007 | 18 | 20 | 0.900 | [0.676; 0.975] |
| Taylor 2021 | 0 | 0 | 1.000 | [0.063; 0.984] |
| Common effect model | | **116** | **0.734** | **[0.646; 0.806]** |
| Random effects model | | | **0.747** | **[0.623; 0.840]** |
| Heterogeneity: $I^2 = 0\%$, $\tau^2 = 0.3559$, $p = 0.84$ | | | | |
| **Pathology = Tumor** | | | | |
| Dauleac 2022 | 11 | 30 | 0.367 | [0.216; 0.549] |
| Forster 2012 | 21 | 21 | 1.000 | [0.723; 0.999] |
| Ghadirpour 2015 | 3 | 3 | 1.000 | [0.266; 0.993] |
| Ghadirpour 2019 | 6 | 7 | 0.857 | [0.419; 0.980] |
| Harel 2017 | 3 | 4 | 0.750 | [0.238; 0.966] |
| Hyun 2009 | 3 | 3 | 1.000 | [0.266; 0.993] |
| Ishida 2019 | 14 | 17 | 0.824 | [0.573; 0.942] |
| Jin (>50%) 2015 | 4 | 4 | 1.000 | [0.326; 0.994] |
| Jin (All or none) 2015 | 2 | 4 | 0.500 | [0.123; 0.877] |
| Kim 2014 | 6 | 7 | 0.857 | [0.419; 0.980] |
| Korn ("Permanent" Change) 2015 | 18 | 22 | 0.818 | [0.604; 0.930] |
| Korn ("Reversible" Change) 2015 | 19 | 23 | 0.826 | [0.618; 0.933] |
| Kumar 2019 | 13 | 14 | 0.929 | [0.630; 0.990] |
| Lakomkin 2017 | 7 | 12 | 0.583 | [0.308; 0.815] |
| Sebastian (Day of Discharge Nerve Root Deficit) 2021 | 15 | 20 | 0.750 | [0.522; 0.892] |
| Sebastian (Day of Discharge) McCormick 2021 | 13 | 15 | 0.867 | [0.595; 0.966] |
| Sebastian (Follow Up McCormick) 2021 | 6 | 7 | 0.857 | [0.419; 0.980] |
| Sebastian (Permanent Deficit) 2021 | 8 | 9 | 0.889 | [0.500; 0.985] |
| Sebastian (Postop Nerve Root Deficit) 2021 | 19 | 25 | 0.760 | [0.558; 0.888] |
| Skinner 2005 | 8 | 8 | 1.000 | [0.495; 0.997] |
| Sutter I 2007 | 24 | 26 | 0.923 | [0.739; 0.981] |
| Tiruchelvan 2013 | 2 | 2 | 1.000 | [0.194; 0.990] |
| Van der Wal 2021 | 8 | 11 | 0.727 | [0.414; 0.910] |
| Common effect model | | **294** | **0.793** | **[0.742; 0.835]** |
| Random effects model | | | **0.839** | **[0.756; 0.898]** |
| Heterogeneity: $I^2 = 31\%$, $\tau^2 = 0.6695$, $p = 0.08$ | | | | |
| **Pathology = Trauma** | | | | |
| Yu 2018 | 1 | 1 | 1.000 | [0.109; 0.987] |
| **Common effect model** | | **1226** | **0.859** | **[0.838; 0.877]** |
| **Random effects model** | | | **0.910** | **[0.860; 0.943]** |

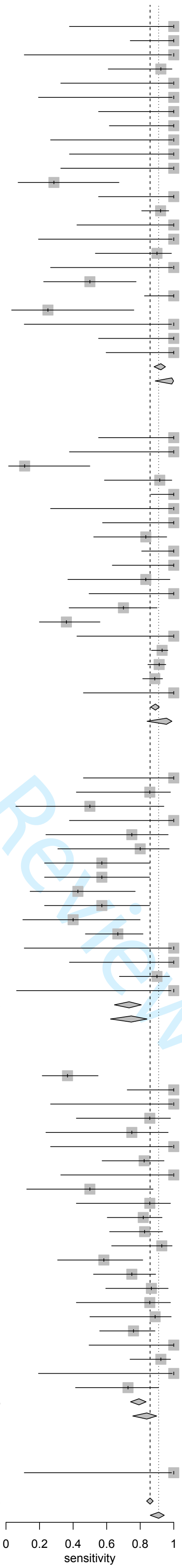


Heterogeneity: $I^2 = 40\%$, $\tau^2 = 2.4511$, $p < 0.01$
Test for subgroup differences (common effect): $\chi^2_4 = 36.51$, df = 4 ($p < 0.01$)
Test for subgroup differences (random effects): $\chi^2_4 = 12.90$, df = 4 ($p = 0.01$)

**Supplemental Figure 11b. Forest plot for Specificity for Multimodal by Disease Group**



| Study | Events | Total | Proportion | 95%−CI |
|---|---|---|---|---|
| **Pathology = Deformity** | | | | |
| Accadbled 2006 | 96 | 182 | 0.527 | [0.452; 0.602] |
| Bhagat 2015 | 290 | 292 | 0.993 | [0.975; 0.999] |
| Dicindio 2003 | 68 | 72 | 0.944 | [0.864; 0.985] |
| Eggspuehler (Deformity) 2007 | 201 | 204 | 0.985 | [0.958; 0.997] |
| Ferguson 2014 | 472 | 515 | 0.917 | [0.889; 0.939] |
| Hawary 2006 | 50 | 52 | 0.962 | [0.868; 0.995] |
| Hsu 2008 | 157 | 162 | 0.969 | [0.929; 0.990] |
| Kamerlink 2010 | 268 | 268 | 1.000 | [0.986; 1.000] |
| Kobayashi 2017 | 54 | 65 | 0.831 | [0.717; 0.912] |
| Krishnakumar 2017 | 47 | 47 | 1.000 | [0.925; 1.000] |
| Lau 2022 | 35 | 45 | 0.778 | [0.629; 0.888] |
| Lau 2022 | 35 | 45 | 0.778 | [0.629; 0.888] |
| Lieberman 2008 | 10 | 25 | 0.400 | [0.211; 0.613] |
| Neira 2016 | 221 | 224 | 0.987 | [0.961; 0.997] |
| Noonan 2002 | 122 | 128 | 0.953 | [0.901; 0.983] |
| Padberg 1998 | 491 | 498 | 0.986 | [0.971; 0.994] |
| Pastorelli 2011 | 151 | 162 | 0.932 | [0.882; 0.966] |
| Pastorelli 2015 | 33 | 37 | 0.892 | [0.746; 0.970] |
| Quraishi 2009 | 62 | 92 | 0.674 | [0.568; 0.768] |
| Schwartz 2007 | 1083 | 1083 | 1.000 | [0.997; 1.000] |
| Skaggs 2009 | 539 | 543 | 0.993 | [0.981; 0.998] |
| Tong Yu 2017 | 9 | 11 | 0.818 | [0.482; 0.977] |
| Tsirikos 2020 | 1137 | 1145 | 0.993 | [0.986; 0.997] |
| Vitale 2010 | 131 | 139 | 0.942 | [0.890; 0.975] |
| **Common effect model** | | **6036** | **0.955** | **[0.949; 0.960]** |
| **Random effects model** | | | **0.960** | **[0.914; 0.982]** |
| Heterogeneity: $I^2$ = 95%, $\tau^2$ = 3.7856, $p$ < 0.01 | | | | |
| **Pathology = Various** | | | | |
| Almahdy 2021 | 4 | 10 | 0.400 | [0.122; 0.738] |
| Bhalodia (Acute Deficit) 2013 | 448 | 456 | 0.982 | [0.966; 0.992] |
| Bhalodia (Delayed) 2013 | 448 | 454 | 0.987 | [0.971; 0.995] |
| Bose 2007 | 169 | 226 | 0.748 | [0.686; 0.803] |
| Chen 2015 | 362 | 375 | 0.965 | [0.941; 0.981] |
| Costa 2007 | 45 | 47 | 0.957 | [0.855; 0.995] |
| Costa 2013 | 80 | 90 | 0.889 | [0.805; 0.945] |
| Eggspuehler (Cervical) 2007 | 232 | 234 | 0.991 | [0.969; 0.999] |
| Gavaret 2011 | 255 | 259 | 0.985 | [0.961; 0.996] |
| Gunnarsson 2004 | 49 | 199 | 0.246 | [0.188; 0.312] |
| Lee 2016 | 122 | 140 | 0.871 | [0.804; 0.922] |
| Li 2012 | 192 | 192 | 1.000 | [0.981; 1.000] |
| MacDonald 2007 | 394 | 402 | 0.980 | [0.961; 0.991] |
| Melachuri 2021 | 1012 | 1124 | 0.900 | [0.881; 0.917] |
| Pelosi 2002 | 108 | 118 | 0.915 | [0.850; 0.959] |
| Sutter 2019 − 1 | 2590 | 2613 | 0.991 | [0.987; 0.994] |
| Sutter 2019 − 2 | 2593 | 2613 | 0.992 | [0.988; 0.995] |
| Sutter 2019 − 3 | 2593 | 2613 | 0.992 | [0.988; 0.995] |
| Wilent 2019 | 2376 | 2578 | 0.922 | [0.911; 0.932] |
| **Common effect model** | | **14743** | **0.954** | **[0.951; 0.958]** |
| **Random effects model** | | | **0.958** | **[0.908; 0.981]** |
| Heterogeneity: $I^2$ = 98%, $\tau^2$ = 3.2695, $p$ < 0.01 | | | | |
| **Pathology = Degenerative** | | | | |
| Chen Yu 2021 | 101 | 104 | 0.971 | [0.918; 0.994] |
| Chen Yu 2021 | 101 | 104 | 0.971 | [0.918; 0.994] |
| Delgado−Lo_pez 2022 | 100 | 100 | 1.000 | [0.964; 1.000] |
| Duncan 2012 | 110 | 110 | 1.000 | [0.967; 1.000] |
| Eggspuehler (Thoracic) 2007 | 31 | 32 | 0.969 | [0.838; 0.999] |
| Kim 2017 | 164 | 169 | 0.970 | [0.932; 0.990] |
| Kim 2019 | 56 | 59 | 0.949 | [0.859; 0.989] |
| Kim 2019 | 32 | 59 | 0.542 | [0.408; 0.673] |
| Kim 2019 | 161 | 268 | 0.601 | [0.539; 0.660] |
| Kim 2019 | 161 | 268 | 0.601 | [0.539; 0.660] |
| Kim 2021 | 104 | 111 | 0.937 | [0.874; 0.974] |
| Liu 2022 | 284 | 299 | 0.950 | [0.919; 0.972] |
| Platabello 2015 | 70 | 74 | 0.946 | [0.867; 0.985] |
| Schar 2017 | 2 | 12 | 0.167 | [0.021; 0.484] |
| Sutter II 2007 | 388 | 389 | 0.997 | [0.986; 1.000] |
| Taylor 2021 | 533 | 540 | 0.987 | [0.973; 0.995] |
| **Common effect model** | | **2698** | **0.889** | **[0.876; 0.900]** |
| **Random effects model** | | | **0.952** | **[0.867; 0.983]** |
| Heterogeneity: $I^2$ = 96%, $\tau^2$ = 4.5036, $p$ < 0.01 | | | | |
| **Pathology = Tumor** | | | | |
| Dauleac 2022 | 58 | 86 | 0.674 | [0.565; 0.772] |
| Forster 2012 | 178 | 180 | 0.989 | [0.960; 0.999] |
| Ghadirpour 2015 | 68 | 70 | 0.971 | [0.901; 0.997] |
| Ghadirpour 2019 | 98 | 101 | 0.970 | [0.916; 0.994] |
| Harel 2017 | 35 | 35 | 1.000 | [0.900; 1.000] |
| Hyun 2009 | 4 | 14 | 0.286 | [0.084; 0.581] |
| Ishida 2019 | 78 | 86 | 0.907 | [0.825; 0.959] |
| Jin (>50%) 2015 | 12 | 21 | 0.571 | [0.340; 0.782] |
| Jin (All or none) 2015 | 18 | 21 | 0.857 | [0.637; 0.970] |
| Kim 2014 | 9 | 9 | 1.000 | [0.664; 1.000] |
| Korn ("Permanent" Change) 2015 | 70 | 74 | 0.946 | [0.867; 0.985] |
| Korn ("Reversible" Change) 2015 | 59 | 76 | 0.776 | [0.666; 0.864] |
| Kumar 2019 | 114 | 114 | 1.000 | [0.968; 1.000] |
| Lakomkin 2017 | 21 | 21 | 1.000 | [0.839; 1.000] |
| Sebastian (Day of Discharge Nerve Root Deficit) 2021 | 33 | 51 | 0.647 | [0.501; 0.776] |
| Sebastian (Day of Discharge) McCormick 2021 | 36 | 56 | 0.643 | [0.504; 0.766] |
| Sebastian (Follow Up McCormick) 2021 | 37 | 64 | 0.578 | [0.448; 0.701] |
| Sebastian (Permanent Deficit) 2021 | 37 | 62 | 0.597 | [0.464; 0.719] |
| Sebastian (Postop Nerve Root Deficit) 2021 | 32 | 46 | 0.696 | [0.542; 0.823] |
| Skinner 2005 | 5 | 6 | 0.833 | [0.359; 0.996] |
| Sutter I 2007 | 82 | 83 | 0.988 | [0.935; 1.000] |
| Tiruchelvan 2013 | 2 | 9 | 0.222 | [0.028; 0.600] |
| Van der Wal 2021 | 52 | 67 | 0.776 | [0.658; 0.869] |
| **Common effect model** | | **1352** | **0.842** | **[0.821; 0.860]** |
| **Random effects model** | | | **0.886** | **[0.771; 0.947]** |
| Heterogeneity: $I^2$ = 84%, $\tau^2$ = 3.4992, $p$ < 0.01 | | | | |
| **Pathology = Trauma** | | | | |
| Yu 2018 | 8 | 10 | 0.800 | [0.444; 0.975] |
| **Common effect model** | | **24839** | **0.941** | **[0.938; 0.944]** |
| **Random effects model** | | | **0.944** | **[0.914; 0.964]** |

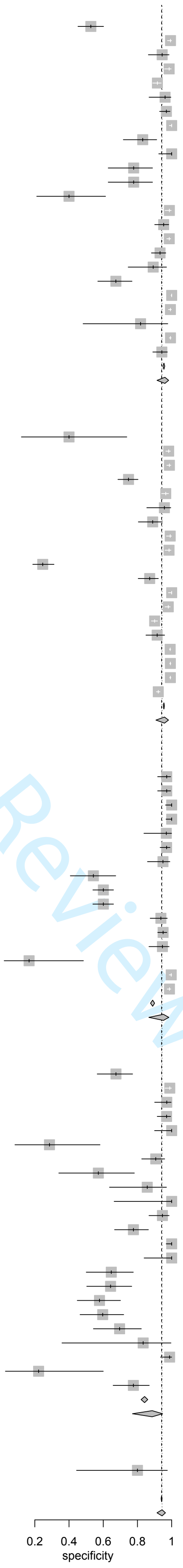


Heterogeneity: $I^2$ = 96%, $\tau^2$ = 3.9819, $p$ = 0
Test for subgroup differences (common effect): $\chi^2_4$ = 401.80, df = 4 ($p$ < 0.01)
Test for subgroup differences (random effects): $\chi^2_4$ = 7.69, df = 4 ($p$ = 0.10)

For Peer Review

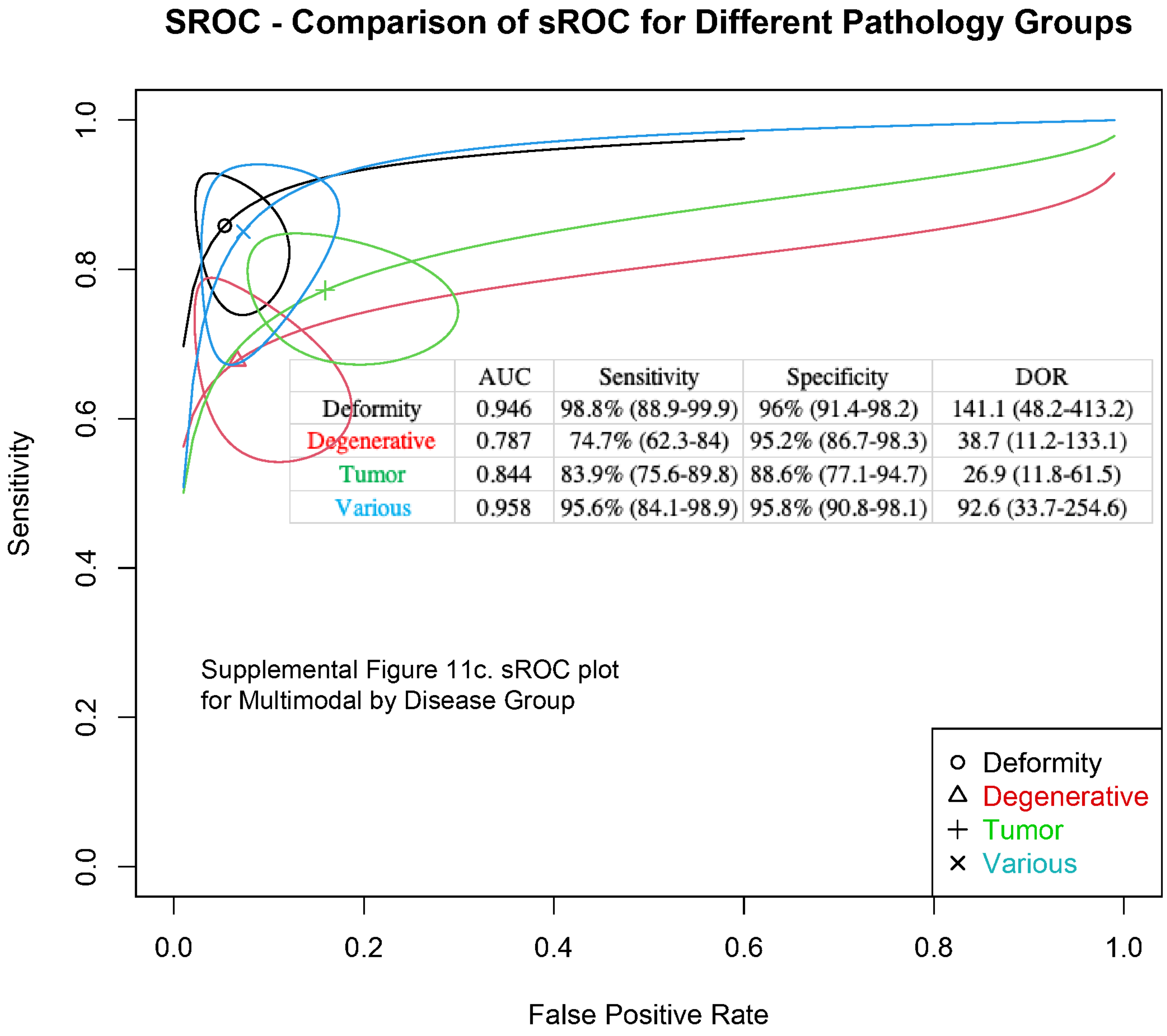


Supplemental Figure 11c. sROC plot for Multimodal by Disease Group

Global Spine Journal

**Supplemental Figure 12a. Forest plot for Sensitivity for Multimodal by Region of Surgery**

| Study | Events | Total | Proportion | 95%−CI |
|---|---|---|---|---|
| **Region = Thoracolumbar** | | | | |
| Accadbled 2006 | 5 | 5 | 1.000 | [0.378; 0.995] |
| Bhagat 2015 | 23 | 23 | 1.000 | [0.741; 0.999] |
| Eggspuehler (Deformity) 2007 | 12 | 13 | 0.923 | [0.609; 0.989] |
| Hsu 2008 | 10 | 10 | 1.000 | [0.552; 0.997] |
| Kobayashi 2017 | 3 | 3 | 1.000 | [0.266; 0.993] |
| Lieberman 2008 | 10 | 10 | 1.000 | [0.552; 0.997] |
| MacDonald 2007 | 7 | 10 | 0.700 | [0.376; 0.900] |
| Noonan 2002 | 6 | 6 | 1.000 | [0.423; 0.996] |
| Pastorelli 2011 | 9 | 10 | 0.900 | [0.533; 0.986] |
| Tong Yu 2017 | 1 | 1 | 1.000 | [0.109; 0.987] |
| Yu 2018 | 1 | 1 | 1.000 | [0.109; 0.987] |
| Common effect model | | 92 | 0.946 | [0.876; 0.977] |
| Random effects model | | | 0.969 | [0.826; 0.995] |
| Heterogeneity: $I^2 = 0\%$, $\tau^2 = 1.4960$, $p = 0.99$ | | | | |
| **Region = Cervicothoracic** | | | | |
| Almahdy 2021 | 10 | 10 | 1.000 | [0.552; 0.997] |
| Lau 2022 | 4 | 4 | 1.000 | [0.326; 0.994] |
| Lau 2022 | 2 | 7 | 0.286 | [0.072; 0.673] |
| Tiruchelvan 2013 | 2 | 2 | 1.000 | [0.194; 0.990] |
| Common effect model | | 23 | 0.783 | [0.572; 0.907] |
| Random effects model | | | 0.984 | [0.064; 1.000] |
| Heterogeneity: $I^2 = 0\%$, $\tau^2 = 13.0216$, $p = 1.00$ | | | | |
| **Region = Cervical** | | | | |
| Bhalodia (Acute Deficit) 2013 | 5 | 5 | 1.000 | [0.378; 0.995] |
| Bhalodia (Delayed) 2013 | 1 | 9 | 0.111 | [0.015; 0.500] |
| Bose 2007 | 11 | 12 | 0.917 | [0.587; 0.988] |
| Delgado−Lo_pez 2022 | 1 | 2 | 0.500 | [0.059; 0.941] |
| Eggspuehler (Cervical) 2007 | 10 | 12 | 0.833 | [0.523; 0.958] |
| Kim 2017 | 4 | 5 | 0.800 | [0.309; 0.973] |
| Kim 2021 | 2 | 5 | 0.400 | [0.100; 0.800] |
| Lee 2016 | 5 | 6 | 0.833 | [0.369; 0.977] |
| Li 2012 | 8 | 8 | 1.000 | [0.495; 0.997] |
| Platabello 2015 | 1 | 1 | 1.000 | [0.109; 0.987] |
| Taylor 2021 | 0 | 0 | 1.000 | [0.063; 0.984] |
| Common effect model | | 66 | 0.740 | [0.622; 0.832] |
| Random effects model | | | 0.812 | [0.540; 0.941] |
| Heterogeneity: $I^2 = 27\%$, $\tau^2 = 2.4484$, $p = 0.19$ | | | | |
| **Region = All** | | | | |
| Chen 2015 | 51 | 51 | 1.000 | [0.864; 0.999] |
| Costa 2007 | 3 | 3 | 1.000 | [0.266; 0.993] |
| Costa 2013 | 11 | 11 | 1.000 | [0.575; 0.997] |
| Dauleac 2022 | 11 | 30 | 0.367 | [0.216; 0.549] |
| Dicindio 2003 | 1 | 1 | 1.000 | [0.109; 0.987] |
| Ferguson 2014 | 4 | 4 | 1.000 | [0.326; 0.994] |
| Forster 2012 | 21 | 21 | 1.000 | [0.723; 0.999] |
| Gavaret 2011 | 34 | 34 | 1.000 | [0.809; 0.999] |
| Ghadirpour 2015 | 3 | 3 | 1.000 | [0.266; 0.993] |
| Ghadirpour 2019 | 6 | 7 | 0.857 | [0.419; 0.980] |
| Gunnarsson 2004 | 14 | 14 | 1.000 | [0.634; 0.998] |
| Harel 2017 | 3 | 4 | 0.750 | [0.238; 0.966] |
| Hawary 2006 | 2 | 2 | 1.000 | [0.194; 0.990] |
| Hyun 2009 | 3 | 3 | 1.000 | [0.266; 0.993] |
| Ishida 2019 | 14 | 17 | 0.824 | [0.573; 0.942] |
| Jin (>50%) 2015 | 4 | 4 | 1.000 | [0.326; 0.994] |
| Jin (All or none) 2015 | 2 | 4 | 0.500 | [0.123; 0.877] |
| Kamerlink 2010 | 13 | 13 | 1.000 | [0.616; 0.998] |
| Kim 2014 | 6 | 7 | 0.857 | [0.419; 0.980] |
| Korn ("Permanent" Change) 2015 | 18 | 22 | 0.818 | [0.604; 0.930] |
| Korn ("Reversible" Change) 2015 | 19 | 23 | 0.826 | [0.618; 0.933] |
| Krishnakumar 2017 | 5 | 5 | 1.000 | [0.378; 0.995] |
| Kumar 2019 | 13 | 14 | 0.929 | [0.630; 0.990] |
| Lakomkin 2017 | 7 | 12 | 0.583 | [0.308; 0.815] |
| Neira 2016 | 47 | 51 | 0.922 | [0.809; 0.970] |
| Padberg 1998 | 2 | 2 | 1.000 | [0.194; 0.990] |
| Pastorelli 2015 | 3 | 3 | 1.000 | [0.266; 0.993] |
| Pelosi 2002 | 6 | 6 | 1.000 | [0.423; 0.996] |
| Quraishi 2009 | 5 | 10 | 0.500 | [0.225; 0.775] |
| Schwartz 2007 | 38 | 38 | 1.000 | [0.825; 0.999] |
| Sebastian (Day of Discharge Nerve Root Deficit) 2021 | 15 | 20 | 0.750 | [0.522; 0.892] |
| Sebastian (Day of Discharge) McCormick 2021 | 13 | 15 | 0.867 | [0.595; 0.966] |
| Sebastian (Follow Up McCormick) 2021 | 6 | 7 | 0.857 | [0.419; 0.980] |
| Sebastian (Permanent Deficit) 2021 | 8 | 9 | 0.889 | [0.500; 0.985] |
| Sebastian (Postop Nerve Root Deficit) 2021 | 19 | 25 | 0.760 | [0.558; 0.888] |
| Skaggs 2009 | 1 | 4 | 0.250 | [0.034; 0.762] |
| Skinner 2005 | 8 | 8 | 1.000 | [0.495; 0.997] |
| Sutter 2019 − 1 | 107 | 115 | 0.930 | [0.867; 0.965] |
| Sutter 2019 − 2 | 105 | 115 | 0.913 | [0.846; 0.953] |
| Sutter 2019 − 3 | 102 | 115 | 0.887 | [0.815; 0.933] |
| Sutter I 2007 | 24 | 26 | 0.923 | [0.739; 0.981] |
| Tsirikos 2020 | 10 | 10 | 1.000 | [0.552; 0.997] |
| Van der Wal 2021 | 8 | 11 | 0.727 | [0.414; 0.910] |
| Vitale 2010 | 12 | 12 | 1.000 | [0.597; 0.998] |
| Wilent 2019 | 7 | 7 | 1.000 | [0.461; 0.996] |
| Common effect model | | 918 | 0.887 | [0.865; 0.906] |
| Random effects model | | | 0.925 | [0.869; 0.958] |
| Heterogeneity: $I^2 = 41\%$, $\tau^2 = 1.9260$, $p < 0.01$ | | | | |
| **Region = Lumbosacral** | | | | |
| Chen Yu 2021 | 7 | 7 | 1.000 | [0.461; 0.996] |
| Chen Yu 2021 | 6 | 7 | 0.857 | [0.419; 0.980] |
| Duncan 2012 | 5 | 5 | 1.000 | [0.378; 0.995] |
| Kim 2019 | 4 | 7 | 0.571 | [0.230; 0.856] |
| Kim 2019 | 4 | 7 | 0.571 | [0.230; 0.856] |
| Kim 2019 | 3 | 7 | 0.429 | [0.144; 0.770] |
| Kim 2019 | 4 | 7 | 0.571 | [0.230; 0.856] |
| Melachuri 2021 | 9 | 25 | 0.360 | [0.199; 0.560] |
| Schar 2017 | 5 | 5 | 1.000 | [0.378; 0.995] |
| Sutter II 2007 | 18 | 20 | 0.900 | [0.676; 0.975] |
| Common effect model | | 97 | 0.670 | [0.571; 0.756] |
| Random effects model | | | 0.762 | [0.544; 0.896] |
| Heterogeneity: $I^2 = 32\%$, $\tau^2 = 1.3540$, $p = 0.15$ | | | | |
| **Region = Thoracic** | | | | |
| Eggspuehler (Thoracic) 2007 | 3 | 4 | 0.750 | [0.238; 0.966] |
| Liu 2022 | 18 | 27 | 0.667 | [0.473; 0.817] |
| Common effect model | | 31 | 0.677 | [0.497; 0.817] |
| Random effects model | | | 0.677 | [0.497; 0.817] |
| Heterogeneity: $I^2 = 0\%$, $\tau^2 = 0$, $p = 0.74$ | | | | |
| **Common effect model** | | **1226** | **0.859** | **[0.838; 0.877]** |
| **Random effects model** | | | **0.910** | **[0.860; 0.943]** |

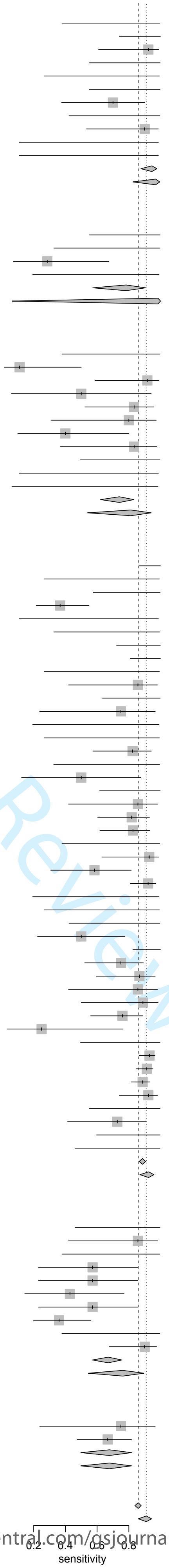


Heterogeneity: $I^2 = 40\%$, $\tau^2 = 2.4511$, $p < 0.01$
Test for subgroup differences (common effect): $\chi^2_5 = 51.43$, df = 5 ($p < 0.01$)
Test for subgroup differences (random effects): $\chi^2_5 = 17.86$, df = 5 ($p < 0.01$)

For Peer Review

Supplemental Figure 12b. Forest plot for Specificity for Multimodal by Region of Surgery

| Study | Events | Total | Proportion | 95%-CI |
|---|---|---|---|---|
| **Region = Thoracolumbar** | | | | |
| Accadbled 2006 | 96 | 182 | 0.527 | [0.452; 0.602] |
| Bhagat 2015 | 290 | 292 | 0.993 | [0.975; 0.999] |
| Eggspuehler (Deformity) 2007 | 201 | 204 | 0.985 | [0.958; 0.997] |
| Hsu 2008 | 157 | 162 | 0.969 | [0.929; 0.990] |
| Kobayashi 2017 | 54 | 65 | 0.831 | [0.717; 0.912] |
| Lieberman 2008 | 10 | 25 | 0.400 | [0.211; 0.613] |
| MacDonald 2007 | 394 | 402 | 0.980 | [0.961; 0.991] |
| Noonan 2002 | 122 | 128 | 0.953 | [0.901; 0.983] |
| Pastorelli 2011 | 151 | 162 | 0.932 | [0.882; 0.966] |
| Tong Yu 2017 | 9 | 11 | 0.818 | [0.482; 0.977] |
| Yu 2018 | 8 | 10 | 0.800 | [0.444; 0.975] |
| Common effect model | | **1643** | **0.908** | **[0.893; 0.921]** |
| Random effects model | | | **0.921** | **[0.812; 0.970]** |
| Heterogeneity: $I^2$ = 96%, $\tau^2$ = 2.5866, $p$ < 0.01 | | | | |
| **Region = Cervicothoracic** | | | | |
| Almahdy 2021 | 4 | 10 | 0.400 | [0.122; 0.738] |
| Lau 2022 | 35 | 45 | 0.778 | [0.629; 0.888] |
| Lau 2022 | 35 | 45 | 0.778 | [0.629; 0.888] |
| Tiruchelvan 2013 | 2 | 9 | 0.222 | [0.028; 0.600] |
| Common effect model | | **109** | **0.697** | **[0.605; 0.776]** |
| Random effects model | | | **0.595** | **[0.337; 0.810]** |
| Heterogeneity: $I^2$ = 77%, $\tau^2$ = 0.8423, $p$ < 0.01 | | | | |
| **Region = Cervical** | | | | |
| Bhalodia (Acute Deficit) 2013 | 448 | 456 | 0.982 | [0.966; 0.992] |
| Bhalodia (Delayed) 2013 | 448 | 454 | 0.987 | [0.971; 0.995] |
| Bose 2007 | 169 | 226 | 0.748 | [0.686; 0.803] |
| Delgado−Lo_pez 2022 | 100 | 100 | 1.000 | [0.964; 1.000] |
| Eggspuehler (Cervical) 2007 | 232 | 234 | 0.991 | [0.969; 0.999] |
| Kim 2017 | 164 | 169 | 0.970 | [0.932; 0.990] |
| Kim 2021 | 104 | 111 | 0.937 | [0.874; 0.974] |
| Lee 2016 | 122 | 140 | 0.871 | [0.804; 0.922] |
| Li 2012 | 192 | 192 | 1.000 | [0.981; 1.000] |
| Platabello 2015 | 70 | 74 | 0.946 | [0.867; 0.985] |
| Taylor 2021 | 533 | 540 | 0.987 | [0.973; 0.995] |
| Common effect model | | **2696** | **0.958** | **[0.949; 0.965]** |
| Random effects model | | | **0.976** | **[0.943; 0.991]** |
| Heterogeneity: $I^2$ = 94%, $\tau^2$ = 2.0009, $p$ < 0.01 | | | | |
| **Region = All** | | | | |
| Chen 2015 | 362 | 375 | 0.965 | [0.941; 0.981] |
| Costa 2007 | 45 | 47 | 0.957 | [0.855; 0.995] |
| Costa 2013 | 80 | 90 | 0.889 | [0.805; 0.945] |
| Dauleac 2022 | 58 | 86 | 0.674 | [0.565; 0.772] |
| Dicindio 2003 | 68 | 72 | 0.944 | [0.864; 0.985] |
| Ferguson 2014 | 472 | 515 | 0.917 | [0.889; 0.939] |
| Forster 2012 | 178 | 180 | 0.989 | [0.960; 0.999] |
| Gavaret 2011 | 255 | 259 | 0.985 | [0.961; 0.996] |
| Ghadirpour 2015 | 68 | 70 | 0.971 | [0.901; 0.997] |
| Ghadirpour 2019 | 98 | 101 | 0.970 | [0.916; 0.994] |
| Gunnarsson 2004 | 49 | 199 | 0.246 | [0.188; 0.312] |
| Harel 2017 | 35 | 35 | 1.000 | [0.900; 1.000] |
| Hawary 2006 | 50 | 52 | 0.962 | [0.868; 0.995] |
| Hyun 2009 | 4 | 14 | 0.286 | [0.084; 0.581] |
| Ishida 2019 | 78 | 86 | 0.907 | [0.825; 0.959] |
| Jin (>50%) 2015 | 12 | 21 | 0.571 | [0.340; 0.782] |
| Jin (All or none) 2015 | 18 | 21 | 0.857 | [0.637; 0.970] |
| Kamerlink 2010 | 268 | 268 | 1.000 | [0.986; 1.000] |
| Kim 2014 | 9 | 9 | 1.000 | [0.664; 1.000] |
| Korn ("Permanent" Change) 2015 | 70 | 74 | 0.946 | [0.867; 0.985] |
| Korn ("Reversible" Change) 2015 | 59 | 76 | 0.776 | [0.666; 0.864] |
| Krishnakumar 2017 | 47 | 47 | 1.000 | [0.925; 1.000] |
| Kumar 2019 | 114 | 114 | 1.000 | [0.968; 1.000] |
| Lakomkin 2017 | 21 | 21 | 1.000 | [0.839; 1.000] |
| Neira 2016 | 221 | 224 | 0.987 | [0.961; 0.997] |
| Padberg 1998 | 491 | 498 | 0.986 | [0.971; 0.994] |
| Pastorelli 2015 | 33 | 37 | 0.892 | [0.746; 0.970] |
| Pelosi 2002 | 108 | 118 | 0.915 | [0.850; 0.959] |
| Quraishi 2009 | 62 | 92 | 0.674 | [0.568; 0.768] |
| Schwartz 2007 | 1083 | 1083 | 1.000 | [0.997; 1.000] |
| Sebastian (Day of Discharge Nerve Root Deficit) 2021 | 33 | 51 | 0.647 | [0.501; 0.776] |
| Sebastian (Day of Discharge) McCormick 2021 | 36 | 56 | 0.643 | [0.504; 0.766] |
| Sebastian (Follow Up McCormick) 2021 | 37 | 64 | 0.578 | [0.448; 0.701] |
| Sebastian (Permanent Deficit) 2021 | 37 | 62 | 0.597 | [0.464; 0.719] |
| Sebastian (Postop Nerve Root Deficit) 2021 | 32 | 46 | 0.696 | [0.542; 0.823] |
| Skaggs 2009 | 539 | 543 | 0.993 | [0.981; 0.998] |
| Skinner 2005 | 5 | 6 | 0.833 | [0.359; 0.996] |
| Sutter 2019 − 1 | 2590 | 2613 | 0.991 | [0.987; 0.994] |
| Sutter 2019 − 2 | 2593 | 2613 | 0.992 | [0.988; 0.995] |
| Sutter 2019 − 3 | 2593 | 2613 | 0.992 | [0.988; 0.995] |
| Sutter I 2007 | 82 | 83 | 0.988 | [0.935; 1.000] |
| Tsirikos 2020 | 1137 | 1145 | 0.993 | [0.986; 0.997] |
| Van der Wal 2021 | 52 | 67 | 0.776 | [0.658; 0.869] |
| Vitale 2010 | 131 | 139 | 0.942 | [0.890; 0.975] |
| Wilent 2019 | 2376 | 2578 | 0.922 | [0.911; 0.932] |
| Common effect model | | **17563** | **0.956** | **[0.953; 0.959]** |
| Random effects model | | | **0.952** | **[0.914; 0.974]** |
| Heterogeneity: $I^2$ = 97%, $\tau^2$ = 4.0282, $p$ < 0.01 | | | | |
| **Region = Lumbosacral** | | | | |
| Chen Yu 2021 | 101 | 104 | 0.971 | [0.918; 0.994] |
| Chen Yu 2021 | 101 | 104 | 0.971 | [0.918; 0.994] |
| Duncan 2012 | 110 | 110 | 1.000 | [0.967; 1.000] |
| Kim 2019 | 56 | 59 | 0.949 | [0.859; 0.989] |
| Kim 2019 | 32 | 59 | 0.542 | [0.408; 0.673] |
| Kim 2019 | 161 | 268 | 0.601 | [0.539; 0.660] |
| Kim 2019 | 161 | 268 | 0.601 | [0.539; 0.660] |
| Melachuri 2021 | 1012 | 1124 | 0.900 | [0.881; 0.917] |
| Schar 2017 | 2 | 12 | 0.167 | [0.021; 0.484] |
| Sutter II 2007 | 388 | 389 | 0.997 | [0.986; 1.000] |
| Common effect model | | **2497** | **0.851** | **[0.836; 0.864]** |
| Random effects model | | | **0.915** | **[0.693; 0.981]** |
| Heterogeneity: $I^2$ = 97%, $\tau^2$ = 5.7884, $p$ < 0.01 | | | | |
| **Region = Thoracic** | | | | |
| Eggspuehler (Thoracic) 2007 | 31 | 32 | 0.969 | [0.838; 0.999] |
| Liu 2022 | 284 | 299 | 0.950 | [0.919; 0.972] |
| Common effect model | | **331** | **0.952** | **[0.923; 0.970]** |
| Random effects model | | | **0.952** | **[0.923; 0.970]** |
| Heterogeneity: $I^2$ = 0%, $\tau^2$ = 0, $p$ = 0.64 | | | | |
| **Common effect model** | | **24839** | **0.941** | **[0.938; 0.944]** |
| **Random effects model** | | | **0.944** | **[0.914; 0.964]** |

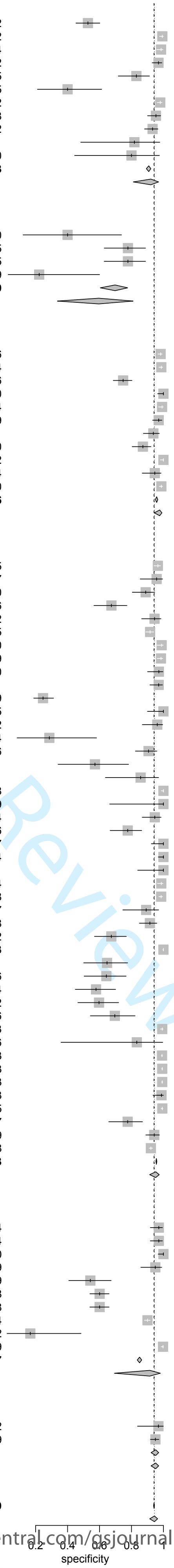


Heterogeneity: $I^2$ = 96%, $\tau^2$ = 3.9819, $p$ = 0
Test for subgroup differences (common effect): $\chi^2_5$ = 517.21, df = 5 ($p$ < 0.01)
Test for subgroup differences (random effects): $\chi^2_5$ = 25.11, df = 5 ($p$ < 0.01)

For Peer Review

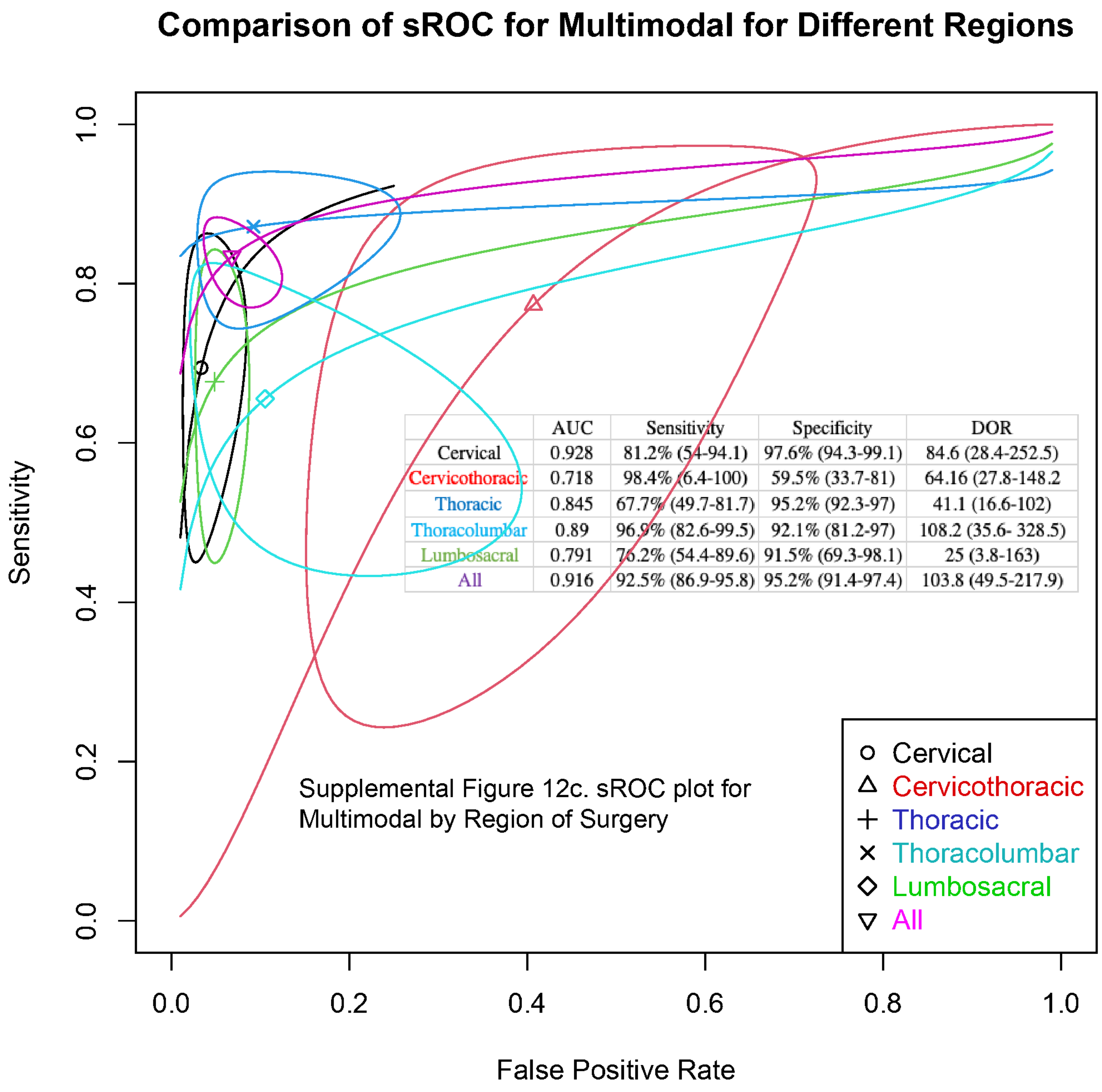


| | AUC | Sensitivity | Specificity | DOR |
|---|---|---|---|---|
| Cervical | 0.928 | 81.2% (54-94.1) | 97.6% (94.3-99.1) | 84.6 (28.4-252.5) |
| Cervicothoracic | 0.718 | 98.4% (6.4-100) | 59.5% (33.7-81) | 64.16 (27.8-148.2 |
| Thoracic | 0.845 | 67.7% (49.7-81.7) | 95.2% (92.3-97) | 41.1 (16.6-102) |
| Thoracolumbar | 0.89 | 96.9% (82.6-99.5) | 92.1% (81.2-97) | 108.2 (35.6- 328.5) |
| Lumbosacral | 0.791 | 76.2% (54.4-89.6) | 91.5% (69.3-98.1) | 25 (3.8-163) |
| All | 0.916 | 92.5% (86.9-95.8) | 95.2% (91.4-97.4) | 103.8 (49.5-217.9) |

Supplemental Figure 12c. sROC plot for Multimodal by Region of Surgery
